# The impact of prioritisation and dosing intervals on the effects of COVID-19 vaccination in Europe: an agent-based cohort model

**DOI:** 10.1038/s41598-021-98216-0

**Published:** 2021-09-22

**Authors:** Martí Català, Xintong Li, Clara Prats, Daniel Prieto-Alhambra

**Affiliations:** 1 Centre for Comparative Medicine and Bioimage (CMCiB), Gemans Trias i Pujol Research Institute (IGTP), Badalona, Spain; 2 Physics Department, Computational Biology and Complex Systems (BIOCOM-SC), Universitat Politècnica de Catalunya, Barcelona, Spain; 3 Nuffield Department of Orthopaedics, Rheumatology and Musculoskeletal Sciences (NDORMS), University of Oxford, Oxford, UK

**Keywords:** Diseases, Infectious diseases, Viral infection

## Abstract

Different strategies have been used to maximise the effect of COVID-19 vaccination campaigns in Europe. We modelled the impact of different prioritisation choices and dose intervals on infections, hospitalisations, mortality, and public health restrictions. An agent-based model was built to quantify the impact of different vaccination strategies over 6 months. Input parameters were derived from published phase 3 trials and official European figures. We explored the effect of prioritising vulnerable people, care-home staff and residents, versus contagious groups; and the impact of dose intervals ranging from 3 to 12 weeks. Prioritising vulnerable people, rather than the most contagious, led to higher numbers of COVID-19 infections, whilst reducing mortality, hospital admissions, and public health restrictions. At a realistic vaccination speed of ≤ 0·1% population/day, separating doses by 12 weeks (vs a baseline scenario of 3 weeks) reduced hospitalisations, mortality, and restrictions for vaccines with similar first- and second-dose efficacy (e.g., the Oxford-AstraZeneca and Moderna vaccines), but not for those with lower first vs second-dose efficacy (e.g., the Pfizer/BioNTech vaccine). Mass vaccination will dramatically reduce the effect of COVID-19 on Europe’s health and economy. Early vaccination of vulnerable populations will reduce mortality, hospitalisations, and public health restrictions compared to prioritisation of the most contagious people. The choice of interval between doses should be based on expected vaccine availability and first-dose efficacy, with 12-week intervals preferred over shorter intervals in most realistic scenarios.

## Introduction

By February 2021, the SARS-CoV-2 virus had caused over 100 million confirmed cases and 2 million COVID-19 deaths globally^1^. Several vaccines have been approved by regulators worldwide, and there are twenty more under investigation^2^. The approved vaccines have showed high efficacy (70·4% for Oxford/AstraZeneca, 95% for Pfizer/BioNTech, and 94·1% for Moderna) in clinical trials, which have tested schedules of two doses given 3 or 4 weeks apart^3–5^. Since 8 December 2020, when the first Pfizer/BioNTech vaccine was given to an elderly woman in the UK, many countries have started mass immunisation programs.

Given constraints in vaccine supply and healthcare workers, vaccine strategies should be optimised to make full use of available resources. One option is to prioritise certain groups for early vaccination. The UK’s Joint Committee for Vaccination and Immunisation (JCVI) created a list of priority populations that includes care-home residents and their caregivers, those over 80 years old, and frontline care workers^6^. Indonesia has prioritised young and middle-aged adults as they are more likely to contribute to community transmission^7^.

In parallel, many experts have proposed modifying vaccination schedules, despite uncertainty surrounding the effect of using longer dosing intervals than those studied in clinical trials. From 30 December 2020, the UK Department of Health moved from the previously proposed and trialled interval of 3 weeks to 3 months. This suggestion was informed by an exploratory analysis of the ChadOx1 vaccine by Oxford/AstraZeneca. According to unpublished data cited by the JCVI, one dose of the Oxford/AstraZeneca vaccine provides an efficacy of 73% (95% CI: 48·79 to 85·76) for up to 12 weeks^8^. More than half of the participants in phase 2/3 trials for the Oxford/AstraZeneca vaccine had their two doses separated by > 12 weeks, which further supported this change^9^. The effectiveness and safety of other approved vaccines beyond the trialled interval (3 and 4 weeks for the Pfizer/BioNTech and Moderna vaccines, respectively) is not well-understood. However, reduced availability due to logistical challenges remains an issue with these vaccines^10^. Public health officials must decide whether to vaccinate more people quickly, possibly with reduced effectiveness, or vaccinate more slowly speed and maintain the high, proven efficacy.

In the meantime, new variants of the coronavirus are emerging. While it is not yet known whether these variants cause more serious illness, the UK, South Africa, and Brazil variants may be more contagious. For example, the UK variant may be 56% more transmissible than the pre-existing SARS-CoV-2^11–13^. Vaccine efficacy against the new variants and their potential impact on vaccine programmes remain unclear.

We aimed to answer two strategic questions related to planning European COVID-19 immunisation campaigns while vaccine availability is limited. We modelled the effect of prioritising vulnerable groups or the most contagious groups for early vaccination. We also studied the effect of the original (3- or 4-week) and extended (12-week) dose intervals on infections, hospitalisations, mortality, and public health restrictions. All analyses were conducted for Europe and for each European country separately.

## Methods

### Study design: overview of the agent-based model

We used an age-structured agent-based model based on the susceptible-exposed-infectious-recovered (SEIR) framework. Agent-based models have been widely used to model infectious diseases, including COVID-19^14,15^. We built a bespoke agent-based model that includes vaccination and the effect of public health restrictions- non-pharmaceutical interventions (NPI), which includes lockdowns, social distancing, and other measures intended to reduce the virus transmission.

**Figure 1:**
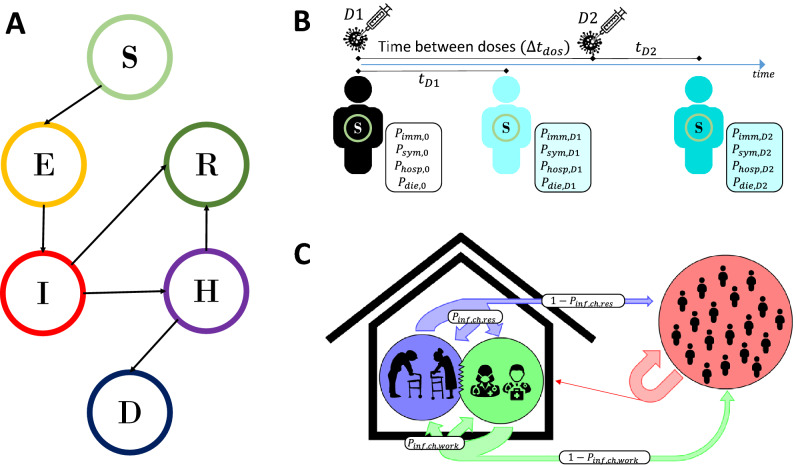
Overview of the agent-based model, showing (**A**) the main individual states and transitions between them; (**B**) the vaccination model: 2-dose regimen and individual variables affected by each dose; and (**C**) care homes: residents, workers, and potential visitors. S = Susceptible, E = Exposed, R = Recovered, I = Infected, H = Hospitalised, D = Dead.

The model accounts for the different states that an agent can acquire in the infection process (susceptible, non-infectious exposed, symptomatic/asymptomatic infectious, hospitalised, recovered, and dead), agent vaccination status, and transitions between these states (Fig. 1). Each agent is assigned personal characteristics, such as age, sex, or time in each state. Transition probabilities between states depend on individual features and vaccination status. Reported COVID-19 deaths were used to determine initial states conditions^16^.

The infection process is based on an individual-level reproduction number that provides a particular contagiousness to each infectious agent. This property is individually assigned and temporarily reduced after restrictions. The times that determine an agent’s infection journey (e.g., incubation time, contagiousness period, and sickness duration) are individually assigned using appropriate distributions. Agents can also be assigned to live in, work at, or visit a care home. Appendix 1 gives all model details.

Distributions of age and people living and working in care homes follow that of the European population and each European country. The probabilities of showing mild symptoms, developing severe symptoms that require hospitalisation, and dying are assessed using UK-based real-time community coronavirus testing, authority-released data on hospitalisation and death, and previous studies^17–19^.

Vaccination and restrictions can change agents’ individual properties. Vaccination modulates susceptibility to infection and the probability of developing symptoms, being hospitalised, and dying. We used published phase 3 trial findings for three available vaccines in Europe for our simulations (Appendix 1 Table 2)^3,4,20^.

The model does not explicitly describe NPI. Instead, we introduced the effects of a certain set of NPI as the resulting reduction in agents’ contagiousness. This NPI level can be held constant or varied during the simulated period, depending on the epidemiological situation.

### Effect of non-pharmaceutical interventions

We translated the presence or absence of NPI, or public health restrictions, into a parameter that reduces agents’ infectivity. This indicator, λ_NPI_, can range between 0 (no restrictions, λ_min_) and a maximum value, λ_max_. It modifies the individual probability to infect as follows:

$$ P_{\inf } = P_{\inf ,0} \cdot \left( {1 - \lambda_{NPI} \left( t \right)} \right), $$

where P_inf,0_ is the individual probability to infect in the absence of restrictions. λ_NPI_ can acquire different values during the simulation, accounting for periods with and without NPI. The level of NPI can be determined from the situation in hospitals, which would activate or release measures in an automated manner (see Supplementary material 1 for details on this sub-model).

### Vaccination strategies

We assumed a steady vaccination rate, r_vac_, over the simulation period, defined as the percentage of the total population at risk who receive one dose per day. We compared four vaccine prioritisation strategies:

A. Vulnerable: People with a higher probability of death are vaccinated first, with mortality risk estimated based on published data^17,19^.
B. Age-based: People are vaccinated in descending order of age.
C. Contagiousness: individuals with higher cumulative probability to infect in absence of restrictions (R_ind_) are vaccinated first.
D. Random: Vaccination is randomly allocated in the population.

We also considered a situation in which care-home residents are vaccinated first, followed by the groups prioritised according to the strategies above. The effect of care-home residence status on outcomes was based on previous publications^21^ and is detailed in Appendix 1.

### Vaccine dose intervals

All three COVID-19 vaccines approved in Europe to date require two doses, with intervals tested in trials varying from 3 to more than 12 weeks. Different levels of efficacy are expected during the time between the first and second dose^3,4,20^. The efficacy of the first dose is expressed as a percentage of the efficacy after receiving two doses.

In our models, first and second doses are separated by Δt_dos_ days. As the default separation depends on the most common interval used in phase 3 trials, it varies by vaccine (Appendix 1 Table 3). Individuals are vaccinated independently of their infection state. However, vaccination is only effective if the individual remains in the susceptible state after the effectivity time (t_1D_ and t_2D_). Vaccines are considered effective during the full simulation (T_max_).

### Simulation outcomes

In each simulation, we monitored the number of individuals in each infection and vaccination state, restriction levels, fraction of care-home residents who are exposed or infected, care-home residents and staff COVID-19 infection history, and the percentage of care homes that have active COVID-19 cases. At the end of each simulation, we obtained the cumulative incidence of deaths, the cumulative incidence of infection, the maximum number of hospitalised cases, the day when this maximum was achieved, the day when the maximum number of infections occurred, and the AUC, estimated as the area under the percentage of restriction/s intensity (y-axis) curve and duration in days (x-axis) over the study period. We also obtained the total number of deaths in care homes, the number of care homes that had COVID-19 cases, the fraction of care-home workers that were infected, the fraction of care-home residents that were infected, and the day with the maximum number of COVID-19 active cases in care homes.

### Model implementation and simulation scheduling

The model is implemented in Matlab and R. The Matlab code is available from https://github.com/catalamarti/ABM_vaccination. Input parameters can be modified in the main file (main.m), and country-level data can be uploaded to populations5.xlsx in the same repository. We have released a user-friendly web application (https://lizncu.shinyapps.io/abm_vaccine) that allows users to choose a country and change the simulation parameters to incorporate future vaccines and reflect future more transmissible and/or lethal variants of the virus.

Each simulation represents the system dynamics for T_max_ days. The time step is 1 day, and a fixed population of 100,000 (N_ind)_ individuals is simulated. We used T_max_ = 6 months to determine how the vaccination strategy and restrictions modified the agents’ probability in each status and the resulting population-level outcomes. The total population, including dead agents, is kept constant. We performed 100 repetitions for each simulation to ensure statistically reliable results.

### Sensitivity analyses

We designed a one-at-a-time parameter sensitivity analysis, performing 20 simulations for each of the 23 parameters to explore 20 equispaced points inside parameter ranges shown in Appendix 1 Table 3. Other parameters were fixed to their default value.

We also performed a full sensitivity analysis using 1000 simulations in the parameter space. We chose appropriate random points using Latin hypercube sampling. Two conditions were imposed. The probability of a certain individual being hospitalised or dead if symptomatic must be between 0 and 1, which entails a correlation between f_S_, f_H_, f_D_, and f_R_. The vaccine effectivity in severe individuals must be equal to or higher than that in symptomatic cases, which must be equal to or higher than that in asymptomatic cases, which entails a correlation between D_2,asy_, D_2,sym_, and D_2,sev_.

### Ethics approval

Only public aggregated data was used for this study. The use of such data does not require ethics approval.

## Results

Our simulations showed that, as expected, vaccination reduced hospital admissions, mortality, and restrictions compared with no vaccination in all scenarios. The effect size and temporality depended on the vaccine and tested parameters.

**Figure 2:**
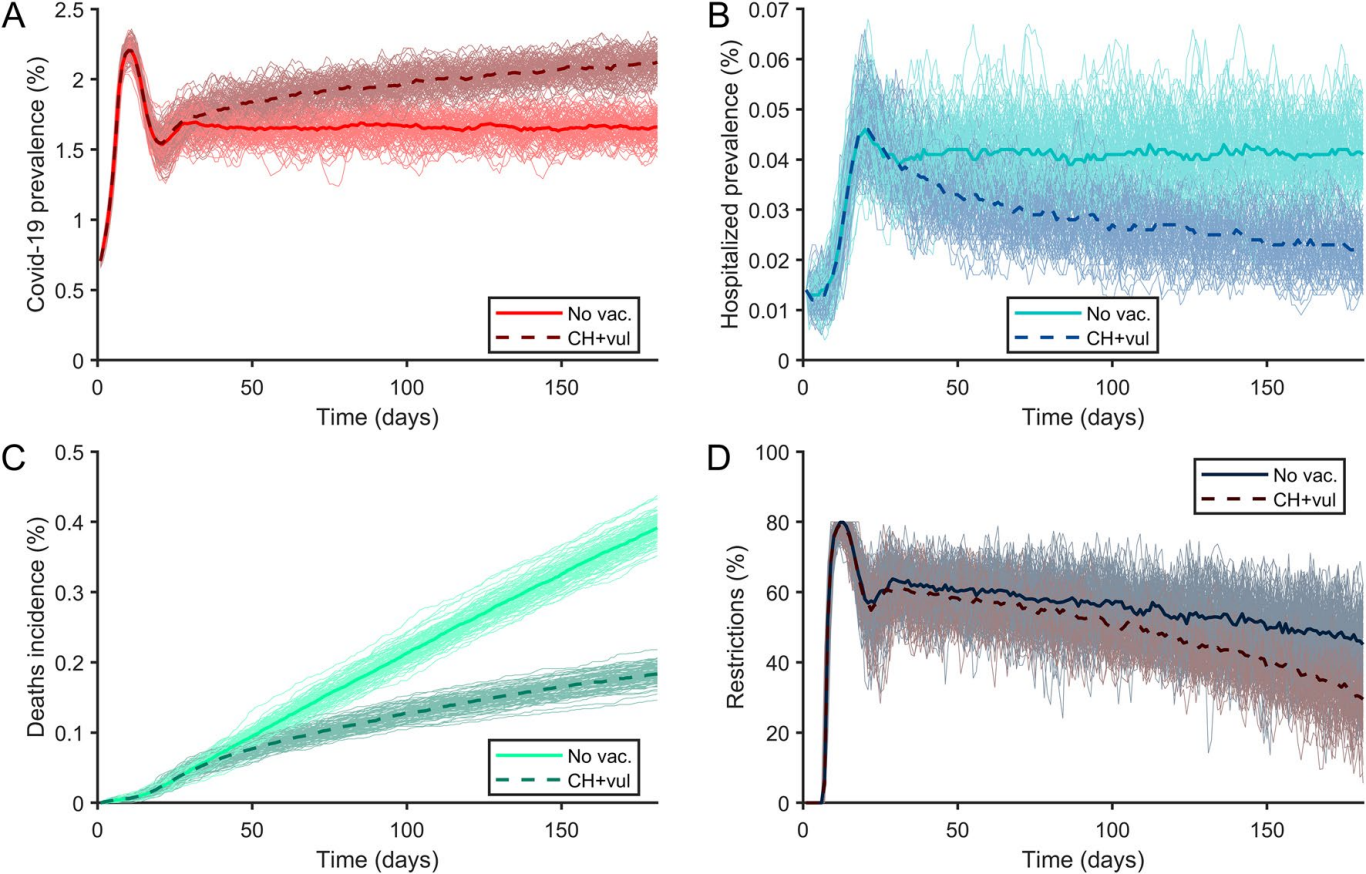
Cumulative incidence of (**A**) infection, (**B**) hospitalisations, and (**C**) mortality, and (**D**) the resulting impact on public health restrictions from the agent-based model for the impact of COVID-19 in Europe without vaccination (No vac.) and when vaccinating with the Oxford/AstraZeneca vaccine with a 4-week separation between doses, when prioritising care-home residents and vulnerable people (CH + vul).

**Figure 3:**
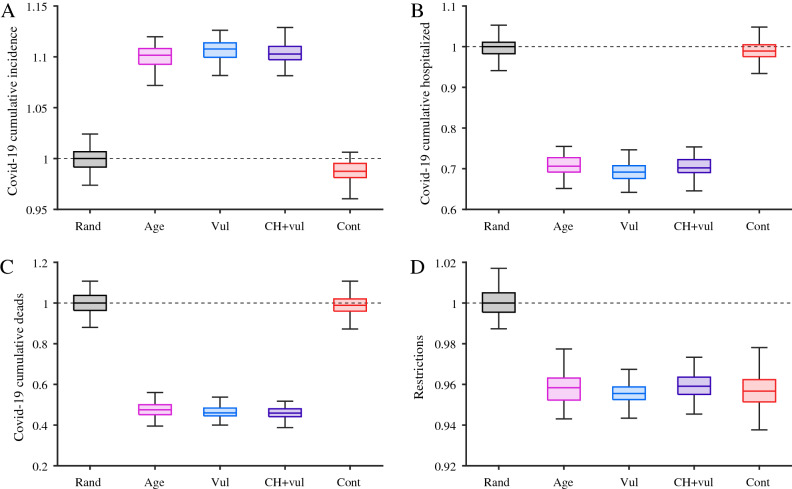
Cumulative incidence of (**A**) infection, (**B**) hospitalisations, and (**C**) mortality, and (**D**) the resulting impact on public health restrictions, according to prioritisation strategy: random (Rand), contagiousness (Cont), age-based (Age), vulnerable (Vul), and vulnerable and care-home residents (CH + Vul). Values are expressed as the ratio between each variable in the vaccination scenario and using random vaccination strategy.

**Figure 4:**
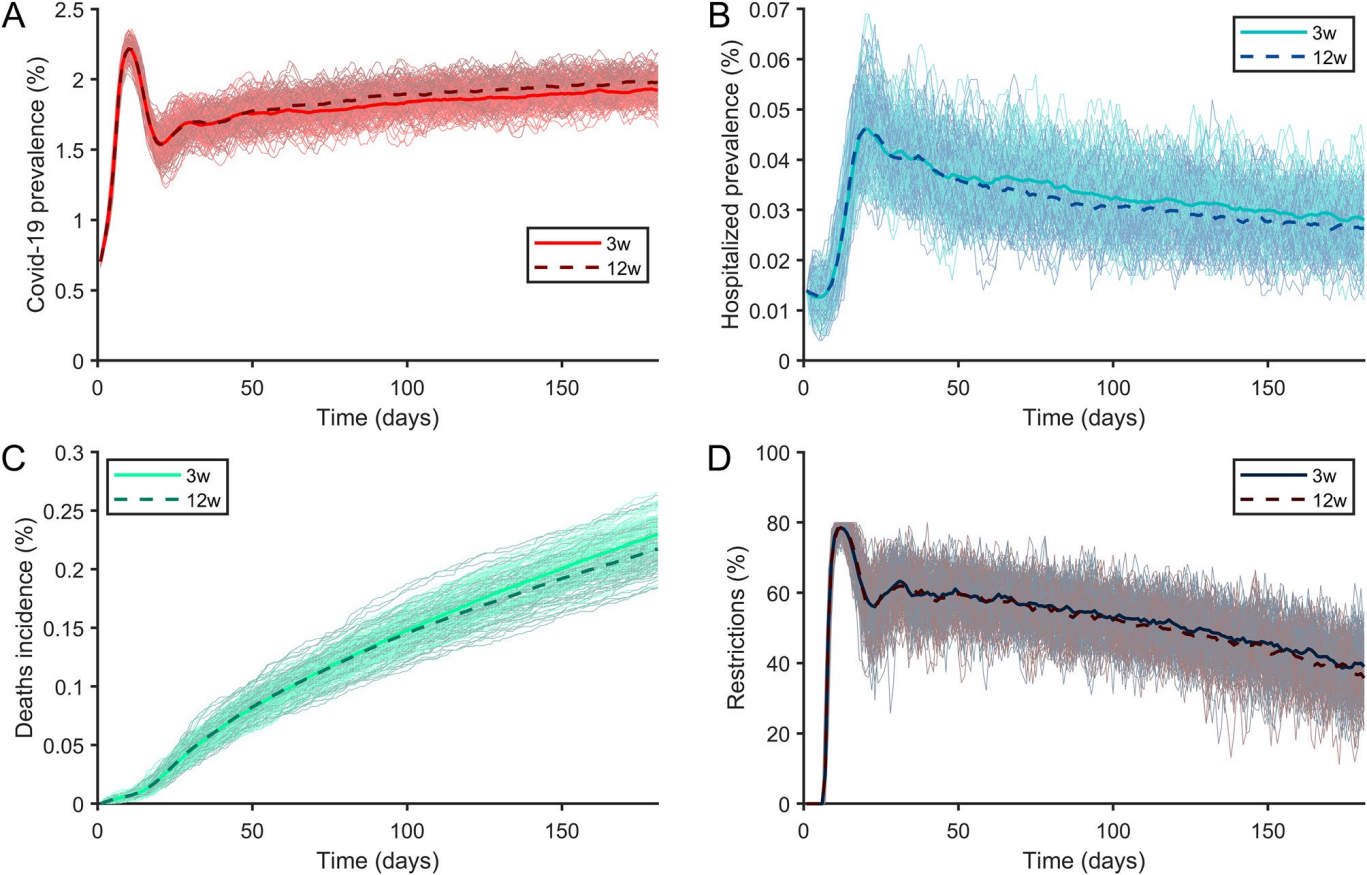
Outcomes of cumulative incidence of (**A**) infection, (**B**) hospitalisations, and (**C**) mortality, and (**D**) the resulting impact on public health restrictions using Oxford/AstraZeneca vaccine with between-dose intervals of 3 or 12 weeks. Each light curve is a single run of the same set of parameters, according to its colour. Each thick curve is the average of the 100 single runs.

Figure 2 shows the effect of a base case vaccination strategy using the Oxford/AstraZeneca vaccine, prioritising vulnerable people and care-home residents, with a 4-week dose interval, and a vaccination rollout speed of 0.2% of the base population per day. This strategy led to observable reductions in mortality and hospital admissions, with benefits increasing over time. The duration and intensity of restrictions therefore reduced compared with no vaccinations. However, when lowered hospital pressure caused a relaxation of restrictions, the number of mild cases increased in some scenarios (Fig. 2A). Appendix 2 shows the results for each European country, adjusted for national figures and socio-demographics. Appendix 4 Figs. 1–4 shows the results when vaccination speed and hospital capacity were varied.

### Prioritisation strategy

Figure 3 shows simulations of the vaccine prioritization schedules. Quantities are shown as boxplots of 100 simulations and renormalized with respect to the mean random prioritization final values. Prioritising the most contagious populations led to the lowest COVID-19 prevalence when using the base-case scenario (Oxford/AstraZeneca vaccine, 4-week dose interval, and vaccination speed of 0·2% per day), with 5% fewer of the population infected than with random allocation and an 11% reduction in the AUC for restrictions. Prioritising care-home residents and vulnerable people led to the lowest proportion of hospitalisations and deaths, reducing deaths by 53% and hospitalisations by 28% compared with random vaccination. Restrictions were again minimised, with the AUC reduced by 11%.

Comparing these two best strategies showed that prioritising the most contagious resulted in 11% less infections than prioritising vulnerable people and care-home residents. However, the latter led to 42% less hospital admissions and 54% less mortality. The two strategies resulted in similar levels of restriction. Note that a lower level of hospitalizations, as the one observed for the scenario where vulnerable people and care-home residents are prioritised, may permit a lower level of restrictions and, therefore, can entail a higher cumulative incidence.

Appendix 4 Figs. 6–15 shows how these results changed with different vaccination speeds and hospital capacities. Based on these findings, we used prioritising vulnerable people and care-home residents as the base-case strategy for studying dose intervals.

### Dosing interval

The effect of interval dosing on the prevalence of infection, percentage hospitalised and dead, and NPI restrictions depended on first-dose efficacy and vaccination speed. We compared dose intervals of 3 and 12 weeks using published phase 3 trial data for the Oxford/AstraZeneca vaccine^4^, which suggested similar first- and second-dose efficacy, and a realistic vaccination speed of 0·1% of the population per day, based on European vaccination uptake data^22^. The 12-week interval resulted in 2% higher COVID-19 prevalence, but a 3% reduction in hospital admissions, 4% reduction in mortality, and 1% reduction in the AUC of restrictions (Fig. 4). Appendix 4 Figs. 16–20 shows results obtained using different vaccination speeds and hospital capacities.

Similar results were seen when modelling the Moderna vaccine with 80% efficacy after one dose, 95% efficacy after two doses^20^, a sustained efficacy between the doses regardless of interval duration, and a vaccination speed of 0·1% of the population per day (Appendix 4 Fig. 21). Appendix 4 Figs. 22–25 show results with variations on vaccination speed and hospital capacity.

Phase 3 trials have shown that the Pfizer/BioNTech vaccine has lower first-dose efficacy (52%) than second-dose efficacy (95%)^3^. Modelling this vaccine with a vaccination speed of 0·1% of the population per day and a 12-week interval resulted in a small reduction in prevalence, 12% increase in hospital admissions, 3% increase in mortality, and a greater need for restrictions (Appendix 4 Fig. 26, with variations in Appendix 4 Figs. 27–30).

**Figure 5:**
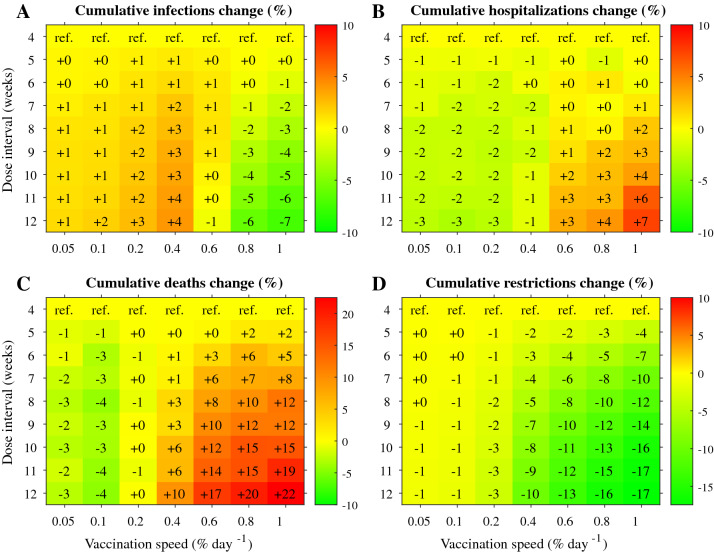
Heatmaps showing the impact of dose intervals and vaccination speed on (**A**) infections, (**B**) hospital admissions, (**C**) mortality, (**D**) and the AUC of non-pharmaceutical public health restrictions, when using the Oxford/AstraZeneca vaccine efficacy data. A 4-week dose interval was used as a reference, and all changes are given as percentages with respect to the reference scenario. Default parameters are gathered in Table 1 of Appendix 1.

Vaccination speeds of under 0·2% of the population per day favoured the 12-week interval over the 3-week interval for the Oxford/AstraZeneca (Fig. 5) and Moderna (Appendix 4 Fig. 39) vaccines, with clear reductions in hospitalisations, mortality, and restrictions with the longer interval. These reductions disappeared when vaccinating ≥ 0·4% of the population per day. Longer dose intervals again did not improve hospital admissions, mortality, or restrictions for the Pfizer/BioNTech vaccine (Appendix 4 Fig. 40).

Appendix 3 shows sensitivity analysis results. They support the robustness of our findings, whilst demonstrating the expected impact of many model parameters (R0, hospital capacity, acceptable restrictions, vaccine efficacy, etc.) on the key study outcomes. Simulation outcomes were most affected by parameters related to restrictions, such as disease time, maximum restrictions, and hospital capacity.

One of the most important outcomes that must be considered is the effect that each parameter has on NPI restrictions. In the simulations, restrictions are automatized to assure not to surpass hospitals capacity. For example, in supplementary material 3 (Figs. 2.20 and 4.8) we can observe the effect of changing the disease time. If the disease time (t_sev_) is increased, the cumulative incidence, total number of deaths and hospitalizations are reduced. This contra intuitive result is due to an increase in NPI restrictions that is triggered by the increase in disease time that, consequently, favours a decrease in the number of cases, total hospitalizations and deaths. Another interesting effect to observe is how the different parameters change the effective reproduction number, as it governs the dynamics of the system^23^.

## Discussion

Given Europe’s vaccine shortage and the pressures on its healthcare workforce, decisions need to be made to maximise the benefits of COVID-19 vaccination on health and the economy. Using an agent-based model informed by current vaccine efficacy evidence and up-to-date COVID-19 disease dynamics parameters, we demonstrated that prioritising vulnerable populations for early vaccination most effectively prevented hospitalisations and mortality and minimised public health restrictions. Our results suggest that optimal dosing intervals should be informed by vaccine efficacy data and achievable vaccination speeds. We found that with the current slow vaccination rates (≤ 0·1% population/day) in most European countries, a longer (12-week) interval would preferrable for vaccines with similar first- and second-dose efficacy (e.g., Oxford/AstraZeneca and Moderna), whilst shorter (3-week) intervals would be preferrable for vaccines with lower first-dose efficacy than second-dose efficacy (e.g., Pfizer/BioNTech).

Mathematical modelling has been widely used for studying infectious disease transmission, including COVID-19, and planning pharmaceutical and non-pharmaceutical interventions^24–28^. However, most reports to date have focused on one component of vaccine strategies, such as population prioritisation or dose schedule. In general, studies on prioritisation groups have found that vaccinating elderly populations first maximises the expected reductions in hospitalisations and mortality, while vaccinating the most contagious groups first reduces total cases^26,29–31^.

Our findings generally align with previous studies. We found that vaccinating vulnerable people and care-home residents maximised the benefits of COVID-19 vaccination on hospital admissions, mortality, and restrictions. Similarly, we found that prioritising the most contagious groups reduced the prevalence of COVID-19. Both strategies have been applied widely. High-income countries, including the US and Europe, have prioritised the vulnerable and/or elderly, while low- and middle-income countries have tended to target the most contagious populations. These choices are related to the type of vaccines authorised, population structure, and country strategies. Prioritising vulnerable people results in fewer hospitalisations and deaths and minimises restrictions when these are triggered in response to hospital pressure, which are all factors contributing to the COVID-19 pandemic’s effect on the European economy^32,33^. However, although minimising severe disease by vaccinating vulnerable people makes sense, the long-term effects of COVID-19 in young people^34,35^ and potential productivity losses should be considered as more data emerge.

Consensus has not yet been reached on the optimal vaccine dosing regimen. There is little evidence on the effects of different dose intervals from those tested in COVID-19 vaccine phase 3 trials. Supporters of extended dosing intervals cite data from manufacturers’ briefing documents, which suggest that the efficacy after dose one was 52·4% (95% CI: 29·5% to 68·4%) of the efficacy after dose two for the Pfizer/BioNTech vaccine and 80.2% (95% CI: 55·2% to 92·5%) for the Moderna vaccine^5,36,37^. We used these efficacy data as the base values in our model and explored expanded ranges in sensitivity analyses. Our results suggest that increasing the dosing interval only reduces hospitalisations and mortality when the difference between first- and second-dose efficacy is small (a ratio of 90% or higher compared with full dose). This cut-off varied with vaccination speed. Longer 12-week intervals only appeared preferrable to 3–4 week schedules in most realistic scenarios when vaccination was slow (≤ 0·1% population/day).

Recent studies on dosing schedules have drawn similar conclusions. Salomon and colleagues reported that a “flexible” strategy of gradually giving the second dose over the next 8 weeks after the first dose could avert an additional 23–29% of COVID-19 cases in the US, assuming a first-dose efficacy of 52·4% and a second-dose efficacy of 94·8%^25^. Paltiel et al. found that a single-dose vaccine of 55% or higher efficacy may benefit more than a two-dose regimen with 95% efficacy^38^. Another study using Oxford/AstraZeneca vaccine data showed that one dose was preferable to two doses if the single dose was at least 75% as efficacious as the double dose^39^. Similar findings have also been reported using a US-based model, where a single-dose strategy was preferred when efficacy was 72% or more^40^.

However, there is still notable ambiguity on the efficacy after the first dose. Emerging evidence has suggested that the first dose of Pfizer/BioNTech vaccine could reach around 90% efficacy after day 21, which is higher than the value in our base-case simulation^8,41^. The effects of the Pfizer/BioNTech vaccine would then be similar to the Moderna vaccine, with 12-week intervals resulting in reduced hospitalisations and little-to-no notable effects on mortality (Appendix 4 Figs. 31–33 and 41).

Our study has several limitations and is based on strong assumptions. We assumed that vaccination rollout was constant during the 6-month period. Nevertheless, the simulations provided are aimed at helping to decide the best vaccination strategy, rather than predicting the outcome of the epidemic in a specific country which, in addition, will depend on many other factors. The steady vaccination rate is a simplification that should not affect the main conclusions about the best prioritization strategy, since it is used to compare different vaccination velocities. Moreover, improvements in the production of existing vaccines and approval of new vaccines are likely to affect our findings. Greater vaccination availability is expected to lead to faster rollout, minimising the benefits of longer between-dose intervals.

We assumed sustained efficacy between the two doses, regardless of interval duration. Evidence is scarce on how long the first dose’s benefit will persist beyond day 21 (for Pfizer/BioNTech) or 28 (for Moderna). Waning efficacy is likely to favour shorter dose intervals, whereas increasing efficacy over time is likely to strengthen the value of longer dose intervals. Recent data suggest that the latter is true for the Oxford/AstraZeneca vaccine^42^, reinforcing the longer interval strategy.

We did not explicitly account for some potentially relevant parameters, including effects on household clusters, the effect of pre-existing health conditions in age-structured disease dynamics probabilities, and the duration of acquired (post-infection) and vaccine-induced immunity. However, we conducted multiple sensitivity analyses that confirmed the robustness of our findings and showed that the R_0_, hospital capacity, and vaccine efficacy affect the study outcomes. Further research is needed to understand how new variants might change transmissibility, severity, and vaccine efficacy.

Our study also has major strengths. Although limited information is available about vaccine strategies, we used country-specific population characteristics to build models for each European country. Our country-specific results can be used by decision-makers to inform national vaccination strategies.

We explicitly included care-home residents and staff in our models, which had not been done in previous models. Those who live in care homes have higher infection rates and are at greater risk of severe disease and death than the rest of the population^21,43,44^.

We provide an interactive web application for studying the effect of different vaccination strategies in different moments of the pandemic, using national figures for each and all European countries (https://lizncu.shinyapps.io/abm_vaccine). Once priority groups have been vaccinated, our web app can be used by policy makers to test potential next steps. Data from non-European countries, sociodemographic structures and epidemiological situations, can be added to the app to extend its use.

In summary, our study suggests that prioritising vaccinating vulnerable people and care-home residents will reduce hospitalisations, mortality, and public health restrictions. We have demonstrated that the choice of dose interval should be informed by vaccine efficacy data and availability. Future studies on single-dose or delayed-dosing strategies are needed to design better-informed vaccination campaigns.

## Supplementary Information

Supplementary Information 1.

Supplementary Information 2.

Supplementary Information 3.

Supplementary Information 4.

## Data Availability

All code used in this study is publicly available at https://github.com/catalamarti/ABM_vaccination. A user-friendly web application for exploring parameters changes can be found at https://lizncu.shinyapps.io/abm_vaccine.
