# Supplementary material for: The impact of prioritisation and dosing intervals on the effects of COVID-19 vaccination in Europe: an agent-based cohort model
Source: Sci Rep. 2021 Sep 22;11:18812. doi: 10.1038/s41598-021-98216-0 (PMC8458447; doi:10.1038/s41598-021-98216-0)
Supplement: Supplementary file 1 — Supplementary Information 1. [file 41598_2021_98216_MOESM1_ESM.pdf]

The impact of prioritisation and dosing intervals on the effects of COVID-19 vaccination in Europe: an agent-based cohort model

Martí Català, Xintong Li, Clara Prats*, Daniel Prieto-Alhambra

* clara.prats@upc.edu

SUPPLEMENTARY MATERIAL: Model description

Basic entities and processes

We have built an ad-hoc Agent-Based Model where we included the vaccination process and the effect of non-pharmaceutical interventions (NPI). The model accounts for the different states that an agent can acquire with regards to the infection process: susceptible, non-infectious exposed, symptomatic/asymptomatic infectious, hospitalized, recovered and dead.

The basic transitions between these states are modelled as follows:

1. Exposed individuals (E) become infectious (I) after the incubation period is completed (t_{inc}).
2. Infectious individuals become recovered (R) or hospitalized (H) after the infectiousness period is completed (t_{con}).
3. Hospitalized (H) may recover (R) or die (D) after the severe disease period is completed (t_{sev}). Hospitalized agents are not considered to be contagious.
4. Infectious individuals cause new infections with a certain probability that depends on each individual (R_{ind}). In case that new infections occur, corresponding susceptible agents (S) become exposed (E).
5. Recovered and dead individuals remain in their state. Recovered cannot be infected again during the simulation period.

All agents have a set of 13 individual properties and variables, which are:

- Instantaneous status within the infection process (S, E, I, R, H, D).
- Age and sex. Age is set in 5-year-old ranges 90 years old (0-4, 5-9, …, 85-89, >=90). As for assigning the sex and age range to the agents, we use the European population pyramid as default (supplementary material table 1).(1) Nevertheless, the model is prepared to upload and apply any European country pyramid or to implement a personalized pyramid.(1)
- Status with regards to the vaccination process: non-vaccinated (NV); vaccinated with an effective dose (D1), and vaccinated with an effective second dose (D2), First dose is assumed to be effective after t_{1D} days, while the second dose is assumed to be effective the day after the administration. Initially all individuals are non-vaccinated.
- Individual contagiousness index (R_{ind}). This index assesses how many people are going to be infected by a certain agent. Given a certain community basic reproduction number (R_0), the individual contagiousness index is chosen from a homogeneous distribution between 0 and $2 \cdot R_0$.
- Symptomatic or asymptomatic. The probability to be symptomatic (P_{sym}) depends on age, as seen in supplementary information table 1.(2–4) Probability of being symptomatic is multiplied by an overall pre-factor (f_S) in order to increase or decrease the default probabilities, keeping constant their profile with age. There are three values

for the probability to be symptomatic: without vaccine, with first effective dose vaccine, and with vaccine full effect. Symptomatic state does not affect the infection probability.

- Hospitalized or not, if symptomatic. The probability to be hospitalized (P_{hos}), if infected, depends on individual characteristics (age and sex), as shown in supplementary information table 1. Hospitalization probabilities were obtained from the coronavirus data dashboard released by Public Health England and reports by the office of National Statistics.(2–4) Males probability was multiplied by 1.30 and females by 0.77 in order not to change global probability and reproduce male-female ratio in observations.(1,3,5) Hospitalized probability is multiplied by an overall pre-factor (f_H) in order to increase or decrease the default probabilities, keeping constant the age-dependence profile. There are three values for hospitalization probability, depending on the vaccination state: without a vaccine, with first effective dose vaccine, vaccine full effect.

- Dead or not, if hospitalized. The probability to die (P_{die}), if infected, depends on individual characteristics (age and sex), as seen in supplementary information table 1. Death probabilities were obtained from the UK coronavirus data dashboard combined with the population level infection rates.(2) Males probability was multiplied by 1.30 and females by 0.77 in order not to change global probability and reproduce male-female ratio in observations.(1,3,5) Death probability is multiplied by an overall prefactor (f_D) in order to increase or decrease the default probabilities. There are three values for dying probability, according to the vaccination status: without vaccine, with first effective dose vaccine, vaccine full effect.

- The incubation period if infected, i.e., time between the agent is exposed to virus and they become contagious ($t_{inc,ind}$). This individual parameter is chosen from a zero-truncated Poisson distribution with $\lambda = t_{inc}$. Its minimum value is one day.

- The infectiousness period if infected, i.e., the time during which an infectious agent remains contagious ($t_{con,ind}$). This individual parameter is chosen from a zero-truncated Poisson distribution with $\lambda = t_{con}$. Its minimum value is one day. This time is also used for assessing the basic average number of infections that one individual may produce per day (i.e., in absence of control measures), if infectious ($P_{inf,0}$), which is approximated as $R_{ind}/t_{con,ind}$.

- The hospitalization duration if hospitalized, i.e., the time during which individuals are in hospital with a severe disease ($t_{sev,ind}$). This period follows a negative binomial distribution with r=3 and p = 3/(3+t_{sev}), where t_{sev} is the expected mean time of stay in hospital. Its minimum value is one day.

- Each individual can live in a care home, work there or be a potential visitor. Individuals that live or work in a care home are bound to one particular care home.

Agents can be infected only if they are susceptible. If vaccinated, agents have a certain probability of avoiding the infection.

Supplementary material. Table 1. Europe's average population pyramid. Mean probabilities of being symptomatic, being hospitalized, dying and living in a care home by age range.(2–4)

Age	Europe's population pyramid		Prob. to be symptomatic (if infected)	Prob. to be hospitalized (if infected)		Prob. to die (x10^4) (if infected)		Prob. to live in a care home
	Male	Female		Male	Female	Male	Female	
0-4	2.68%	2.54%	20%	0.17%	0.10%	0.02	0.01	0.01%
5-9	2.80%	2.65%	20%	0.08%	0.05%	0.01	0.01	0.03%
10-14	2.77%	2.62%	20%	0.08%	0.05%	0.03	0.02	0.12%
15-19	2.60%	2.47%	20%	0.48%	0.28%	0.10	0.06	0.19%
20-24	2.67%	2.54%	25%	0.43%	0.26%	0.32	0.19	0.11%
25-29	3.08%	2.95%	30%	0.53%	0.31%	0.64	0.37	0.11%
30-34	3.55%	3.45%	35%	0.53%	0.31%	1.27	0.75	0.13%
35-39	3.56%	3.52%	40%	0.68%	0.40%	4.89	2.88	0.16%
40-44	3.48%	3.51%	45%	0.68%	0.40%	9.78	5.76	0.20%
45-49	3.44%	3.51%	50%	2.34%	1.38%	15.68	9.23	0.24%
50-54	3.37%	3.52%	55%	2.34%	1.38%	20.64	12.14	0.32%
55-59	3.38%	3.67%	60%	2.85%	1.67%	52.10	30.66	0.39%
60-64	3.04%	3.50%	70%	2.85%	1.67%	72.14	42.45	0.43%
65-69	2.58%	3.18%	70%	7.19%	4.23%	233.18	137.20	0.59%
70-74	2.05%	2.64%	70%	7.19%	4.23%	325.33	191.43	0.91%
75-79	1.39%	1.98%	70%	15.39%	9.05%	660.44	388.60	1.71%
80-84	1.05%	1.77%	70%	15.39%	9.05%	1125.40	662.19	4.04%
85-89	0.54%	1.04%	70%	34.10%	20.06%	2102.80	1237.29	8.97%
≥90	0.24%	0.65%	70%	34.10%	20.06%	4067.37	2393.24	19.98%

All parameters of the model are listed and described in supplementary table 2, together with their default values and explored ranges.

Initial conditions of individuals

The initial status of N_{ind} individuals is chosen randomly from the set of initial possible states, considering the epidemiological situation of the scenario to be simulated. Initially, there are S_0 susceptible individuals, E_0 exposed agents, I_0 infectious agents, H_0 hospitalized and Re_0 recovered. Each country has different initial conditions. In particular, Re_0 (i.e., the amount of people immunized by a previous infection) is computed from the total reported deaths of each country on January 19, 2021 (6).

$$Re_0 = \frac{deaths \cdot N_{ind}}{P_D \cdot population}$$

where P_D is the probability that a random individual will die due to COVID-19 if infected. It is computed using the country's pyramid and death rates reported on supplementary table S1. In the case of Europe as a whole, we assume P_D = 1.22%.

The estimated number of real cases (diagnosed and not diagnosed) that one country had by January 1st 2021 is determined from the average of number of deaths between 8 and 21st January ($deaths_0$). As explained in (7), we can determine the estimated number of cases as:

$$Estimated = \frac{deaths_0}{P_D}$$

This quantity is translated to our simulation into n_0 (initial number of daily cases) that can be determined from the estimated cases by using the quotient between the number of individuals in our simulation (N_{ind}) and the countries' population (Population):

$$n_0 \; = \; Estimated \cdot \frac{Nind}{Population}.$$

From the number of initial daily cases (n_0) and the time that a case is in each state (on average), we can determine the initial number of individuals at each state (E_0, I_0 and H_0):

$$E_0 = n_0 \cdot t_{inc}$$

$$I_0 = n_0 \cdot t_{con}$$

$$H_0 = n_0 \cdot t_{sev} \cdot P_H$$

where P_H is the probability that a random individual will be hospitalized due to COVID-19, if infected. It is computed using the country's pyramid and hospitalization rates reported on supplementary material table 1. In the European case, P_H = 2.64%. Finally, the initial number of susceptible individuals is set as:

$$S_0 \; = \; N_{ind} \; - \; E_0 \; - \; I_0 \; - \; H_0 \; - \; Re_0$$

The initial values for the European scenario are shown in supplementary table 2.

Transmission process

The population-level basic reproduction number (i.e., the average of new contagions per infected individual in absence of control measures) is set to R_0. In addition, we know that these infections can occur during a certain period whose average we name t_{con}. Nevertheless, both the capability to transmit and the period at which these infections can occur depend on the individual. We denote R_{ind} the individual capability of transmission. Therefore, for each individual, we assign a random value for their own transmissibility coefficient (R_{ind}) and contagious time ($t_{con,ind}$). R_{ind} follows a uniform distribution between 0 and 2·R_0. $t_{con,ind}$ follows a zero-truncated Poisson distribution with $\lambda = t_{con}$. Given each individual R_{ind} and $t_{con,ind}$, we can compute the contagiousness probability of a certain individual per day as: $p_{inf,0} = R_{ind}/t_{con,ind}$. These random numbers are sorted before the simulation starts, when initial conditions of individuals are computed. When an individual is in the infected state and without restrictions, they infect a random number of individuals at each step. This number follows a Poisson distribution with $\lambda = p_{inf,0}$.

Each individual infects another random individual without any other restriction except if the individual is assigned to a care home. Individuals that live or work in a care home have an increased probability to infect another individual from the care home, as explained at care homes sections.

Care homes

Infection inside care homes follows a different dynamic than outside. At each simulation, there is a random number of care homes that depends on the number of care homes per million people, r_{nh}. The precise number of care homes is a random Poisson number with $\lambda = \; r_{nh} \cdot$

$N_{ind}/10^6$. Individuals have a probability to live in a care home that depends on age.(8–10) Supplementary information table 1 shows these probabilities. Probability to live in a care home is multiplied by a pre-factor (f_R) in order to increase or decrease the default probability. Agents are assigned to a particular care home at random. Individuals that do not live in a care home have a certain probability to work there if they are between 20 and 59 years old:

$$P = Residents \cdot r_{nh,work} / \ Pop(20 - 59)$$

where residents is the expected number of residents taking into account populations age structure, $r_{nh,work}$ is the number of workers per resident in each country and *Pop(20-59)* is the number of individuals aged between 20 and 59 years old.

Individuals in care homes are not included in hospitalized counts, because it is considered that they receive treatment in the care home. Each worker is assigned to a care home. The probability to be assigned to each care home depends on the number of residents in each care home.

Infection probabilities for people in care homes are different than outside. If a resident is infected, they have a probability to infect an individual from the care home of $P_{inf,nh,res}$. If they do not infect an individual from the care home, they infect a random individual. If a worker is infected, they have a probability to infect an individual from the care home with probability $P_{inf,nh,work}$. Otherwise, they infect a random individual. Figure 1C in the main text shows an outline of the infection model in care homes.

Mortality probability is increased at care homes by f_{NH}, without modifying mean probability to die in each age group.

Vaccination effect

Once a susceptible is vaccinated, their individual properties are modified after a certain period. These time periods are named t_{1D} and t_{2D}, after first and second dose respectively, and are related with the effectiveness dynamics of the vaccine. The vaccination may change the probabilities of being infected, developing symptoms, being hospitalized and dying. These modulations of the individual properties only occur if the agent is symptomatic at the moment when the vaccine is effective. Changes in these probabilities depend on the vaccine that is administered, and are based on the results available for each of them (supplementary material table 2).

A non-vaccinated individual has initially a certain probability to develop symptoms if infected ($P_{sym,0}$), to be hospitalized if infected ($P_{hos,0}$) and to die if infected ($P_{die,0}$). These probabilities depend on the age and sex of this individual. When the individual is not vaccinated, their probability to be immune (P_{imm}) is zero and the probability to be asymptomatic if infected is computed as: $P_{asy,0}$= 1- $P_{sym,0}$. After receiving a vaccine, these probabilities change depending on the effectivity of the first dose with respect to the full dose ($D_{1,eff}$), and the effectivity of the fully vaccinated individuals on asymptomatic ($D_{2,asy}$), symptomatic ($D_{2,sym}$) or against severe forms of the illness ($D_{2,sev}$).

After receiving the first dose of vaccine, these probabilities are re-evaluated as:

$$P_{asy,D1} = P_{asy,0} \cdot \left(1 - D_{1,eff} \cdot D_{2,asy}\right)$$

$$P_{sym,D1} = P_{sym,0} \cdot (1 - D_{1,eff} \cdot D_{2,sym})$$

$$P_{hos,D1} = P_{hos,0} \cdot (1 - D_{1,eff} \cdot D_{2,sev})$$

$$P_{die,D1} = P_{die,0} \cdot (1 - D_{1,eff} \cdot D_{2,sev})$$

$$P_{imm,D1} = 1 - P_{asy,D1} - P_{sym,D1}$$

The same procedure is used to evaluate the individual effect of the second dose, setting $D_{1,eff}$ equal to 1.

Table S3 shows an example of how the individual properties of two agents may change when a vaccine is administered. First, we show the changes produced by Moderna in a 70-74 years old female. Then, we show the modulation of properties in a 50-54 years old male when the AstraZeneca vaccine is administered.

Table 2 Parameters of the model. Description, default value (using Europe's data), and range explored in the sensitivity analysis. Sources are indicated on the last column.

Parameter	Description	Default value	Sensitivity analysis	Source
Simulation				
N_{rep}	Number of repetitions	100		
T_{max}	Simulation time	181		
N_{ind}	Number of individuals	100,000		
P_{pop}	Probability to be a given age and sex	Supplementary table 1		(1)
$P_{pop,nh}$	Probability to live in a care home for a given age and sex	Supplementary table 1		(8–10)
Initial conditions				
S_0	Number of initial susceptible individuals	94,183	$N_{ind} - E_0 - I_0 - H_0 - Re_0$	(6)
E_0	Number of initial exposed individuals	120	$t_{inc} \cdot n_0$ $n_0 \in [10{,}500]$	(6)
I_0	Number of initial infected individuals	360	$t_{con} \cdot n_0$ $n_0 \in [10{,}500]$	(6)
H_0	Number of initial hospitalized individuals	37	$t_{inf} \cdot n_0 \cdot P_h$ $n_0 \in [10{,}500]$	(6)
Re_0	Number of initial recovered individuals	5,300	[1,000-20,000]	(6)
Infection dynamics				
t_{inc}	Time between individual is exposed and becomes contagious. It follows Poisson distribution.	λ =2 days	[1, 6] days	(11)
t_{con}	Time during individual is contagious. It follows Poisson distribution.	λ =6 days	[2, 14] days	(12)
t_{sev}	Time during individual is hospitalized or treated in a care home. It follows a negative binomial distribution with R = 3.	9 days	[5-20] days	(13)
R_0	Basic reproduction number	3	[1.5-5]	(14)

P_{die}	Probability to die (depends on age and sex).	Supplementary table 1		(2–4)
f_D	Increased dead probability factor.	1	[0.5-4]	
P_{sym}	Probability to be symptomatic (depends on age and sex).	Supplementary table 1		(15)
f_S	Increased symptomatic probability factor.	1	[0.5-4]	
P_{hos}	Probability to be hospitalized (depends on age and sex).	Supplementary table 1		(2–4)
f_H	Increased hospitalization probability factor.	1	[0.5-4]	
Care homes				
$P_{inf,nh,work}$	Probability that a worker infects inside care home.	0.6	[0.5-1]	Assumed
$P_{inf,nh,res}$	Probability that a resident infects inside care home.	0.8	[0.5-1]	Assumed
f_R	Increased probability to live in a care home factor.	1	[0.5-2]	
$prop_{nh}$	Number of nurses per resident.	0.2	[0.1-0.3]	(8–10)
f_{NH}	Increased factor to dead inside care home.	2	[0.5-4]	(16)
r_{nh}	Number of care homes per million people.	200	[100-400]	(17)
P_{work}	Probability to work on a care home, if age between 20 and 59.	0.00684	Given by: $P_{pop,nh}$, f_R, $prop_{nh}$, P_{pop}	
Restrictions				
H_{lim}	Limit of hospitalized per million that restrictions will prevent to surpass.	500	[250-1000]	Assumed
λ_{min}	Minimum restrictive level (%)	0%	0%	
λ_{max}	Maximum restrictive level (%)	80%	[60%-90%]	Assumed
Vaccination				
r_{vac}	Vaccinating rate of doses per day as percentage of population.	0.2%	[0.025%-1%]	(6)
$D_{1,eff}$	Effect of 1st vaccine compared to 2nd dose.	Pfizer 55%<br>Moderna 84%<br>AstraZeneca 95%	[30%-100%]	(18–20)
$D_{2,asy}$	Effect of 2nd vaccine dose on asymptomatic.	Pfizer 41%<br>Moderna 41%<br>AstraZeneca 30%	[0%-95%]	(18–20)
$D_{2,sym}$	Effect of 2nd vaccine dose on symptomatic.	Pfizer 95%<br>Moderna 95%<br>AstraZeneca 70%	[55%-99%]	(18–20)
$D_{2,sev}$	Effect of 2nd vaccine dose on severe.	Pfizer 95%<br>Moderna 95%<br>AstraZeneca 90%	[55%-99%]	(18–20)
t_{1D}	Time till 1st dose is effective.	10 days	[7-21] days	(18–20)
t_{2D}	Time till 2nd dose is effective.	1 day	1 day	(18–20)
Δt_{dos}	Separation of both doses.	Pfizer 21 days<br>Moderna & Astra Zeneca 28 days	[21-84] days	(18–20)

Supplementary material. Table 3: Example of change in individual parameters for a 70-74-year-old female vaccinated with Moderna vaccine and a 50-54-year-old male vaccinated with AstraZeneca vaccine. We consider the effect when doses are effective.

Probability	70-74-year-old female Moderna			50-54-year-old male AstraZeneca		
	No vac.	1st dose	Full dose	No vac.	1st dose	Full dose
Immune	0.0%	66.2%	78.8%	0.0%	49.4%	52.0%
Symptomatic	70.0%	14.1%	3.5%	55.0%	18.4%	16.5%
Hospitalized	4.23%	0.85%	0.21%	2.34%	0.34%	0.23%
Death	1.91%	0.39%	0.09%	0.21%	0.03%	0.02%

Effect of NPIs

The presence or absence of restrictive measures is translated into a parameter that reduces the infectivity of agents. This indicator, λ_{NPI}, can range between 0 (no restrictions, λ_{min}) and a maximum value, λ_{max}. It modifies the individual probability to infect as follows:

$$P_{inf} = P_{inf,0} \cdot (1 - \lambda_{NPI}(t))$$

where $P_{inf,0}$ is the average number of infections that one individual produces per day in absence of restrictions. This can acquire different values during the simulation, accounting for periods of NPI followed by periods without interventions. In particular, the level of NPI can be determined from the situation in hospitals, which would activate or release measures in an automated manner.

The automatic restrictions boot uses a simple prediction of the hospitalizations after $t_{inc} + t_{con}$ + 2 days. The number of hospitalized at that day can be approximated by:

$$H_{pred}(t + t_{inc} + t_{con} + 2) = E(t) \cdot P_H' \cdot P_{sev}\left(\frac{t_{inc}}{2} + 2\right) + I(t) \cdot P_H' \cdot P_{sev}\left(\frac{t_{con}}{2} + t_{inc} + 2\right) + H(t) \cdot P_{sev}(t_{inc} + t_{con} + 2)$$

$P_{sev}(d)$ is the probability to remain at hospital after d days, which can be determined assuming a binomial distribution. P_H' is the fraction of people that goes to hospital when infected, which depends on the baseline probability to be hospitalized, P_H, but also on the population vaccinated, as follows:

$$P_H' = \frac{P_H \cdot (N_{ind} - N_{1D} - N_{2D}) + P_{H,1D} \cdot N_{1D} + P_{H,2D} \cdot N_{2D}}{N_{ind}}$$

Where N_{1D} and N_{2D} are the number of agents that have been vaccinated with first and second dose, respectively, when these doses are already effective. $P_{H,1D}$ and $P_{H,2D}$ are the average probabilities to be hospitalized for those that have received first and second dose, respectively. For each country, we fix a maximum number of hospitalizations per million people (H_{lim}). Then, restrictions are computed so that this limit is not surpassed:

$$\lambda_{NPI}(t) = 1 - \frac{\frac{H_{lim} \cdot N_{ind}}{10^6} - H_{pred}(t + t_{inc} + t_{con} + 2)}{\frac{2}{t_{con}} \cdot R_0 \cdot P'_H \cdot P_{sev}(1) \cdot I(t) \cdot \frac{S(t)}{N_{ind}}}$$

Number of simulations

An analysis to determine how the error changes with the number of simulations was carried out. We performed 10,000 simulations with default parameters without the vaccination process and also other 10,000 simulations with the vaccination implemented, using the prioritization of vulnerable people first (CH+vul). From all the simulations, we picked 1,000 times N random simulations to see how the relative error of the median changed for different quantities: cumulative hospitalizations, area under the curve of non-pharmaceutical restrictions, cumulative number of deaths and cumulative incidence as a function of N ($N = 1;\ 10;\ 100;\ 1{,}000$).

As it can be observed in figures 1 (non-vaccination strategy) and 2 (CH+vul strategy), with 100 simulations we can guarantee that the error of the median is less than 2% for the different observed quantities and strategies.

Same strategy is used to determine the 95% confidence intervals for the mean for the studied compartments time evolution (infected, hospitalized, dead and restrictions). In figures 3 (non-vaccination strategy) and 4 (CH+vul strategy) the relative error using different numbers of simulations is shown. As shown in these figures, less than 2% errors are expected for the mean value.

Thus, we conclude that 100 simulations is a sufficient representative sample.

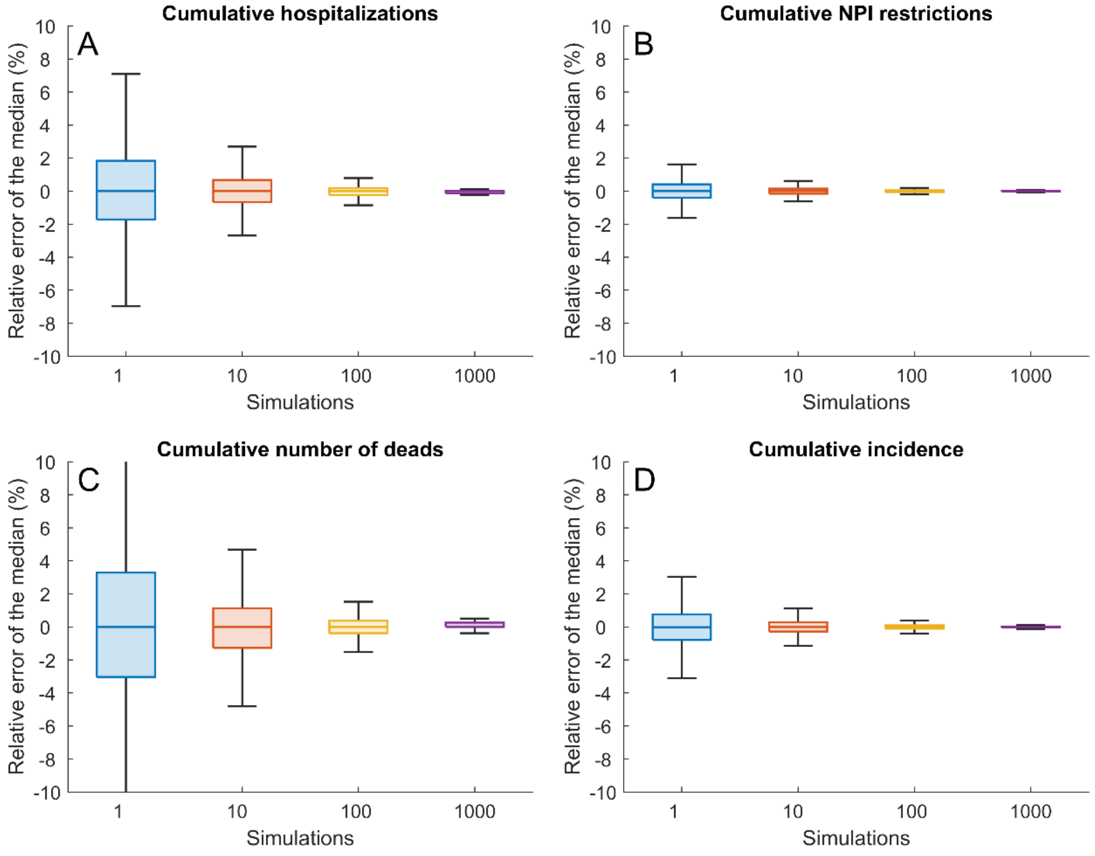

Figure 1. Relative error of the mean for different number of simulations using default parameters and without vaccination. The box represents 25 and 75% quantiles, the line in-between the median and black bars represents the 95% confidence intervals. (A) Cumulative

number of hospitalizations. (B) Cumulative Non-Pharmaceutical restrictions, which is equivalent to the area under de curve of restrictions evolution. (C) Cumulative number of deaths. (D) Cumulative incidence.

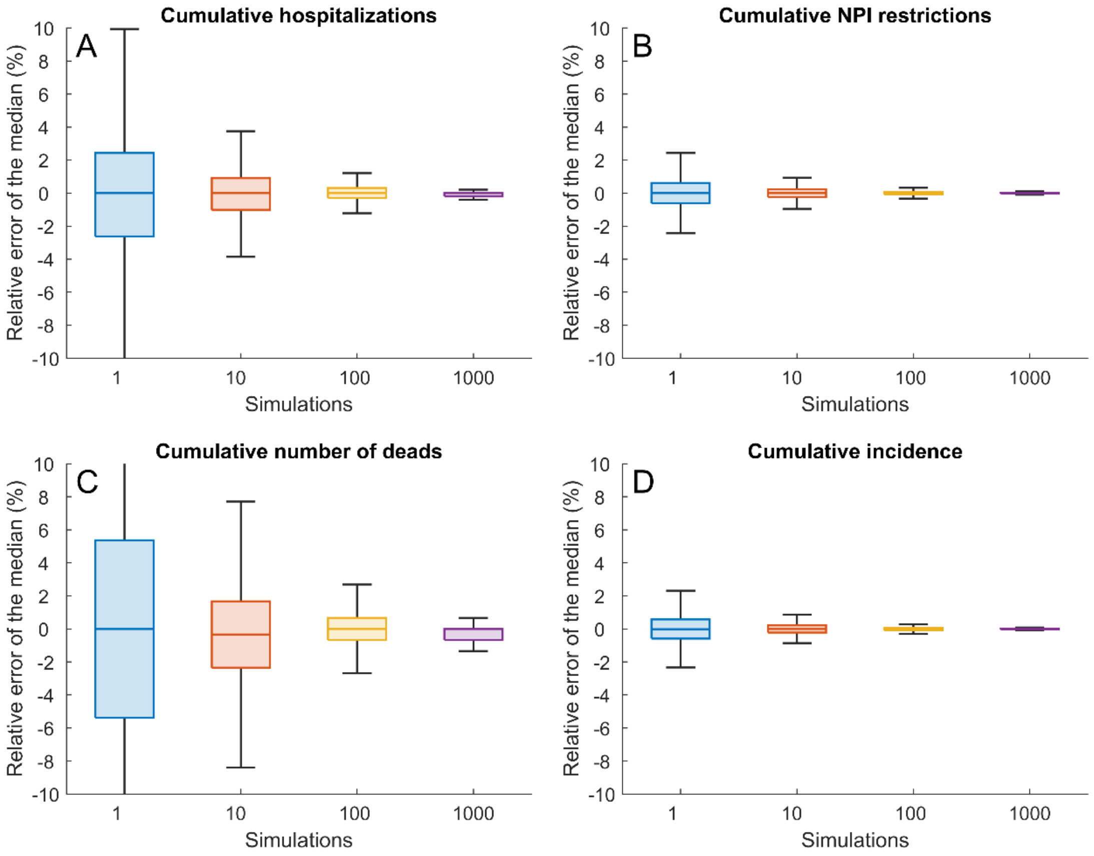

Figure 2. Relative error of the mean for different number of simulations using default parameters and CH+vul vaccination strategy. The box represents 25 and 75% quantiles, the line in-between the median and black bars represents the 95% confidence intervals. (A) Cumulative number of hospitalizations. (B) Cumulative Non-Pharmaceutical restrictions, which is equivalent to the area under de curve of restrictions evolution. (C) Cumulative number of deaths. (D) Cumulative incidence.

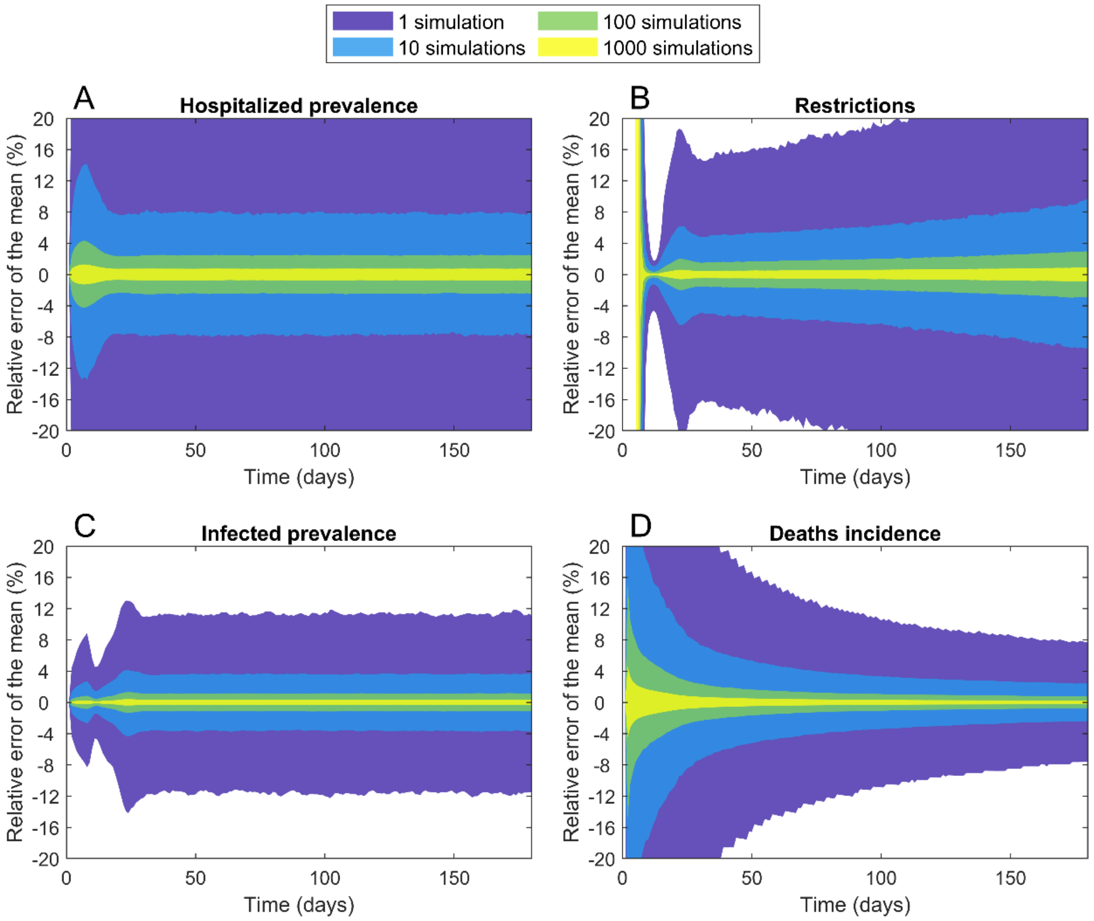

Figure 3. Evolution of relative error of the mean for different quantities using different number of simulations. Default parameters were used without vaccination strategy. Coloured parts represent the 95% confidence intervals. (A) Hospitalized individual prevalence. (B) Restrictions at each time point. (C) Infected individual prevalence. (D) Number of deaths evolution.

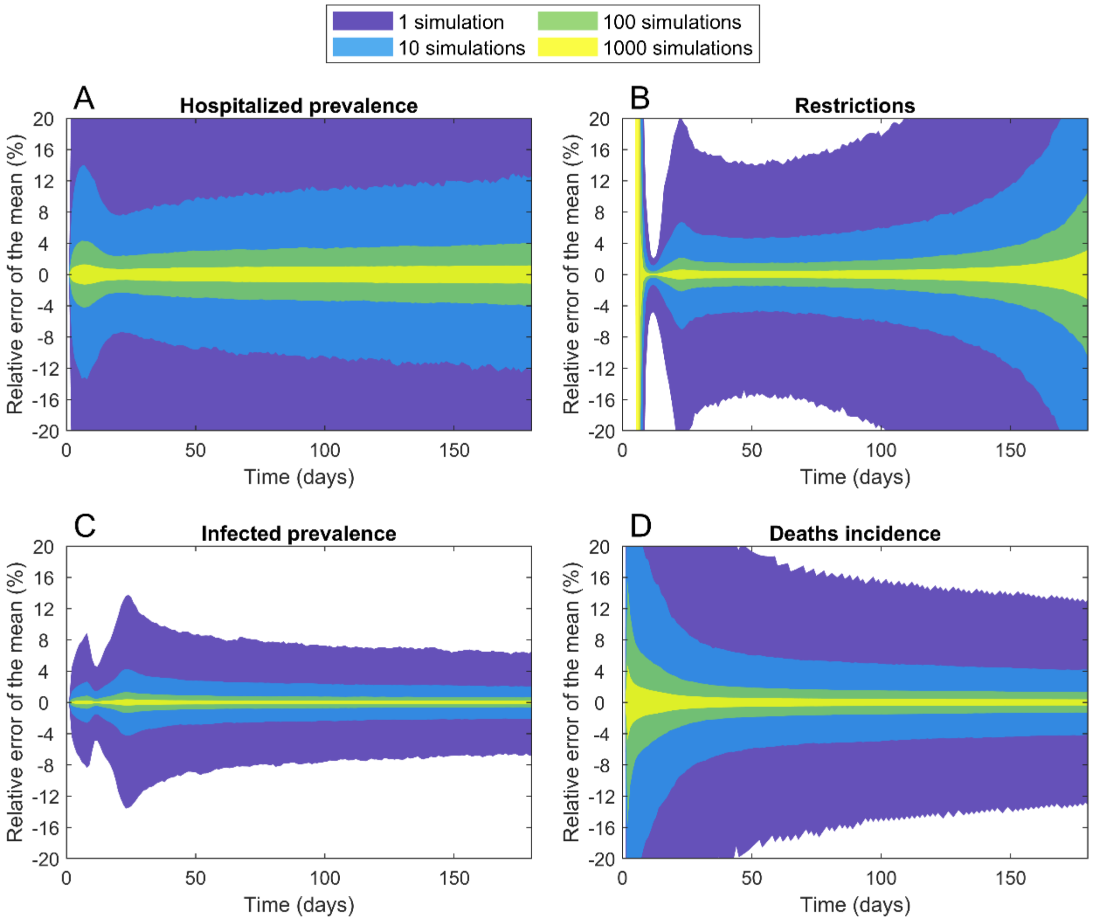

Figure 4. Evolution of relative error of the mean for different quantities using different number of simulations. Default parameters were used with CH+vul vaccination strategy. Coloured parts represent the 95% confidence intervals. (A) Hospitalized individual prevalence. (B) Restrictions at each time point. (C) Infected individual prevalence. (D) Number of deaths evolution.

References

1. Eurostat [Internet]. [cited 2021 Feb 18]. Available from: https://appsso.eurostat.ec.europa.eu/nui/submitViewTableAction.do

2. Official UK Coronavirus Dashboard [Internet]. [cited 2021 Feb 9]. Available from: https://coronavirus.data.gov.uk/

3. Report 34 - COVID-19 Infection Fatality Ratio estimates from seroprevalence [Internet]. [cited 2021 Feb 10]. Available from: https://www.imperial.ac.uk/mrc-global-infectious-disease-analysis/covid-19/report-34-ifr/

4. Riley S, Eales O, Walters CE, Wang H, Ainslie KEC, Atchison C, et al. REACT-1 round 8 final report: high average prevalence with regional heterogeneity of trends in SARS-CoV-2 infection in the community in England during January 2021 [Internet]. bioRxiv. medRxiv; 2021 [cited 2021 Feb 9]. Available from: https://spiral.imperial.ac.uk/handle/10044/1/85703

5. COVID-19 confirmed deaths in England (to 31 December 2020): report [Internet]. [cited 2021 Feb 23]. Available from: https://www.gov.uk/government/publications/covid-19-reported-sars-cov-2-deaths-in-england/covid-19-confirmed-deaths-in-england-to-31-december-2020-report

6. Coronavirus Pandemic Data Explorer [Internet]. [cited 2021 Feb 10]. Available from: https://ourworldindata.org/coronavirus-data-explorer?zoomToSelection=true&time=2020-03-01..latest&country=IND~USA~GBR~CAN~DEU~FRA&region=World&vaccinationsMetric=true&interval=smoothed&perCapita=true&smoothing=7&pickerMetric=total_cases&pickerSort=desc

7. Català M, Pino D, Marchena M, Palacios P, Urdiales T, Cardona P-J, et al. Robust estimation of diagnostic rate and real incidence of COVID-19 for European policymakers. PLoS One. 2021 Jan 7;16(1):e0243701.

8. Scheil-Adlung X, Others. Long-term care protection for older persons: A review of coverage deficits in 46 countries. ILO; 2015.

9. Nursing and elderly home beds per 100 000 [Internet]. [cited 2021 Feb 10]. Available from: https://gateway.euro.who.int/en/indicators/hfa_490-5100-nursing-and-elderly-home-beds-per-100-000/

10. OECD. Long-Term Care Resources and Utilisation : Long-term care recipients [Internet]. [cited 2021 Feb 10]. Available from: https://stats.oecd.org/index.aspx?queryid=30143

11. Li Q, Guan X, Wu P, Wang X, Zhou L, Tong Y, et al. Early transmission dynamics in Wuhan, China, of novel Coronavirus-infected pneumonia. N Engl J Med. 2020 Mar 26;382(13):1199–207.

12. To KK-W, Tsang OT-Y, Leung W-S, Tam AR, Wu T-C, Lung DC, et al. Temporal profiles of viral load in posterior oropharyngeal saliva samples and serum antibody responses during infection by SARS-CoV-2: an observational cohort study. Lancet Infect Dis. 2020 May 1;20(5):565–74.

13. Faes C, Abrams S, Van Beckhoven D, Meyfroidt G, Vlieghe E, Hens N, et al. Time between symptom onset, hospitalisation and recovery or death: Statistical analysis of Belgian COVID-19 patients. Int J Environ Res Public Health. 2020 Oct 17;17(20):7560.

14. Billah MA, Miah MM, Khan MN. Reproductive number of coronavirus: A systematic review and meta-analysis based on global level evidence. PLoS One. 2020 Nov 11;15(11):e0242128.

15. Davies NG, Klepac P, Liu Y, Prem K, Jit M, CMMID COVID-19 working group, et al. Age-dependent effects in the transmission and control of COVID-19 epidemics. Nat Med. 2020 Aug 1;26(8):1205–11.

16. Prieto-Alhambra D, Balló E, Coma E, Mora N, Aragón M, Prats-Uribe A, et al. Filling the gaps in the characterization of the clinical management of COVID-19: 30-day hospital admission and fatality rates in a cohort of 118 150 cases diagnosed in outpatient settings in Spain. Int J Epidemiol. 2021 Jan 23;49(6):1930–9.

17. Institut d'Estadística de Catalunya [Internet]. [cited 2021 Feb 23]. Available from: https://www.idescat.cat/

18. Polack FP, Thomas SJ, Kitchin N, Absalon J, Gurtman A, Lockhart S, et al. Safety and Efficacy of the BNT162b2 mRNA Covid-19 Vaccine. N Engl J Med. 2020 Dec 31;383(27):2603–15.

19. Voysey M, Clemens SAC, Madhi SA, Weckx LY, Folegatti PM, Aley PK, et al. Safety and efficacy of the ChAdOx1 nCoV-19 vaccine (AZD1222) against SARS-CoV-2: an interim analysis of four randomised controlled trials in Brazil, South Africa, and the UK. Lancet. 2021 Jan 9;397(10269):99–111.

20. Baden LR, El Sahly HM, Essink B, Kotloff K, Frey S, Novak R, et al. Efficacy and Safety of the mRNA-1273 SARS-CoV-2 Vaccine. N Engl J Med. 2021 Feb 4;384(5):403–16.
