# Supplementary material for: The impact of prioritisation and dosing intervals on the effects of COVID-19 vaccination in Europe: an agent-based cohort model
Source: Sci Rep. 2021 Sep 22;11:18812. doi: 10.1038/s41598-021-98216-0 (PMC8458447; doi:10.1038/s41598-021-98216-0)
Supplement: Supplementary file 2 — Supplementary Information 2. [file 41598_2021_98216_MOESM2_ESM.pdf]

The impact of prioritisation and dosing intervals on the effects of COVID-19 vaccination in Europe: an agent-based cohort model

Martí Català, Xintong Li, Clara Prats*, Daniel Prieto-Alhambra

*clara.prats@upc.edu

SUPPLEMENTARY MATERIAL: Different countries configuration

Table 1. Initial parameters configuration for different countries simulations. Other parameters have their default value. Population structures follow their own age and sex distribution.(1)

	**Population** (1)	**Total deaths** (2)	**Last week deaths** (2)	**Vaccination speed** (2)	**Increase in people living in care homes** (3–5)	**Proportion of nurses per resident** (3–5)
Austria	9006400	7237	743	0,14%	0,87	Def.
Belgium	11589616	20572	723	0,10%	2,16	Def.
Bulgaria	6948445	8651	730	0,01%	0,05	Def.
Croatia	4105267	4711	505	0,07%	0,25	Def.
Cyprus	1207361	176	42	0,09%	1,18	Def.
Czech Republic	10708982	14820	2389	0,10%	0,73	Def.
Denmark	5792203	1873	416	0,16%	0,98	0,24
Europe	747636045	538618	61799	0,14%	0,60	Def.
Estonia	1326538	349	86	0,08%	1,40	0,05
Finland	5540718	632	56	0,10%	1,26	Def.
France	65273510	71792	5260	0,09%	1,18	0,09
Germany	83783943	50010	12104	0,09%	1,15	0,15
Greece	10423056	5545	477	0,05%	0,02	Def.
Hungary	9660350	11615	1440	0,07%	0,84	0,06
Iceland	341250	29	1	0,02%	1,68	Def.
Ireland	4937796	2768	351	0,16%	0,92	0,22
Italy	60461828	83681	6874	0,10%	0,44	Def.
Latvia	1886201	1032	291	0,06%	0,11	Def.
Lithuania	2722289	2514	479	0,18%	2,97	Def.
Luxembourg	625975	562	49	0,07%	1,51	0,35
Malta	441539	244	16	0,27%	1,24	Def.
Netherlands	17134872	13350	1391	0,04%	1,12	0,26
Norway	5421241	543	72	0,08%	1,15	Def.
Poland	37846605	34141	4196	0,09%	0,22	Def.
Portugal	10196707	9465	1832	0,05%	0,36	0,03
Romania	19237681	17485	1214	0,10%	0,14	Def.
Slovakia	5459642	3737	1034	0,10%	0,88	Def.
Slovenia	2078931	3257	480	0,13%	1,03	Def.

Spain	46754782	54637	2441	0,15%	0,62	Def.
Sweden	10099270	10797	869	0,10%	1,20	Def.
Switzerland	8654617	8924	809	0,08%	1,57	0,29
United Kingdom	67886004	93469	14429	0,42%	0,92	Def.

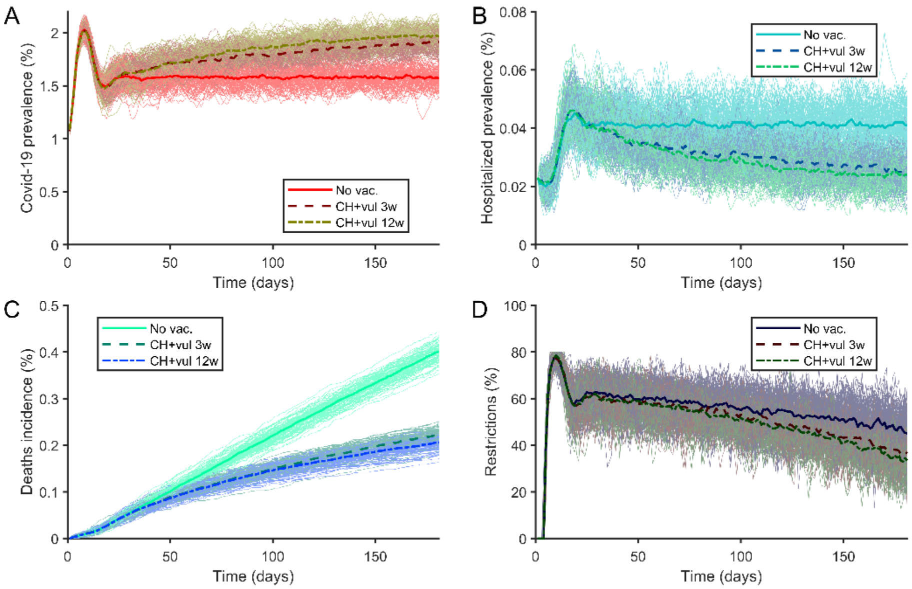

Figure 1. (A) Prevalence of covid-19 cases. (B) Prevalence of hospitalized covid-19 patients. (C) Covid-19 deaths. (D) Restrictions. Solid line for a non-vaccinated population. Dashed line for a population where they are vaccinating first care homes then vulnerable population. Dashed line for doses separation of 3 weeks. Dash-dot line for doses separation of 12 weeks. Figures for AstraZeneca vaccine using Austria conditions described in table 1.

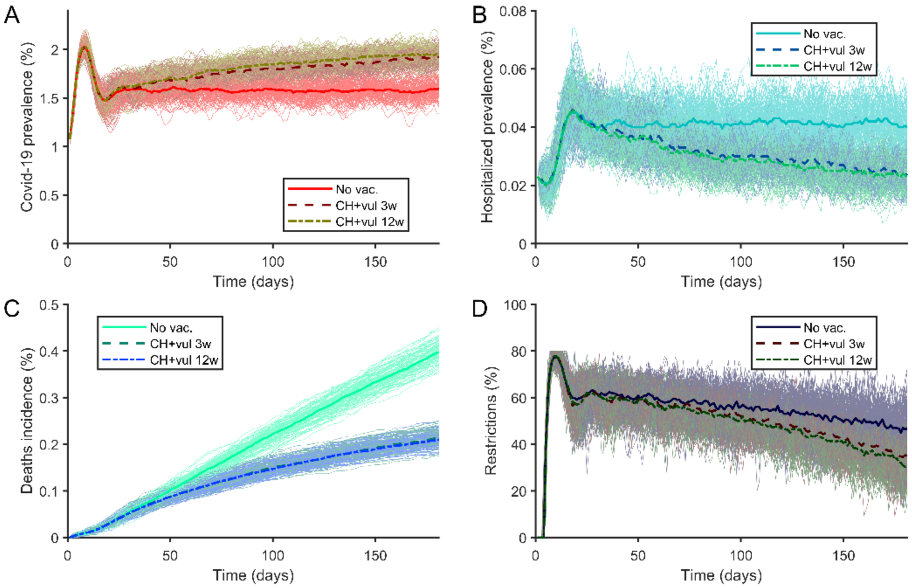

Figure 2. (A) Prevalence of covid-19 cases. (B) Prevalence of hospitalized covid-19 patients. (C) Covid-19 deaths. (D) Restrictions. Solid line for a non-vaccinated population. Dashed line for a population where

they are vaccinating first care homes then vulnerable population. Dashed line for doses separation of 3 weeks. Dash-dot line for doses separation of 12 weeks. Figures for Moderna vaccine using Austria conditions described in table 1.

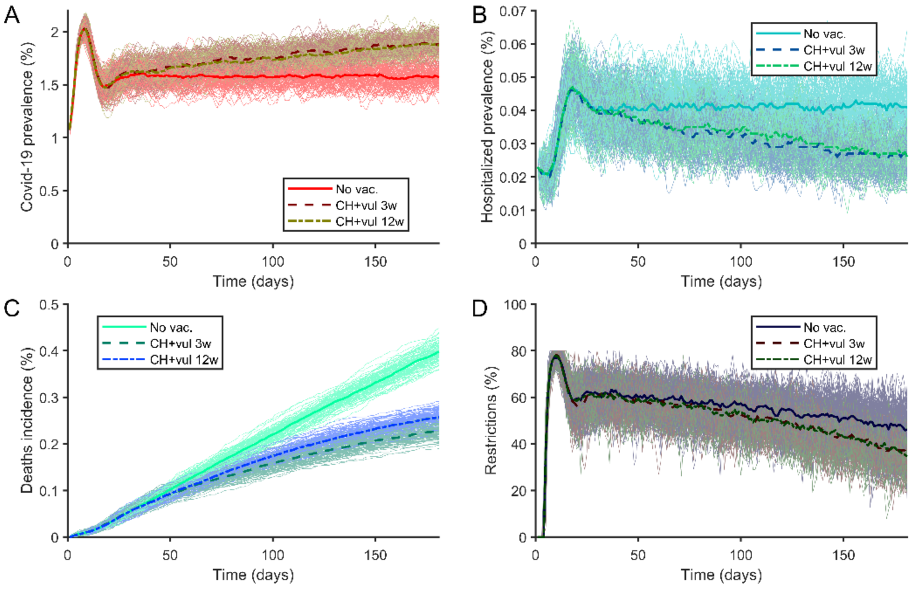

Figure 3. (A) Prevalence of covid-19 cases. (B) Prevalence of hospitalized covid-19 patients. (C) Covid-19 deaths. (D) Restrictions. Solid line for a non-vaccinated population. Dashed line for a population where they are vaccinating first care homes then vulnerable population. Dashed line for doses separation of 3 weeks. Dash-dot line for doses separation of 12 weeks. Figures for Pfizer vaccine using Austria conditions described in table 1.

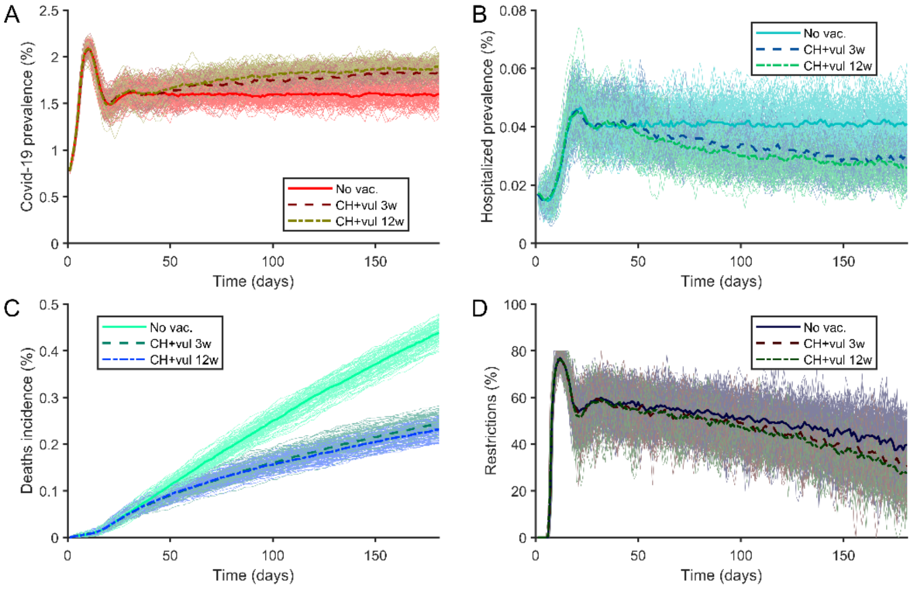

Figure 4. (A) Prevalence of covid-19 cases. (B) Prevalence of hospitalized covid-19 patients. (C) Covid-19 deaths. (D) Restrictions. Solid line for a non-vaccinated population. Dashed line for a population where they are vaccinating first care homes then vulnerable population. Dashed line for doses separation of 3

weeks. Dash-dot line for doses separation of 12 weeks. Figures for AstraZeneca vaccine using Belgium conditions described in table 1.

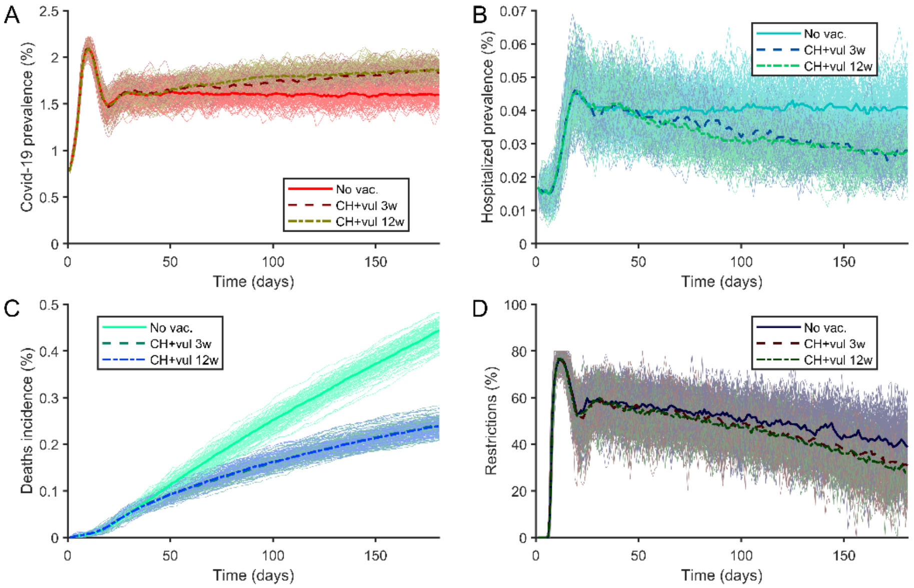

Figure 5. (A) Prevalence of covid-19 cases. (B) Prevalence of hospitalized covid-19 patients. (C) Covid-19 deaths. (D) Restrictions. Solid line for a non-vaccinated population. Dashed line for a population where they are vaccinating first care homes then vulnerable population. Dashed line for doses separation of 3 weeks. Dash-dot line for doses separation of 12 weeks. Figures for Moderna vaccine using Belgium conditions described in table 1.

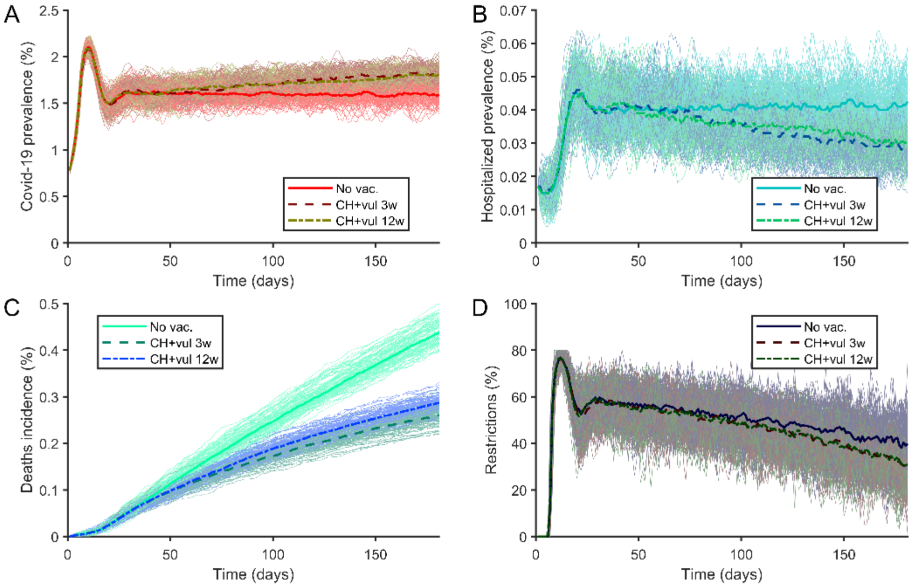

Figure 6. (A) Prevalence of covid-19 cases. (B) Prevalence of hospitalized covid-19 patients. (C) Covid-19 deaths. (D) Restrictions. Solid line for a non-vaccinated population. Dashed line for a population where they are vaccinating first care homes then vulnerable population. Dashed line for doses separation of 3 weeks. Dash-dot line for doses separation of 12 weeks. Figures for Pfizer vaccine using Belgium conditions described in table 1.

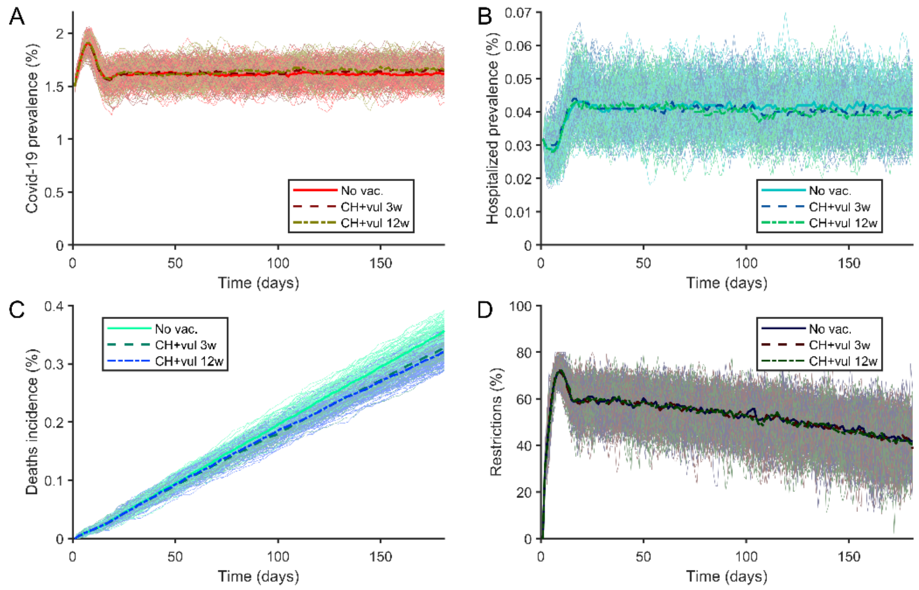

Figure 7. (A) Prevalence of covid-19 cases. (B) Prevalence of hospitalized covid-19 patients. (C) Covid-19 deaths. (D) Restrictions. Solid line for a non-vaccinated population. Dashed line for a population where they are vaccinating first care homes then vulnerable population. Dashed line for doses separation of 3 weeks. Dash-dot line for doses separation of 12 weeks. Figures for AstraZeneca vaccine using Bulgaria conditions described in table 1.

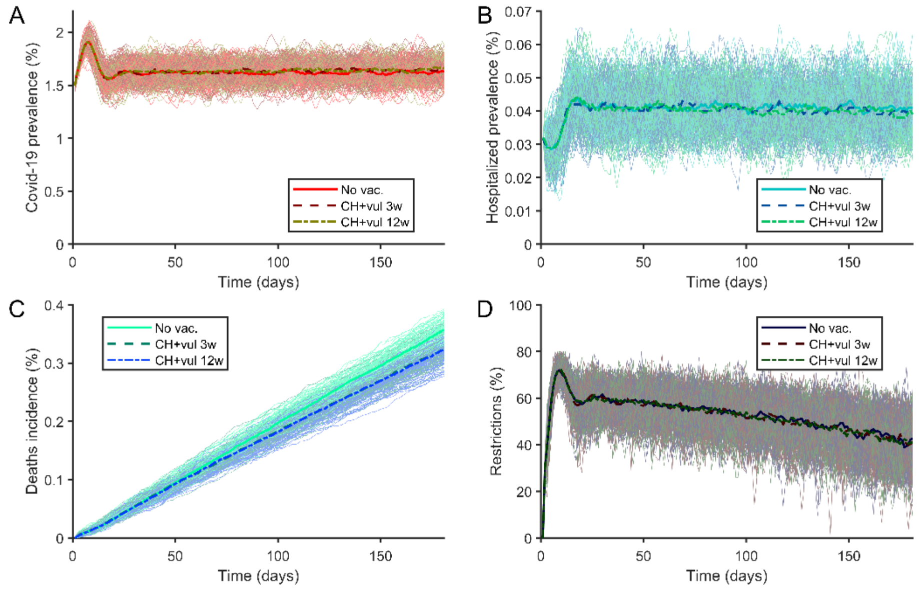

Figure 8. (A) Prevalence of covid-19 cases. (B) Prevalence of hospitalized covid-19 patients. (C) Covid-19 deaths. (D) Restrictions. Solid line for a non-vaccinated population. Dashed line for a population where they are vaccinating first care homes then vulnerable population. Dashed line for doses separation of 3 weeks. Dash-dot line for doses separation of 12 weeks. Figures for Moderna vaccine using Bulgaria conditions described in table 1.

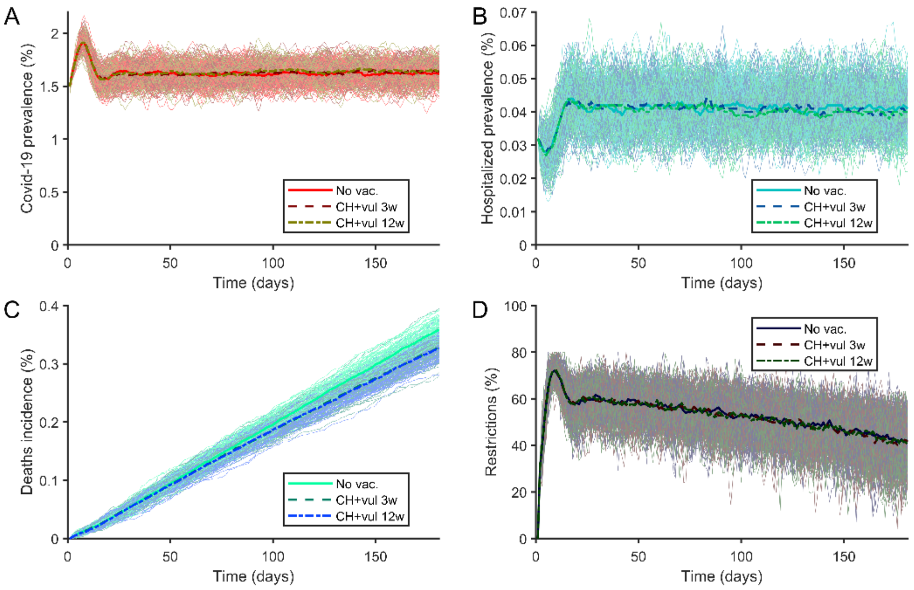

Figure 9. (A) Prevalence of covid-19 cases. (B) Prevalence of hospitalized covid-19 patients. (C) Covid-19 deaths. (D) Restrictions. Solid line for a non-vaccinated population. Dashed line for a population where they are vaccinating first care homes then vulnerable population. Dashed line for doses separation of 3 weeks. Dash-dot line for doses separation of 12 weeks. Figures for Pfizer vaccine using Bulgaria conditions described in table 1.

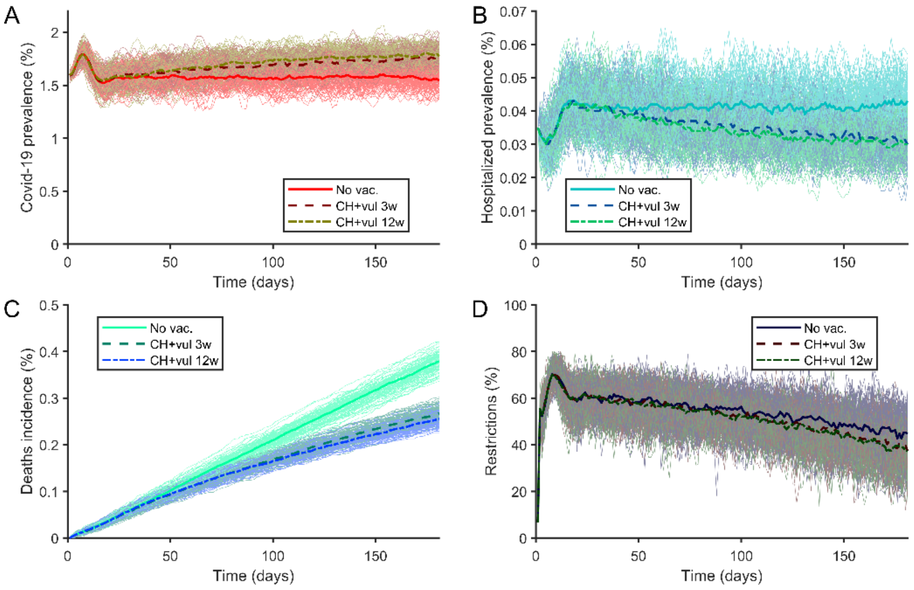

Figure 10. (A) Prevalence of covid-19 cases. (B) Prevalence of hospitalized covid-19 patients. (C) Covid-19 deaths. (D) Restrictions. Solid line for a non-vaccinated population. Dashed line for a population where they are vaccinating first care homes then vulnerable population. Dashed line for doses separation of 3 weeks. Dash-dot line for doses separation of 12 weeks. Figures for AstraZeneca vaccine using Croatia conditions described in table 1.

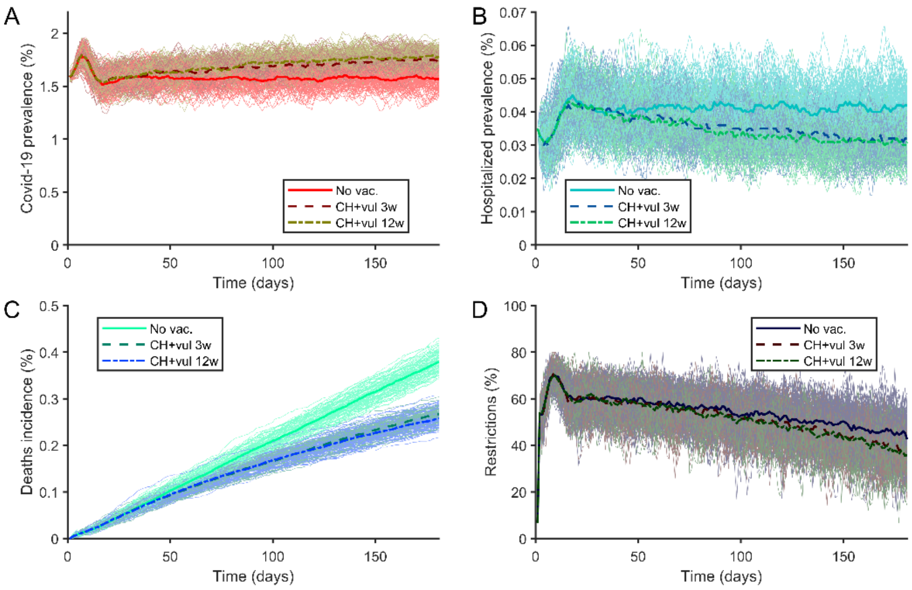

Figure 11. (A) Prevalence of covid-19 cases. (B) Prevalence of hospitalized covid-19 patients. (C) Covid-19 deaths. (D) Restrictions. Solid line for a non-vaccinated population. Dashed line for a population where they are vaccinating first care homes then vulnerable population. Dashed line for doses separation of 3 weeks. Dash-dot line for doses separation of 12 weeks. Figures for Moderna vaccine using Croatia conditions described in table 1.

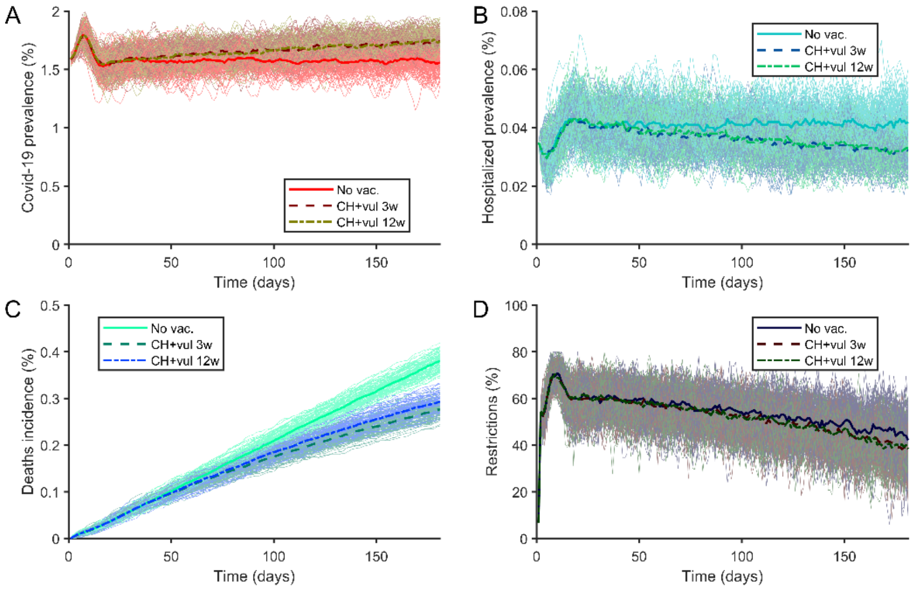

Figure 12. (A) Prevalence of covid-19 cases. (B) Prevalence of hospitalized covid-19 patients. (C) Covid-19 deaths. (D) Restrictions. Solid line for a non-vaccinated population. Dashed line for a population where they are vaccinating first care homes then vulnerable population. Dashed line for doses separation of 3 weeks. Dash-dot line for doses separation of 12 weeks. Figures for Pfizer vaccine using Croatia conditions described in table 1.

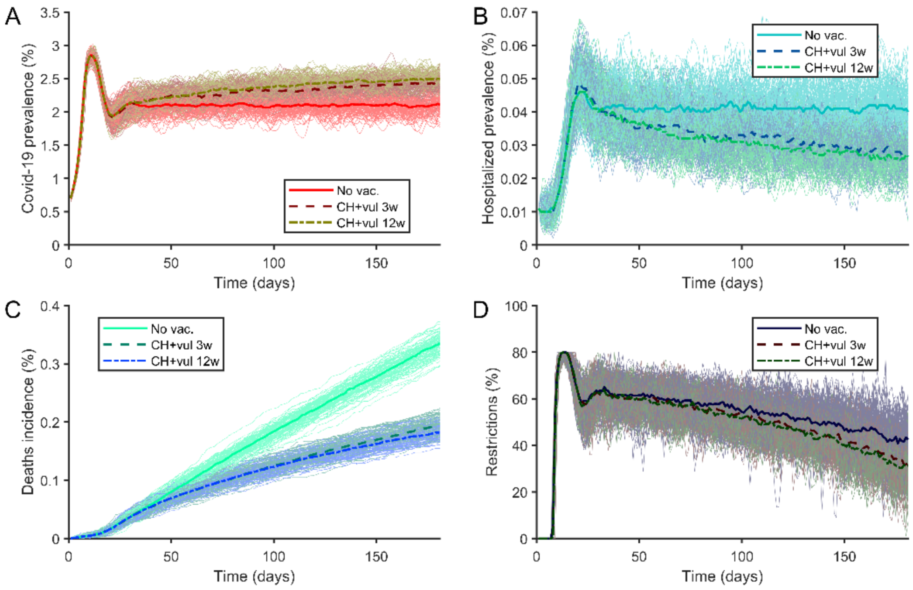

Figure 13. (A) Prevalence of covid-19 cases. (B) Prevalence of hospitalized covid-19 patients. (C) Covid-19 deaths. (D) Restrictions. Solid line for a non-vaccinated population. Dashed line for a population where they are vaccinating first care homes then vulnerable population. Dashed line for doses separation of 3 weeks. Dash-dot line for doses separation of 12 weeks. Figures for AstraZeneca vaccine using Cyprus conditions described in table 1.

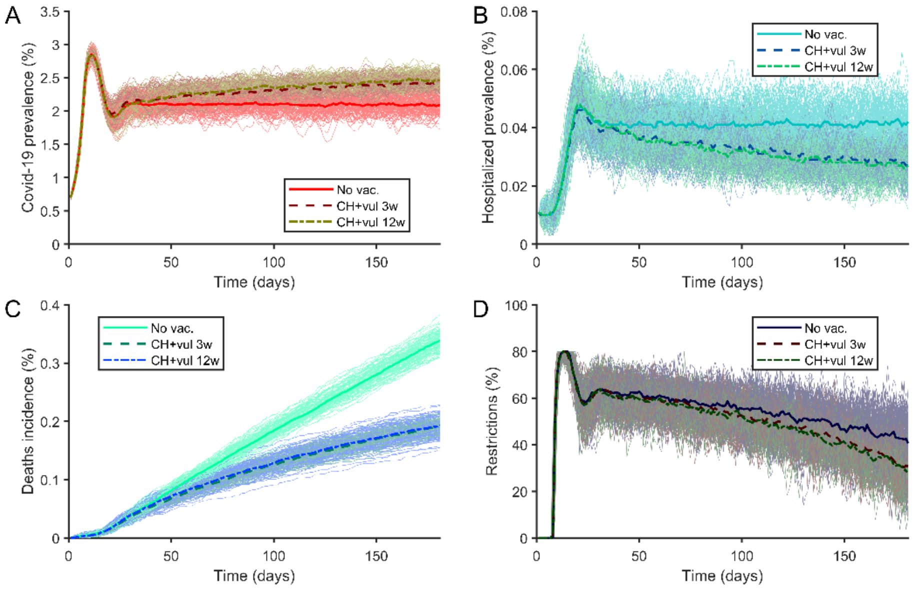

Figure 14. (A) Prevalence of covid-19 cases. (B) Prevalence of hospitalized covid-19 patients. (C) Covid-19 deaths. (D) Restrictions. Solid line for a non-vaccinated population. Dashed line for a population where they are vaccinating first care homes then vulnerable population. Dashed line for doses separation of 3 weeks. Dash-dot line for doses separation of 12 weeks. Figures for Moderna vaccine using Cyprus conditions described in table 1.

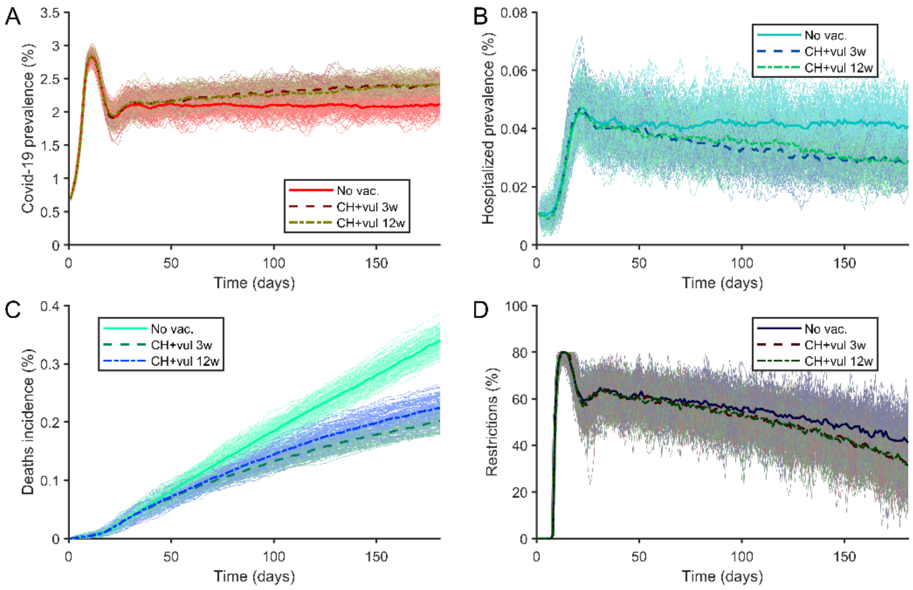

Figure 15. (A) Prevalence of covid-19 cases. (B) Prevalence of hospitalized covid-19 patients. (C) Covid-19 deaths. (D) Restrictions. Solid line for a non-vaccinated population. Dashed line for a population where they are vaccinating first care homes then vulnerable population. Dashed line for doses separation of 3 weeks. Dash-dot line for doses separation of 12 weeks. Figures for Pfizer vaccine using Cyprus conditions described in table 1.

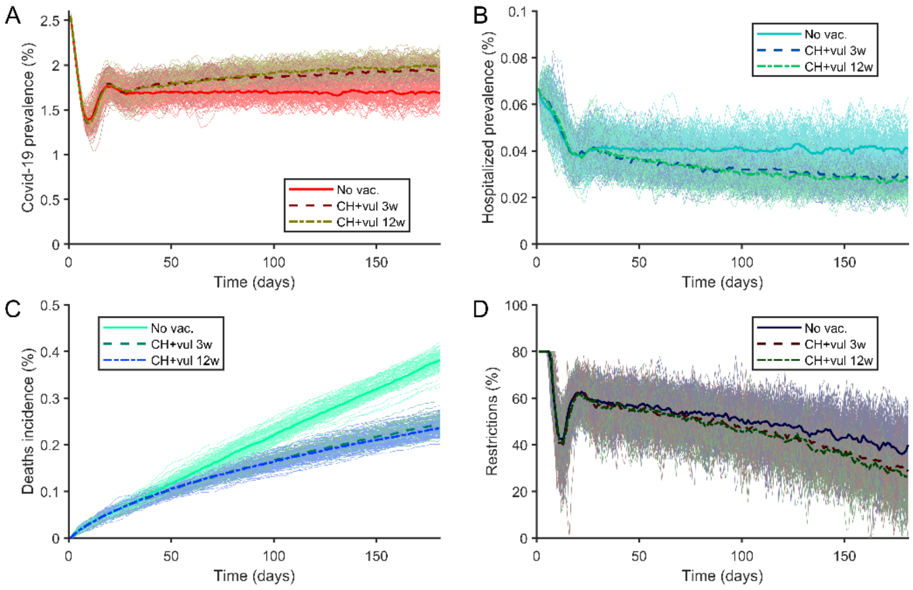

Figure 16. (A) Prevalence of covid-19 cases. (B) Prevalence of hospitalized covid-19 patients. (C) Covid-19 deaths. (D) Restrictions. Solid line for a non-vaccinated population. Dashed line for a population where they are vaccinating first care homes then vulnerable population. Dashed line for doses separation of 3 weeks. Dash-dot line for doses separation of 12 weeks. Figures for AstraZeneca vaccine using Czech Republic conditions described in table 1.

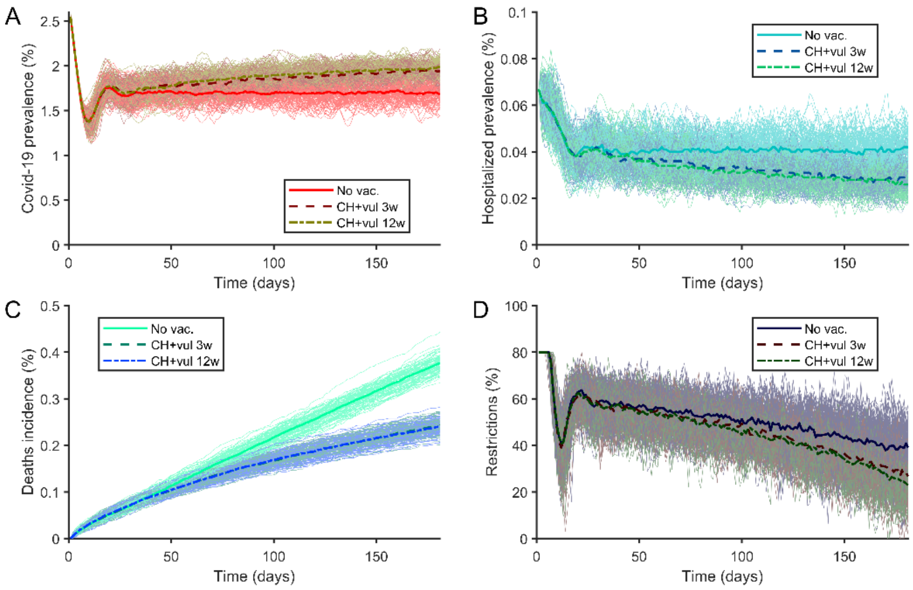

Figure 17. (A) Prevalence of covid-19 cases. (B) Prevalence of hospitalized covid-19 patients. (C) Covid-19 deaths. (D) Restrictions. Solid line for a non-vaccinated population. Dashed line for a population where they are vaccinating first care homes then vulnerable population. Dashed line for doses separation of 3 weeks. Dash-dot line for doses separation of 12 weeks. Figures for Moderna vaccine using Czech Republic conditions described in table 1.

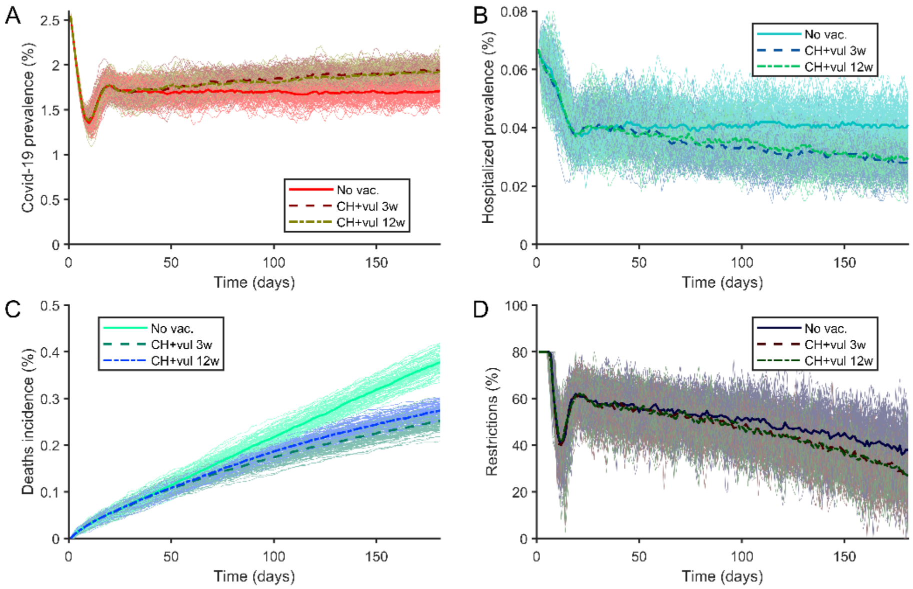

Figure 18. (A) Prevalence of covid-19 cases. (B) Prevalence of hospitalized covid-19 patients. (C) Covid-19 deaths. (D) Restrictions. Solid line for a non-vaccinated population. Dashed line for a population where they are vaccinating first care homes then vulnerable population. Dashed line for doses separation of 3 weeks. Dash-dot line for doses separation of 12 weeks. Figures for Pfizer vaccine using Czech Republic conditions described in table 1.

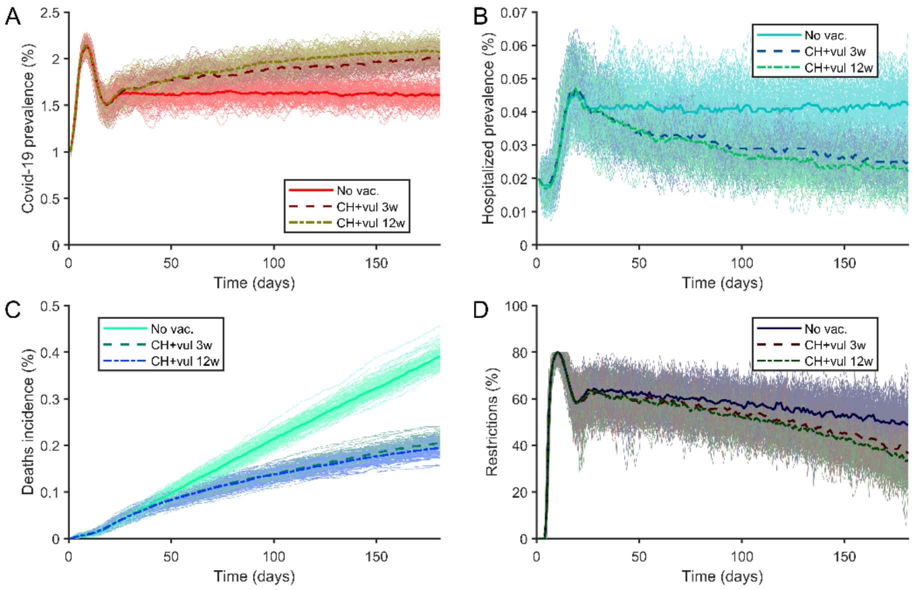

Figure 19. (A) Prevalence of covid-19 cases. (B) Prevalence of hospitalized covid-19 patients. (C) Covid-19 deaths. (D) Restrictions. Solid line for a non-vaccinated population. Dashed line for a population where they are vaccinating first care homes then vulnerable population. Dashed line for doses separation of 3 weeks. Dash-dot line for doses separation of 12 weeks. Figures for AstraZeneca vaccine using Denmark conditions described in table 1.

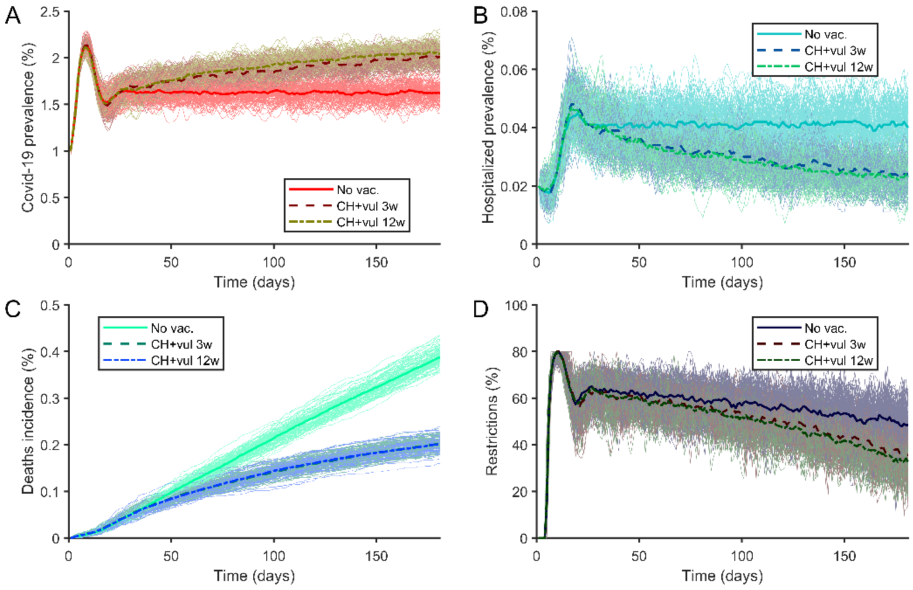

Figure 20. (A) Prevalence of covid-19 cases. (B) Prevalence of hospitalized covid-19 patients. (C) Covid-19 deaths. (D) Restrictions. Solid line for a non-vaccinated population. Dashed line for a population where they are vaccinating first care homes then vulnerable population. Dashed line for doses separation of 3 weeks. Dash-dot line for doses separation of 12 weeks. Figures for Moderna vaccine using Denmark conditions described in table 1.

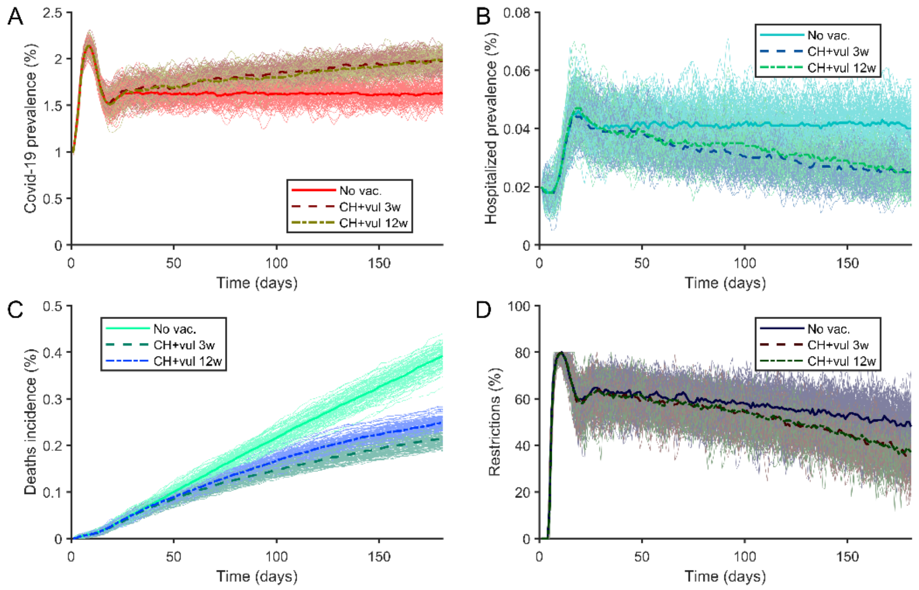

Figure 21. (A) Prevalence of covid-19 cases. (B) Prevalence of hospitalized covid-19 patients. (C) Covid-19 deaths. (D) Restrictions. Solid line for a non-vaccinated population. Dashed line for a population where they are vaccinating first care homes then vulnerable population. Dashed line for doses separation of 3 weeks. Dash-dot line for doses separation of 12 weeks. Figures for Pfizer vaccine using Denmark conditions described in table 1.

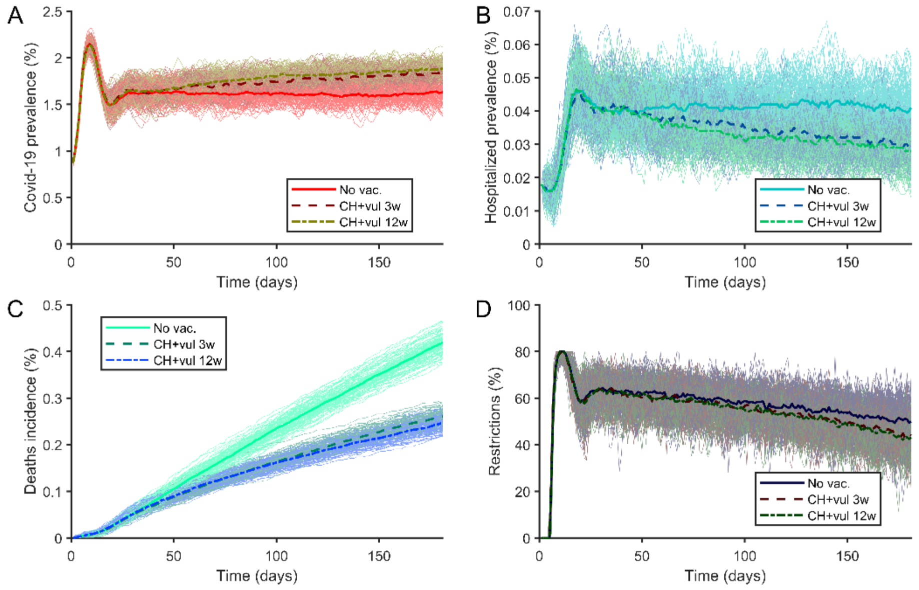

Figure 22. (A) Prevalence of covid-19 cases. (B) Prevalence of hospitalized covid-19 patients. (C) Covid-19 deaths. (D) Restrictions. Solid line for a non-vaccinated population. Dashed line for a population where they are vaccinating first care homes then vulnerable population. Dashed line for doses separation of 3 weeks. Dash-dot line for doses separation of 12 weeks. Figures for AstraZeneca vaccine using Estonia conditions described in table 1.

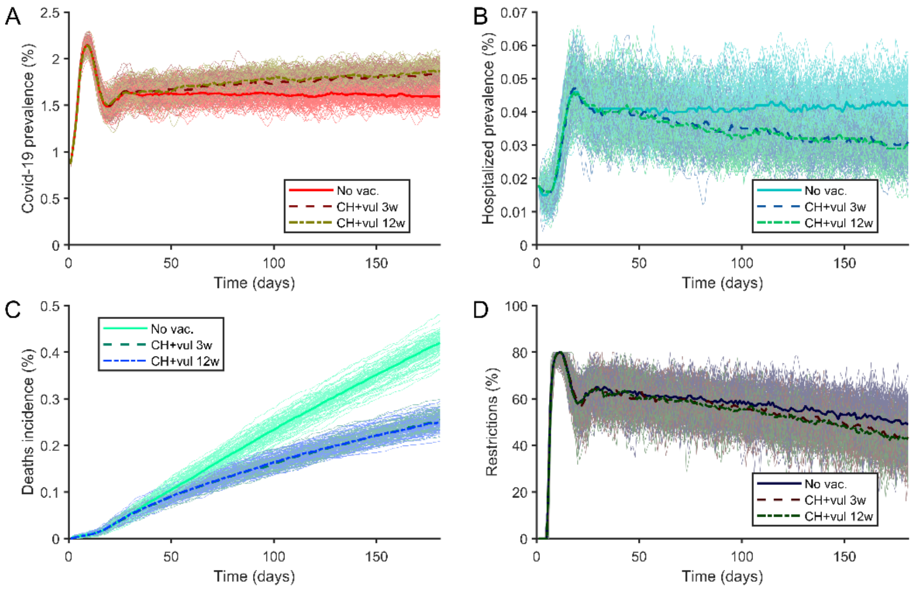

Figure 23. (A) Prevalence of covid-19 cases. (B) Prevalence of hospitalized covid-19 patients. (C) Covid-19 deaths. (D) Restrictions. Solid line for a non-vaccinated population. Dashed line for a population where they are vaccinating first care homes then vulnerable population. Dashed line for doses separation of 3 weeks. Dash-dot line for doses separation of 12 weeks. Figures for Moderna vaccine using Estonia conditions described in table 1.

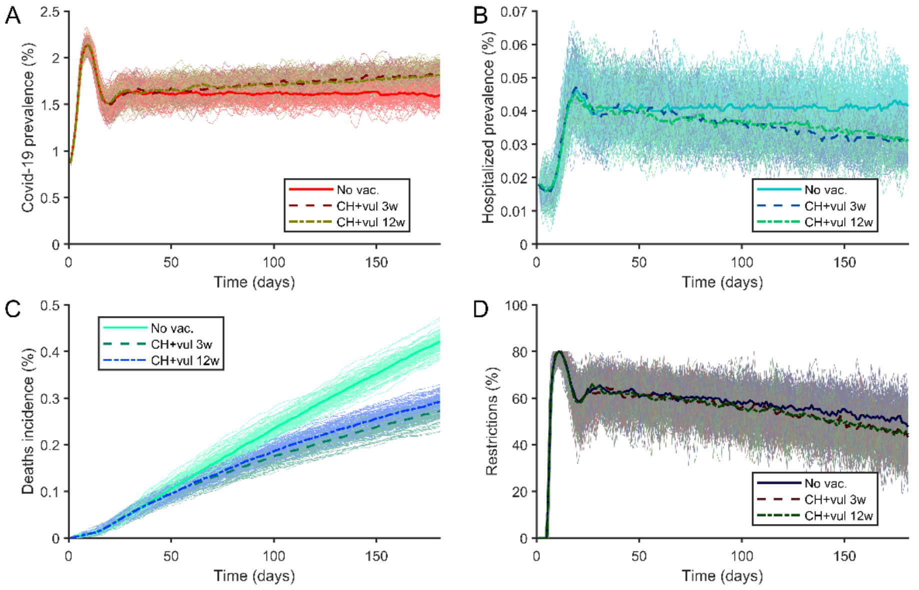

Figure 24. (A) Prevalence of covid-19 cases. (B) Prevalence of hospitalized covid-19 patients. (C) Covid-19 deaths. (D) Restrictions. Solid line for a non-vaccinated population. Dashed line for a population where they are vaccinating first care homes then vulnerable population. Dashed line for doses separation of 3 weeks. Dash-dot line for doses separation of 12 weeks. Figures for Pfizer vaccine using Estonia conditions described in table 1.

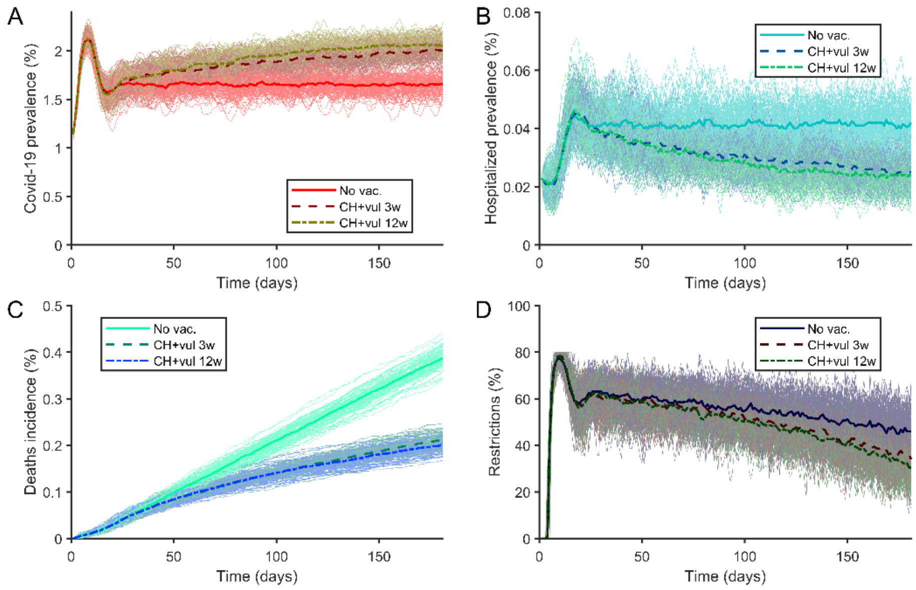

Figure 25. (A) Prevalence of covid-19 cases. (B) Prevalence of hospitalized covid-19 patients. (C) Covid-19 deaths. (D) Restrictions. Solid line for a non-vaccinated population. Dashed line for a population where they are vaccinating first care homes then vulnerable population. Dashed line for doses separation of 3 weeks. Dash-dot line for doses separation of 12 weeks. Figures for AstraZeneca vaccine using Europe conditions described in table 1.

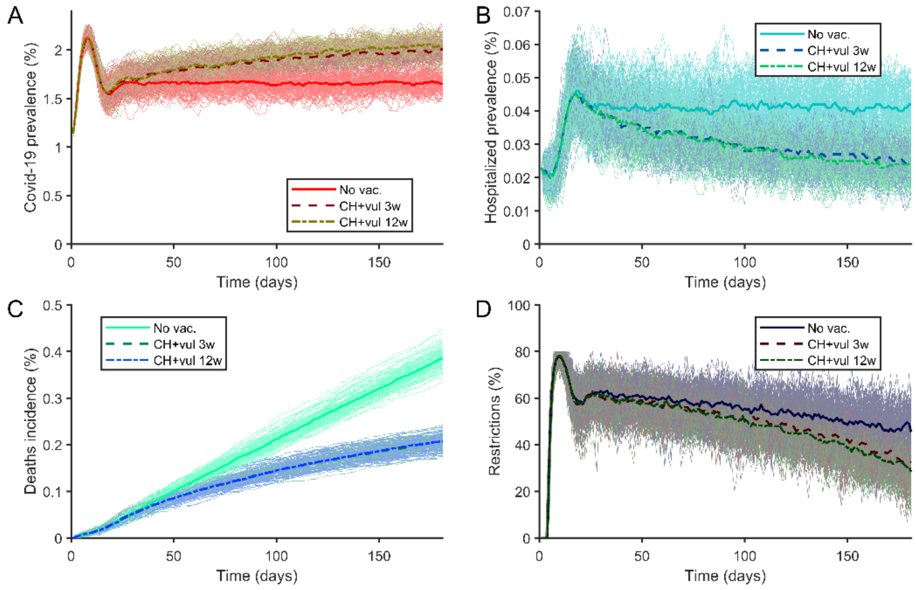

Figure 26. (A) Prevalence of covid-19 cases. (B) Prevalence of hospitalized covid-19 patients. (C) Covid-19 deaths. (D) Restrictions. Solid line for a non-vaccinated population. Dashed line for a population where they are vaccinating first care homes then vulnerable population. Dashed line for doses separation of 3 weeks. Dash-dot line for doses separation of 12 weeks. Figures for Moderna vaccine using Europe conditions described in table 1.

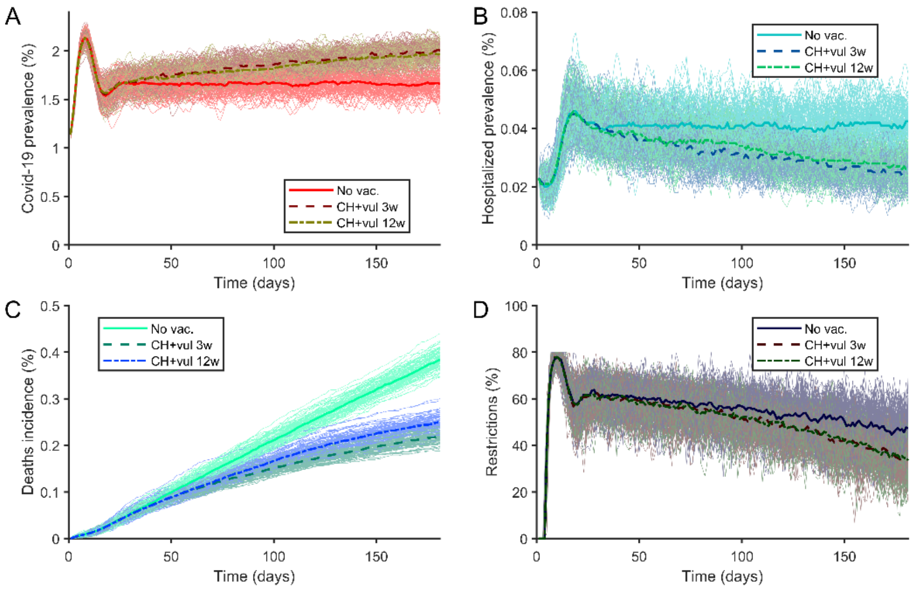

Figure 27. (A) Prevalence of covid-19 cases. (B) Prevalence of hospitalized covid-19 patients. (C) Covid-19 deaths. (D) Restrictions. Solid line for a non-vaccinated population. Dashed line for a population where they are vaccinating first care homes then vulnerable population. Dashed line for doses separation of 3 weeks. Dash-dot line for doses separation of 12 weeks. Figures for Pfizer vaccine using Europe conditions described in table 1.

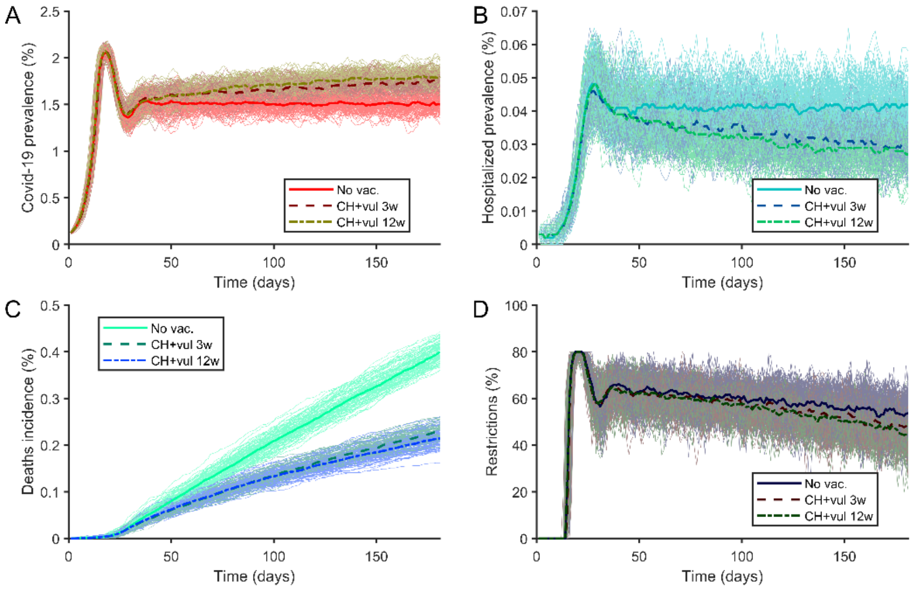

Figure 28. (A) Prevalence of covid-19 cases. (B) Prevalence of hospitalized covid-19 patients. (C) Covid-19 deaths. (D) Restrictions. Solid line for a non-vaccinated population. Dashed line for a population where they are vaccinating first care homes then vulnerable population. Dashed line for doses separation of 3 weeks. Dash-dot line for doses separation of 12 weeks. Figures for AstraZeneca vaccine using Finland conditions described in table 1.

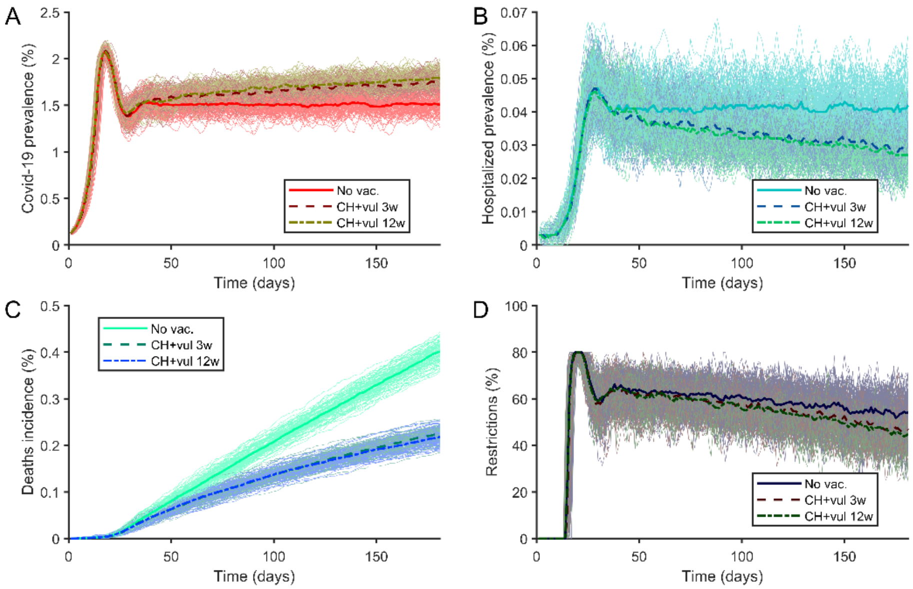

Figure 29. (A) Prevalence of covid-19 cases. (B) Prevalence of hospitalized covid-19 patients. (C) Covid-19 deaths. (D) Restrictions. Solid line for a non-vaccinated population. Dashed line for a population where they are vaccinating first care homes then vulnerable population. Dashed line for doses separation of 3 weeks. Dash-dot line for doses separation of 12 weeks. Figures for Moderna vaccine using Finland conditions described in table 1.

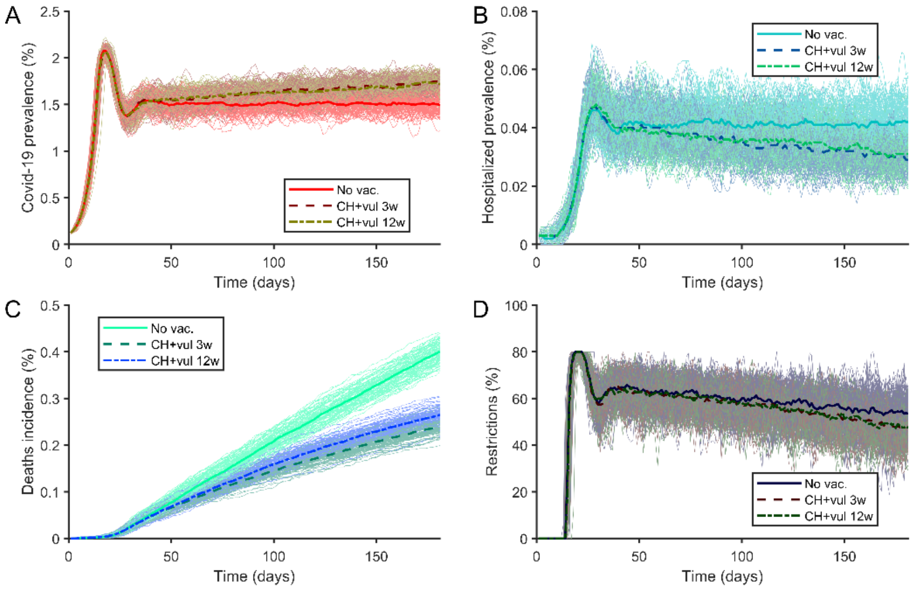

Figure 30. (A) Prevalence of covid-19 cases. (B) Prevalence of hospitalized covid-19 patients. (C) Covid-19 deaths. (D) Restrictions. Solid line for a non-vaccinated population. Dashed line for a population where they are vaccinating first care homes then vulnerable population. Dashed line for doses separation of 3 weeks. Dash-dot line for doses separation of 12 weeks. Figures for Pfizer vaccine using Finland conditions described in table 1.

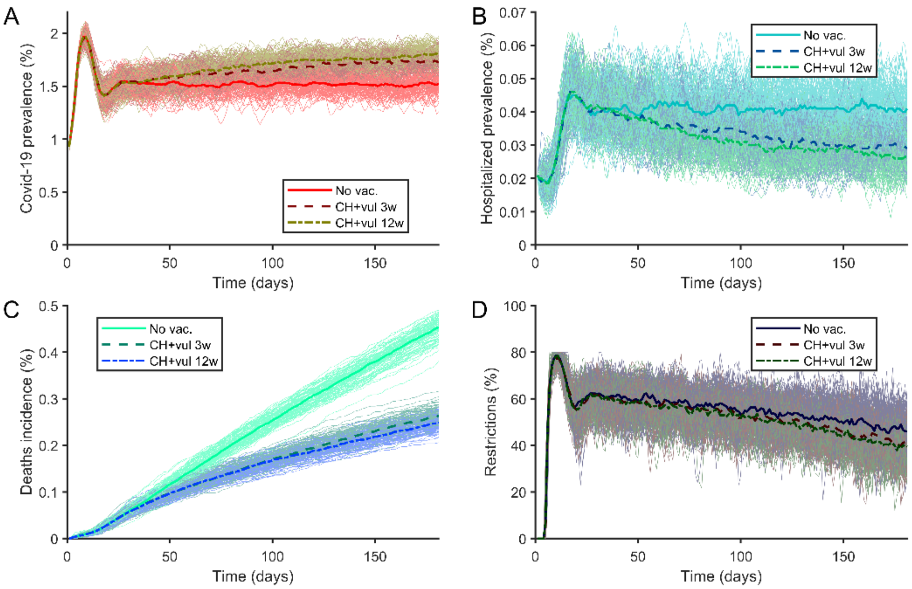

Figure 31. (A) Prevalence of covid-19 cases. (B) Prevalence of hospitalized covid-19 patients. (C) Covid-19 deaths. (D) Restrictions. Solid line for a non-vaccinated population. Dashed line for a population where they are vaccinating first care homes then vulnerable population. Dashed line for doses separation of 3 weeks. Dash-dot line for doses separation of 12 weeks. Figures for AstraZeneca vaccine using France conditions described in table 1.

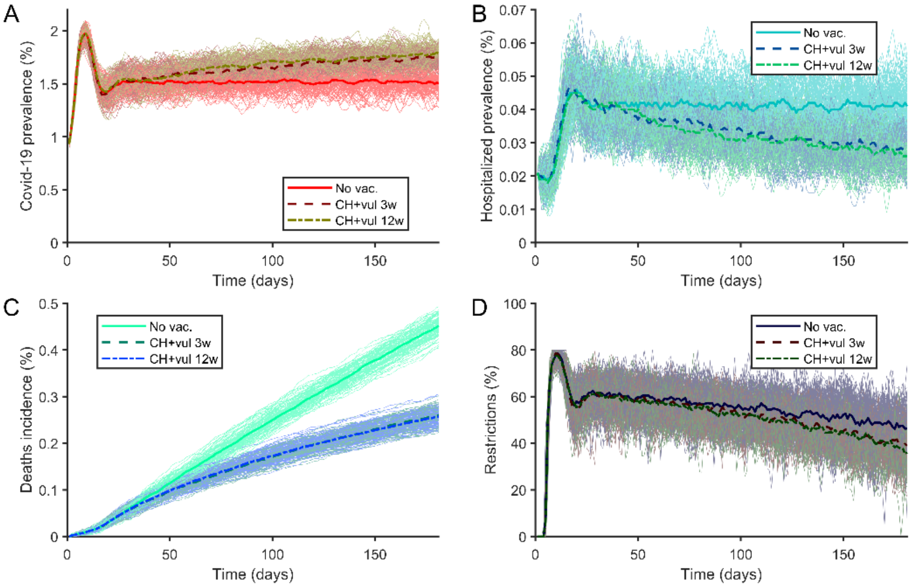

Figure 32. (A) Prevalence of covid-19 cases. (B) Prevalence of hospitalized covid-19 patients. (C) Covid-19 deaths. (D) Restrictions. Solid line for a non-vaccinated population. Dashed line for a population where they are vaccinating first care homes then vulnerable population. Dashed line for doses separation of 3 weeks. Dash-dot line for doses separation of 12 weeks. Figures for Moderna vaccine using France conditions described in table 1.

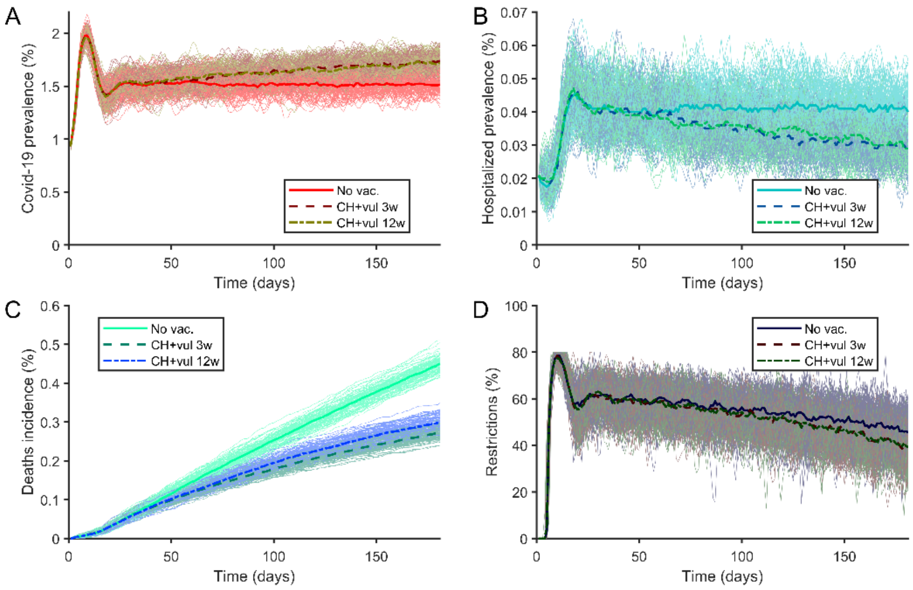

Figure 33. (A) Prevalence of covid-19 cases. (B) Prevalence of hospitalized covid-19 patients. (C) Covid-19 deaths. (D) Restrictions. Solid line for a non-vaccinated population. Dashed line for a population where they are vaccinating first care homes then vulnerable population. Dashed line for doses separation of 3 weeks. Dash-dot line for doses separation of 12 weeks. Figures for Pfizer vaccine using France conditions described in table 1.

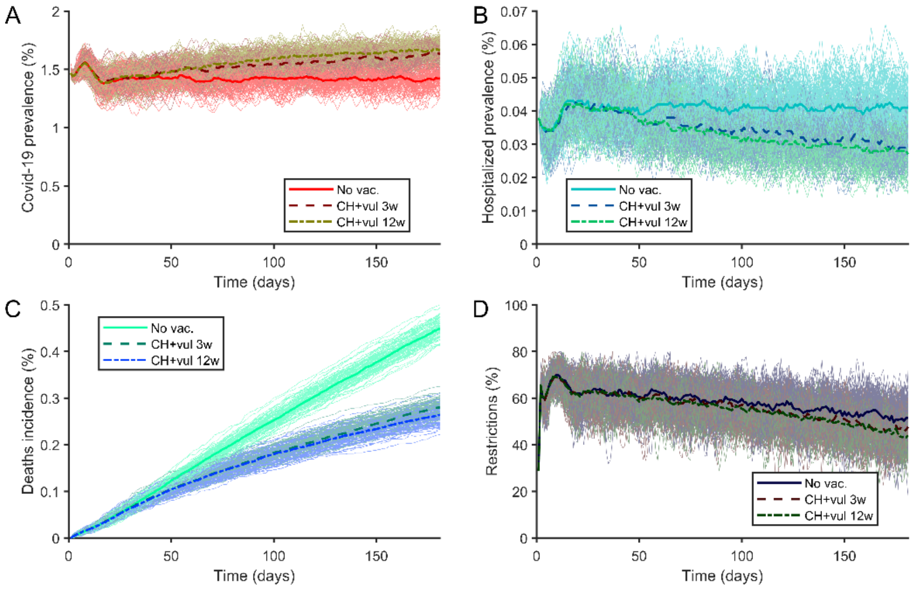

Figure 34. (A) Prevalence of covid-19 cases. (B) Prevalence of hospitalized covid-19 patients. (C) Covid-19 deaths. (D) Restrictions. Solid line for a non-vaccinated population. Dashed line for a population where they are vaccinating first care homes then vulnerable population. Dashed line for doses separation of 3 weeks. Dash-dot line for doses separation of 12 weeks. Figures for AstraZeneca vaccine using Germany conditions described in table 1.

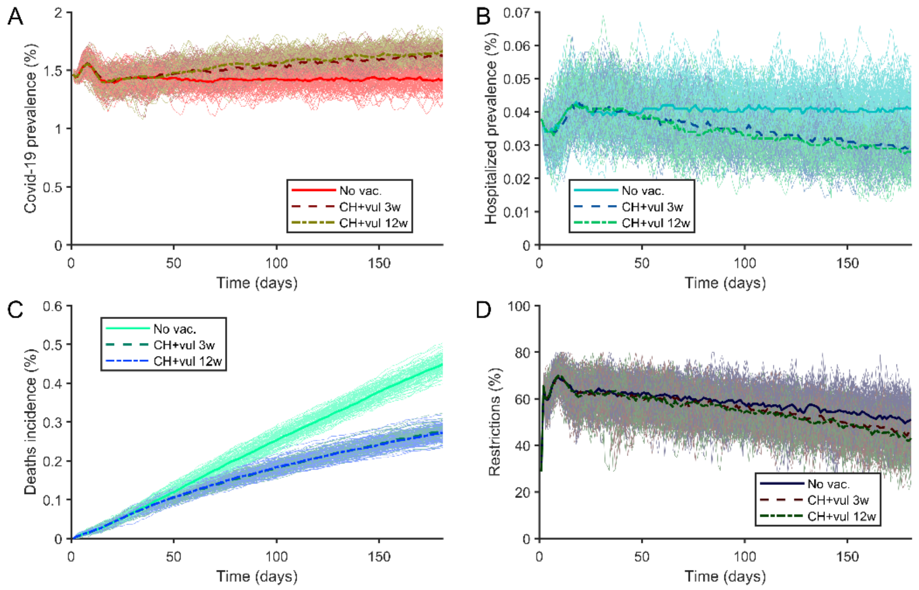

Figure 35. (A) Prevalence of covid-19 cases. (B) Prevalence of hospitalized covid-19 patients. (C) Covid-19 deaths. (D) Restrictions. Solid line for a non-vaccinated population. Dashed line for a population where they are vaccinating first care homes then vulnerable population. Dashed line for doses separation of 3 weeks. Dash-dot line for doses separation of 12 weeks. Figures for Moderna vaccine using Germany conditions described in table 1.

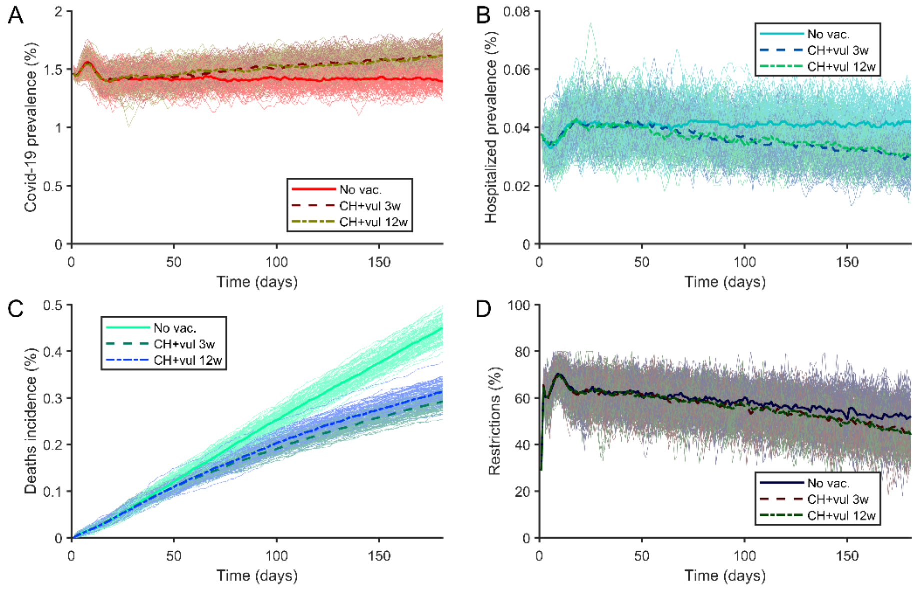

Figure 36. (A) Prevalence of covid-19 cases. (B) Prevalence of hospitalized covid-19 patients. (C) Covid-19 deaths. (D) Restrictions. Solid line for a non-vaccinated population. Dashed line for a population where they are vaccinating first care homes then vulnerable population. Dashed line for doses separation of 3 weeks. Dash-dot line for doses separation of 12 weeks. Figures for Pfizer vaccine using Germany conditions described in table 1.

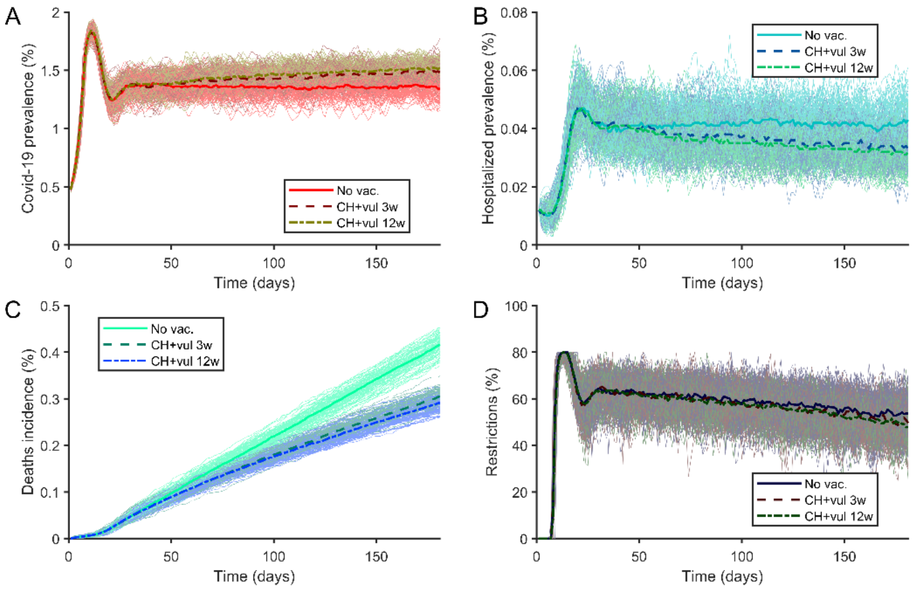

Figure 37. (A) Prevalence of covid-19 cases. (B) Prevalence of hospitalized covid-19 patients. (C) Covid-19 deaths. (D) Restrictions. Solid line for a non-vaccinated population. Dashed line for a population where they are vaccinating first care homes then vulnerable population. Dashed line for doses separation of 3 weeks. Dash-dot line for doses separation of 12 weeks. Figures for AstraZeneca vaccine using Greece conditions described in table 1.

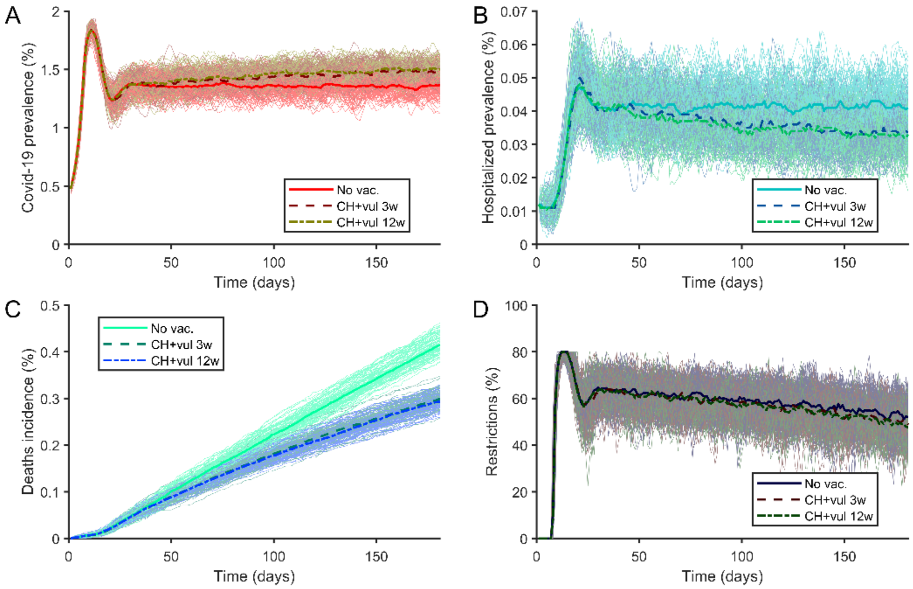

Figure 38. (A) Prevalence of covid-19 cases. (B) Prevalence of hospitalized covid-19 patients. (C) Covid-19 deaths. (D) Restrictions. Solid line for a non-vaccinated population. Dashed line for a population where they are vaccinating first care homes then vulnerable population. Dashed line for doses separation of 3 weeks. Dash-dot line for doses separation of 12 weeks. Figures for Moderna vaccine using Greece conditions described in table 1.

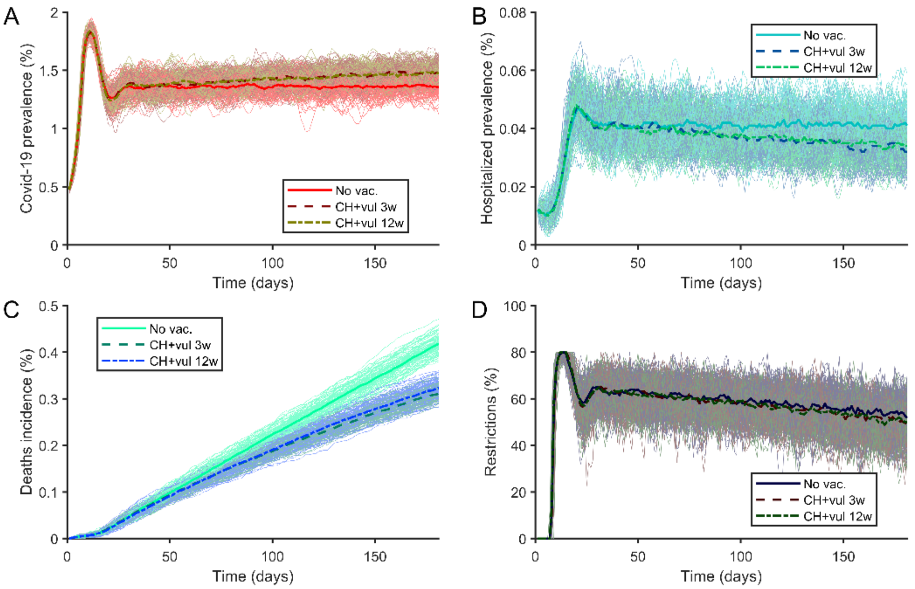

Figure 39. (A) Prevalence of covid-19 cases. (B) Prevalence of hospitalized covid-19 patients. (C) Covid-19 deaths. (D) Restrictions. Solid line for a non-vaccinated population. Dashed line for a population where they are vaccinating first care homes then vulnerable population. Dashed line for doses separation of 3 weeks. Dash-dot line for doses separation of 12 weeks. Figures for Pfizer vaccine using Greece conditions described in table 1.

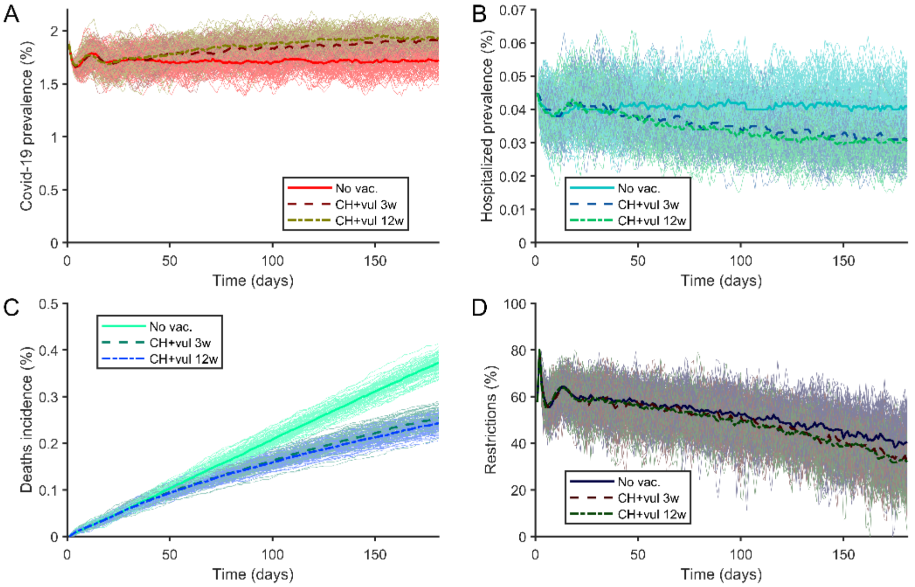

Figure 40. (A) Prevalence of covid-19 cases. (B) Prevalence of hospitalized covid-19 patients. (C) Covid-19 deaths. (D) Restrictions. Solid line for a non-vaccinated population. Dashed line for a population where they are vaccinating first care homes then vulnerable population. Dashed line for doses separation of 3 weeks. Dash-dot line for doses separation of 12 weeks. Figures for AstraZeneca vaccine using Hungary conditions described in table 1.

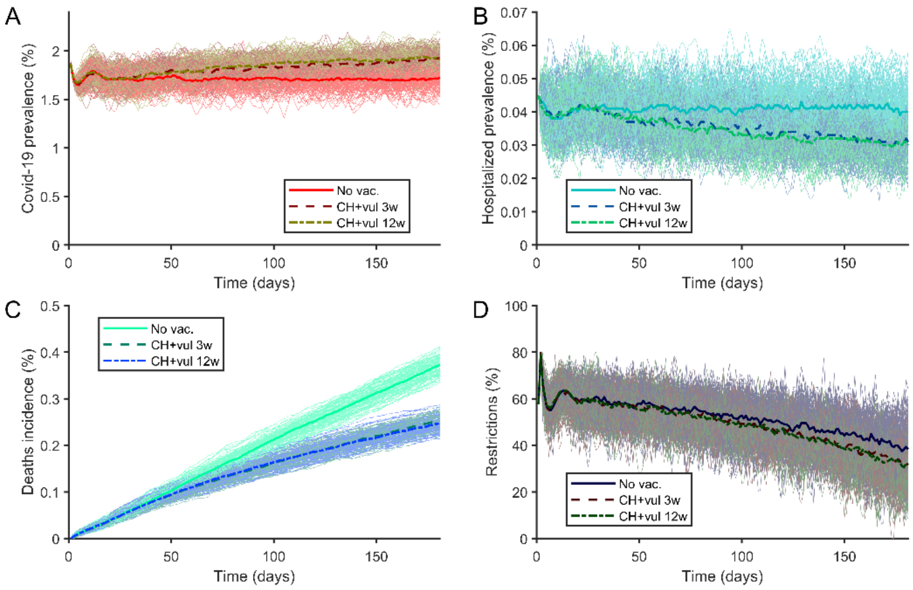

Figure 41. (A) Prevalence of covid-19 cases. (B) Prevalence of hospitalized covid-19 patients. (C) Covid-19 deaths. (D) Restrictions. Solid line for a non-vaccinated population. Dashed line for a population where they are vaccinating first care homes then vulnerable population. Dashed line for doses separation of 3 weeks. Dash-dot line for doses separation of 12 weeks. Figures for Moderna vaccine using Hungary conditions described in table 1.

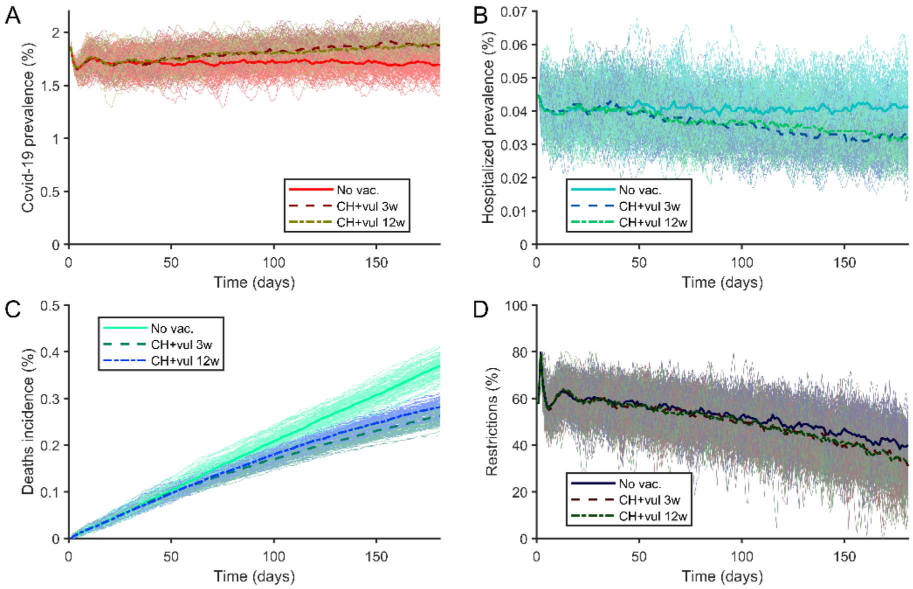

Figure 42. (A) Prevalence of covid-19 cases. (B) Prevalence of hospitalized covid-19 patients. (C) Covid-19 deaths. (D) Restrictions. Solid line for a non-vaccinated population. Dashed line for a population where they are vaccinating first care homes then vulnerable population. Dashed line for doses separation of 3 weeks. Dash-dot line for doses separation of 12 weeks. Figures for Pfizer vaccine using Hungary conditions described in table 1.

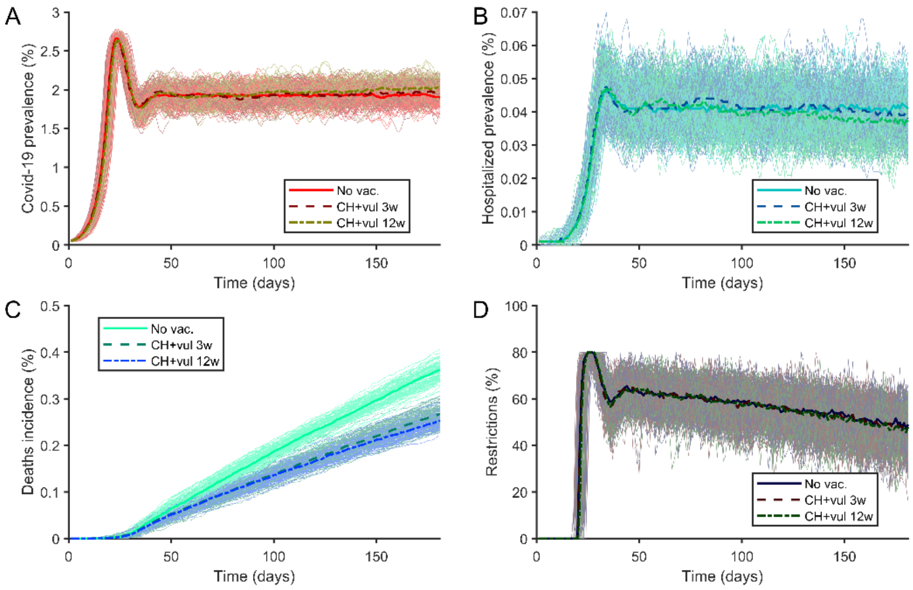

Figure 43. (A) Prevalence of covid-19 cases. (B) Prevalence of hospitalized covid-19 patients. (C) Covid-19 deaths. (D) Restrictions. Solid line for a non-vaccinated population. Dashed line for a population where they are vaccinating first care homes then vulnerable population. Dashed line for doses separation of 3 weeks. Dash-dot line for doses separation of 12 weeks. Figures for AstraZeneca vaccine using Iceland conditions described in table 1.

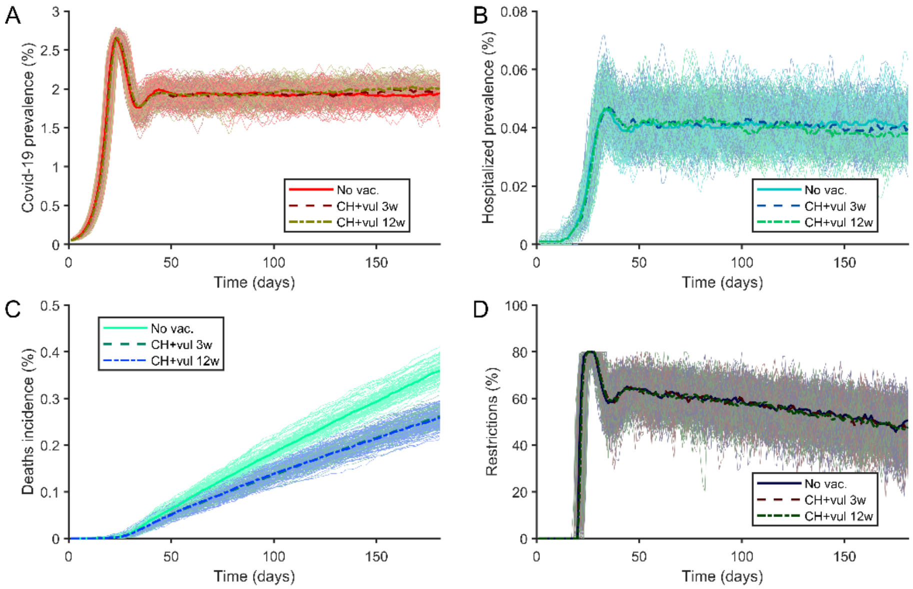

Figure 44. (A) Prevalence of covid-19 cases. (B) Prevalence of hospitalized covid-19 patients. (C) Covid-19 deaths. (D) Restrictions. Solid line for a non-vaccinated population. Dashed line for a population where they are vaccinating first care homes then vulnerable population. Dashed line for doses separation of 3 weeks. Dash-dot line for doses separation of 12 weeks. Figures for Moderna vaccine using Iceland conditions described in table 1.

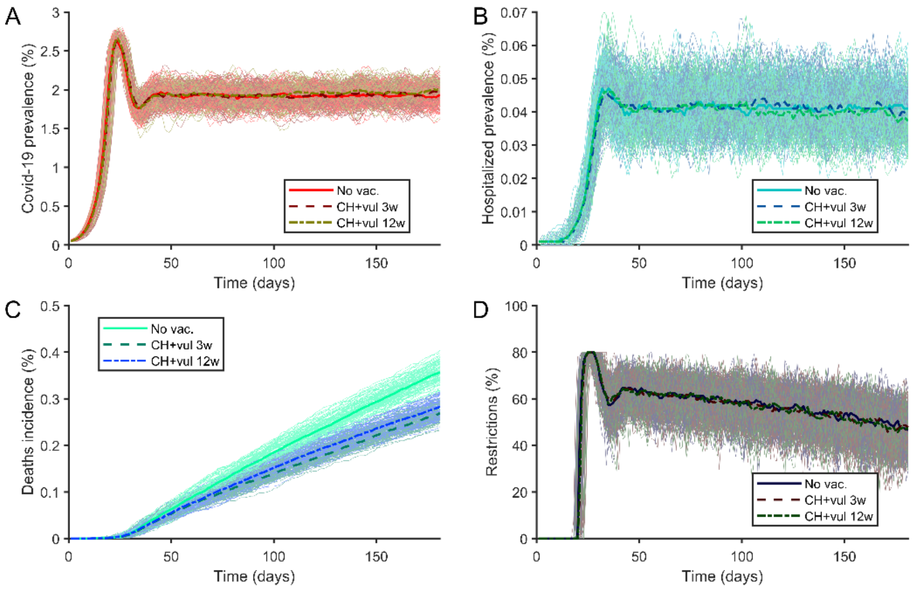

Figure 45. (A) Prevalence of covid-19 cases. (B) Prevalence of hospitalized covid-19 patients. (C) Covid-19 deaths. (D) Restrictions. Solid line for a non-vaccinated population. Dashed line for a population where they are vaccinating first care homes then vulnerable population. Dashed line for doses separation of 3 weeks. Dash-dot line for doses separation of 12 weeks. Figures for Pfizer vaccine using Iceland conditions described in table 1.

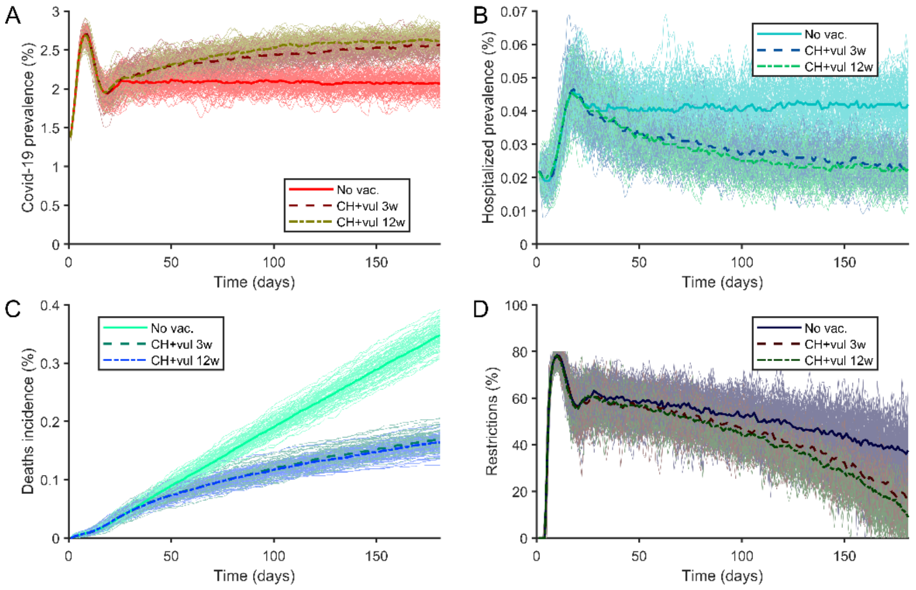

Figure 46. (A) Prevalence of covid-19 cases. (B) Prevalence of hospitalized covid-19 patients. (C) Covid-19 deaths. (D) Restrictions. Solid line for a non-vaccinated population. Dashed line for a population where they are vaccinating first care homes then vulnerable population. Dashed line for doses separation of 3 weeks. Dash-dot line for doses separation of 12 weeks. Figures for AstraZeneca vaccine using Ireland conditions described in table 1.

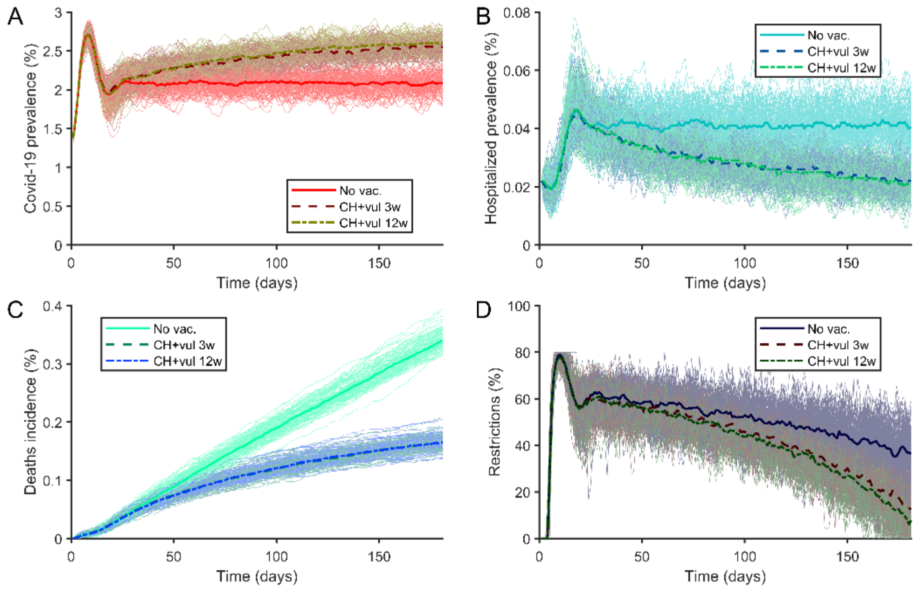

Figure 47. (A) Prevalence of covid-19 cases. (B) Prevalence of hospitalized covid-19 patients. (C) Covid-19 deaths. (D) Restrictions. Solid line for a non-vaccinated population. Dashed line for a population where they are vaccinating first care homes then vulnerable population. Dashed line for doses separation of 3 weeks. Dash-dot line for doses separation of 12 weeks. Figures for Moderna vaccine using Ireland conditions described in table 1.

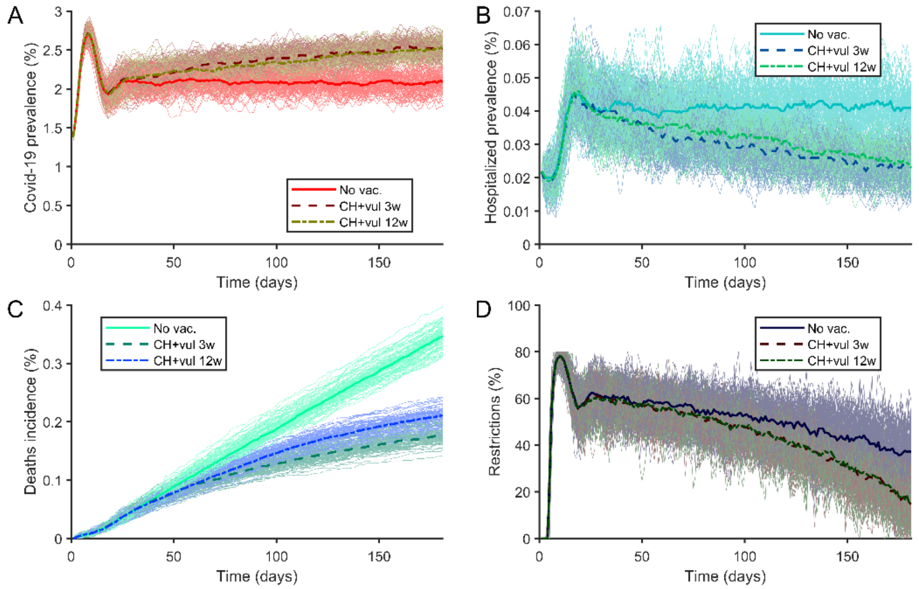

Figure 48. (A) Prevalence of covid-19 cases. (B) Prevalence of hospitalized covid-19 patients. (C) Covid-19 deaths. (D) Restrictions. Solid line for a non-vaccinated population. Dashed line for a population where they are vaccinating first care homes then vulnerable population. Dashed line for doses separation of 3 weeks. Dash-dot line for doses separation of 12 weeks. Figures for Pfizer vaccine using Ireland conditions described in table 1.

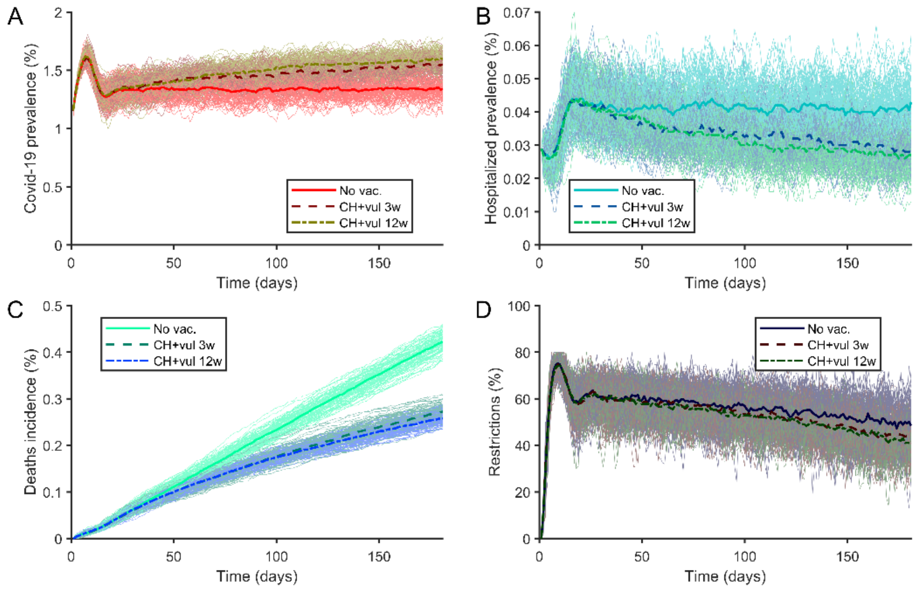

Figure 49. (A) Prevalence of covid-19 cases. (B) Prevalence of hospitalized covid-19 patients. (C) Covid-19 deaths. (D) Restrictions. Solid line for a non-vaccinated population. Dashed line for a population where they are vaccinating first care homes then vulnerable population. Dashed line for doses separation of 3 weeks. Dash-dot line for doses separation of 12 weeks. Figures for AstraZeneca vaccine using Italy conditions described in table 1.

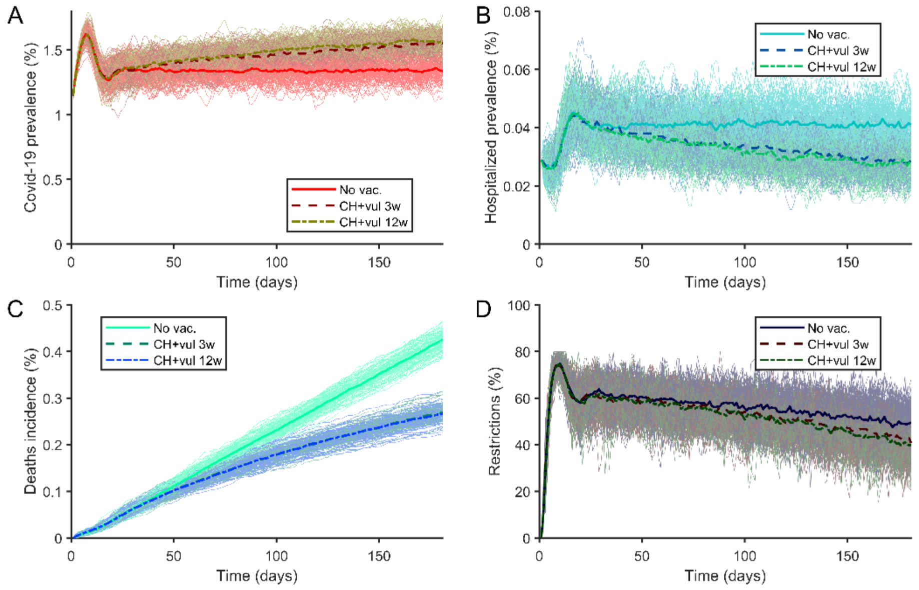

Figure 50. (A) Prevalence of covid-19 cases. (B) Prevalence of hospitalized covid-19 patients. (C) Covid-19 deaths. (D) Restrictions. Solid line for a non-vaccinated population. Dashed line for a population where they are vaccinating first care homes then vulnerable population. Dashed line for doses separation of 3 weeks. Dash-dot line for doses separation of 12 weeks. Figures for Moderna vaccine using Italy conditions described in table 1.

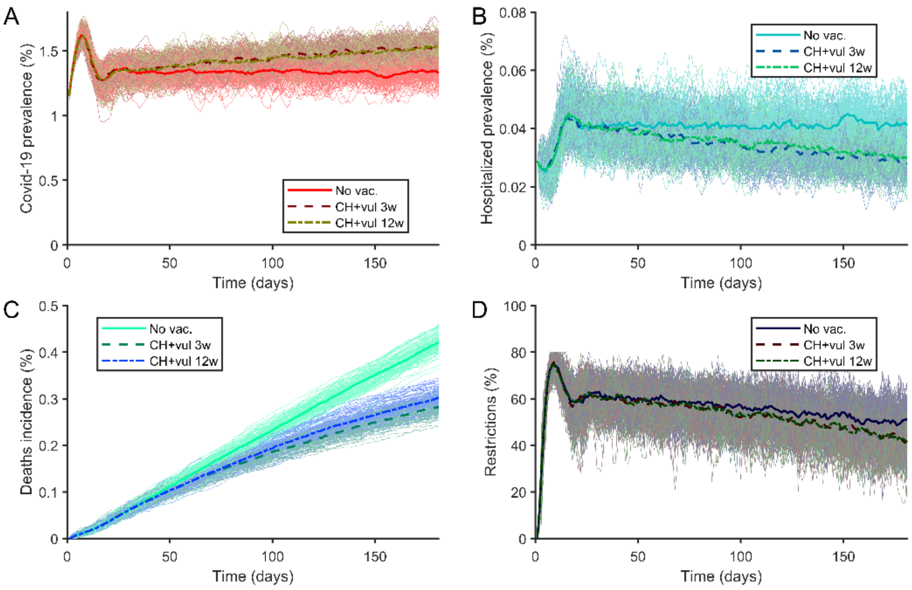

Figure 51. (A) Prevalence of covid-19 cases. (B) Prevalence of hospitalized covid-19 patients. (C) Covid-19 deaths. (D) Restrictions. Solid line for a non-vaccinated population. Dashed line for a population where they are vaccinating first care homes then vulnerable population. Dashed line for doses separation of 3 weeks. Dash-dot line for doses separation of 12 weeks. Figures for Pfizer vaccine using Italy conditions described in table 1.

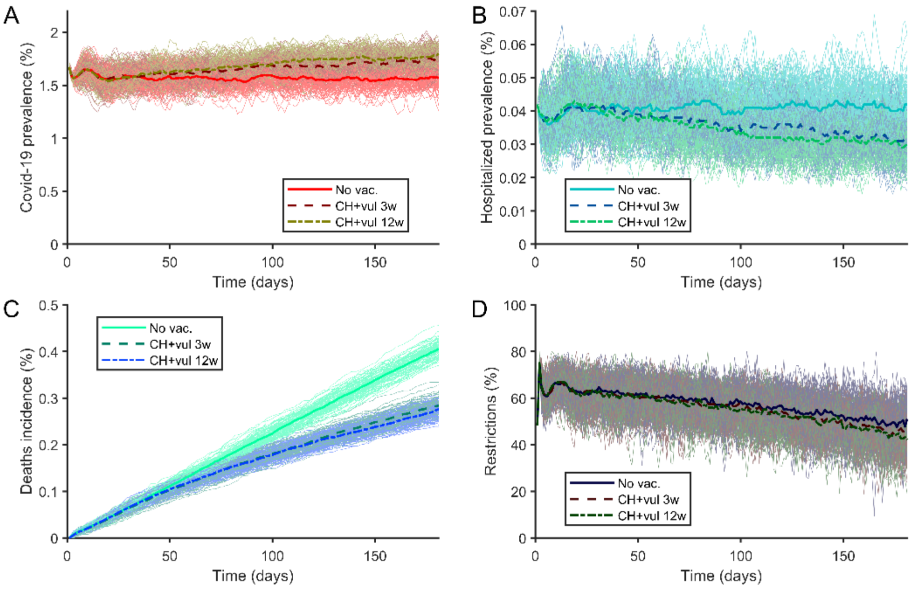

Figure 52. (A) Prevalence of covid-19 cases. (B) Prevalence of hospitalized covid-19 patients. (C) Covid-19 deaths. (D) Restrictions. Solid line for a non-vaccinated population. Dashed line for a population where they are vaccinating first care homes then vulnerable population. Dashed line for doses separation of 3 weeks. Dash-dot line for doses separation of 12 weeks. Figures for AstraZeneca vaccine using Latvia conditions described in table 1.

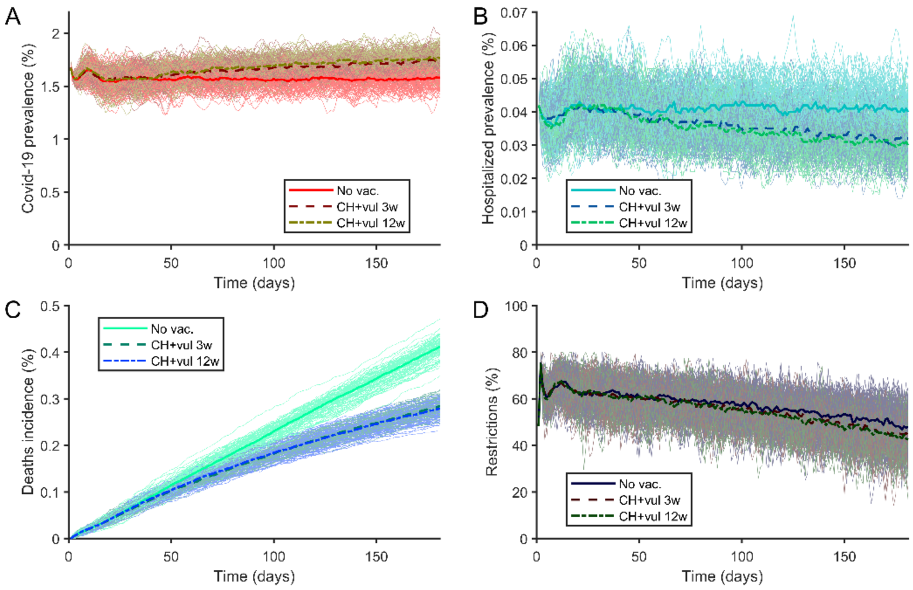

Figure 53. (A) Prevalence of covid-19 cases. (B) Prevalence of hospitalized covid-19 patients. (C) Covid-19 deaths. (D) Restrictions. Solid line for a non-vaccinated population. Dashed line for a population where they are vaccinating first care homes then vulnerable population. Dashed line for doses separation of 3 weeks. Dash-dot line for doses separation of 12 weeks. Figures for Moderna vaccine using Latvia conditions described in table 1.

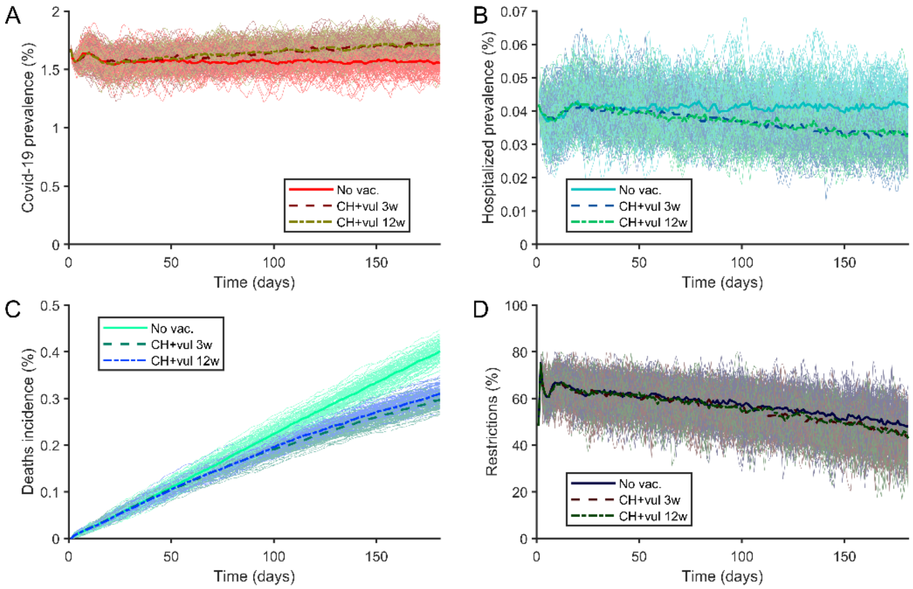

Figure 54. (A) Prevalence of covid-19 cases. (B) Prevalence of hospitalized covid-19 patients. (C) Covid-19 deaths. (D) Restrictions. Solid line for a non-vaccinated population. Dashed line for a population where they are vaccinating first care homes then vulnerable population. Dashed line for doses separation of 3 weeks. Dash-dot line for doses separation of 12 weeks. Figures for Pfizer vaccine using Latvia conditions described in table 1.

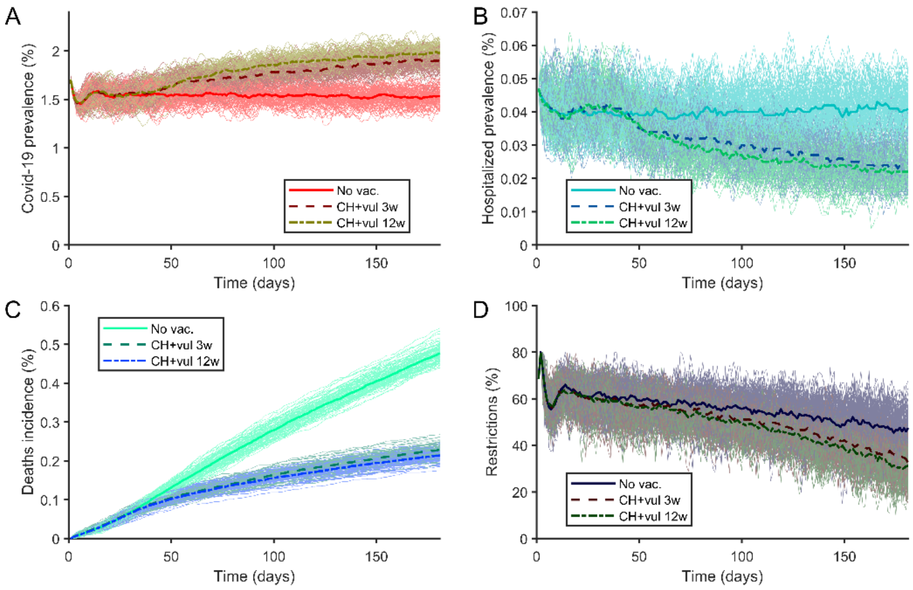

Figure 55. (A) Prevalence of covid-19 cases. (B) Prevalence of hospitalized covid-19 patients. (C) Covid-19 deaths. (D) Restrictions. Solid line for a non-vaccinated population. Dashed line for a population where they are vaccinating first care homes then vulnerable population. Dashed line for doses separation of 3 weeks. Dash-dot line for doses separation of 12 weeks. Figures for AstraZeneca vaccine using Lithuania conditions described in table 1.

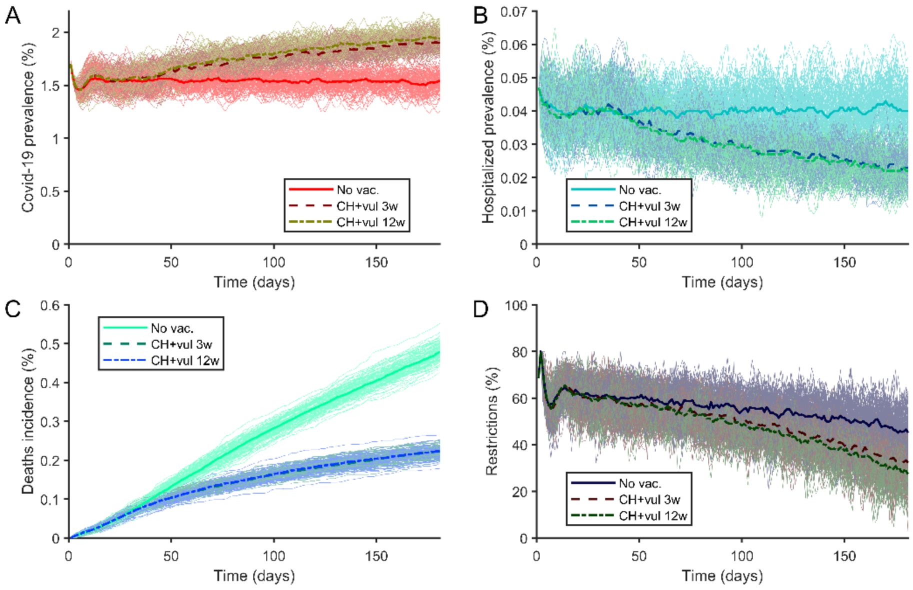

Figure 56. (A) Prevalence of covid-19 cases. (B) Prevalence of hospitalized covid-19 patients. (C) Covid-19 deaths. (D) Restrictions. Solid line for a non-vaccinated population. Dashed line for a population where they are vaccinating first care homes then vulnerable population. Dashed line for doses separation of 3 weeks. Dash-dot line for doses separation of 12 weeks. Figures for Moderna vaccine using Lithuania conditions described in table 1.

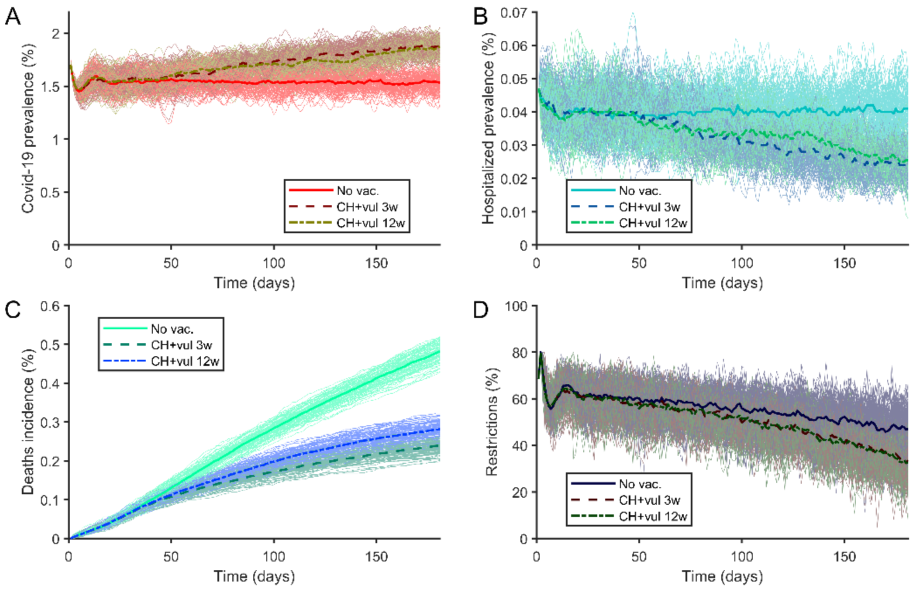

Figure 57. (A) Prevalence of covid-19 cases. (B) Prevalence of hospitalized covid-19 patients. (C) Covid-19 deaths. (D) Restrictions. Solid line for a non-vaccinated population. Dashed line for a population where they are vaccinating first care homes then vulnerable population. Dashed line for doses separation of 3 weeks. Dash-dot line for doses separation of 12 weeks. Figures for Pfizer vaccine using Lithuania conditions described in table 1.

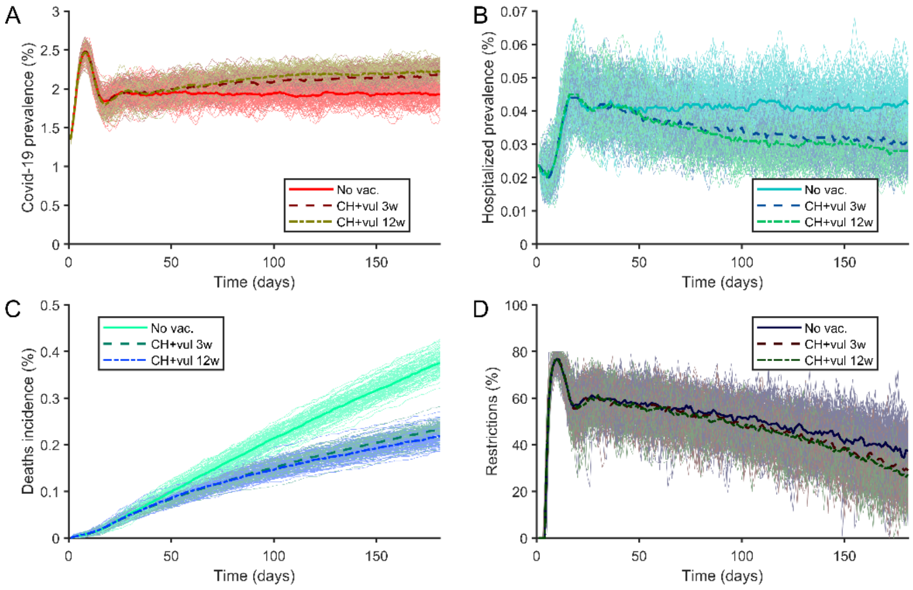

Figure 58. (A) Prevalence of covid-19 cases. (B) Prevalence of hospitalized covid-19 patients. (C) Covid-19 deaths. (D) Restrictions. Solid line for a non-vaccinated population. Dashed line for a population where they are vaccinating first care homes then vulnerable population. Dashed line for doses separation of 3 weeks. Dash-dot line for doses separation of 12 weeks. Figures for AstraZeneca vaccine using Luxembourg conditions described in table 1.

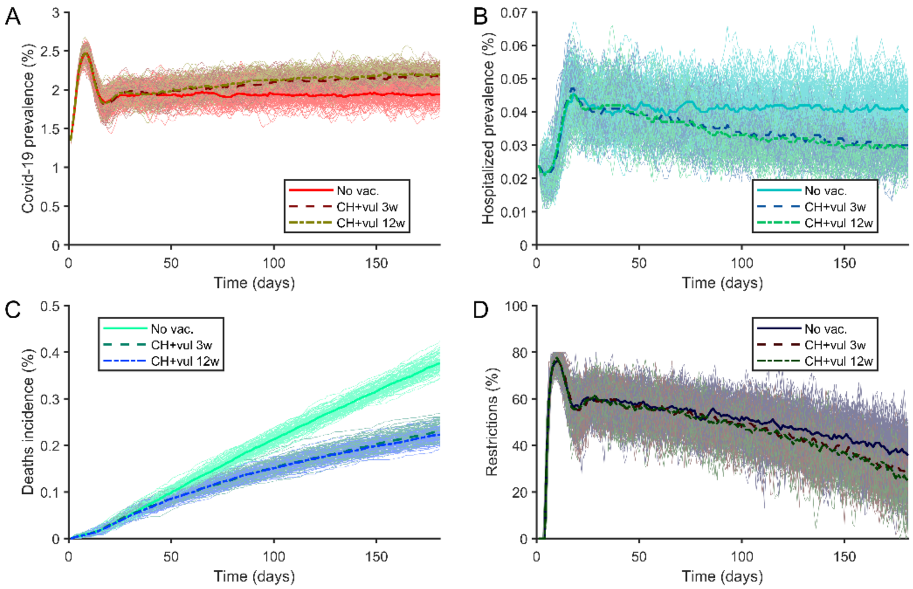

Figure 59. (A) Prevalence of covid-19 cases. (B) Prevalence of hospitalized covid-19 patients. (C) Covid-19 deaths. (D) Restrictions. Solid line for a non-vaccinated population. Dashed line for a population where they are vaccinating first care homes then vulnerable population. Dashed line for doses separation of 3 weeks. Dash-dot line for doses separation of 12 weeks. Figures for Moderna vaccine using Luxembourg conditions described in table 1.

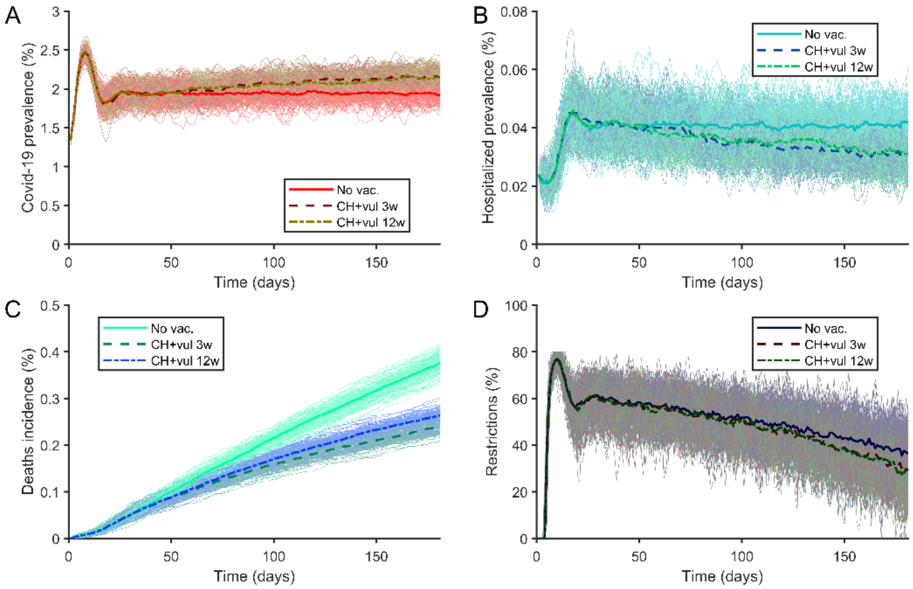

Figure 60. (A) Prevalence of covid-19 cases. (B) Prevalence of hospitalized covid-19 patients. (C) Covid-19 deaths. (D) Restrictions. Solid line for a non-vaccinated population. Dashed line for a population where they are vaccinating first care homes then vulnerable population. Dashed line for doses separation of 3 weeks. Dash-dot line for doses separation of 12 weeks. Figures for Pfizer vaccine using Luxembourg conditions described in table 1.

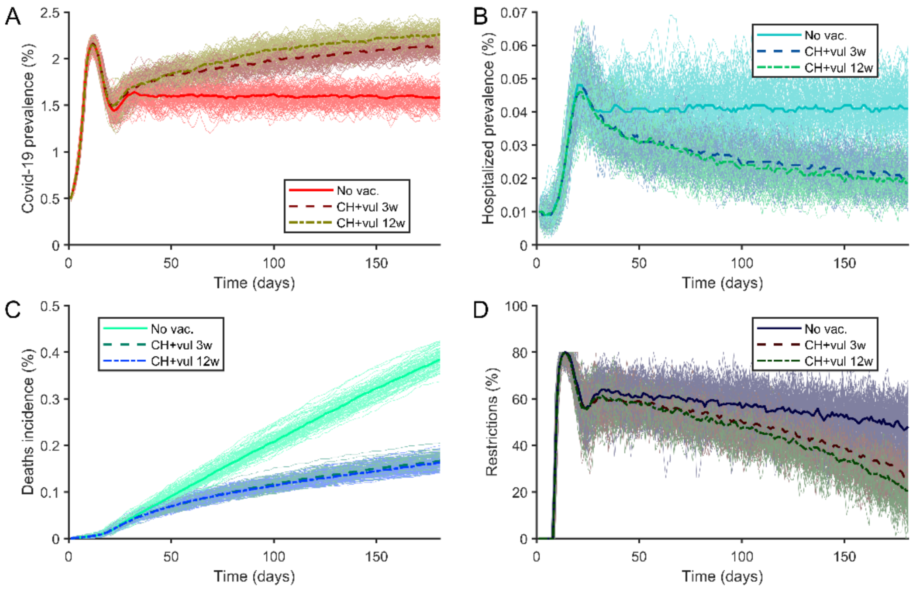

Figure 61. (A) Prevalence of covid-19 cases. (B) Prevalence of hospitalized covid-19 patients. (C) Covid-19 deaths. (D) Restrictions. Solid line for a non-vaccinated population. Dashed line for a population where they are vaccinating first care homes then vulnerable population. Dashed line for doses separation of 3 weeks. Dash-dot line for doses separation of 12 weeks. Figures for AstraZeneca vaccine using Malta conditions described in table 1.

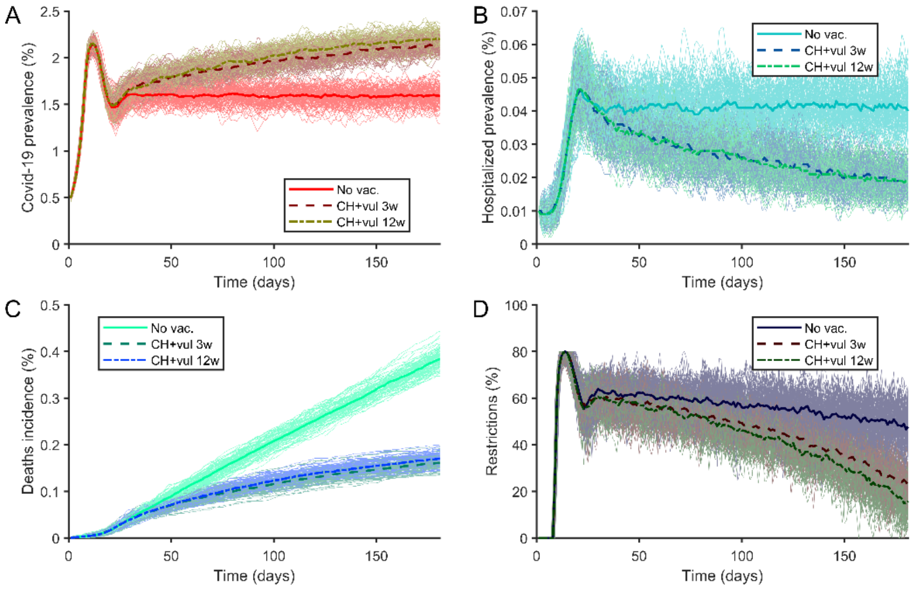

Figure 62. (A) Prevalence of covid-19 cases. (B) Prevalence of hospitalized covid-19 patients. (C) Covid-19 deaths. (D) Restrictions. Solid line for a non-vaccinated population. Dashed line for a population where they are vaccinating first care homes then vulnerable population. Dashed line for doses separation of 3 weeks. Dash-dot line for doses separation of 12 weeks. Figures for Moderna vaccine using Malta conditions described in table 1.

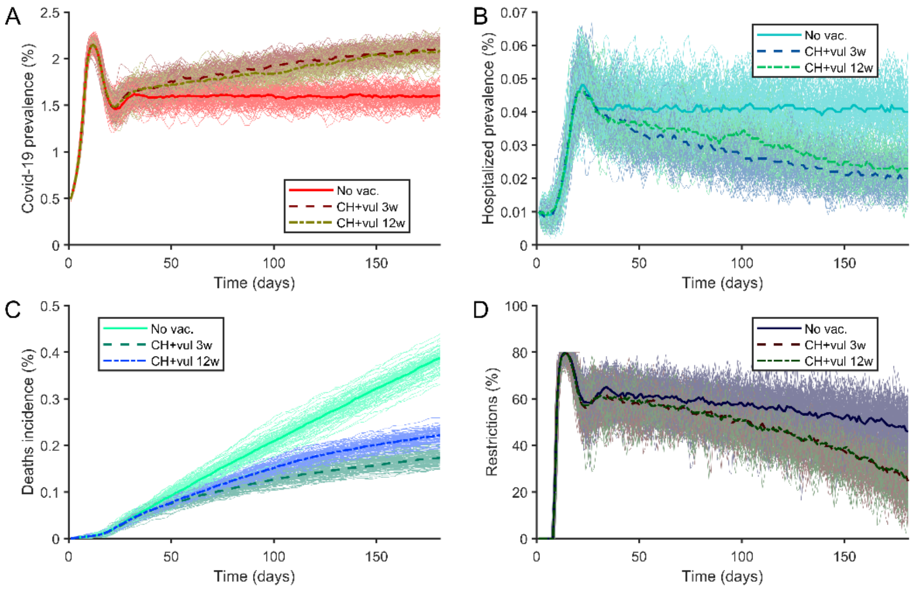

Figure 63. (A) Prevalence of covid-19 cases. (B) Prevalence of hospitalized covid-19 patients. (C) Covid-19 deaths. (D) Restrictions. Solid line for a non-vaccinated population. Dashed line for a population where they are vaccinating first care homes then vulnerable population. Dashed line for doses separation of 3 weeks. Dash-dot line for doses separation of 12 weeks. Figures for Pfizer vaccine using Malta conditions described in table 1.

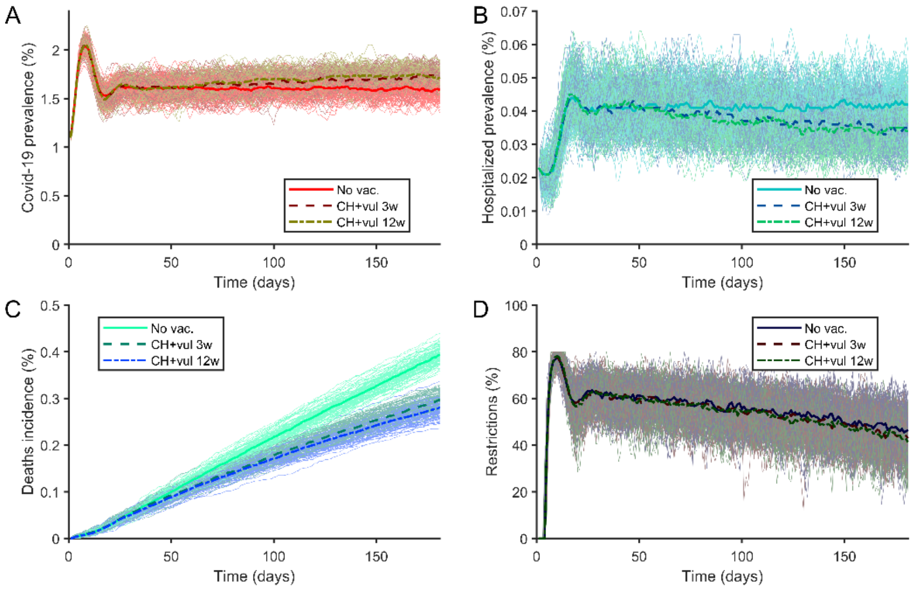

Figure 64. (A) Prevalence of covid-19 cases. (B) Prevalence of hospitalized covid-19 patients. (C) Covid-19 deaths. (D) Restrictions. Solid line for a non-vaccinated population. Dashed line for a population where they are vaccinating first care homes then vulnerable population. Dashed line for doses separation of 3 weeks. Dash-dot line for doses separation of 12 weeks. Figures for AstraZeneca vaccine using Netherlands conditions described in table 1.

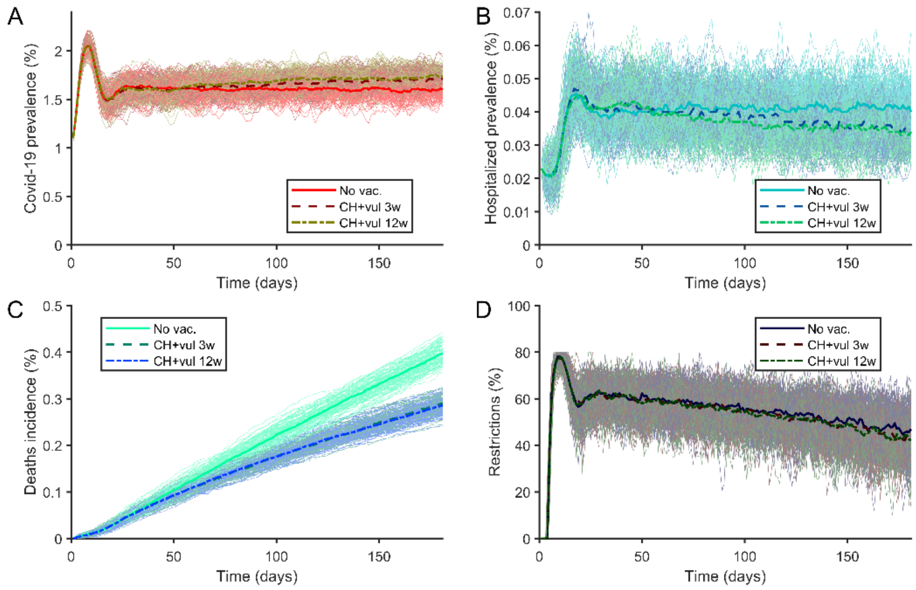

Figure 65. (A) Prevalence of covid-19 cases. (B) Prevalence of hospitalized covid-19 patients. (C) Covid-19 deaths. (D) Restrictions. Solid line for a non-vaccinated population. Dashed line for a population where they are vaccinating first care homes then vulnerable population. Dashed line for doses separation of 3 weeks. Dash-dot line for doses separation of 12 weeks. Figures for Moderna vaccine using Netherlands conditions described in table 1.

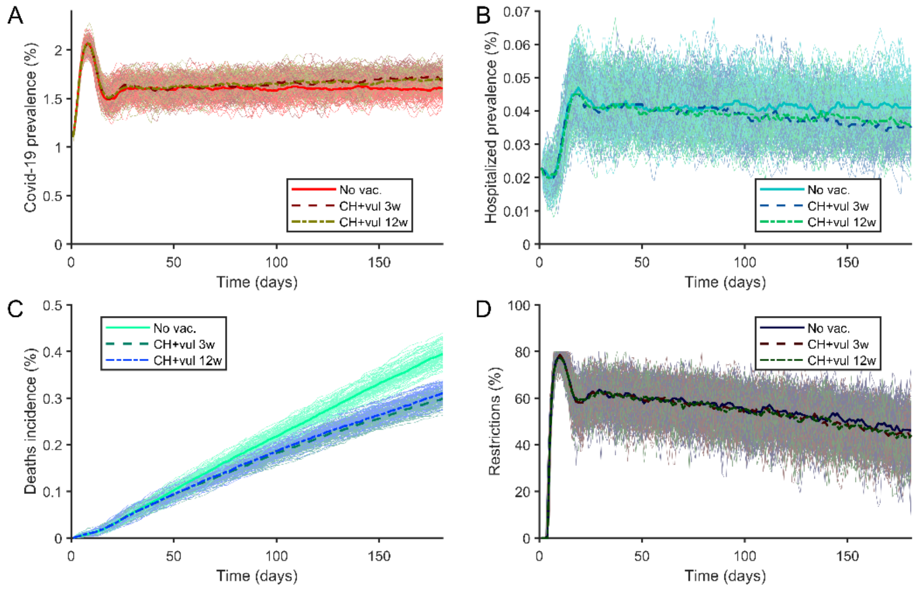

Figure 66. (A) Prevalence of covid-19 cases. (B) Prevalence of hospitalized covid-19 patients. (C) Covid-19 deaths. (D) Restrictions. Solid line for a non-vaccinated population. Dashed line for a population where they are vaccinating first care homes then vulnerable population. Dashed line for doses separation of 3 weeks. Dash-dot line for doses separation of 12 weeks. Figures for Pfizer vaccine using Netherlands conditions described in table 1.

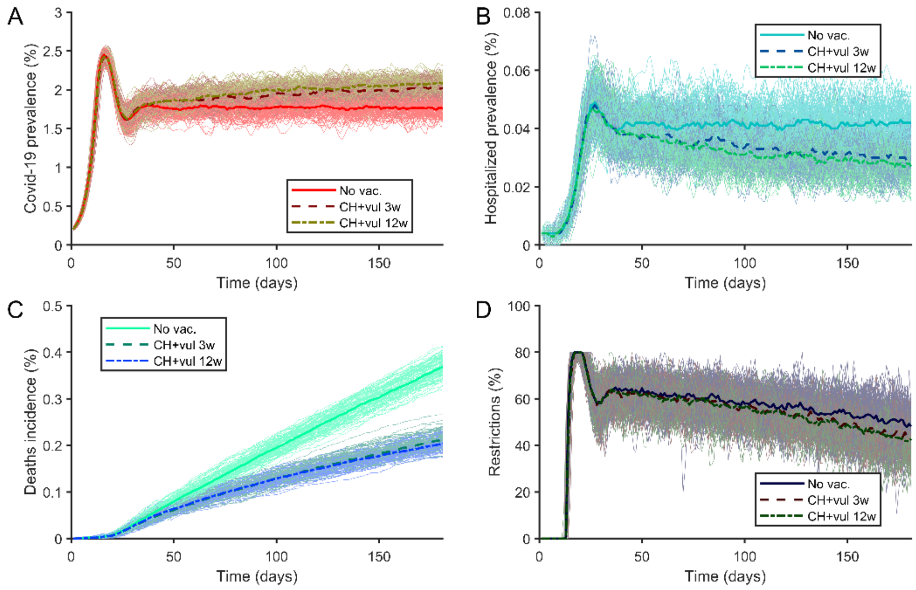

Figure 67. (A) Prevalence of covid-19 cases. (B) Prevalence of hospitalized covid-19 patients. (C) Covid-19 deaths. (D) Restrictions. Solid line for a non-vaccinated population. Dashed line for a population where they are vaccinating first care homes then vulnerable population. Dashed line for doses separation of 3 weeks. Dash-dot line for doses separation of 12 weeks. Figures for AstraZeneca vaccine using Norway conditions described in table 1.

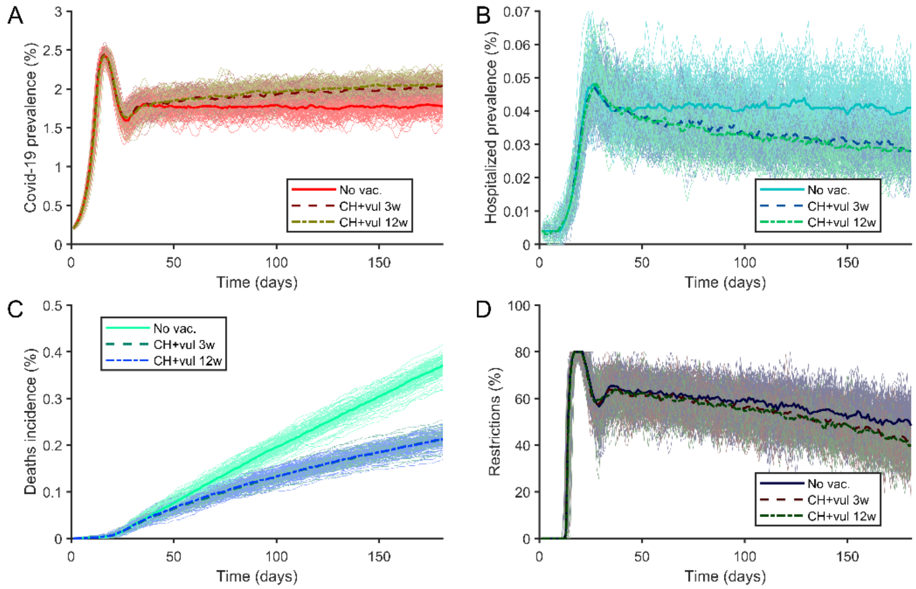

Figure 68. (A) Prevalence of covid-19 cases. (B) Prevalence of hospitalized covid-19 patients. (C) Covid-19 deaths. (D) Restrictions. Solid line for a non-vaccinated population. Dashed line for a population where they are vaccinating first care homes then vulnerable population. Dashed line for doses separation of 3 weeks. Dash-dot line for doses separation of 12 weeks. Figures for Moderna vaccine using Norway conditions described in table 1.

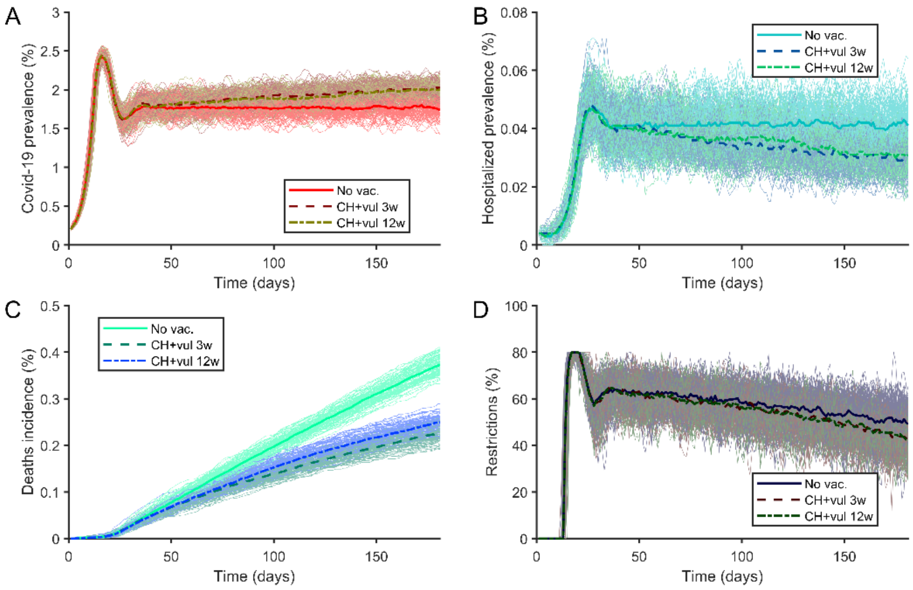

Figure 69. (A) Prevalence of covid-19 cases. (B) Prevalence of hospitalized covid-19 patients. (C) Covid-19 deaths. (D) Restrictions. Solid line for a non-vaccinated population. Dashed line for a population where they are vaccinating first care homes then vulnerable population. Dashed line for doses separation of 3 weeks. Dash-dot line for doses separation of 12 weeks. Figures for Pfizer vaccine using Norway conditions described in table 1.

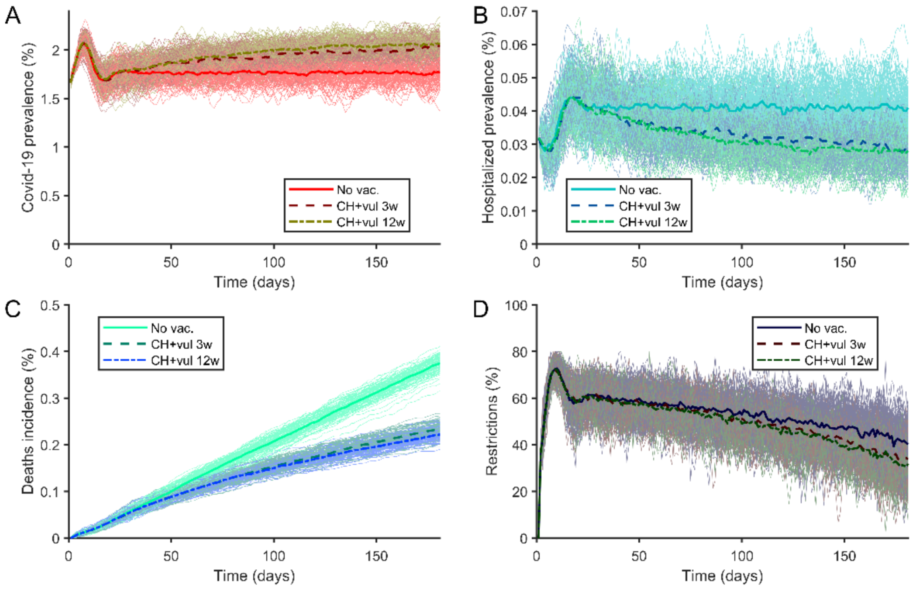

Figure 70. (A) Prevalence of covid-19 cases. (B) Prevalence of hospitalized covid-19 patients. (C) Covid-19 deaths. (D) Restrictions. Solid line for a non-vaccinated population. Dashed line for a population where they are vaccinating first care homes then vulnerable population. Dashed line for doses separation of 3 weeks. Dash-dot line for doses separation of 12 weeks. Figures for AstraZeneca vaccine using Poland conditions described in table 1.

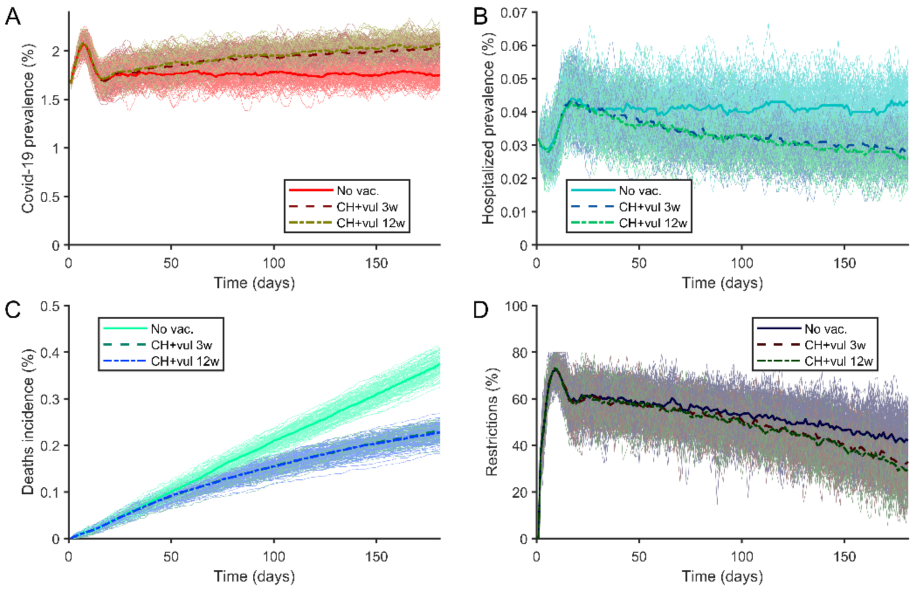

Figure 71. (A) Prevalence of covid-19 cases. (B) Prevalence of hospitalized covid-19 patients. (C) Covid-19 deaths. (D) Restrictions. Solid line for a non-vaccinated population. Dashed line for a population where they are vaccinating first care homes then vulnerable population. Dashed line for doses separation of 3 weeks. Dash-dot line for doses separation of 12 weeks. Figures for Moderna vaccine using Poland conditions described in table 1.

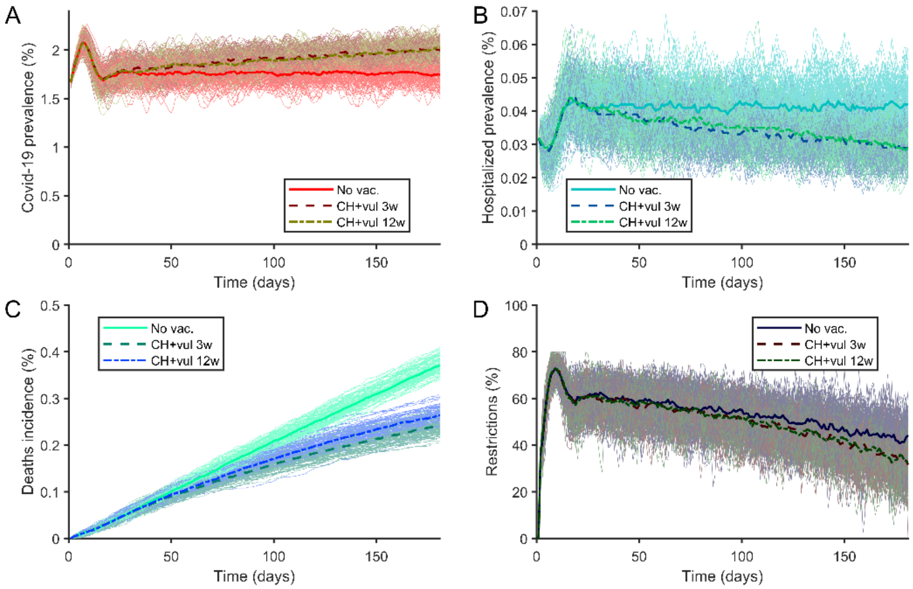

Figure 72. (A) Prevalence of covid-19 cases. (B) Prevalence of hospitalized covid-19 patients. (C) Covid-19 deaths. (D) Restrictions. Solid line for a non-vaccinated population. Dashed line for a population where they are vaccinating first care homes then vulnerable population. Dashed line for doses separation of 3 weeks. Dash-dot line for doses separation of 12 weeks. Figures for Pfizer vaccine using Poland conditions described in table 1.

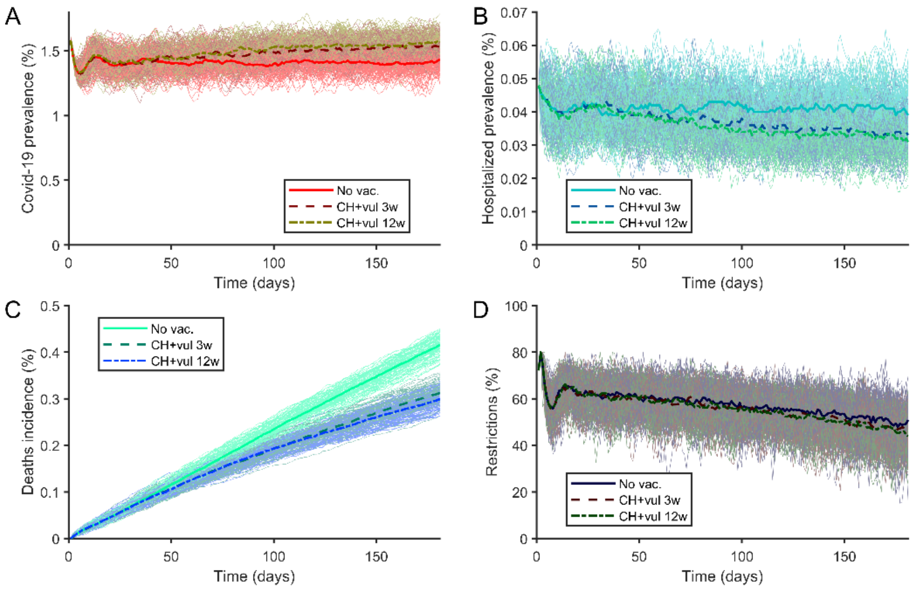

Figure 73. (A) Prevalence of covid-19 cases. (B) Prevalence of hospitalized covid-19 patients. (C) Covid-19 deaths. (D) Restrictions. Solid line for a non-vaccinated population. Dashed line for a population where they are vaccinating first care homes then vulnerable population. Dashed line for doses separation of 3 weeks. Dash-dot line for doses separation of 12 weeks. Figures for AstraZeneca vaccine using Portugal conditions described in table 1.

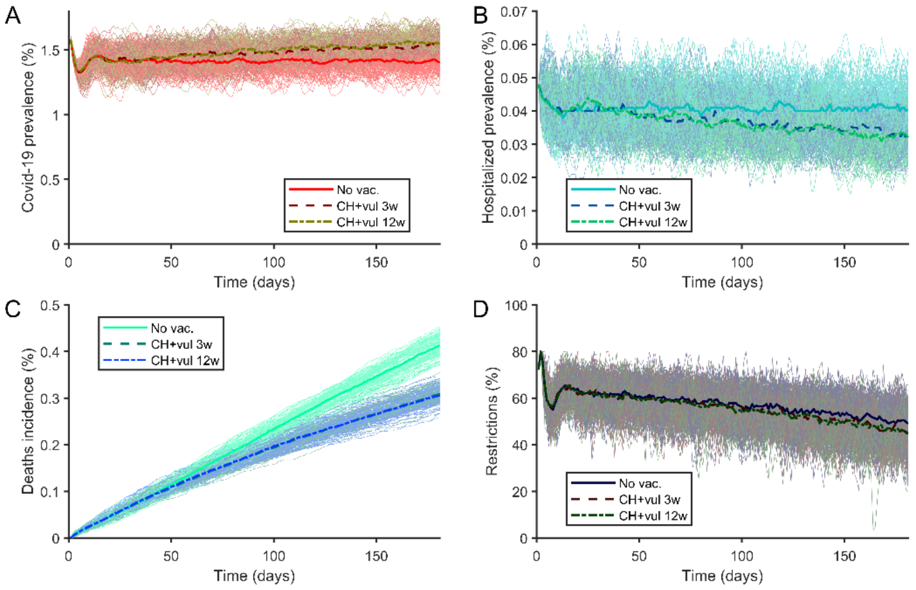

Figure 74. (A) Prevalence of covid-19 cases. (B) Prevalence of hospitalized covid-19 patients. (C) Covid-19 deaths. (D) Restrictions. Solid line for a non-vaccinated population. Dashed line for a population where they are vaccinating first care homes then vulnerable population. Dashed line for doses separation of 3 weeks. Dash-dot line for doses separation of 12 weeks. Figures for Moderna vaccine using Portugal conditions described in table 1.

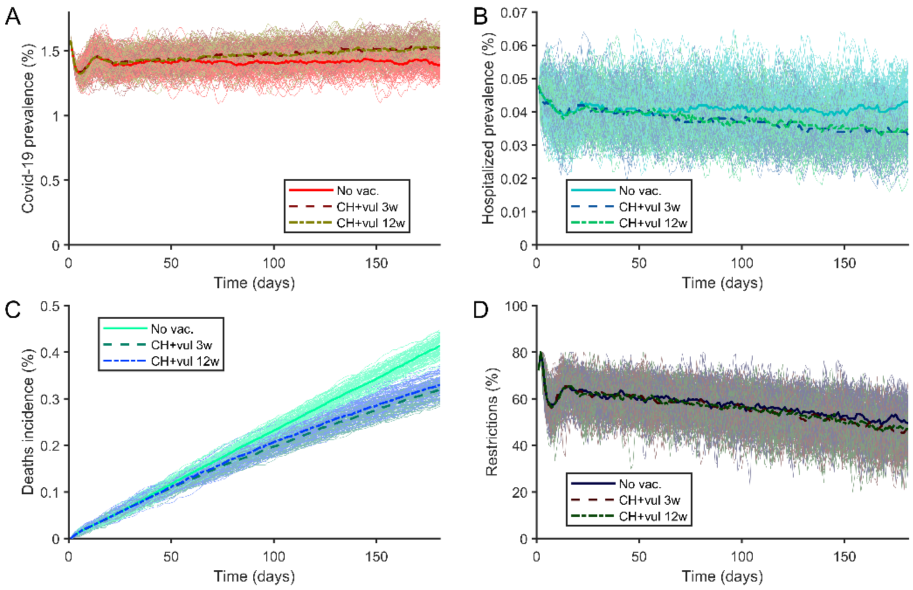

Figure 75. (A) Prevalence of covid-19 cases. (B) Prevalence of hospitalized covid-19 patients. (C) Covid-19 deaths. (D) Restrictions. Solid line for a non-vaccinated population. Dashed line for a population where they are vaccinating first care homes then vulnerable population. Dashed line for doses separation of 3 weeks. Dash-dot line for doses separation of 12 weeks. Figures for Pfizer vaccine using Portugal conditions described in table 1.

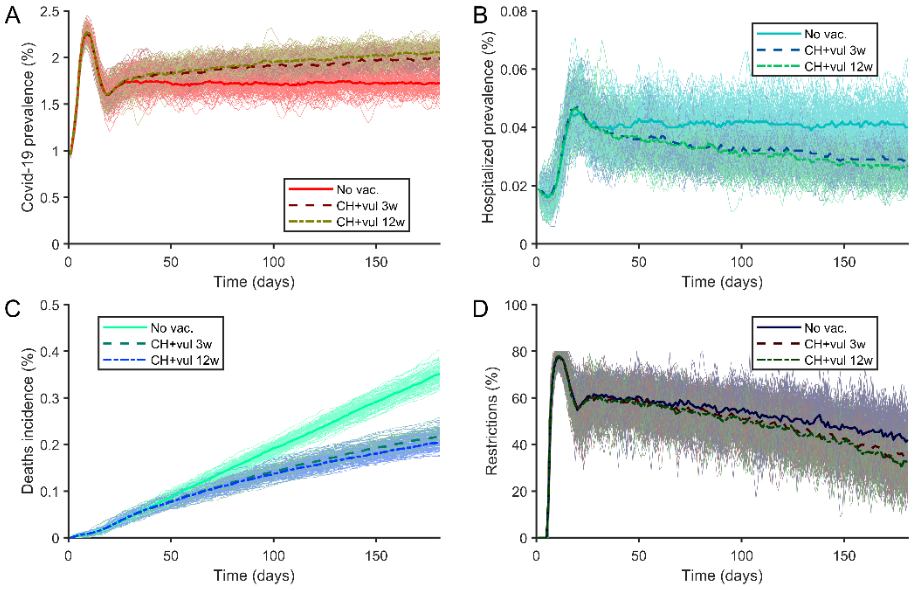

Figure 76. (A) Prevalence of covid-19 cases. (B) Prevalence of hospitalized covid-19 patients. (C) Covid-19 deaths. (D) Restrictions. Solid line for a non-vaccinated population. Dashed line for a population where they are vaccinating first care homes then vulnerable population. Dashed line for doses separation of 3 weeks. Dash-dot line for doses separation of 12 weeks. Figures for AstraZeneca vaccine using Romania conditions described in table 1.

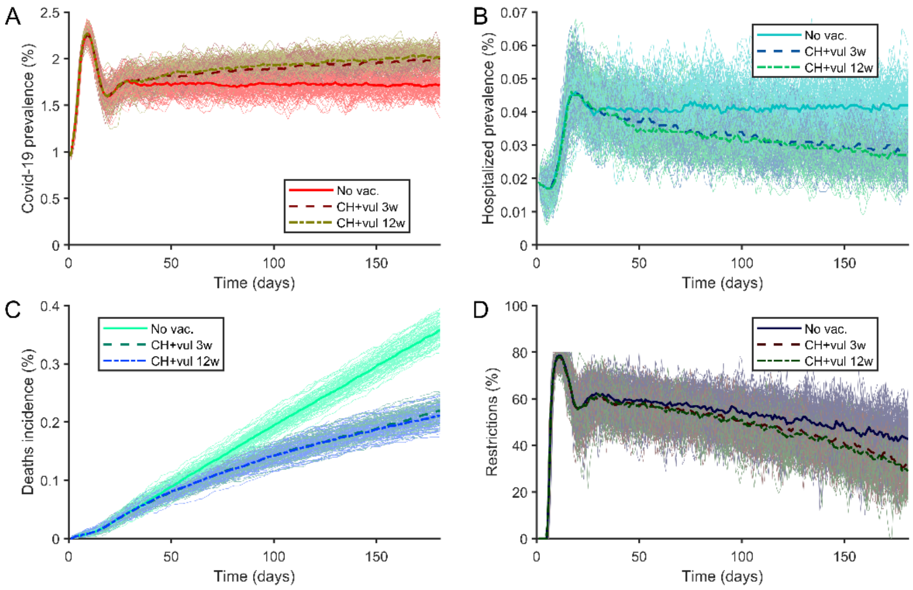

Figure 77. (A) Prevalence of covid-19 cases. (B) Prevalence of hospitalized covid-19 patients. (C) Covid-19 deaths. (D) Restrictions. Solid line for a non-vaccinated population. Dashed line for a population where they are vaccinating first care homes then vulnerable population. Dashed line for doses separation of 3 weeks. Dash-dot line for doses separation of 12 weeks. Figures for Moderna vaccine using Romania conditions described in table 1.

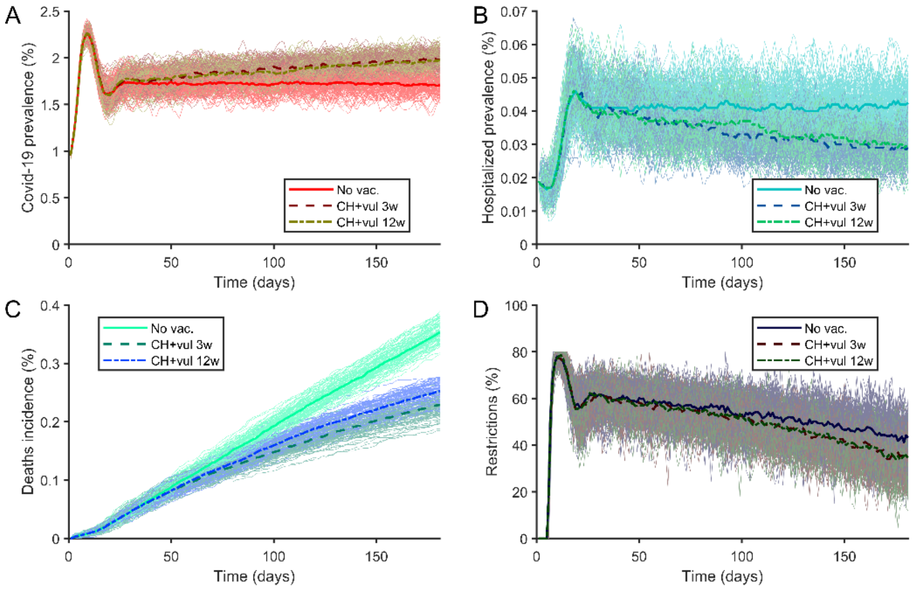

Figure 78. (A) Prevalence of covid-19 cases. (B) Prevalence of hospitalized covid-19 patients. (C) Covid-19 deaths. (D) Restrictions. Solid line for a non-vaccinated population. Dashed line for a population where they are vaccinating first care homes then vulnerable population. Dashed line for doses separation of 3 weeks. Dash-dot line for doses separation of 12 weeks. Figures for Pfizer vaccine using Romania conditions described in table 1.

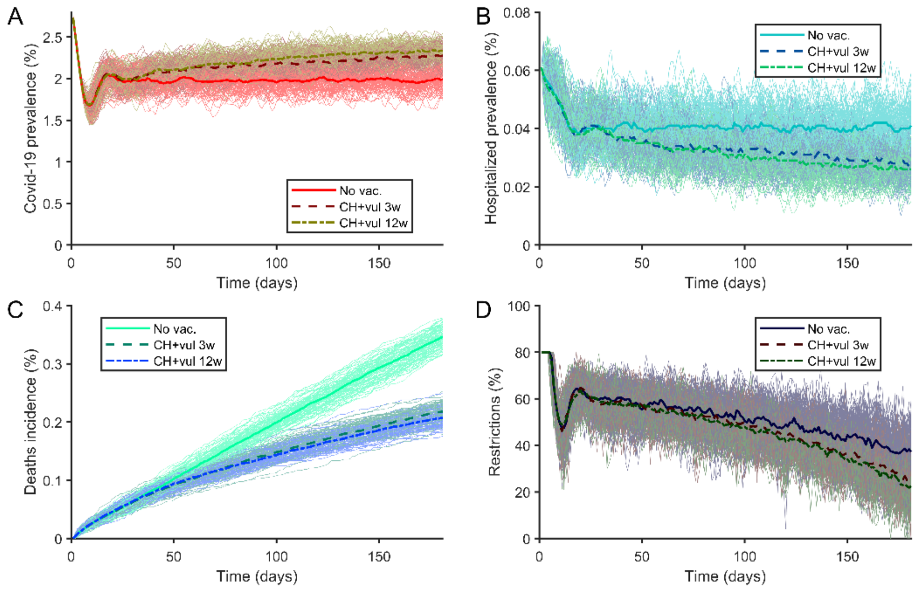

Figure 79. (A) Prevalence of covid-19 cases. (B) Prevalence of hospitalized covid-19 patients. (C) Covid-19 deaths. (D) Restrictions. Solid line for a non-vaccinated population. Dashed line for a population where they are vaccinating first care homes then vulnerable population. Dashed line for doses separation of 3 weeks. Dash-dot line for doses separation of 12 weeks. Figures for AstraZeneca vaccine using Slovakia conditions described in table 1.

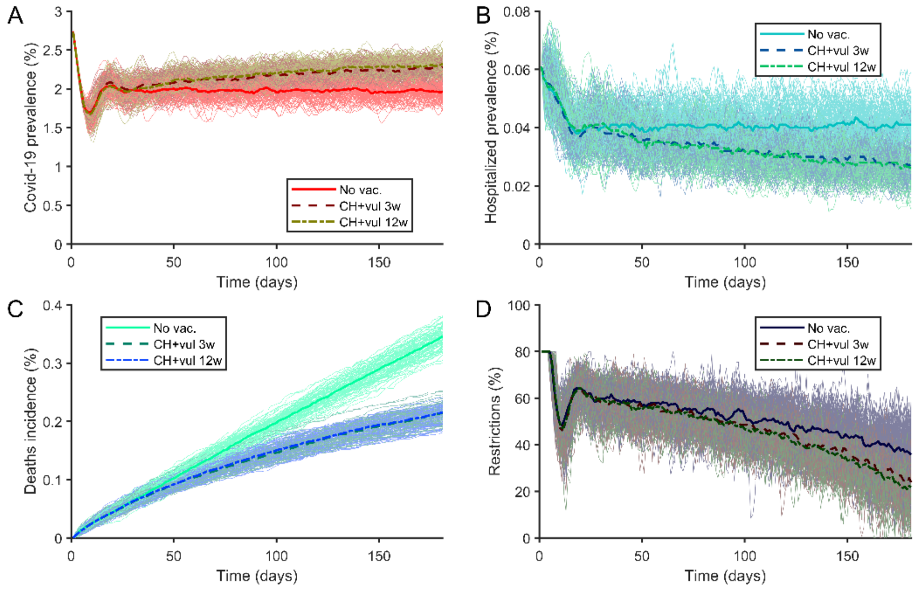

Figure 80. (A) Prevalence of covid-19 cases. (B) Prevalence of hospitalized covid-19 patients. (C) Covid-19 deaths. (D) Restrictions. Solid line for a non-vaccinated population. Dashed line for a population where they are vaccinating first care homes then vulnerable population. Dashed line for doses separation of 3 weeks. Dash-dot line for doses separation of 12 weeks. Figures for Moderna vaccine using Slovakia conditions described in table 1.

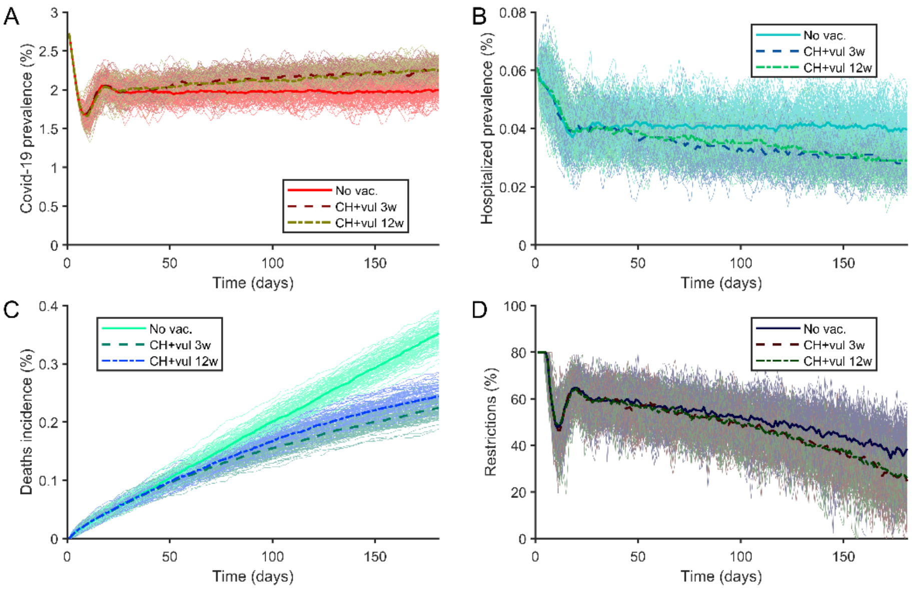

Figure 81. (A) Prevalence of covid-19 cases. (B) Prevalence of hospitalized covid-19 patients. (C) Covid-19 deaths. (D) Restrictions. Solid line for a non-vaccinated population. Dashed line for a population where they are vaccinating first care homes then vulnerable population. Dashed line for doses separation of 3 weeks. Dash-dot line for doses separation of 12 weeks. Figures for Pfizer vaccine using Slovakia conditions described in table 1.

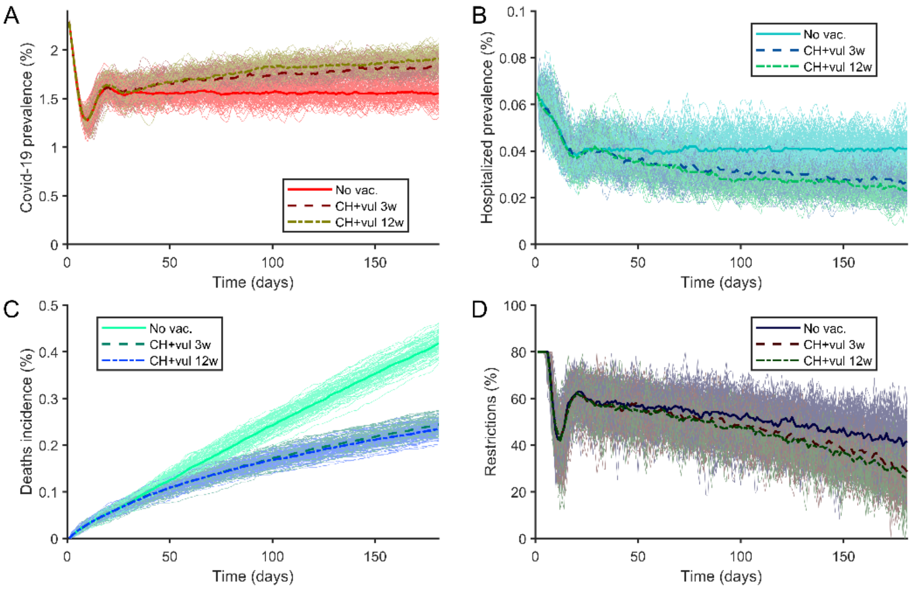

Figure 82. (A) Prevalence of covid-19 cases. (B) Prevalence of hospitalized covid-19 patients. (C) Covid-19 deaths. (D) Restrictions. Solid line for a non-vaccinated population. Dashed line for a population where they are vaccinating first care homes then vulnerable population. Dashed line for doses separation of 3 weeks. Dash-dot line for doses separation of 12 weeks. Figures for AstraZeneca vaccine using Slovenia conditions described in table 1.

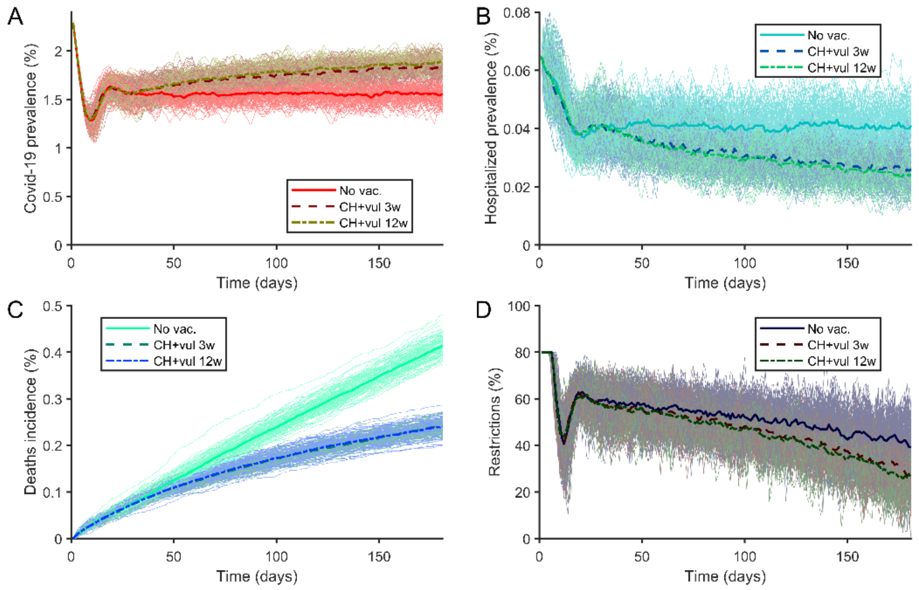

Figure 83. (A) Prevalence of covid-19 cases. (B) Prevalence of hospitalized covid-19 patients. (C) Covid-19 deaths. (D) Restrictions. Solid line for a non-vaccinated population. Dashed line for a population where they are vaccinating first care homes then vulnerable population. Dashed line for doses separation of 3 weeks. Dash-dot line for doses separation of 12 weeks. Figures for Moderna vaccine using Slovenia conditions described in table 1.

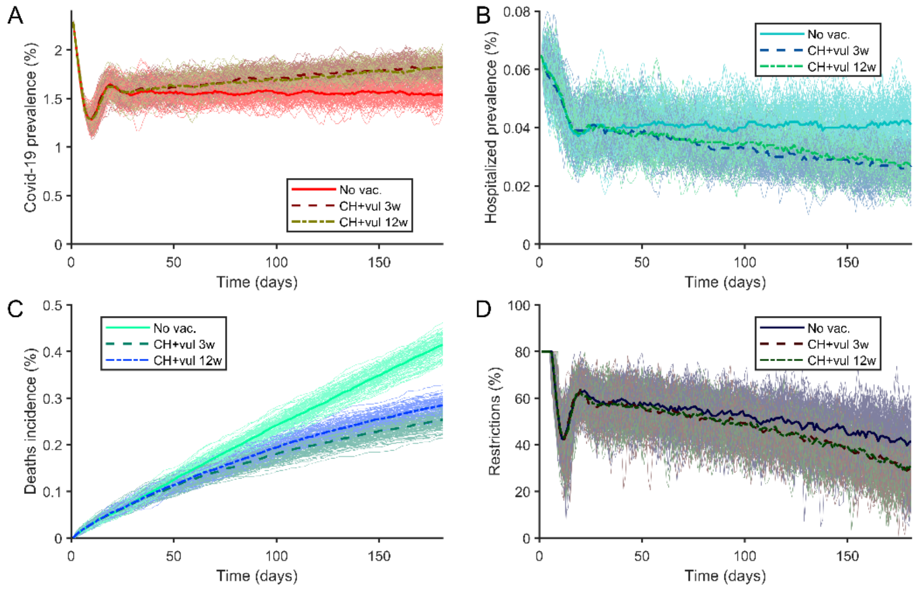

Figure 84. (A) Prevalence of covid-19 cases. (B) Prevalence of hospitalized covid-19 patients. (C) Covid-19 deaths. (D) Restrictions. Solid line for a non-vaccinated population. Dashed line for a population where they are vaccinating first care homes then vulnerable population. Dashed line for doses separation of 3 weeks. Dash-dot line for doses separation of 12 weeks. Figures for Pfizer vaccine using Slovenia conditions described in table 1.

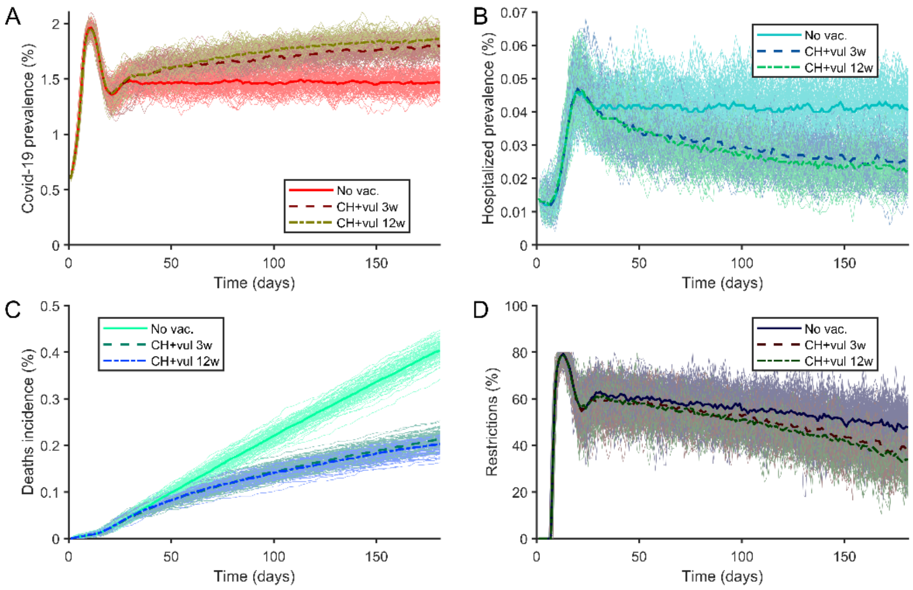

Figure 85. (A) Prevalence of covid-19 cases. (B) Prevalence of hospitalized covid-19 patients. (C) Covid-19 deaths. (D) Restrictions. Solid line for a non-vaccinated population. Dashed line for a population where they are vaccinating first care homes then vulnerable population. Dashed line for doses separation of 3 weeks. Dash-dot line for doses separation of 12 weeks. Figures for AstraZeneca vaccine using Spain conditions described in table 1.

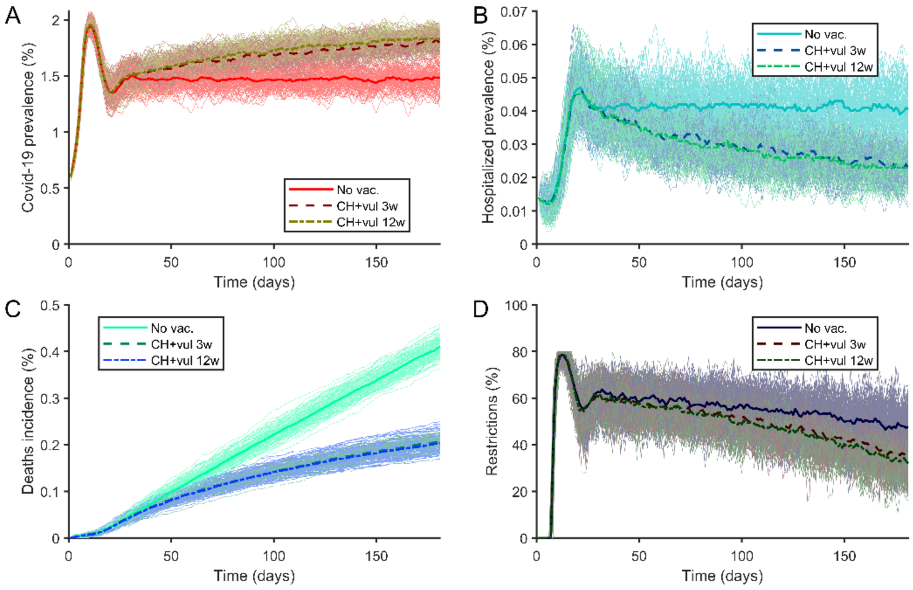

Figure 86. (A) Prevalence of covid-19 cases. (B) Prevalence of hospitalized covid-19 patients. (C) Covid-19 deaths. (D) Restrictions. Solid line for a non-vaccinated population. Dashed line for a population where they are vaccinating first care homes then vulnerable population. Dashed line for doses separation of 3 weeks. Dash-dot line for doses separation of 12 weeks. Figures for Moderna vaccine using Spain conditions described in table 1.

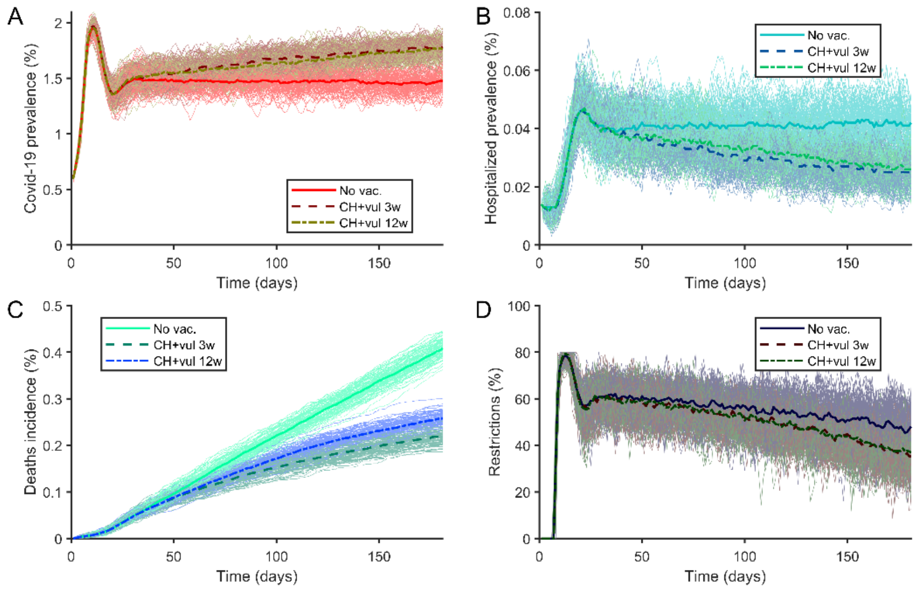

Figure 87. (A) Prevalence of covid-19 cases. (B) Prevalence of hospitalized covid-19 patients. (C) Covid-19 deaths. (D) Restrictions. Solid line for a non-vaccinated population. Dashed line for a population where they are vaccinating first care homes then vulnerable population. Dashed line for doses separation of 3 weeks. Dash-dot line for doses separation of 12 weeks. Figures for Pfizer vaccine using Spain conditions described in table 1.

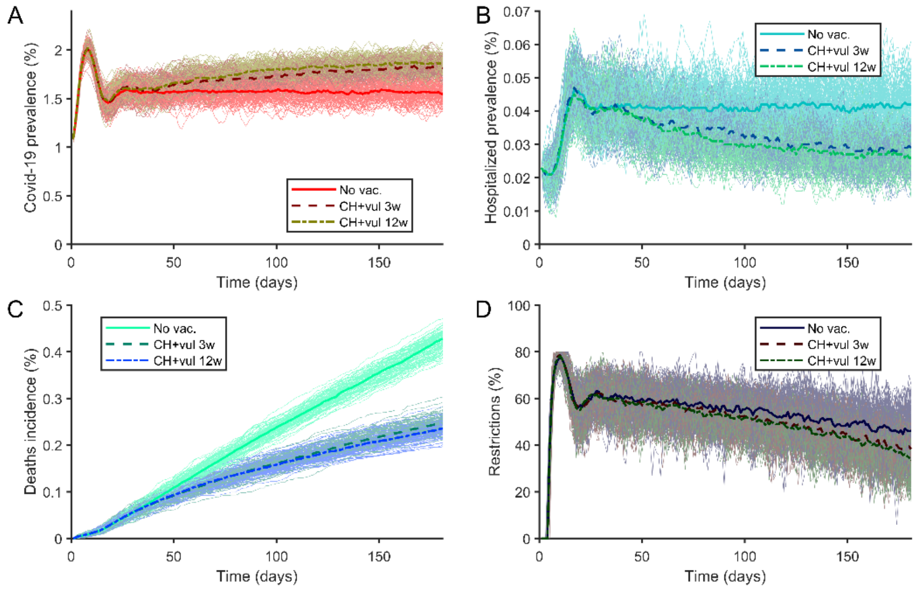

Figure 88. (A) Prevalence of covid-19 cases. (B) Prevalence of hospitalized covid-19 patients. (C) Covid-19 deaths. (D) Restrictions. Solid line for a non-vaccinated population. Dashed line for a population where they are vaccinating first care homes then vulnerable population. Dashed line for doses separation of 3 weeks. Dash-dot line for doses separation of 12 weeks. Figures for AstraZeneca vaccine using Sweden conditions described in table 1.

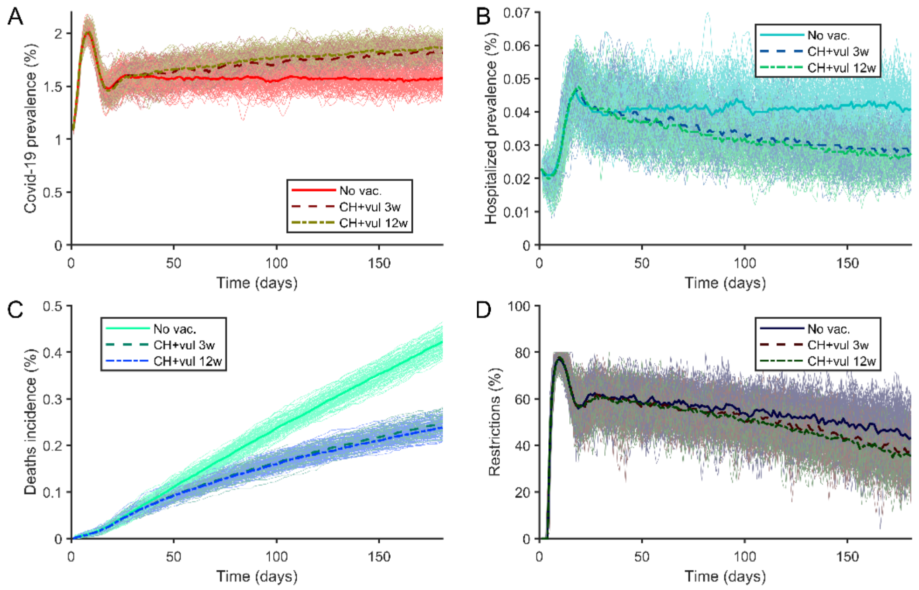

Figure 89. (A) Prevalence of covid-19 cases. (B) Prevalence of hospitalized covid-19 patients. (C) Covid-19 deaths. (D) Restrictions. Solid line for a non-vaccinated population. Dashed line for a population where they are vaccinating first care homes then vulnerable population. Dashed line for doses separation of 3 weeks. Dash-dot line for doses separation of 12 weeks. Figures for Moderna vaccine using Sweden conditions described in table 1.

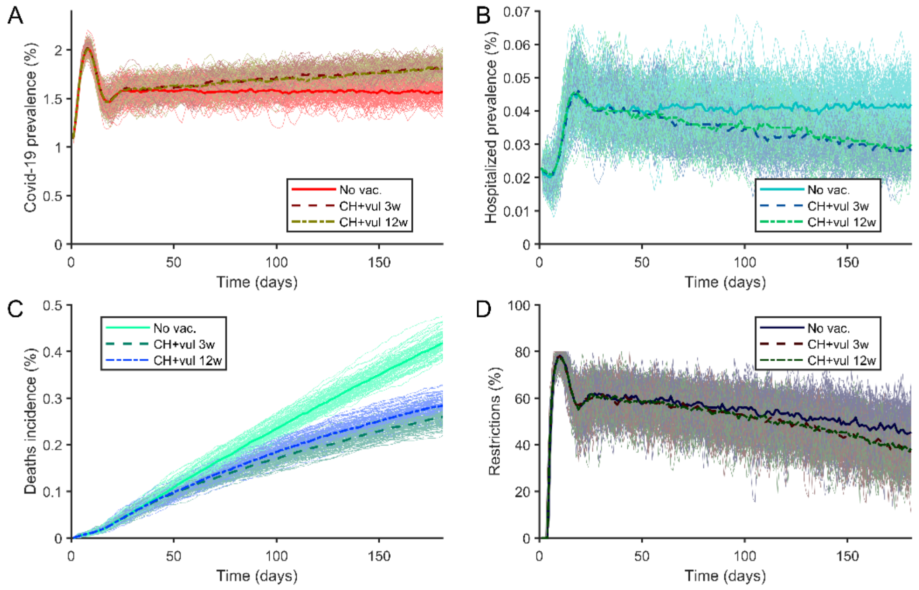

Figure 90. (A) Prevalence of covid-19 cases. (B) Prevalence of hospitalized covid-19 patients. (C) Covid-19 deaths. (D) Restrictions. Solid line for a non-vaccinated population. Dashed line for a population where they are vaccinating first care homes then vulnerable population. Dashed line for doses separation of 3 weeks. Dash-dot line for doses separation of 12 weeks. Figures for Pfizer vaccine using Sweden conditions described in table 1.

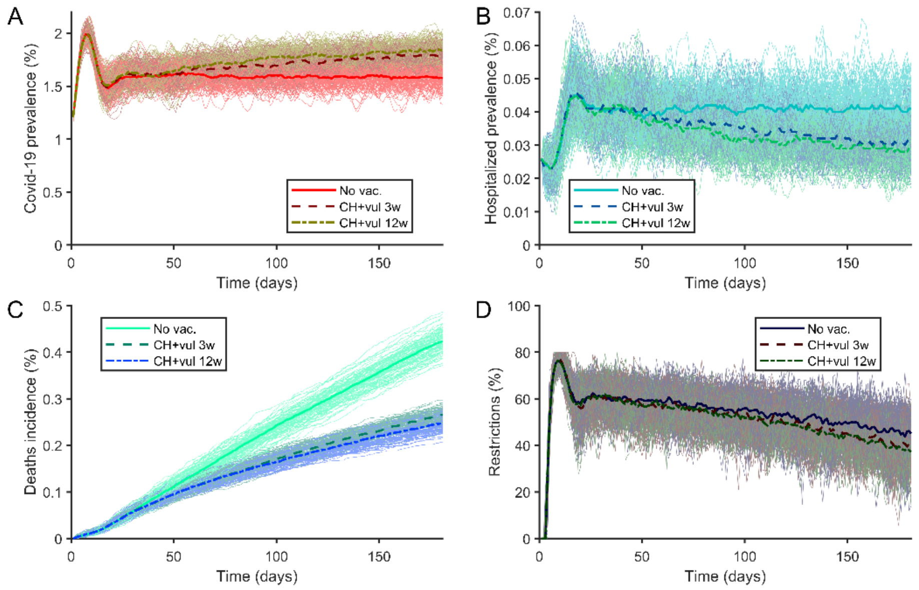

Figure 91. (A) Prevalence of covid-19 cases. (B) Prevalence of hospitalized covid-19 patients. (C) Covid-19 deaths. (D) Restrictions. Solid line for a non-vaccinated population. Dashed line for a population where they are vaccinating first care homes then vulnerable population. Dashed line for doses separation of 3 weeks. Dash-dot line for doses separation of 12 weeks. Figures for AstraZeneca vaccine using Switzerland conditions described in table 1.

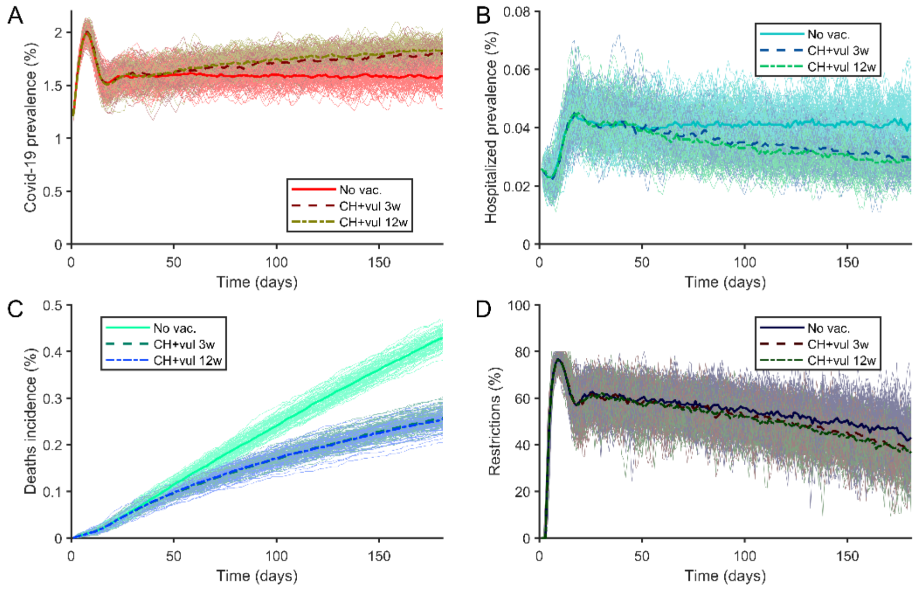

Figure 92. (A) Prevalence of covid-19 cases. (B) Prevalence of hospitalized covid-19 patients. (C) Covid-19 deaths. (D) Restrictions. Solid line for a non-vaccinated population. Dashed line for a population where they are vaccinating first care homes then vulnerable population. Dashed line for doses separation of 3 weeks. Dash-dot line for doses separation of 12 weeks. Figures for Moderna vaccine using Switzerland conditions described in table 1.

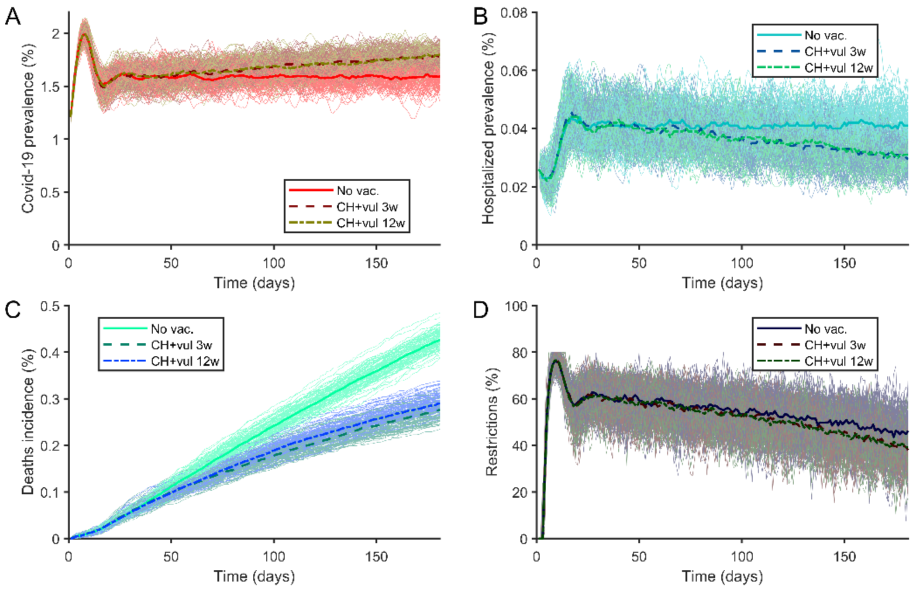

Figure 93. (A) Prevalence of covid-19 cases. (B) Prevalence of hospitalized covid-19 patients. (C) Covid-19 deaths. (D) Restrictions. Solid line for a non-vaccinated population. Dashed line for a population where they are vaccinating first care homes then vulnerable population. Dashed line for doses separation of 3 weeks. Dash-dot line for doses separation of 12 weeks. Figures for Pfizer vaccine using Switzerland conditions described in table 1.

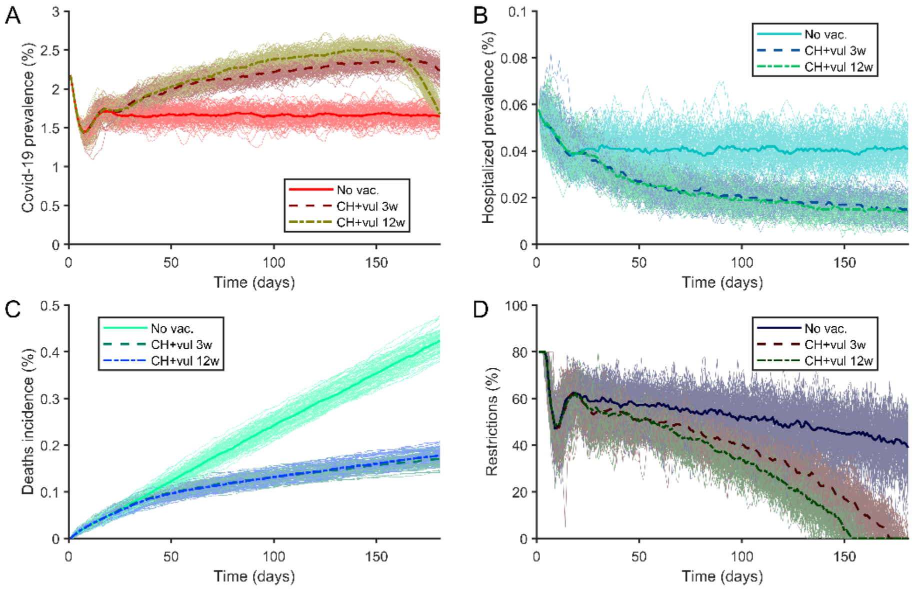

Figure 94. (A) Prevalence of covid-19 cases. (B) Prevalence of hospitalized covid-19 patients. (C) Covid-19 deaths. (D) Restrictions. Solid line for a non-vaccinated population. Dashed line for a population where they are vaccinating first care homes then vulnerable population. Dashed line for doses separation of 3 weeks. Dash-dot line for doses separation of 12 weeks. Figures for AstraZeneca vaccine using United Kingdom conditions described in table 1.

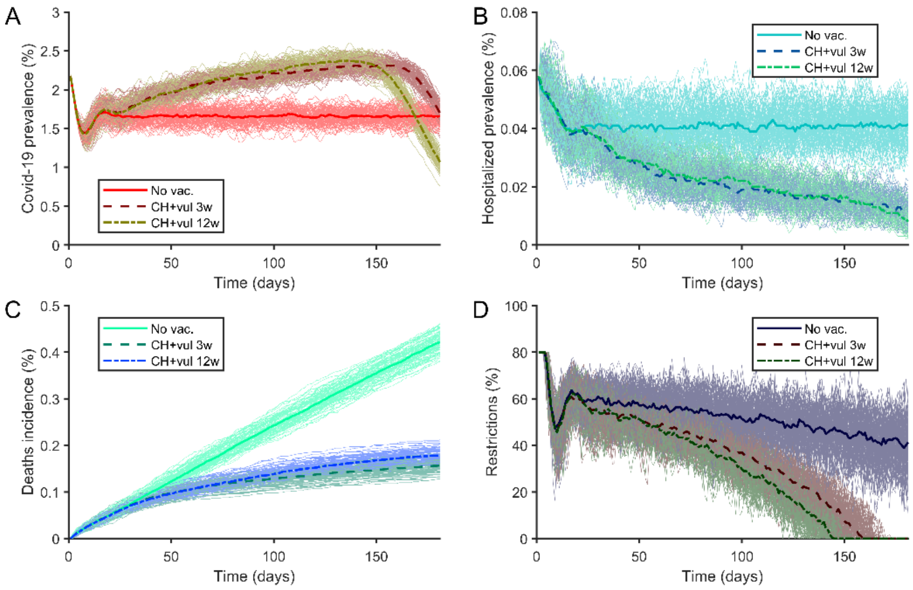

Figure 95. (A) Prevalence of covid-19 cases. (B) Prevalence of hospitalized covid-19 patients. (C) Covid-19 deaths. (D) Restrictions. Solid line for a non-vaccinated population. Dashed line for a population where they are vaccinating first care homes then vulnerable population. Dashed line for doses separation of 3 weeks. Dash-dot line for doses separation of 12 weeks. Figures for Moderna vaccine using United Kingdom conditions described in table 1.

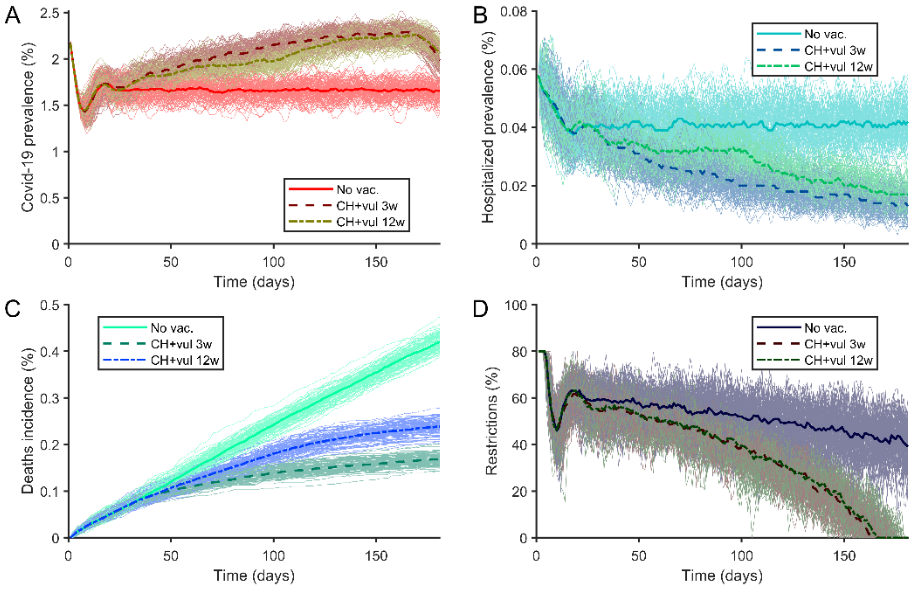

Figure 96. (A) Prevalence of covid-19 cases. (B) Prevalence of hospitalized covid-19 patients. (C) Covid-19 deaths. (D) Restrictions. Solid line for a non-vaccinated population. Dashed line for a population where they are vaccinating first care homes then vulnerable population. Dashed line for doses separation of 3 weeks. Dash-dot line for doses separation of 12 weeks. Figures for Pfizer vaccine using United Kingdom conditions described in table 1.

References:

1. Eurostat [Internet]. [cited 2021 Feb 18]. Available from: https://appsso.eurostat.ec.europa.eu/nui/submitViewTableAction.do

2. Coronavirus Pandemic Data Explorer [Internet]. [cited 2021 Feb 10]. Available from: https://ourworldindata.org/coronavirus-data-explorer?zoomToSelection=true&time=2020-03-01..latest&country=IND~USA~GBR~CAN~DEU~FRA&region=World&vaccinationsMetric=true&interval=smoothed&perCapita=true&smoothing=7&pickerMetric=total_cases&pickerSort=desc

3. Scheil-Adlung X, Others. Long-term care protection for older persons: A review of coverage deficits in 46 countries. ILO; 2015.

4. Nursing and elderly home beds per 100 000 [Internet]. [cited 2021 Feb 10]. Available from: https://gateway.euro.who.int/en/indicators/hfa_490-5100-nursing-and-elderly-home-beds-per-100-000/

5. OECD. Long-Term Care Resources and Utilisation : Long-term care recipients [Internet]. [cited 2021 Feb 10]. Available from: https://stats.oecd.org/index.aspx?queryid=30143
