# Supplementary material for: The impact of prioritisation and dosing intervals on the effects of COVID-19 vaccination in Europe: an agent-based cohort model
Source: Sci Rep. 2021 Sep 22;11:18812. doi: 10.1038/s41598-021-98216-0 (PMC8458447; doi:10.1038/s41598-021-98216-0)
Supplement: Supplementary file 3 — Supplementary Information 3. [file 41598_2021_98216_MOESM3_ESM.pdf]

The impact of prioritisation and dosing intervals on the effects of COVID-19 vaccination in Europe: an agent-based cohort model

Martí Català, Xintong Li, Clara Prats*, Daniel Prieto-Alhambra

*clara.prats@upc.edu

SUPPLEMENTARY MATERIAL: Sensitivity analysis

Summary

1.Methods 1
2.Exploration of individual parameters 2
3.Partial correlation between parameters and different outcomes 14
4.Partial correlation between parameters and different compartments and restrictions 25

1.Methods

Two sensitivity analyses were performed with different methodologies.

First, we designed a one-at-a-time parameter sensitivity analysis. It consisted of performing 20 simulations for each parameter (23), exploring 20 equispaced points inside parameter ranges shown in appendix 1 (model description) table 2. Other parameters were fixed to their default value. At section 2 (exploration of individual parameters) relative changes in deaths, hospitalizations, infected, deaths in care homes, incidence in carehomes and restrictions AUC for each explored value are shown (figures 2.1-2.23).

Then, we performed a full sensitivity analysis by performing 1000 simulations in the parameter space. Latin Hypercube Sample [CITA] was used to choose the appropriate random points. Two conditions were imposed: (1) probability of a certain individual to be hospitalized or dead if being symptomatic must be between 0 and 1, which entails a correlation between f_S, f_H, f_D and f_R; (2) effectivity of vaccine in severe individuals must be equal or higher than that in symptomatic, which must be equal or higher than that in asymptomatic. This entails a correlation between $D_{2,asy}$, $D_{2,sym}$ and $D_{2,sev}$.

At section 3 (partial correlation between parameters and different outcome) partial-rank correlation [CITA] between the 23 explored parameters and 13 outcomes (total amount of deaths, total amount of hospitalized, maximum number of hospitalizations, cumulative incidence, day of the peak in the hospital, last day of restrictions, day of the peak of prevalence, cumulative incidence in care homes, fraction of infected workers, fraction of infected residents, day of the prevalence peak in care homes, deaths in care homes, restrictions AUC) is seen in figures 3.1-3.13. In figures 3.14-3.20 partial-rank correlation over time is seen between parameters and different compartment values and restrictions. Parameters that their maximum absolute value of partial rank correlation coefficient is smaller than 0.2 are not shown.

At Section 4 (partial correlation between parameters and different compartments and restrictions) figures 4.1-4.23 partial-rank correlations between each parameter and different compartments and restrictions over time are shown.

2.Exploration of individual parameters

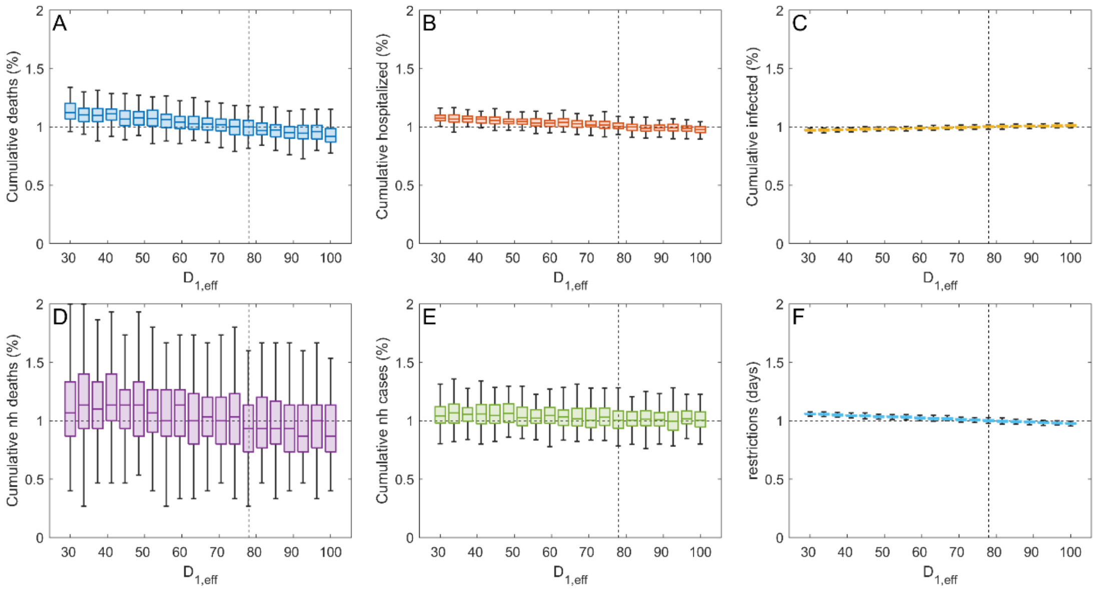

Figure 2.1. Exploration of parameter: efficacy of first dose. (A) Cumulative deaths change respect to baseline values. (B) Cumulative hospitalized change respect to baseline values. (C) Cumulative infected change respect to baseline values. (D) Cumulative care homes deaths change respect to baseline values. (E) Cumulative care homes cases change respect to baseline values. (F) Cumulative restrictions change respect to baseline values.

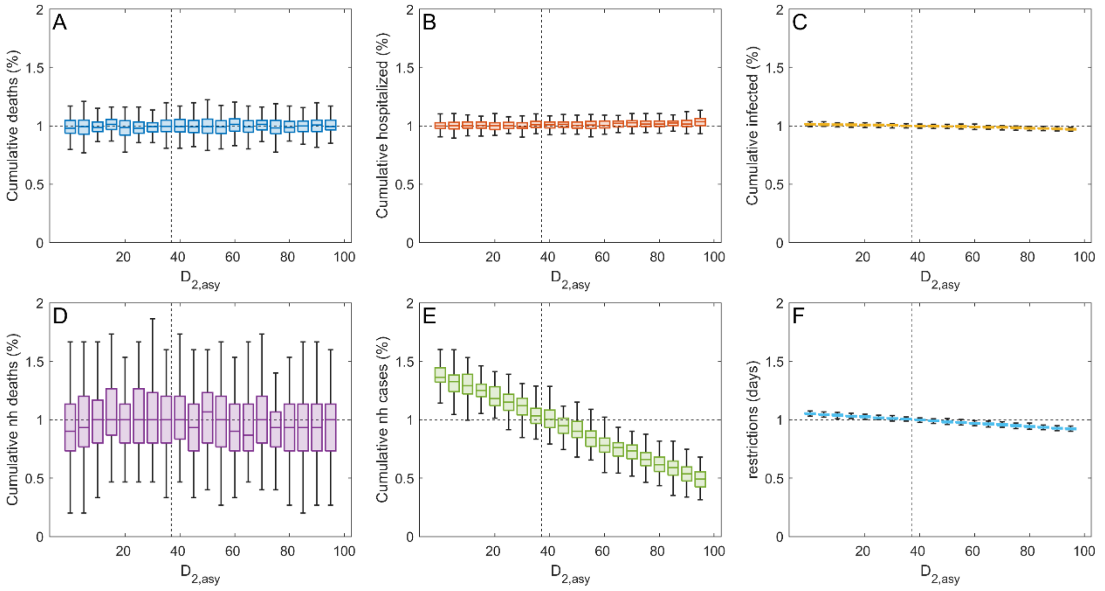

Figure 2.2. Exploration of parameter: efficacy of second dose on asymptomatic cases. (A) Cumulative deaths change respect to baseline values. (B) Cumulative hospitalized change respect to baseline values. (C) Cumulative infected change respect to baseline values. (D) Cumulative care homes deaths change respect to baseline values. (E) Cumulative care homes cases change respect to baseline values. (F) Cumulative restrictions change respect to baseline values.

2.Exploration of individual parameters

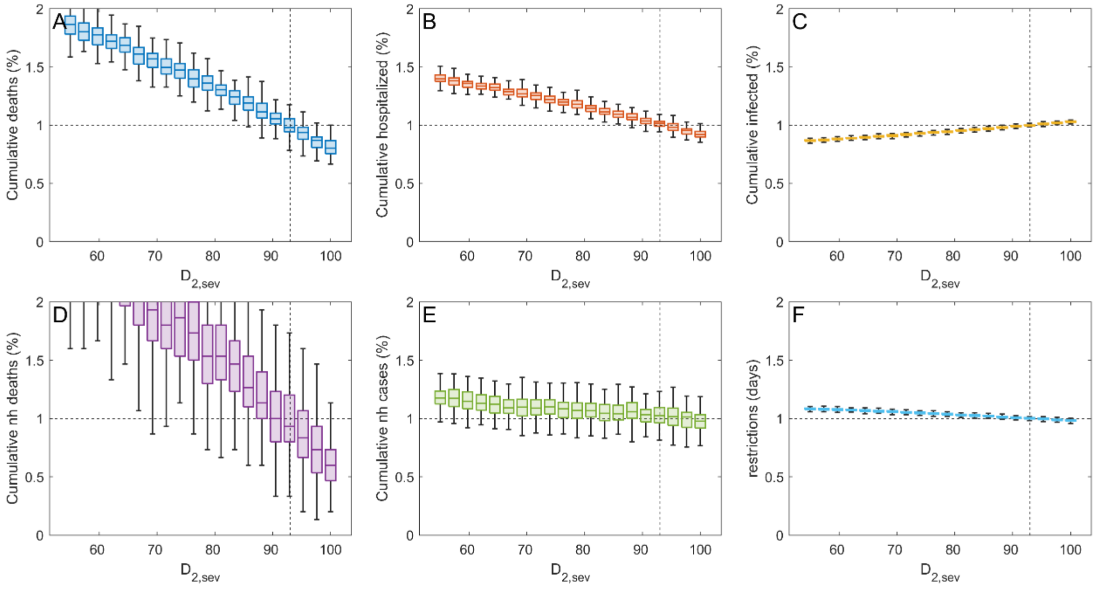

Figure 2.3. Exploration of parameter: efficacy of second dose on symptomatic cases. (A) Cumulative deaths change respect to baseline values. (B) Cumulative hospitalized change respect to baseline values. (C) Cumulative infected change respect to baseline values. (D) Cumulative care homes deaths change respect to baseline values. (E) Cumulative care homes cases change respect to baseline values. (F) Cumulative restrictions change respect to baseline values.

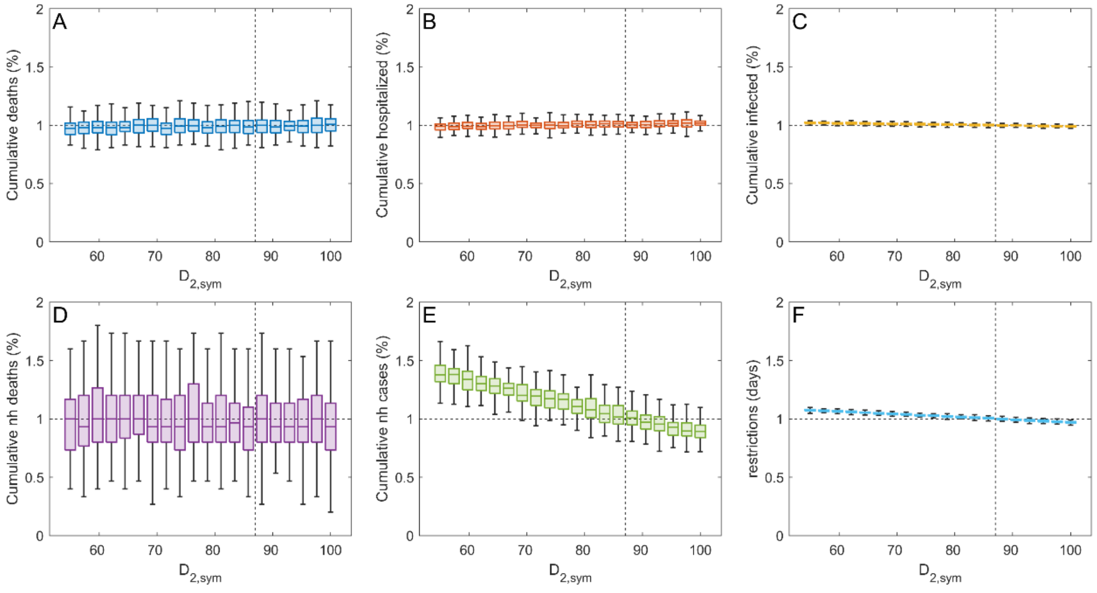

Figure 2.4. Exploration of parameter: efficacy of second dose on symptomatic cases. (A) Cumulative deaths change respect to baseline values. (B) Cumulative hospitalized change respect to baseline values. (C) Cumulative infected change respect to baseline values. (D) Cumulative care homes deaths change respect to baseline values. (E) Cumulative care homes cases change respect to baseline values. (F) Cumulative restrictions change respect to baseline values.

2.Exploration of individual parameters

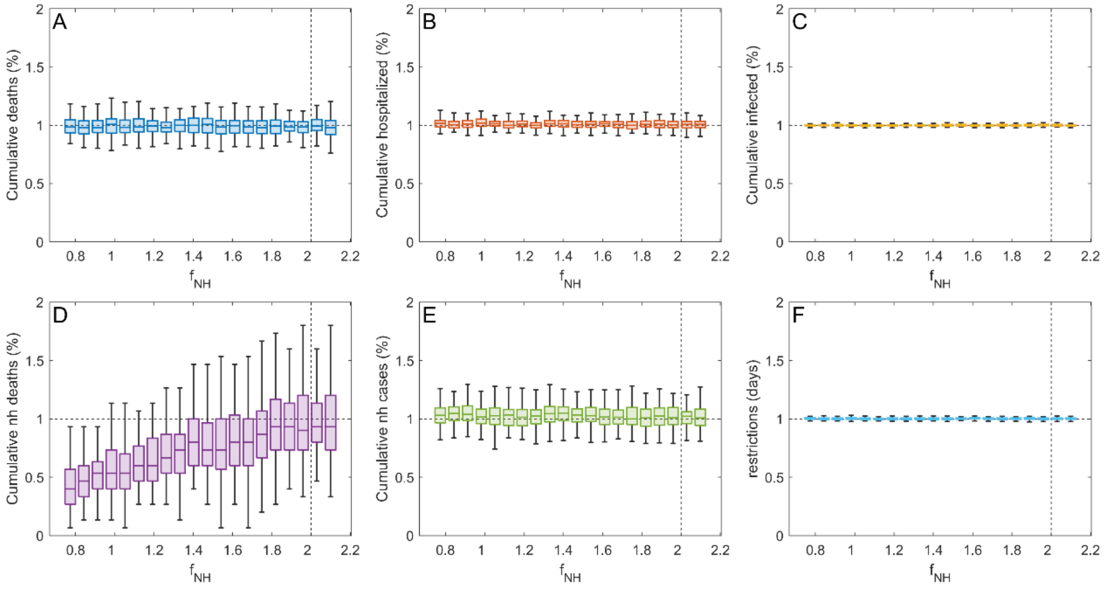

Figure 2.5. Exploration of parameter: factor that increases probability to death in care homes (not changing total probability to dead). (A) Cumulative deaths change respect to baseline values. (B) Cumulative hospitalized change respect to baseline values. (C) Cumulative infected change respect to baseline values. (D) Cumulative care homes deaths change respect to baseline values. (E) Cumulative care homes cases change respect to baseline values. (F) Cumulative restrictions change respect to baseline values.

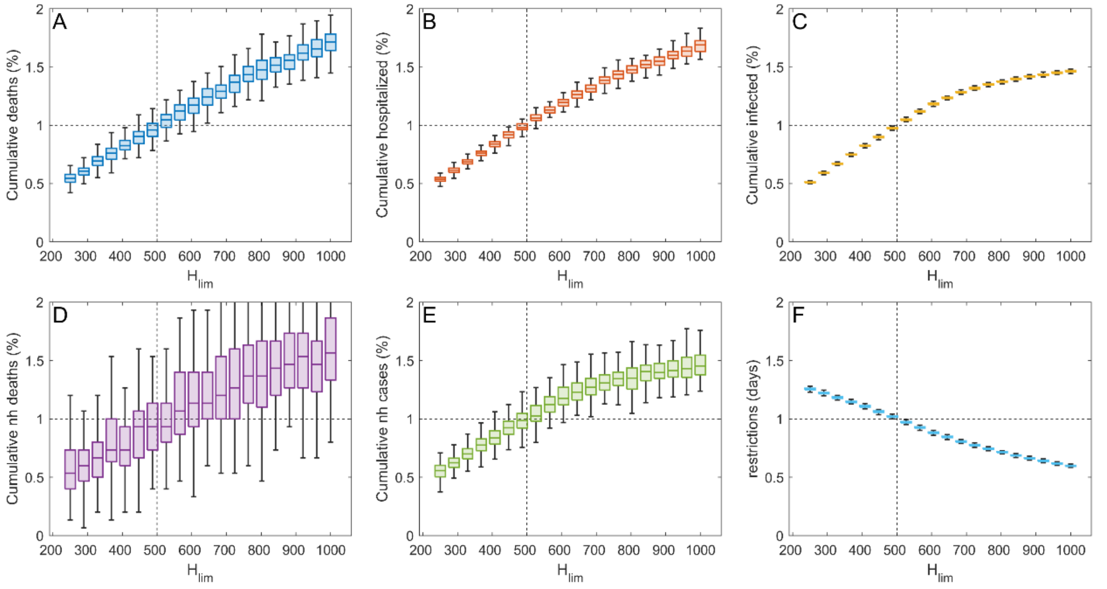

Figure 2.6. Exploration of parameter: number of limiting hospitalized. (A) Cumulative deaths change respect to baseline values. (B) Cumulative hospitalized change respect to baseline values. (C) Cumulative infected change respect to baseline values. (D) Cumulative care homes deaths change respect to baseline values. (E) Cumulative care homes cases change respect to baseline values. (F) Cumulative restrictions change respect to baseline values.

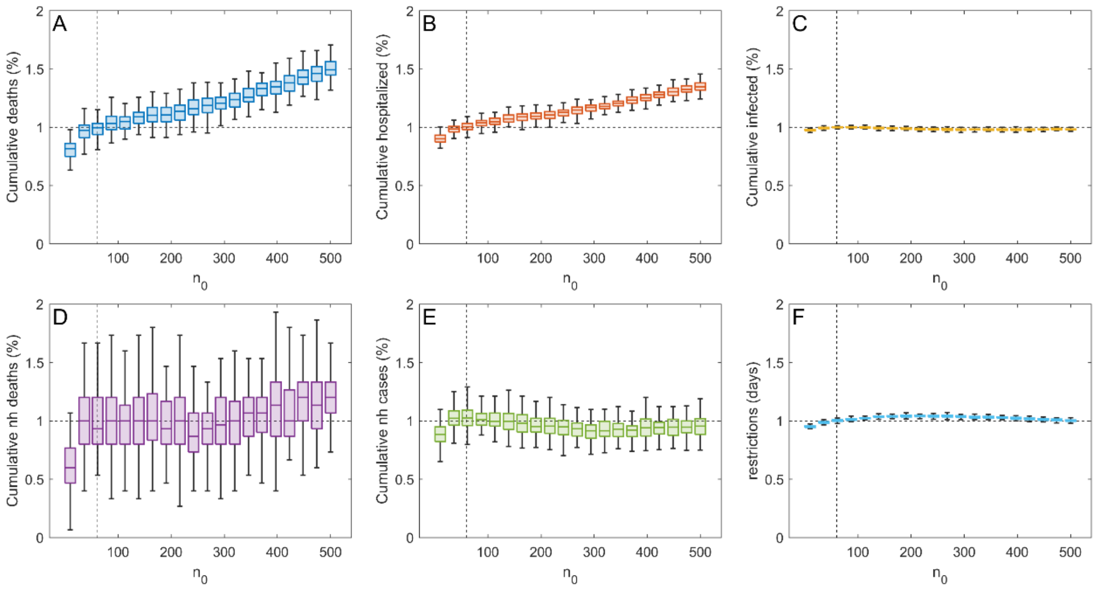

Figure 2.7. Exploration of parameter: initial number of cases per day. (A) Cumulative deaths change respect to baseline values. (B) Cumulative hospitalized change respect to baseline values. (C) Cumulative infected change respect to baseline values. (D) Cumulative care homes deaths change respect to baseline values. (E) Cumulative care homes cases change respect to baseline values. (F) Cumulative restrictions change respect to baseline values.

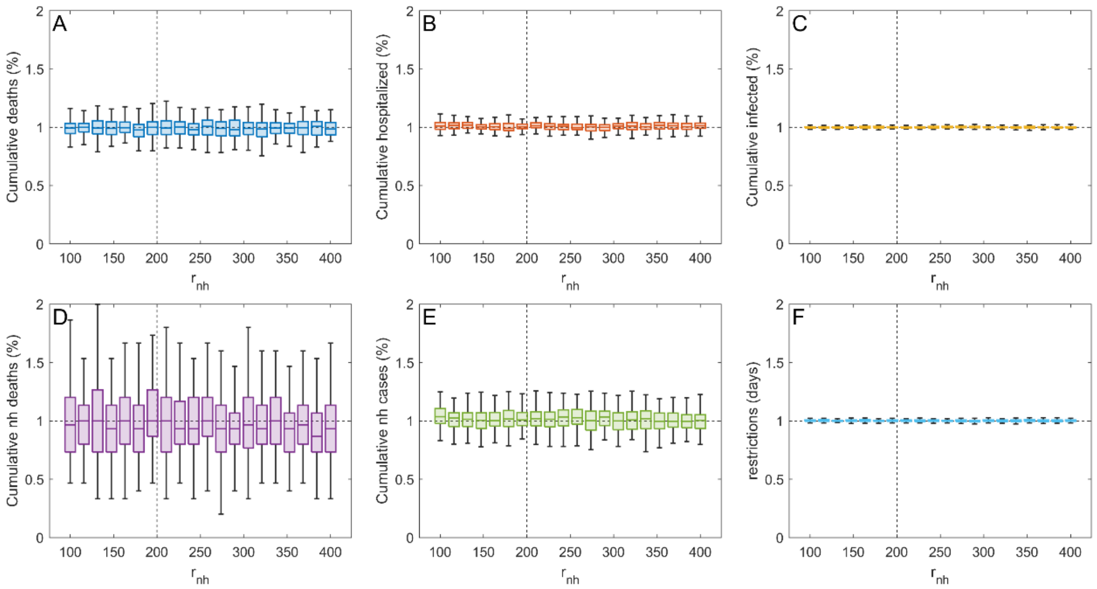

Figure 2.8. Exploration of parameter: care homes rate. (A) Cumulative deaths change respect to baseline values. (B) Cumulative hospitalized change respect to baseline values. (C) Cumulative infected change respect to baseline values. (D) Cumulative care homes deaths change respect to baseline values. (E) Cumulative care homes cases change respect to baseline values. (F) Cumulative restrictions change respect to baseline values.

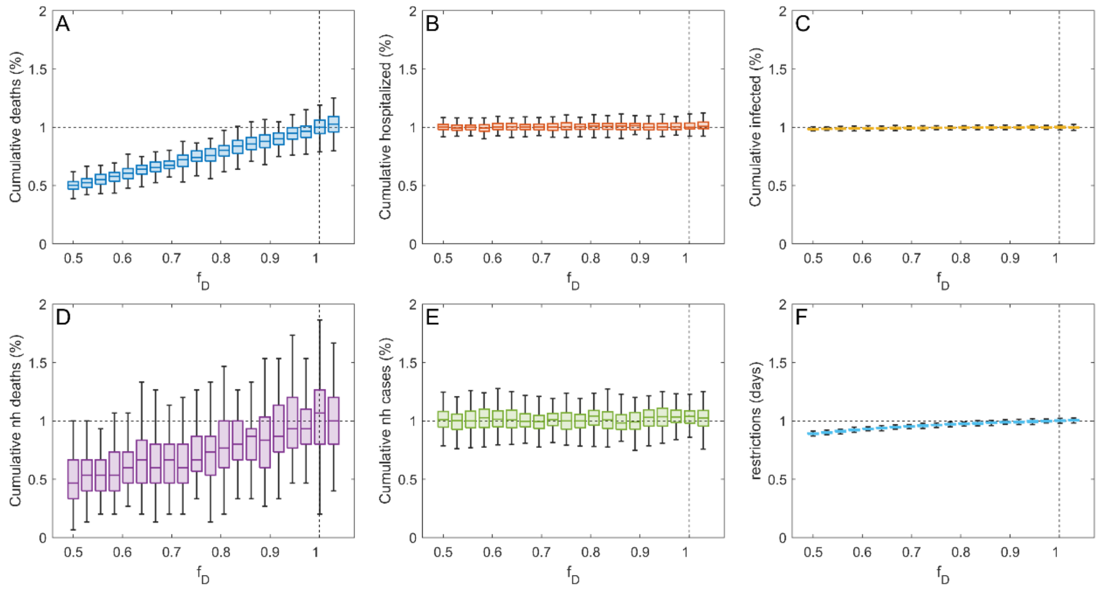

Figure 2.9. Exploration of parameter: increased probability to dead factor. (A) Cumulative deaths change respect to baseline values. (B) Cumulative hospitalized change respect to baseline values. (C) Cumulative infected change respect to baseline values. (D) Cumulative care homes deaths change respect to baseline values. (E) Cumulative care homes cases change respect to baseline values. (F) Cumulative restrictions change respect to baseline values.

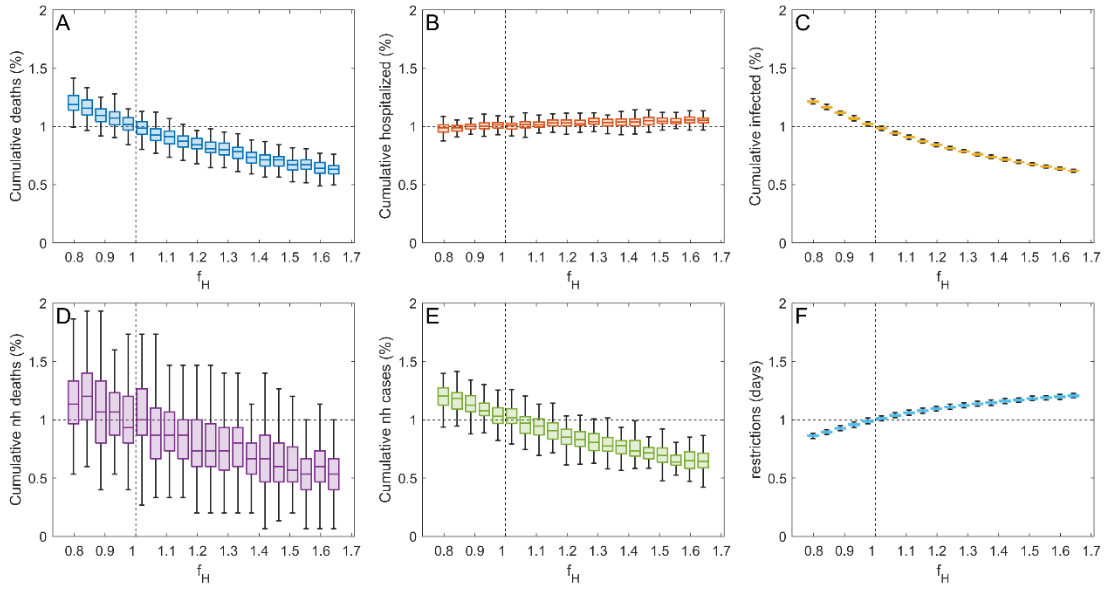

Figure 2.10. Exploration of parameter: increased probability to be hospitalized factor. (A) Cumulative deaths change respect to baseline values. (B) Cumulative hospitalized change respect to baseline values. (C) Cumulative infected change respect to baseline values. (D) Cumulative care homes deaths change respect to baseline values. (E) Cumulative care homes cases change respect to baseline values. (F) Cumulative restrictions change respect to baseline values.

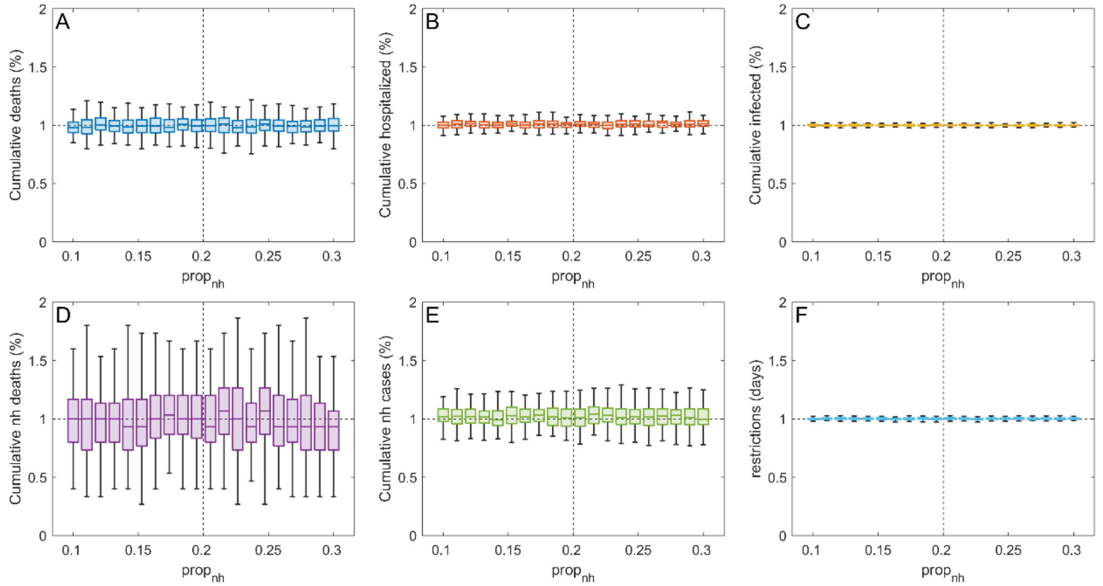

Figure 2.11. Exploration of parameter: proportion of nurses per resident. (A) Cumulative deaths change respect to baseline values. (B) Cumulative hospitalized change respect to baseline values. (C) Cumulative infected change respect to baseline values. (D) Cumulative care homes deaths change respect to baseline values. (E) Cumulative care homes cases change respect to baseline values. (F) Cumulative restrictions change respect to baseline values.

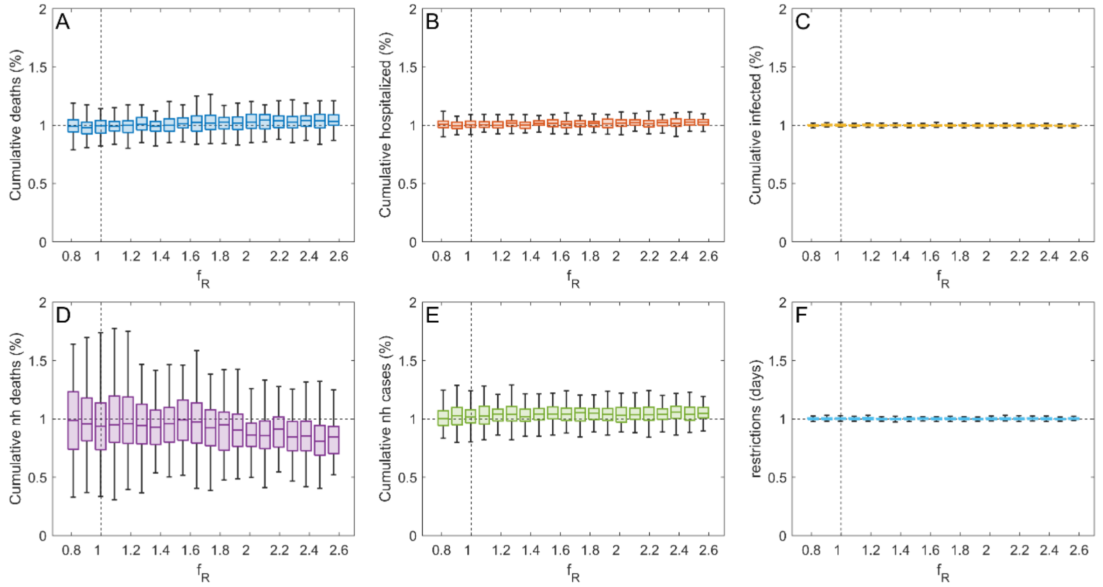

Figure 2.12. Exploration of parameter: increased probability to be a resident in a care home. (A) Cumulative deaths change respect to baseline values. (B) Cumulative hospitalized change respect to baseline values. (C) Cumulative infected change respect to baseline values. (D) Cumulative care homes deaths change respect to baseline values. (E) Cumulative care homes cases change respect to baseline values. (F) Cumulative restrictions change respect to baseline values.

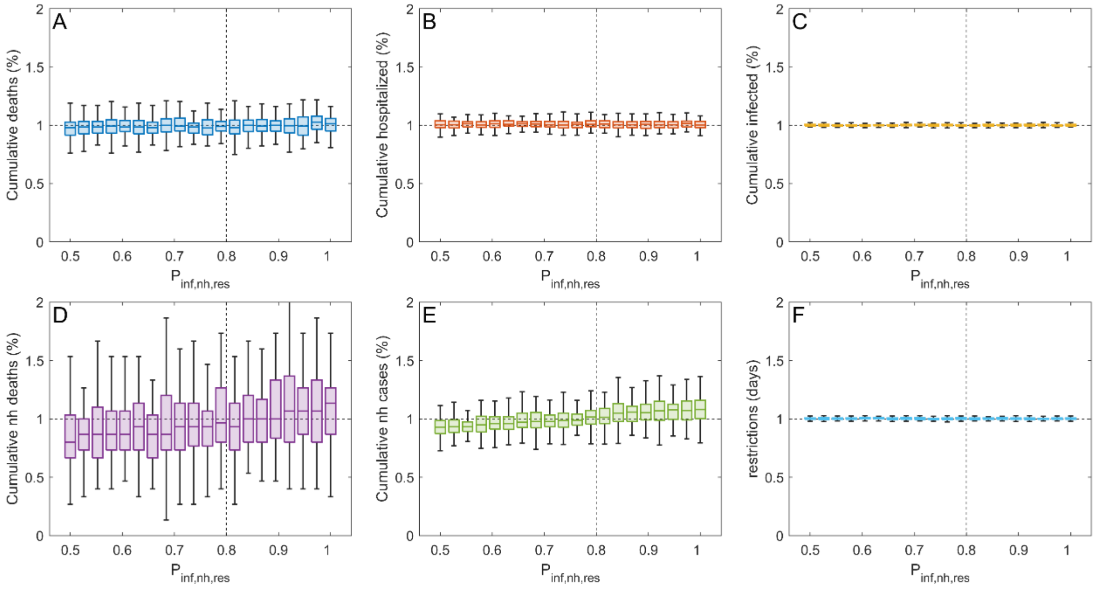

Figure 2.13. Exploration of parameter: probability that residents infect inside the care home. (A) Cumulative deaths change respect to baseline values. (B) Cumulative hospitalized change respect to baseline values. (C) Cumulative infected change respect to baseline values. (D) Cumulative care homes deaths change respect to baseline values. (E) Cumulative care homes cases change respect to baseline values. (F) Cumulative restrictions change respect to baseline values.

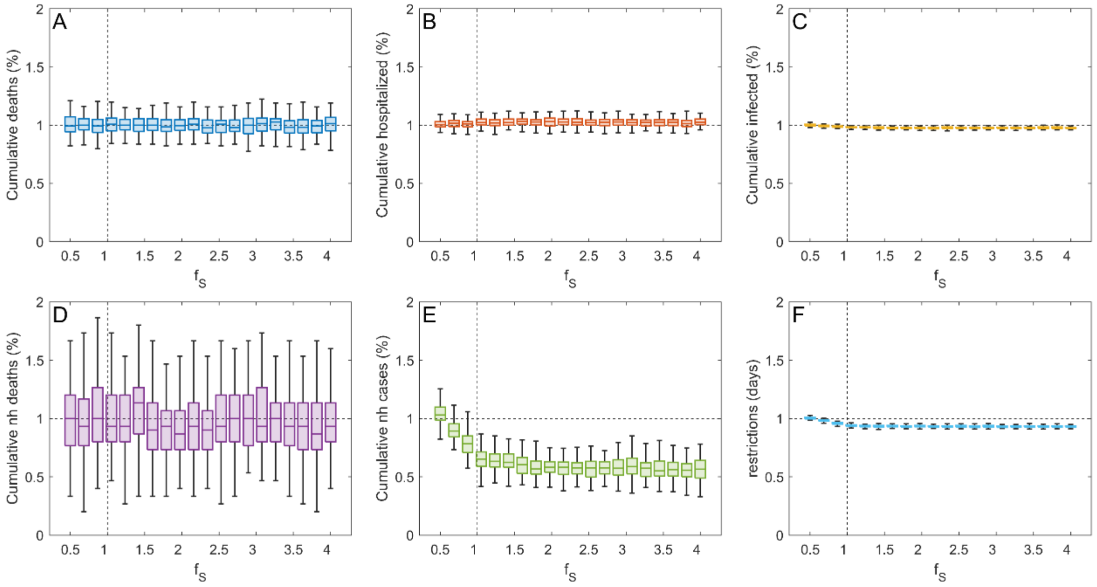

Figure 2.14. Exploration of parameter: increased probability to be symptomatic. (A) Cumulative deaths change respect to baseline values. (B) Cumulative hospitalized change respect to baseline values. (C) Cumulative infected change respect to baseline values. (D) Cumulative care homes deaths change respect to baseline values. (E) Cumulative care homes cases change respect to baseline values. (F) Cumulative restrictions change respect to baseline values.

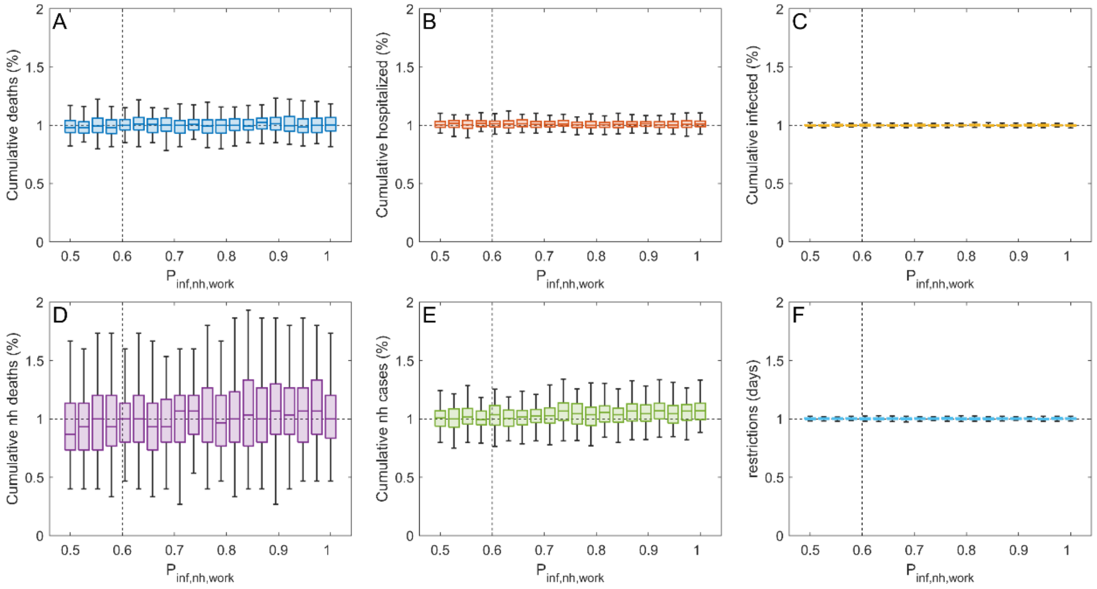

Figure 2.15. Exploration of parameter: probbaility that a nurse infects inside care homes. (A) Cumulative deaths change respect to baseline values. (B) Cumulative hospitalized change respect to baseline values. (C) Cumulative infected change respect to baseline values. (D) Cumulative care homes deaths change respect to baseline values. (E) Cumulative care homes cases change respect to baseline values. (F) Cumulative restrictions change respect to baseline values.

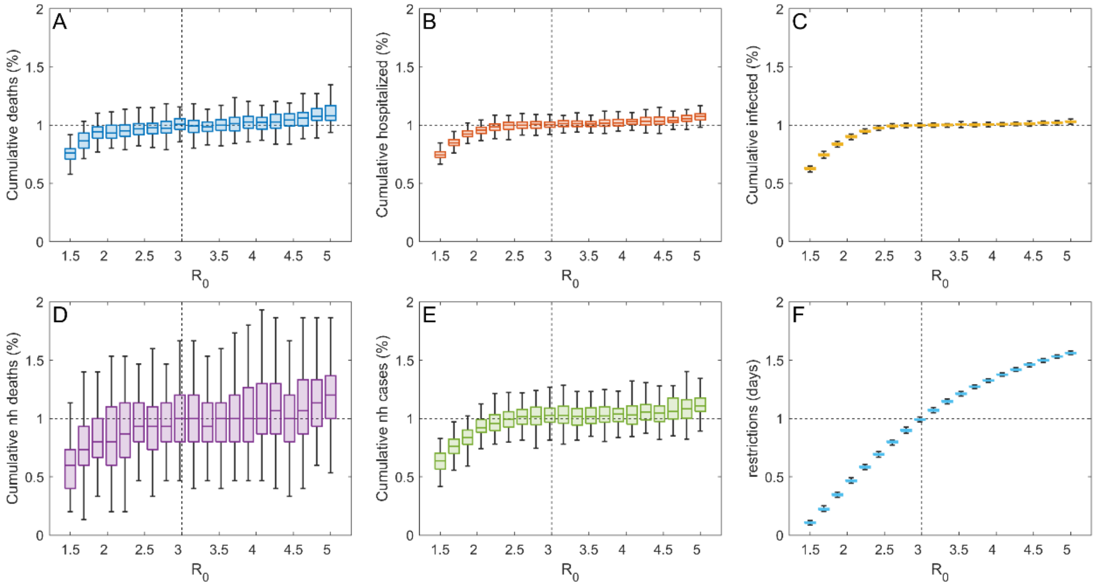

Figure 2.16. Exploration of parameter: reproductive basic number. (A) Cumulative deaths change respect to baseline values. (B) Cumulative hospitalized change respect to baseline values. (C) Cumulative infected change respect to baseline values. (D) Cumulative care homes deaths change respect to baseline values. (E) Cumulative care homes cases change respect to baseline values. (F) Cumulative restrictions change respect to baseline values.

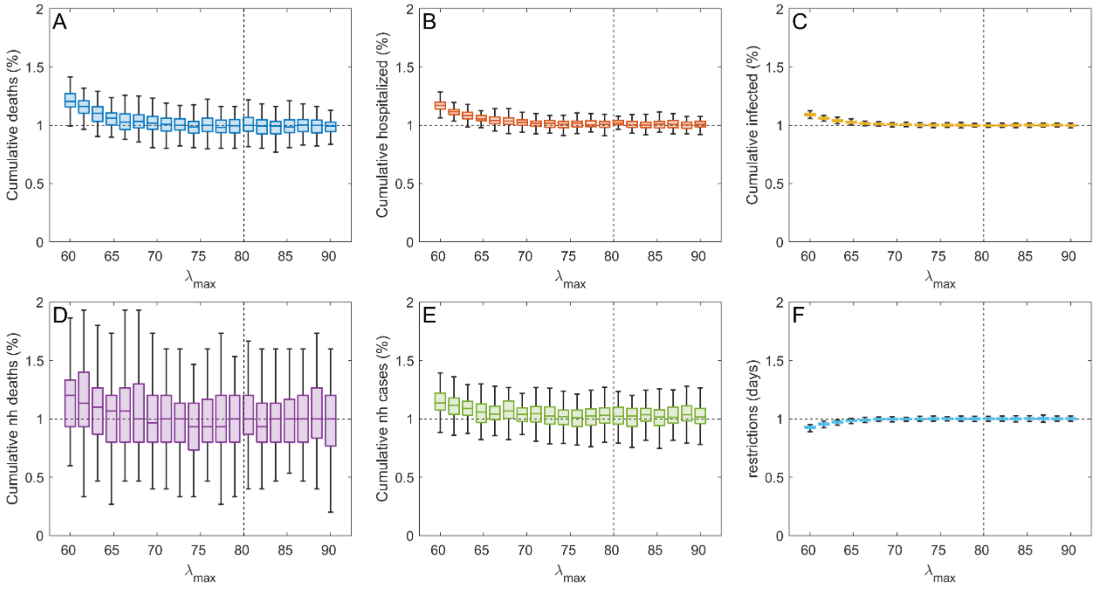

Figure 2.17. Exploration of parameter: maximum restrictions. (A) Cumulative deaths change respect to baseline values. (B) Cumulative hospitalized change respect to baseline values. (C) Cumulative infected change respect to baseline values. (D) Cumulative care homes deaths change respect to baseline values. (E) Cumulative care homes cases change respect to baseline values. (F) Cumulative restrictions change respect to baseline values.

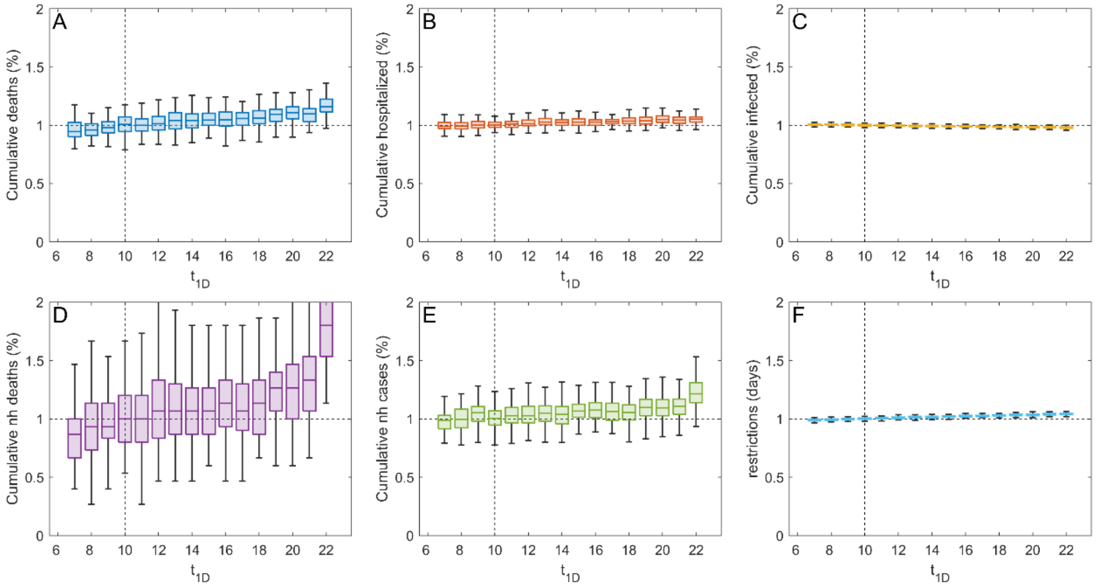

Figure 2.18. Exploration of parameter: time till first dose is effective. (A) Cumulative deaths change respect to baseline values. (B) Cumulative hospitalized change respect to baseline values. (C) Cumulative infected change respect to baseline values. (D) Cumulative care homes deaths change respect to baseline values. (E) Cumulative care homes cases change respect to baseline values. (F) Cumulative restrictions change respect to baseline values.

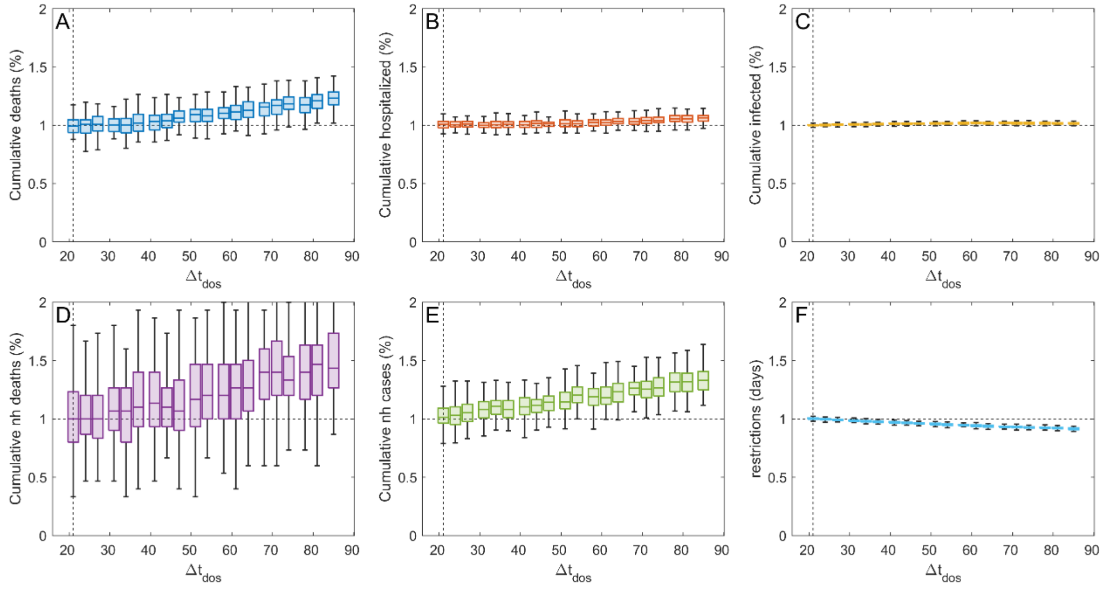

Figure 2.19. Exploration of parameter: time between doses. (A) Cumulative deaths change respect to baseline values. (B) Cumulative hospitalized change respect to baseline values. (C) Cumulative infected change respect to baseline values. (D) Cumulative care homes deaths change respect to baseline values. (E) Cumulative care homes cases change respect to baseline values. (F) Cumulative restrictions change respect to baseline values.

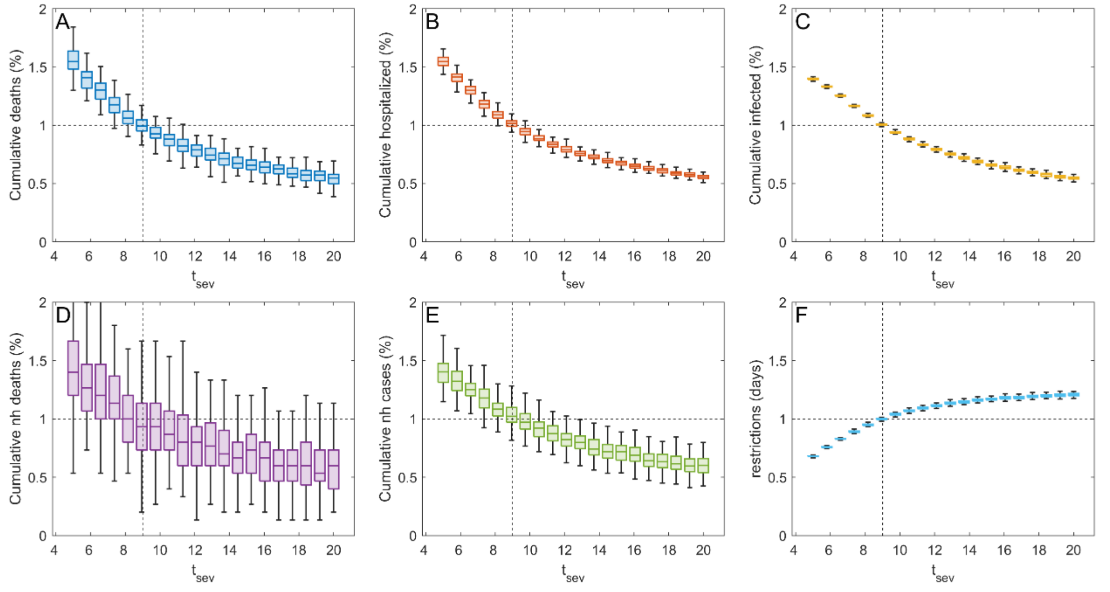

Figure 2.20. Exploration of parameter: disease time. (A) Cumulative deaths change respect to baseline values. (B) Cumulative hospitalized change respect to baseline values. (C) Cumulative infected change respect to baseline values. (D) Cumulative care homes deaths change respect to baseline values. (E) Cumulative care homes cases change respect to baseline values. (F) Cumulative restrictions change respect to baseline values.

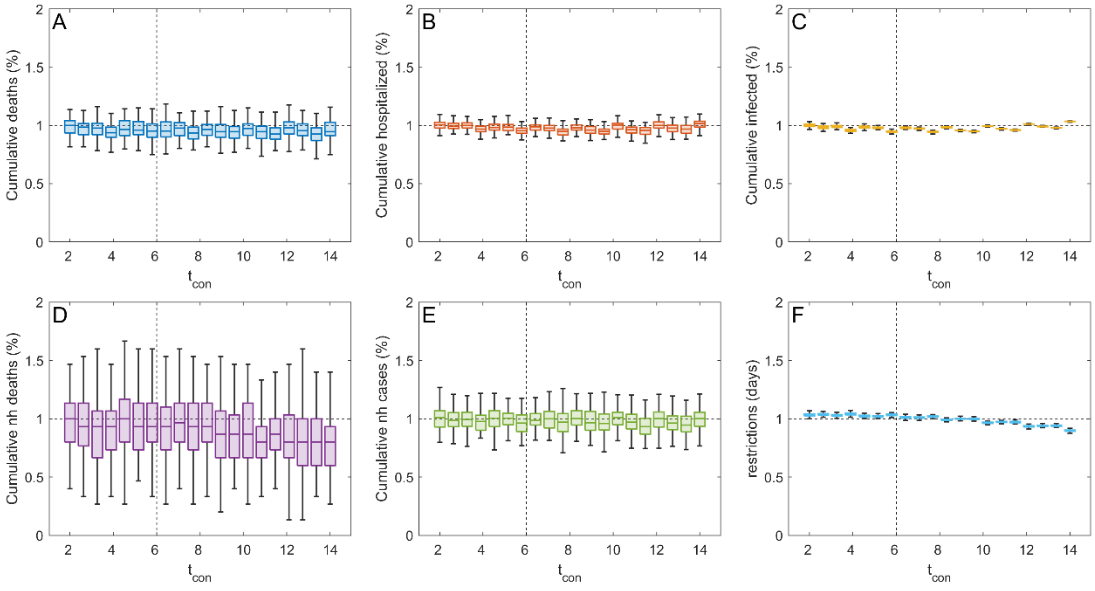

Figure 2.21. Exploration of parameter: contagious time. (A) Cumulative deaths change respect to baseline values. (B) Cumulative hospitalized change respect to baseline values. (C) Cumulative infected change respect to baseline values. (D) Cumulative care homes deaths change respect to baseline values. (E) Cumulative care homes cases change respect to baseline values. (F) Cumulative restrictions change respect to baseline values.

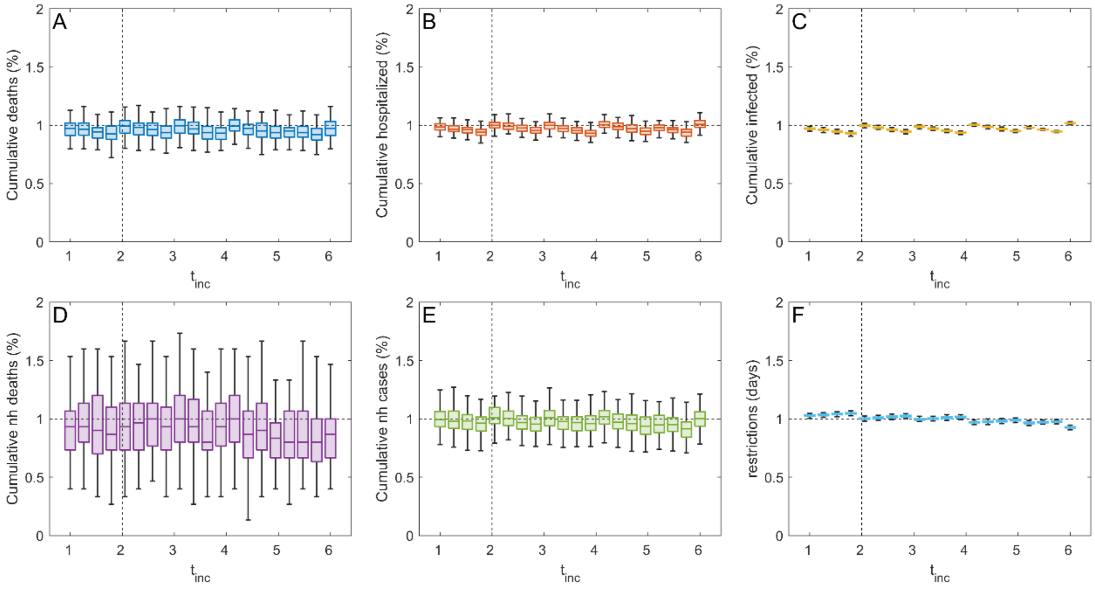

Figure 2.22. Exploration of parameter: pre-infective time. (A) Cumulative deaths change respect to baseline values. (B) Cumulative hospitalized change respect to baseline values. (C) Cumulative infected change respect to baseline values. (D) Cumulative care homes deaths change respect to baseline values. (E) Cumulative care homes cases change respect to baseline values. (F) Cumulative restrictions change respect to baseline values.

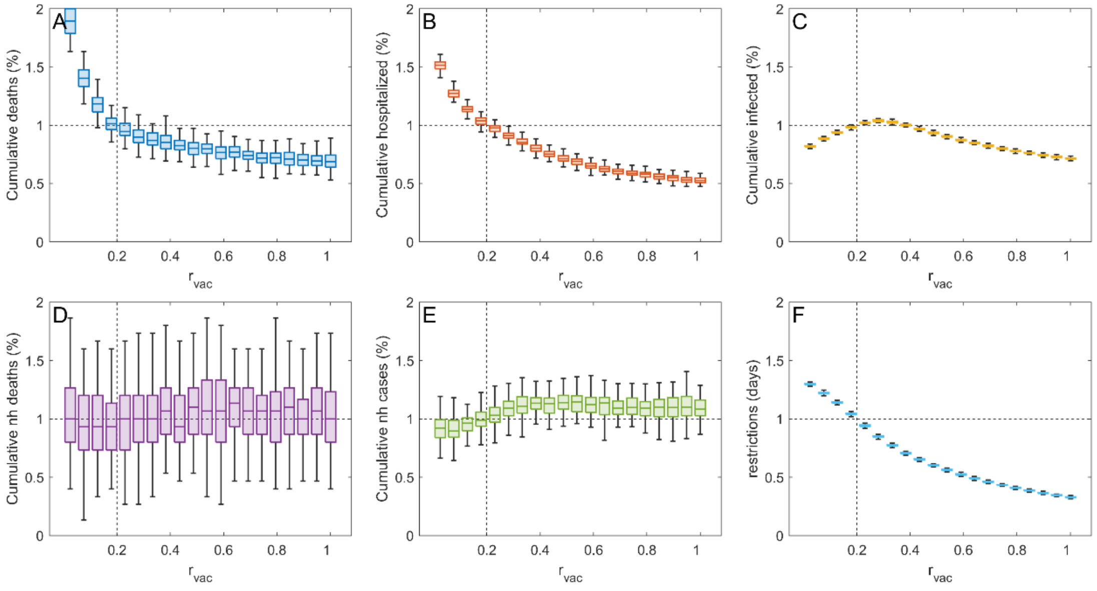

Figure 2.23. Exploration of parameter: vaccination speed. (A) Cumulative deaths change respect to baseline values. (B) Cumulative hospitalized change respect to baseline values. (C) Cumulative infected change respect to baseline values. (D) Cumulative care homes deaths change respect to baseline values. (E) Cumulative care homes cases change respect to baseline values. (F) Cumulative restrictions change respect to baseline values.

3.Partial correlation between parameters and different outcomes

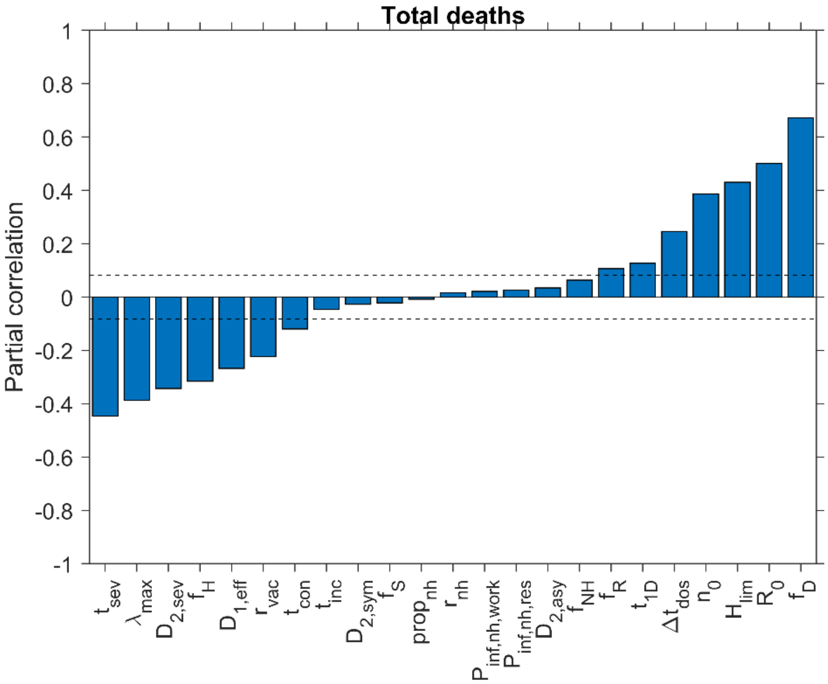

Figure 3.1. Partial correlation between input parameters and total deaths.

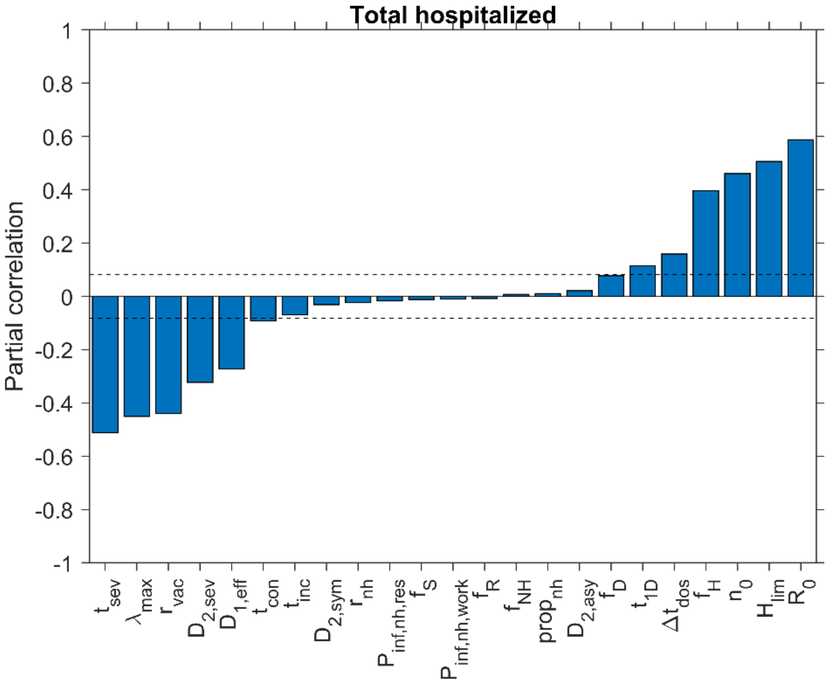

Figure 3.2. Partial correlation between input parameters and total hospitalized.

3.Partial correlation between parameters and different outcomes

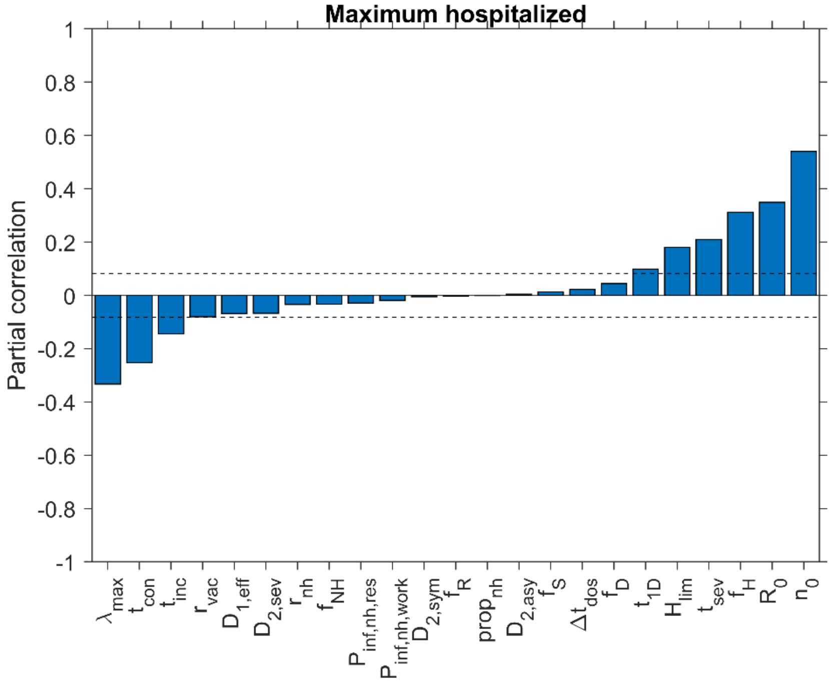

Figure 3.3. Partial correlation between input parameters and peak of hospitalized.

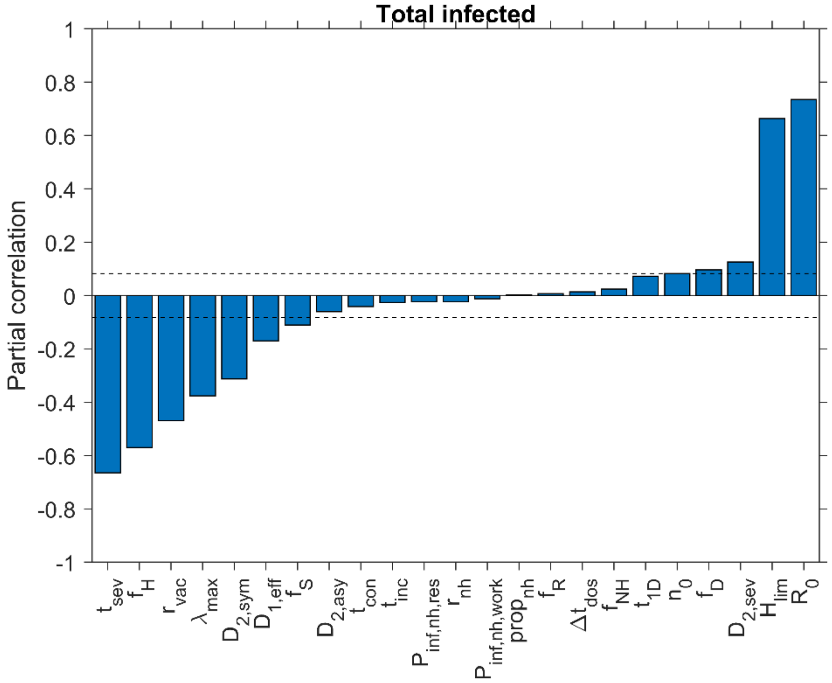

Figure 3.4. Partial correlation between input parameters and total infected.

3.Partial correlation between parameters and different outcomes

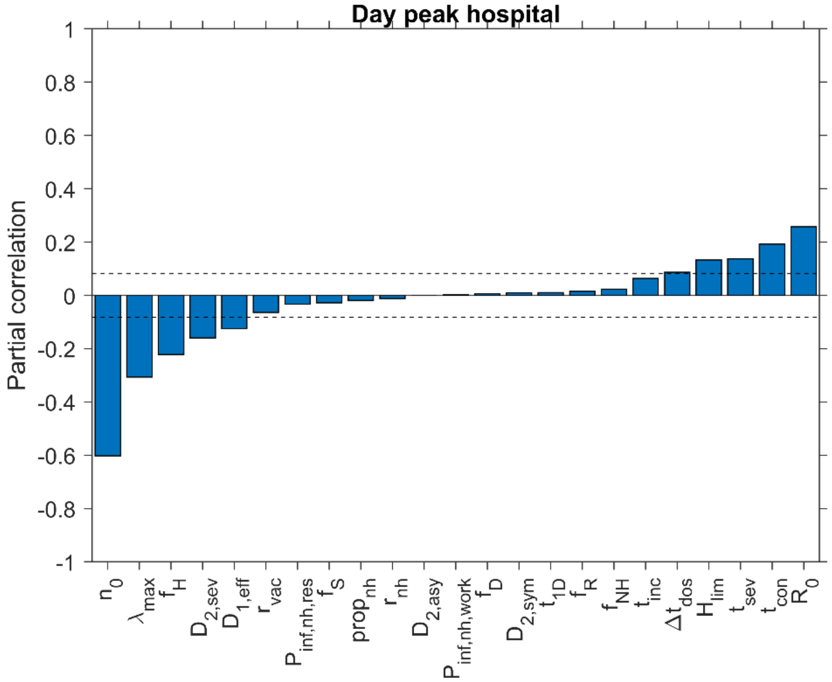

Figure 3.5. Partial correlation between input parameters and day of hospitalized peak.

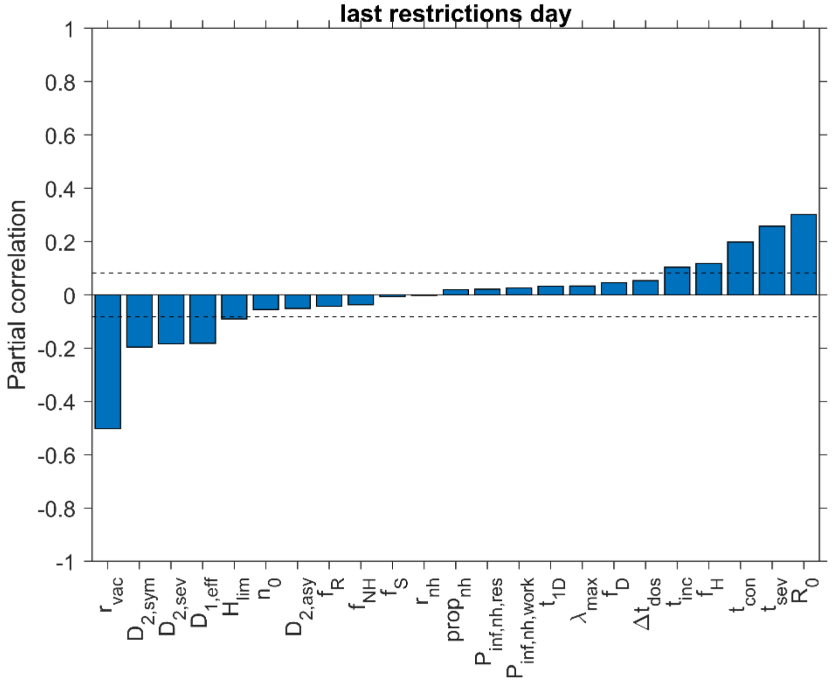

Figure 3.6. Partial correlation between input parameters and last restrictions day.

3.Partial correlation between parameters and different outcomes

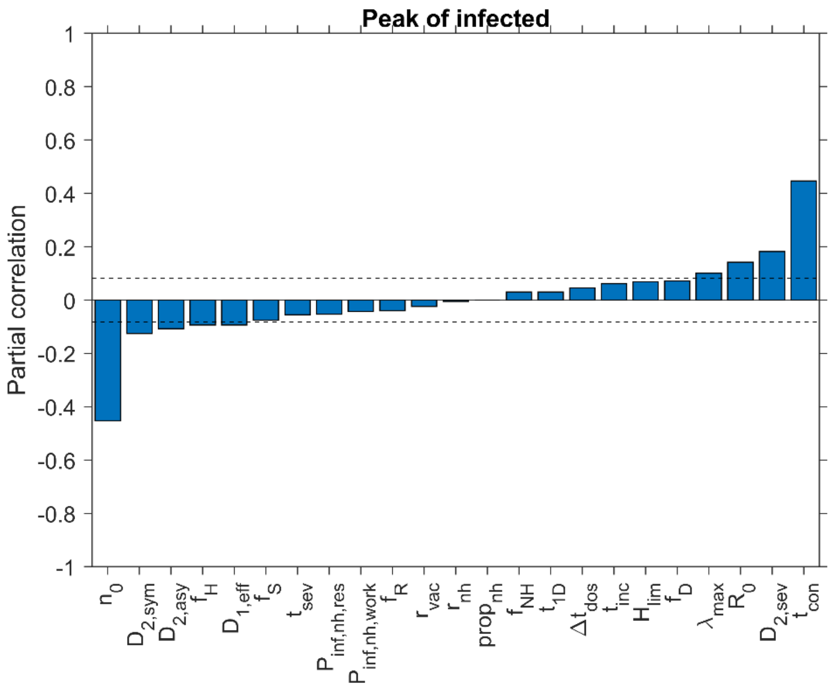

Figure 3.7. Partial correlation between input parameters and peak of infected.

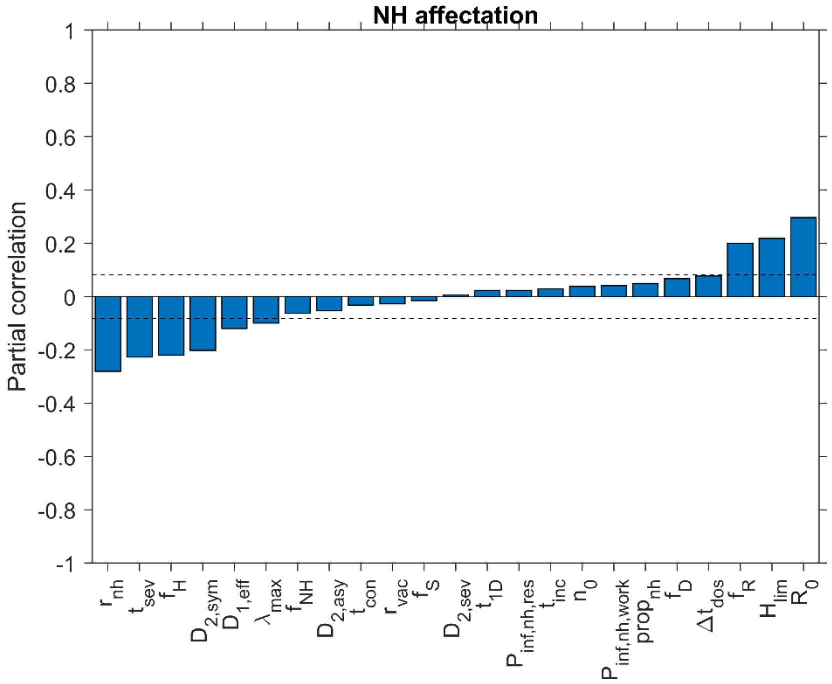

Figure 3.8. Partial correlation between input parameters and incidence in care homes.

3.Partial correlation between parameters and different outcomes

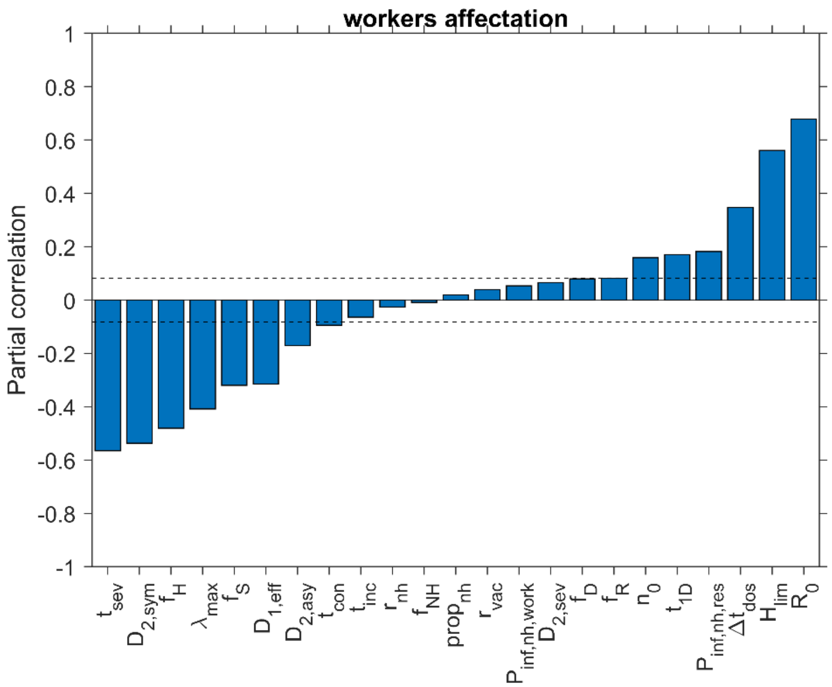

Figure 3.9. Partial correlation between input parameters and incidence on care home workers.

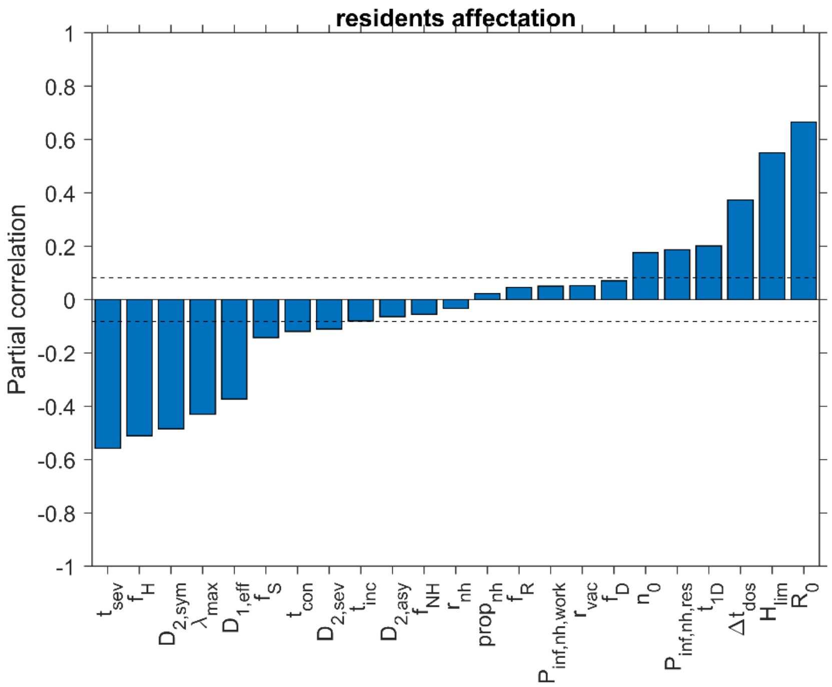

Figure 3.10. Partial correlation between input parameters and incidence in care home residents.

3.Partial correlation between parameters and different outcomes

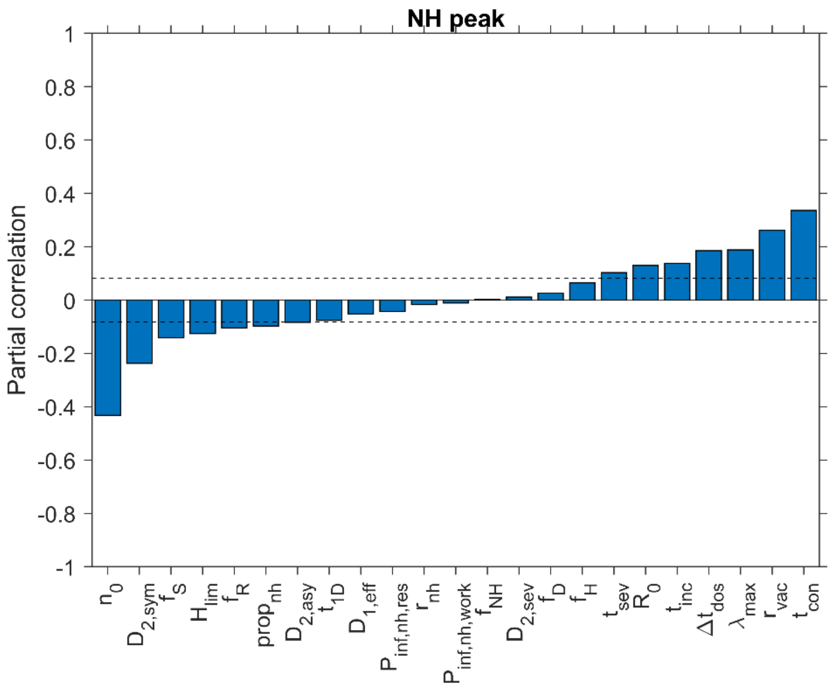

Figure 3.11. Partial correlation between input parameters and incidence peak in care homes.

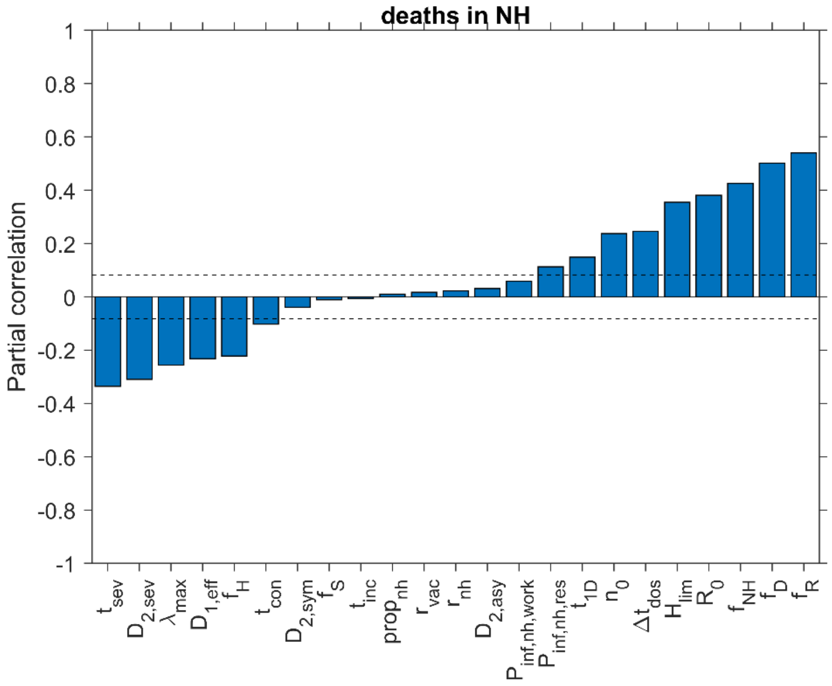

Figure 3.12. Partial correlation between input parameters and deaths in care homes.

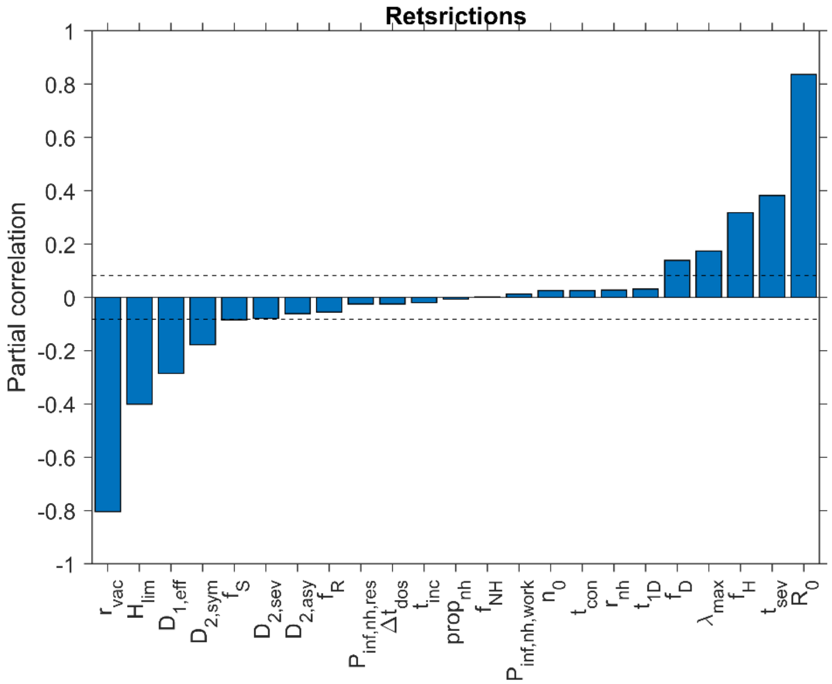

Figure 3.13. Partial correlation between input parameters and cumulative restrictions.

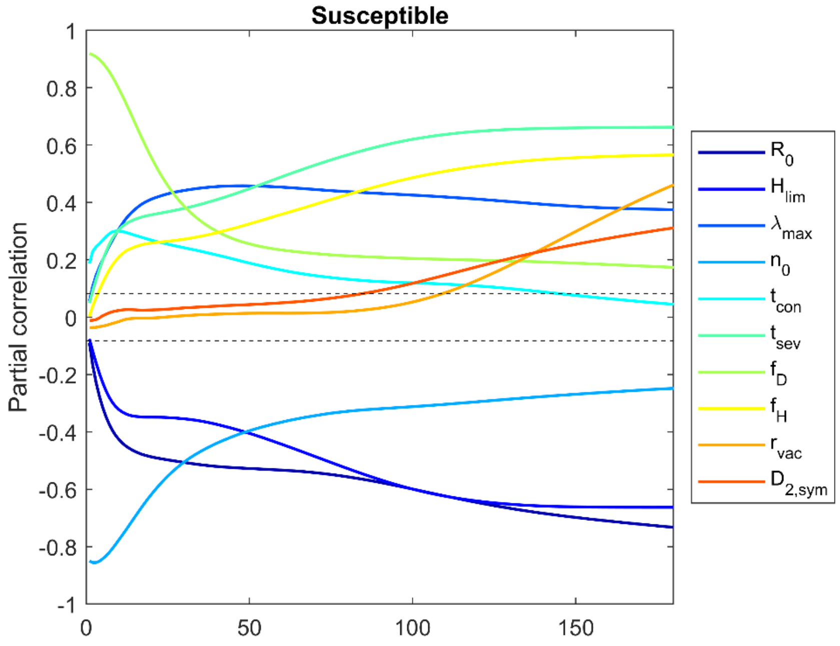

Figure 3.14. Partial correlation between input parameters and susceptible individuals along time.

3.Partial correlation between parameters and different outcomes

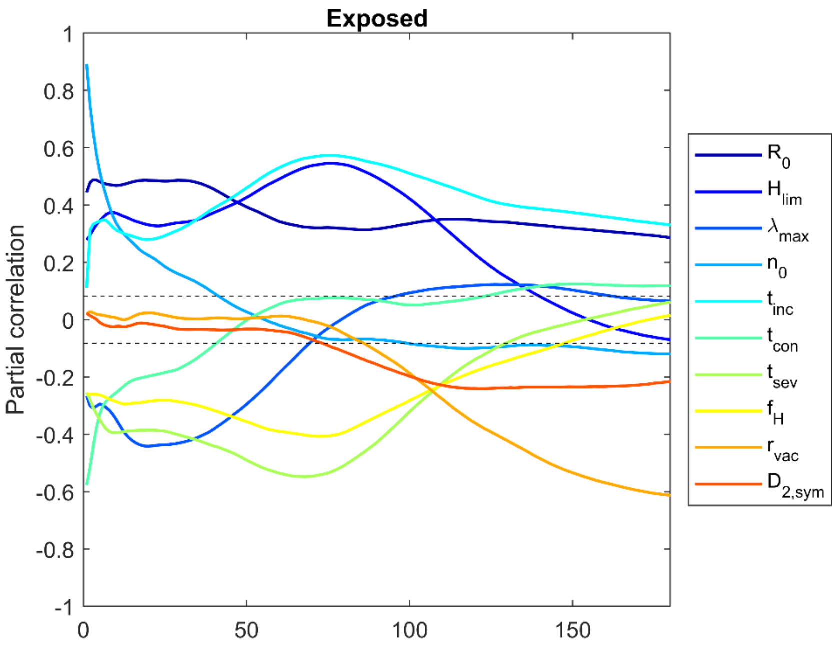

Figure 3.15. Partial correlation between input parameters and exposed individuals along time.

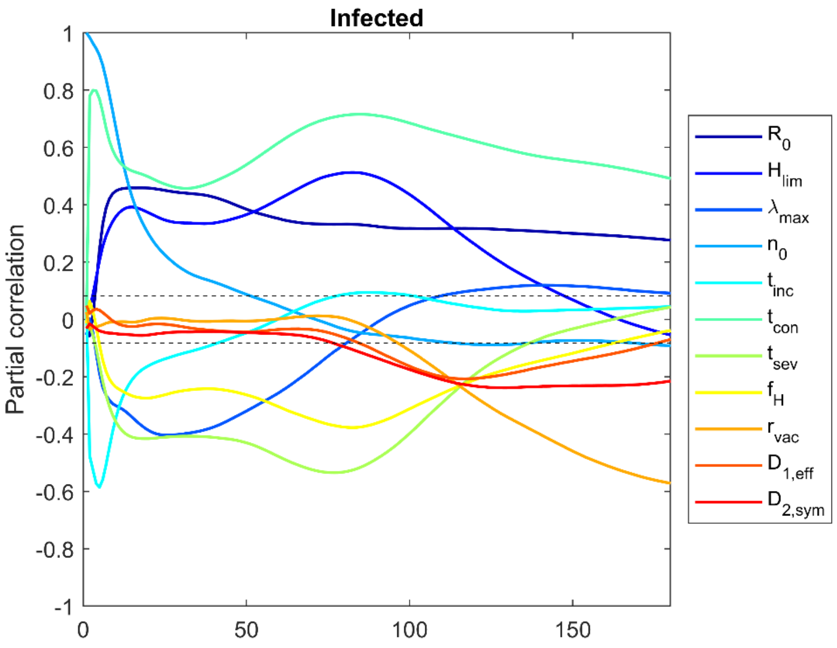

Figure 3.16. Partial correlation between input parameters and infected individuals along time.

3.Partial correlation between parameters and different outcomes

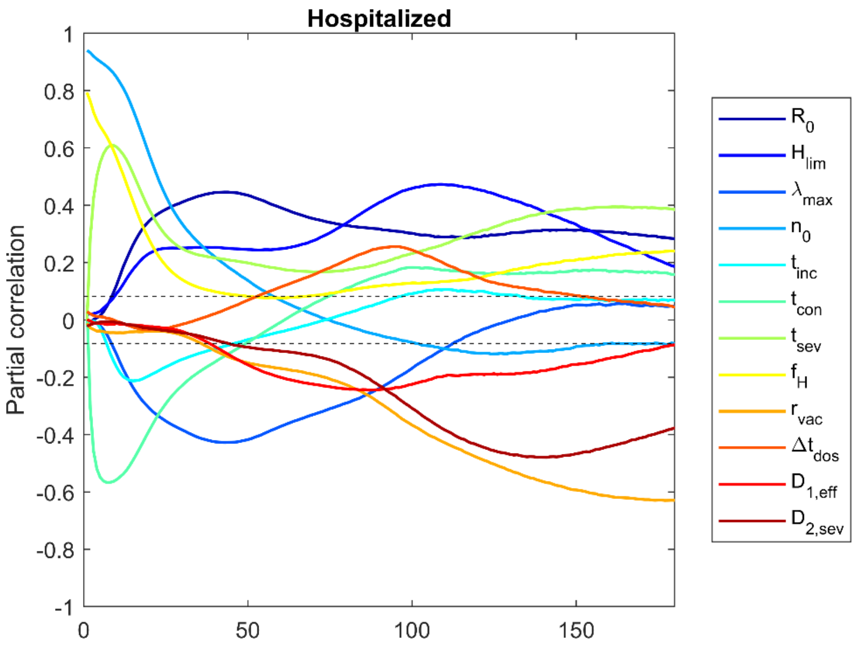

Figure 3.17. Partial correlation between input parameters and hospitalized individuals along time.

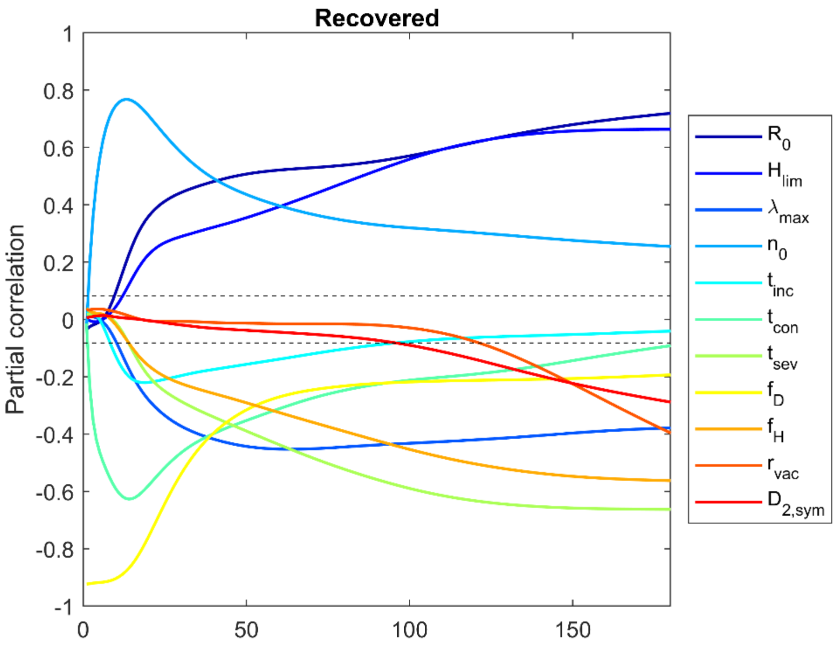

Figure 3.18. Partial correlation between input parameters and recovered individuals along time.

3.Partial correlation between parameters and different outcomes

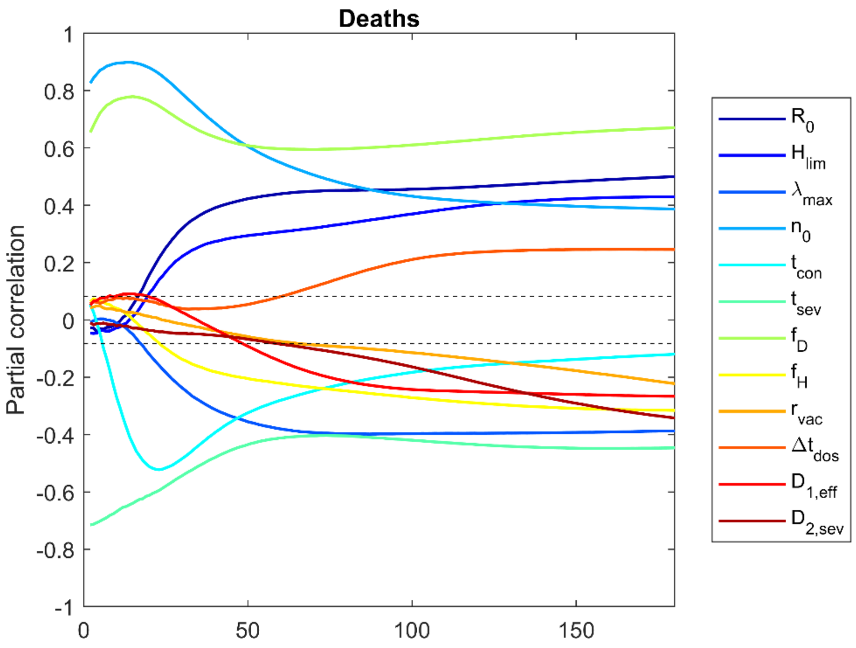

Figure 3.19. Partial correlation between input parameters and cumulative deaths along time.

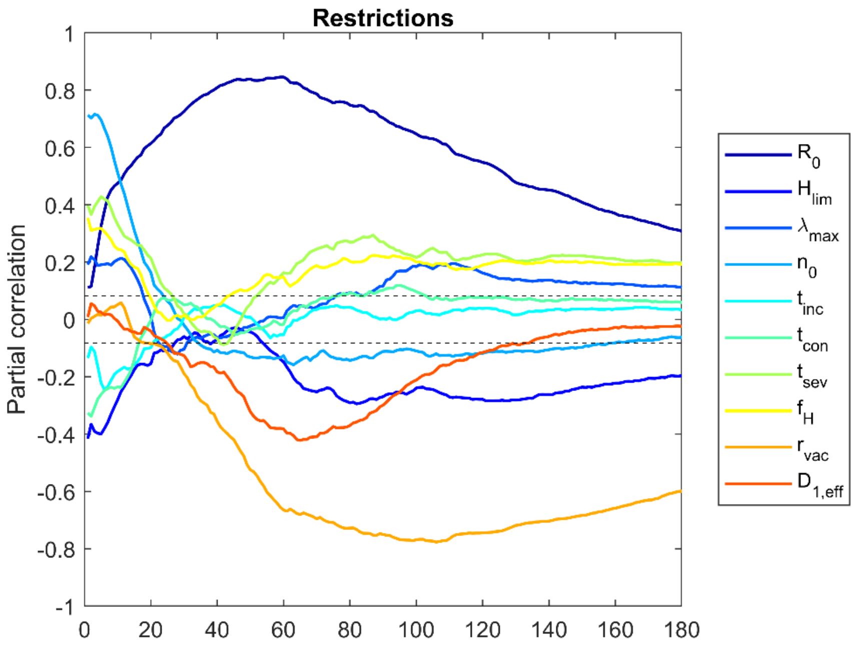

Figure 3.20. Partial correlation between input parameters and restrictions individuals along time.

3.Partial correlation between parameters and different outcomes

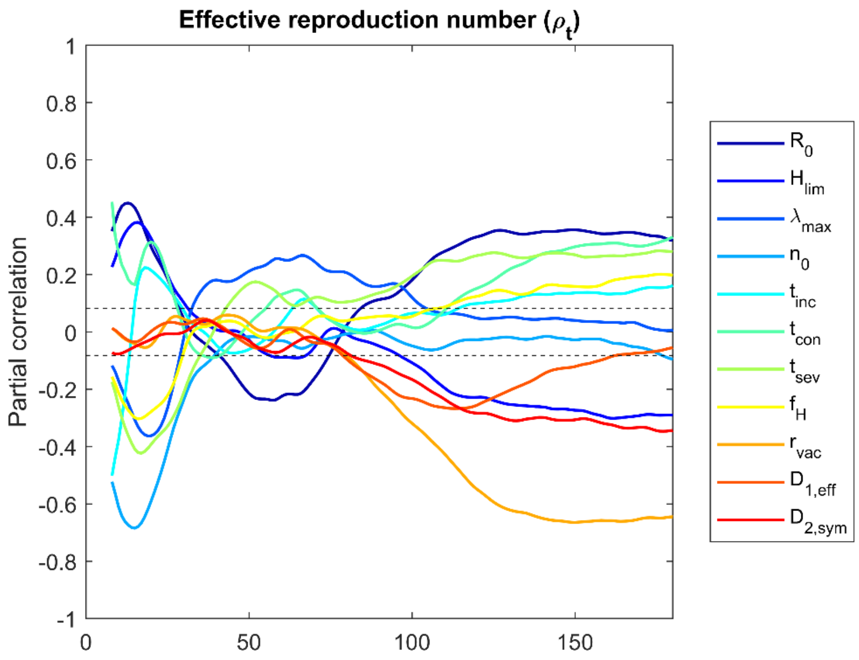

Figure 3.21. Partial correlation between input parameters and effective reproduction number along time.

4.Partial correlation between parameters and different compartments and restrictions

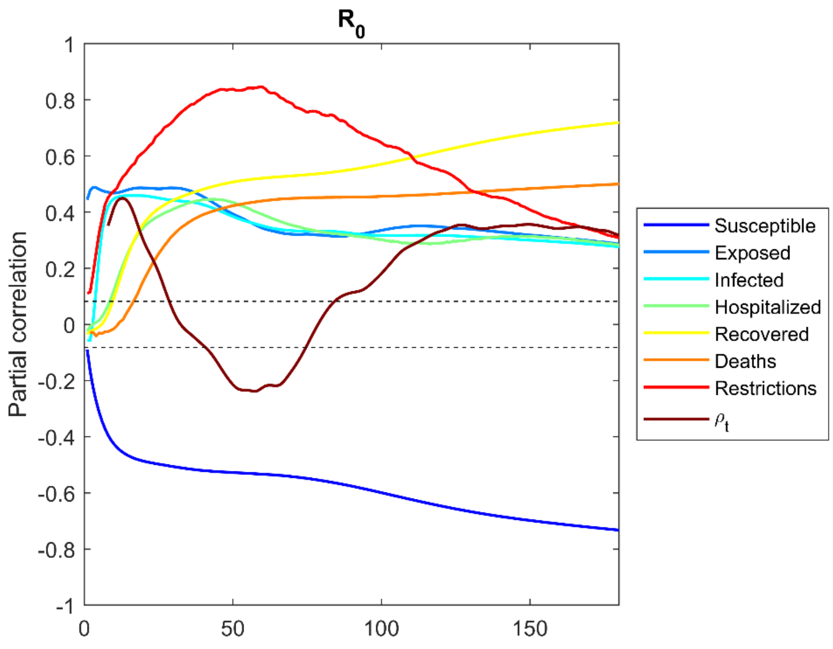

Figure 4.1. Partial correlation between input parameters reproductive basic number and different compartments and restrictions.

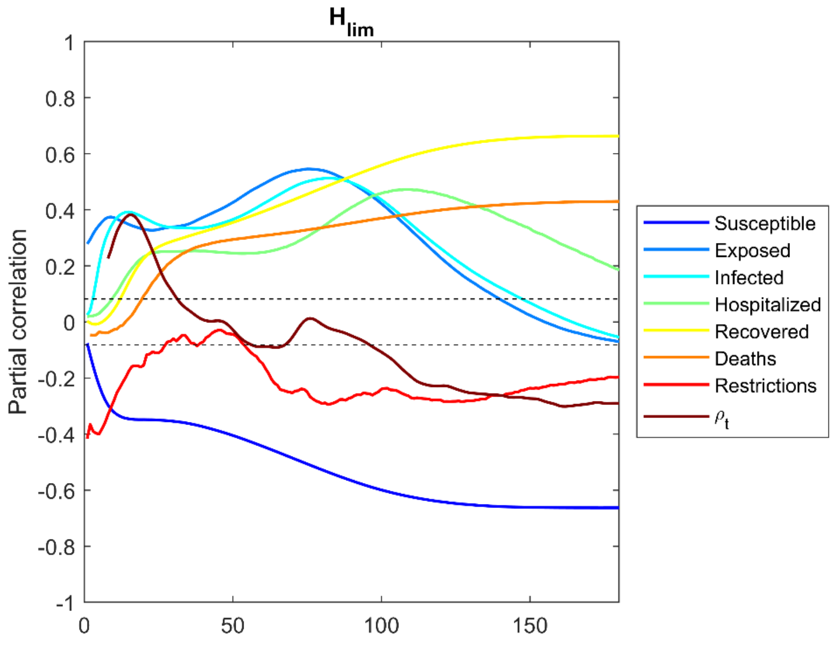

Figure 4.2. Partial correlation between input parameters hospital capacity limit and different compartments and restrictions.

4.Partial correlation between parameters and different compartments and restrictions

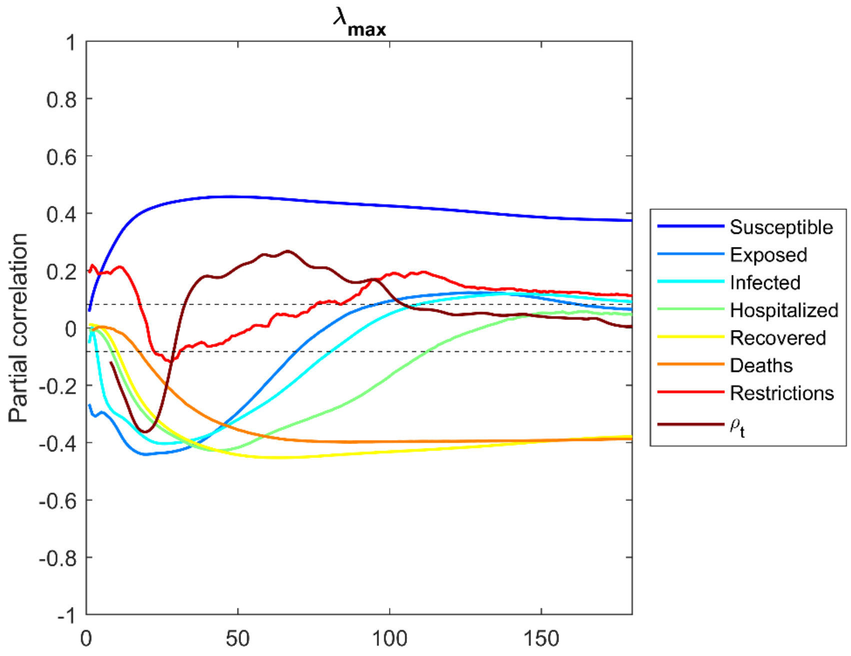

Figure 4.3. Partial correlation between input parameters maximum restrictions level and different compartments and restrictions.

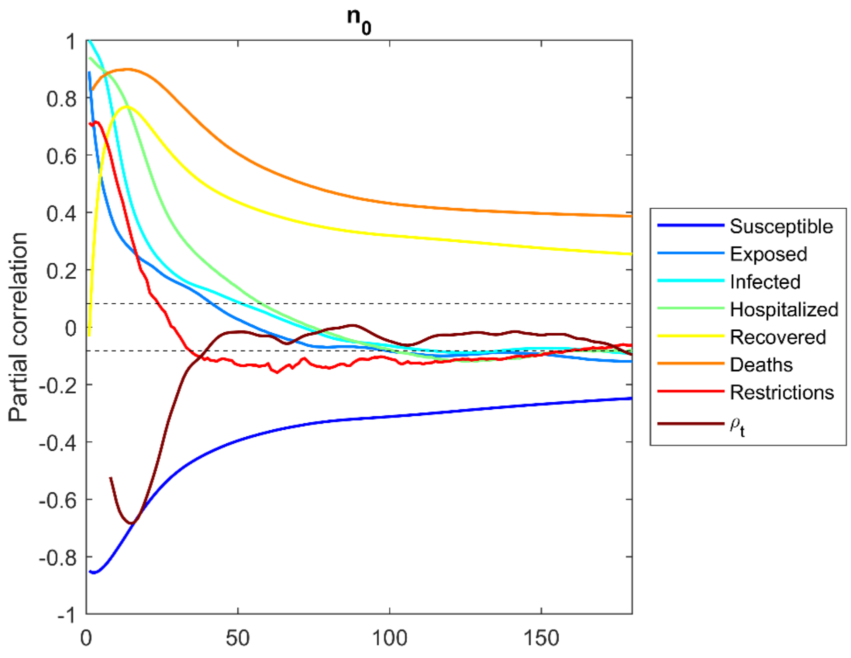

Figure 4.4. Partial correlation between input parameters initial daily cases and different compartments and restrictions.

4.Partial correlation between parameters and different compartments and restrictions

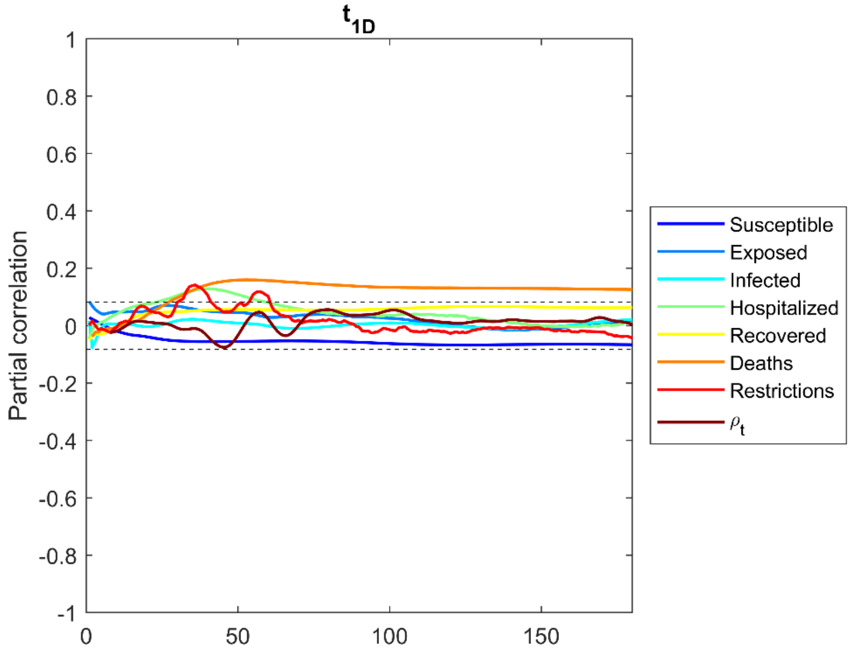

Figure 4.5. Partial correlation between input parameters time till first dose efficacy and different compartments and restrictions.

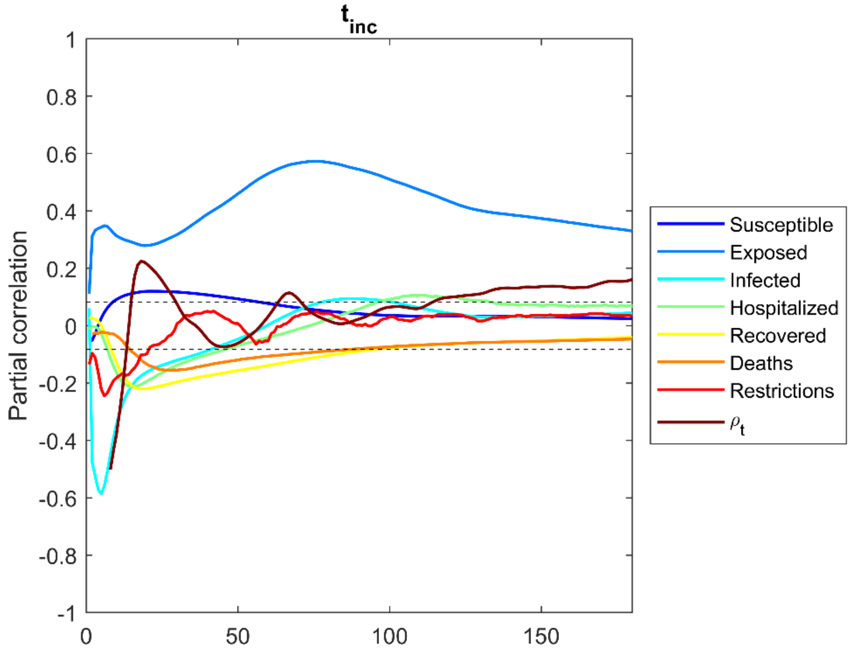

Figure 4.6. Partial correlation between input parameters incubation period and different compartments and restrictions.

4.Partial correlation between parameters and different compartments and restrictions

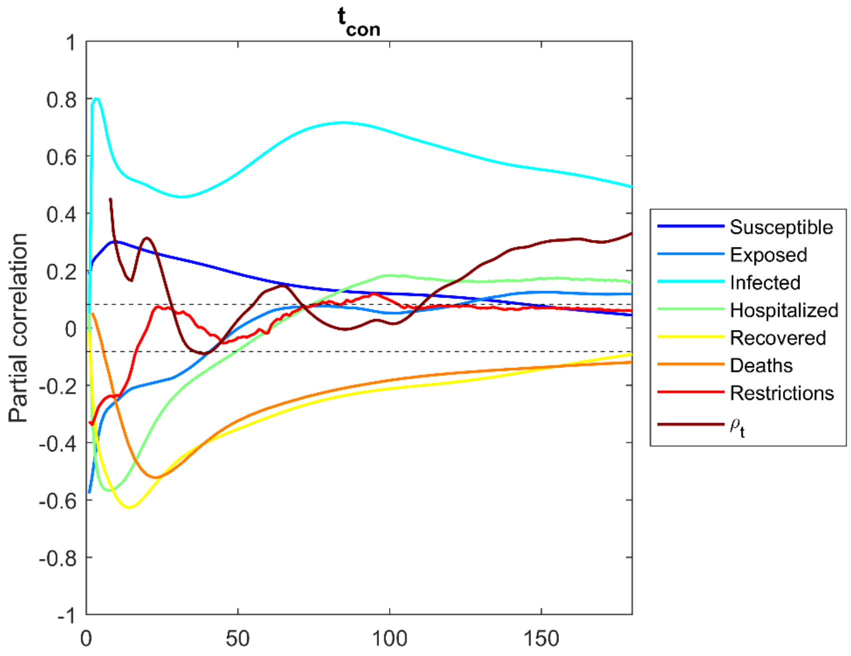

Figure 4.7. Partial correlation between input parameters contagiousness period and different compartments and restrictions.

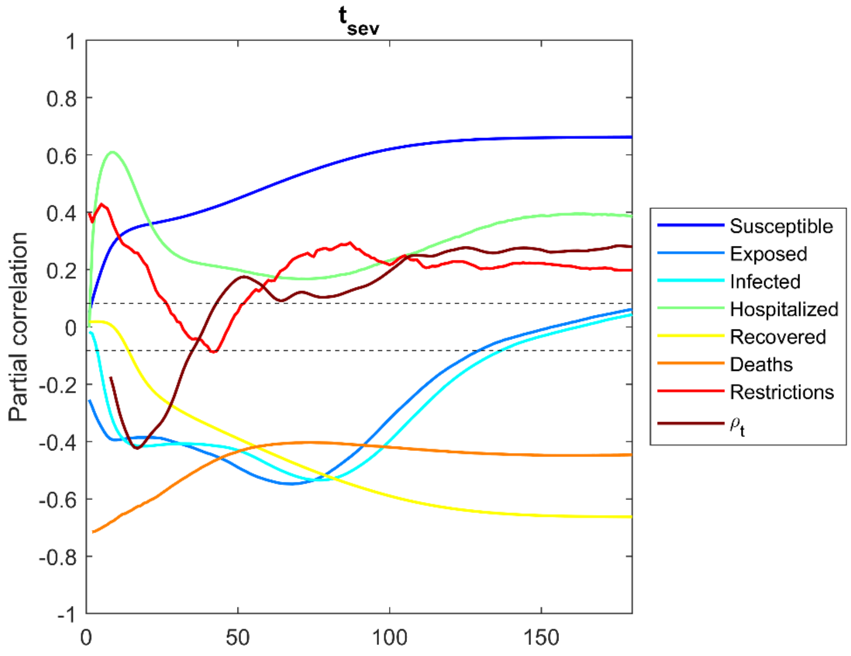

Figure 4.8. Partial correlation between input parameters disease time and different compartments and restrictions.

4.Partial correlation between parameters and different compartments and restrictions

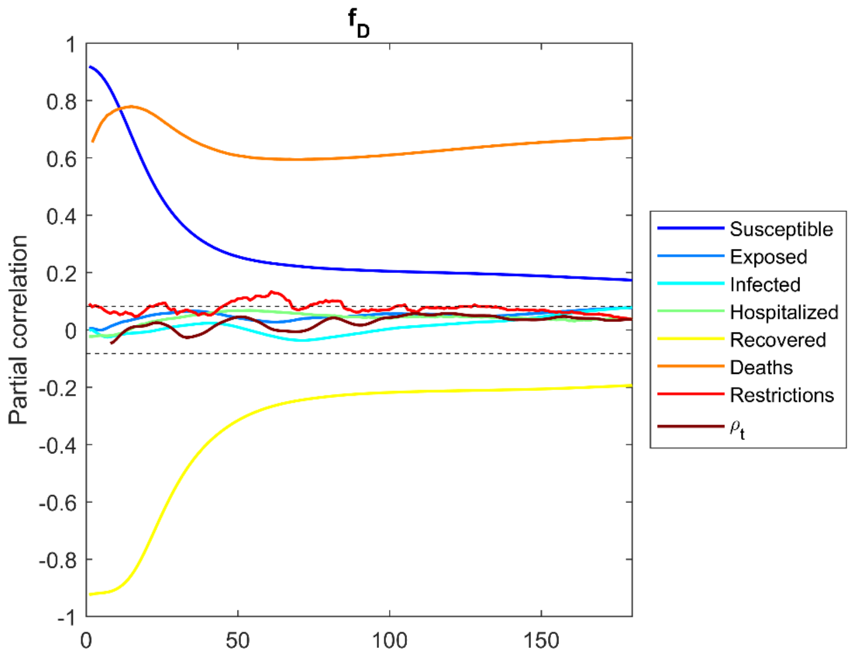

Figure 4.9. Partial correlation between input parameters mortality increase factor and different compartments and restrictions.

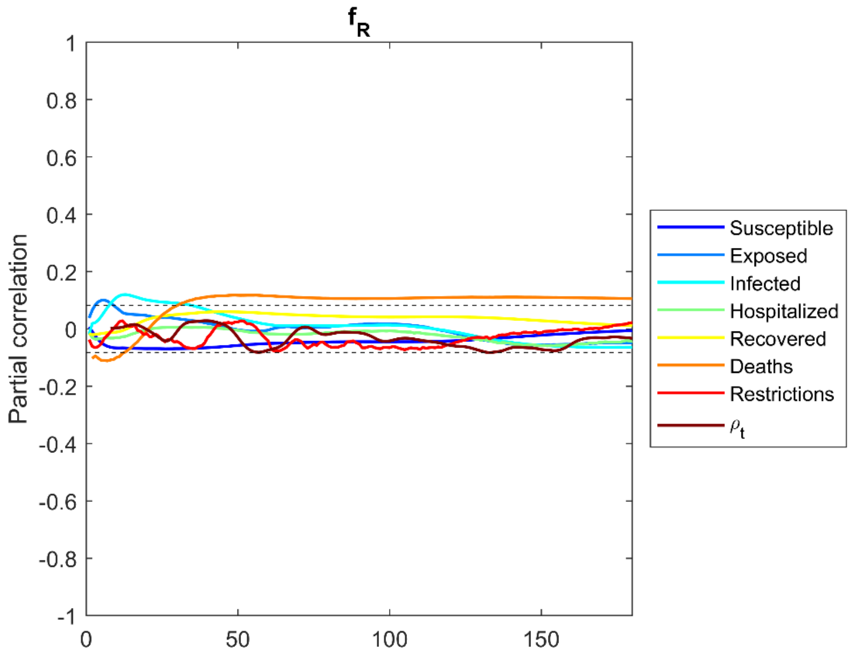

Figure 4.10. Partial correlation between input parameters residence increase factor and different compartments and restrictions.

4.Partial correlation between parameters and different compartments and restrictions

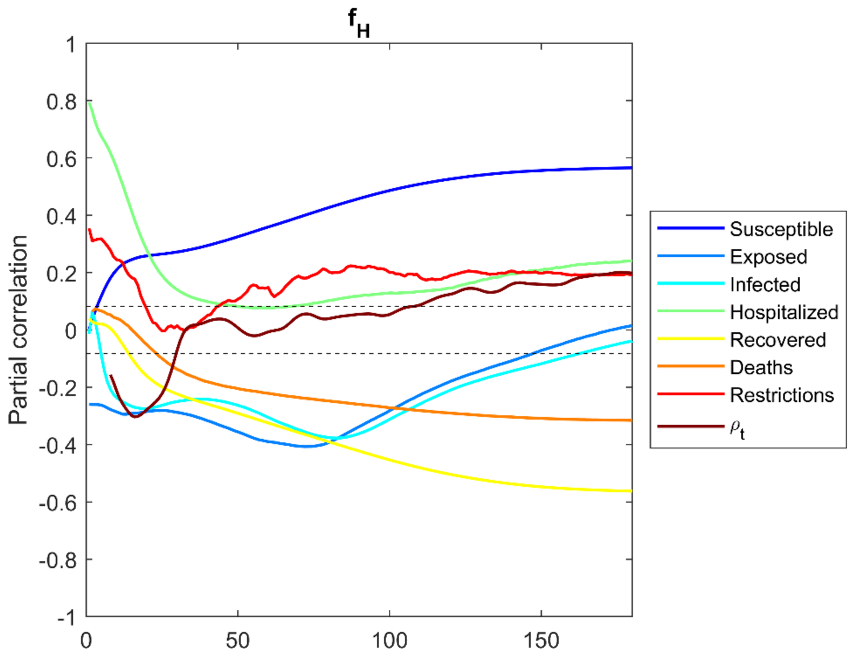

Figure 4.11. Partial correlation between input parameters hospitalization increase factor and different compartments and restrictions.

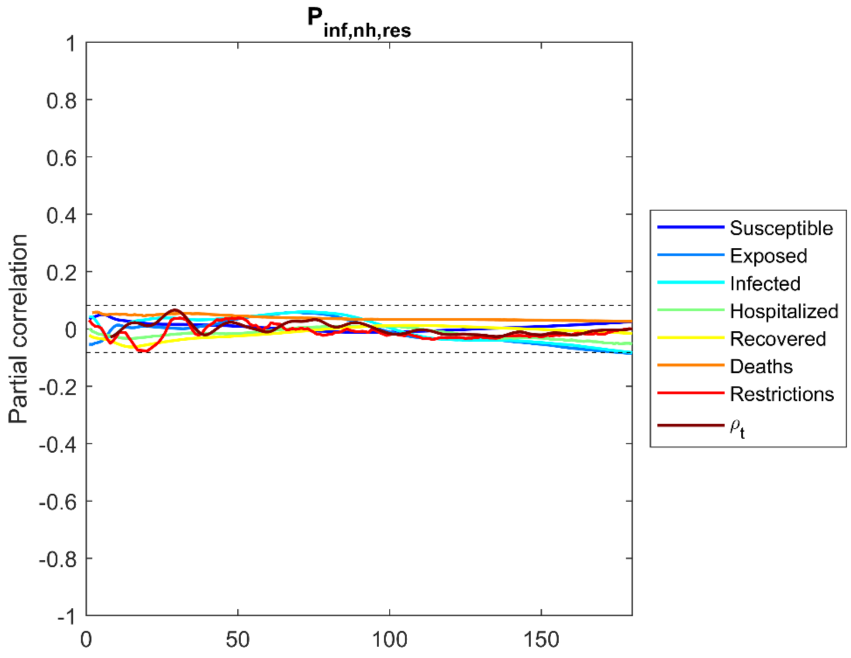

Figure 4.12. Partial correlation between input parameters probability that residents infect inside residence and different compartments and restrictions.

4.Partial correlation between parameters and different compartments and restrictions

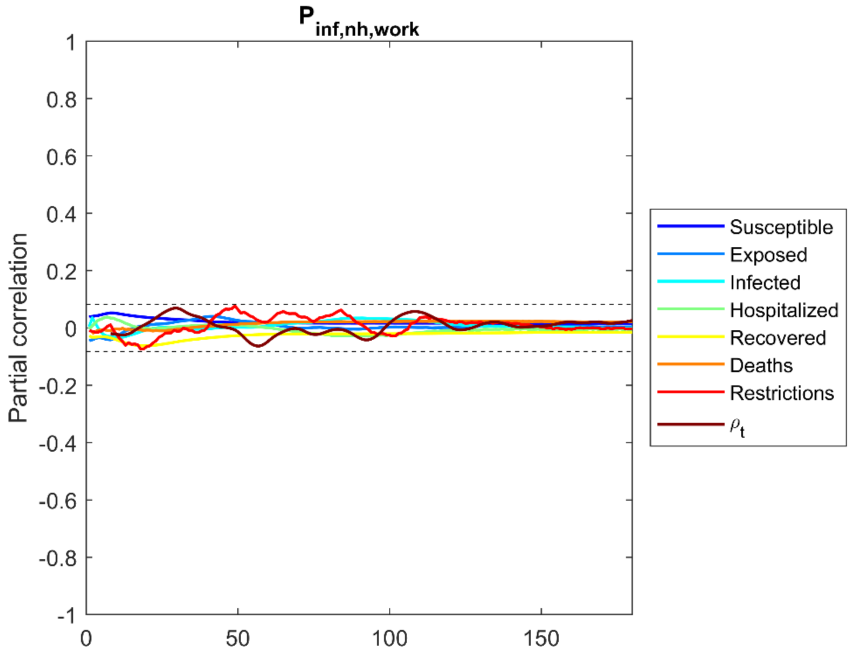

Figure 4.13. Partial correlation between input parameters probability that workers infect inside residence and different compartments and restrictions.

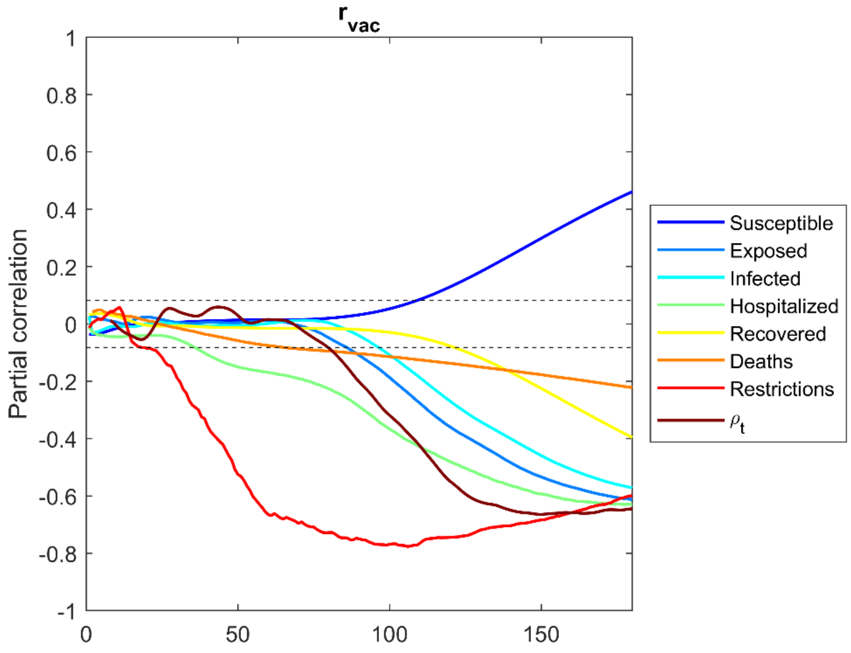

Figure 4.14. Partial correlation between input parameters vaccination speed and different compartments and restrictions.

4.Partial correlation between parameters and different compartments and restrictions

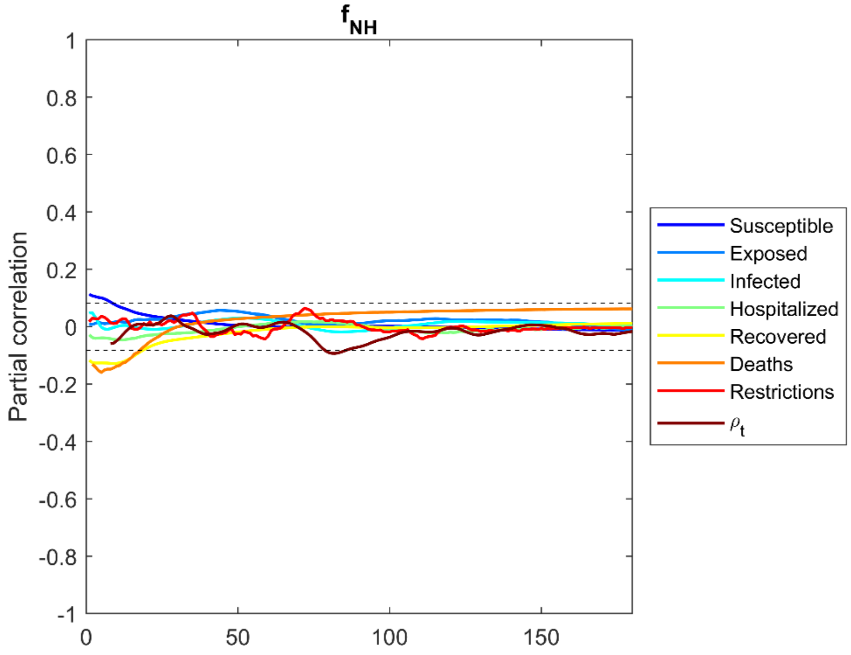

Figure 4.15. Partial correlation between input parameters increased death probability in care homes and different compartments and restrictions.

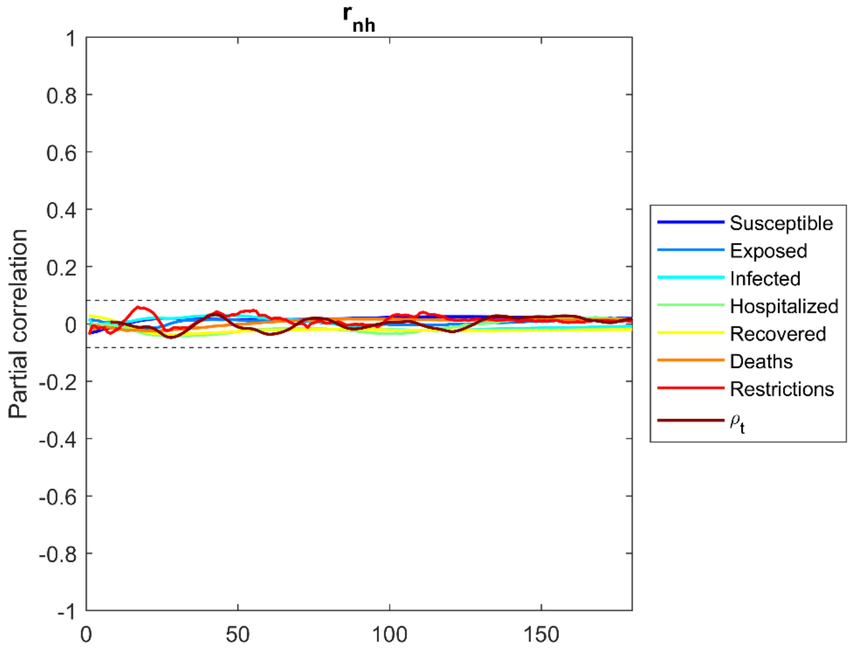

Figure 4.16. Partial correlation between input parameters rate of care homes and different compartments and restrictions.

4.Partial correlation between parameters and different compartments and restrictions

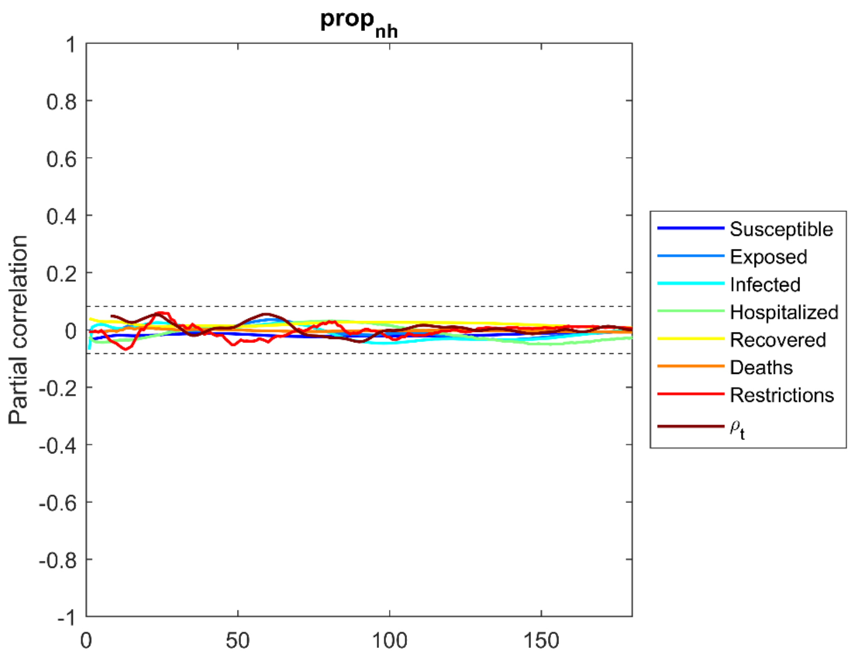

Figure 4.17. Partial correlation between input parameters proportion of nurses per resident and different compartments and restrictions.

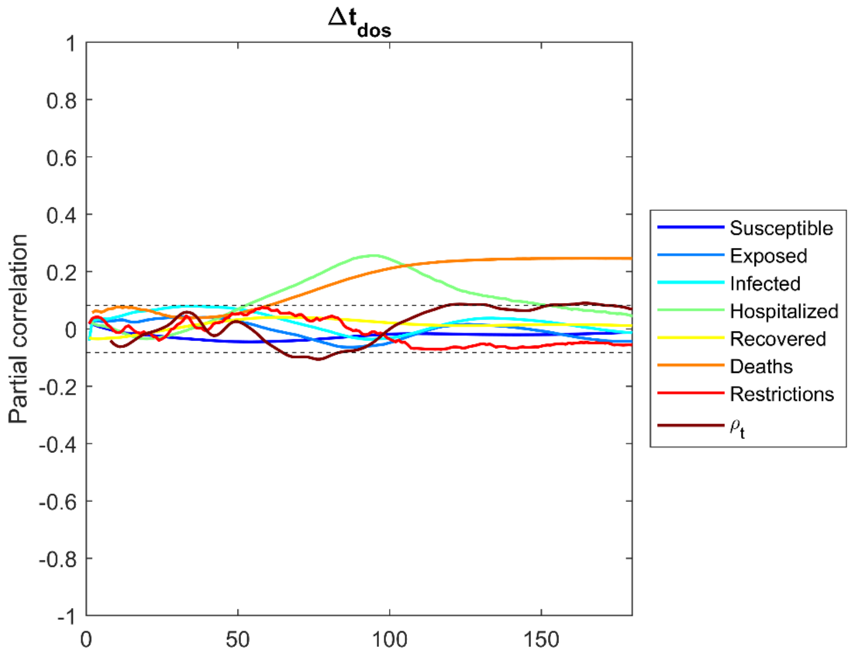

Figure 4.18. Partial correlation between input parameters time between doses and different compartments and restrictions.

4.Partial correlation between parameters and different compartments and restrictions

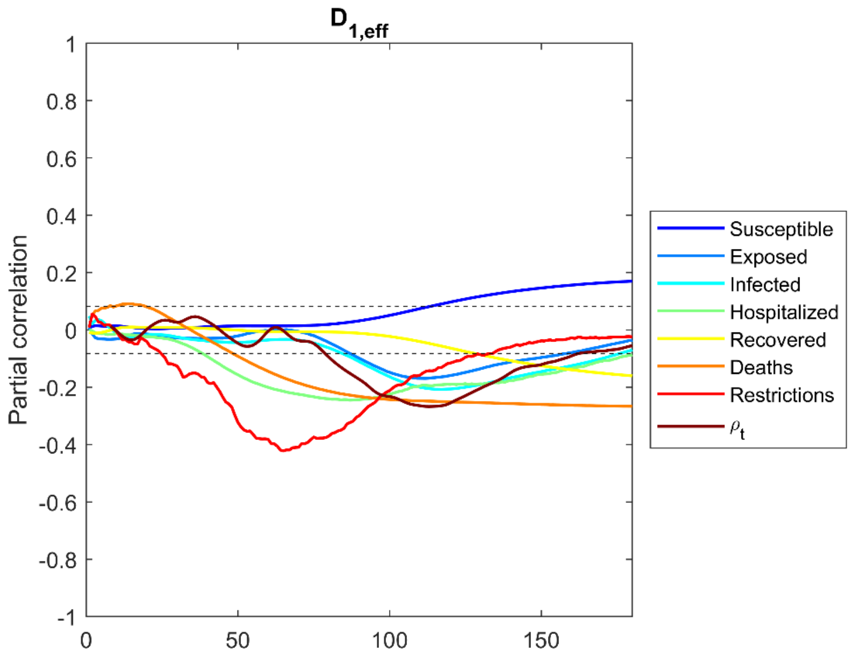

Figure 4.19. Partial correlation between input parameters efficacy of first dose and different compartments and restrictions.

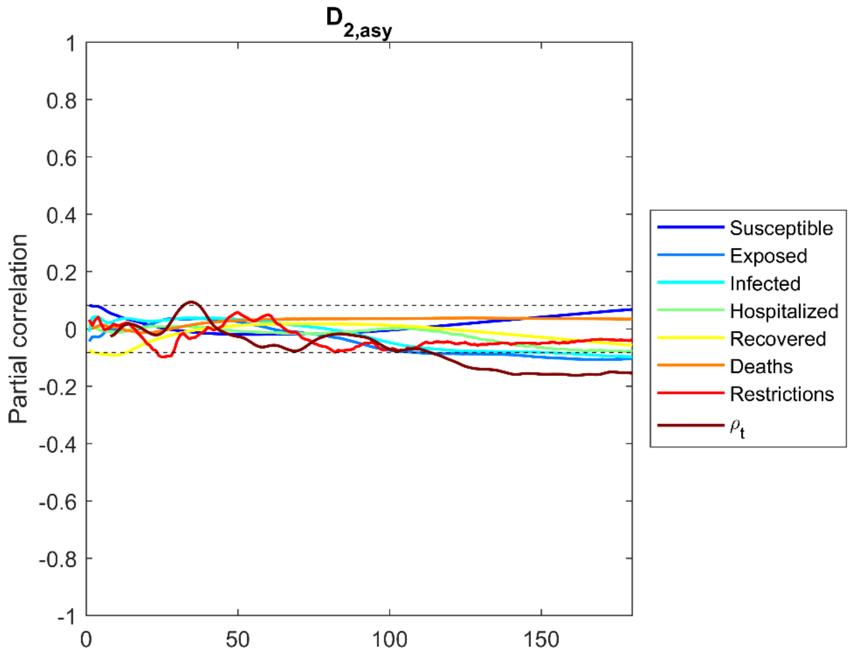

Figure 4.20. Partial correlation between input parameters efficacy of second dose on asymptomatics and different compartments and restrictions.

4.Partial correlation between parameters and different compartments and restrictions

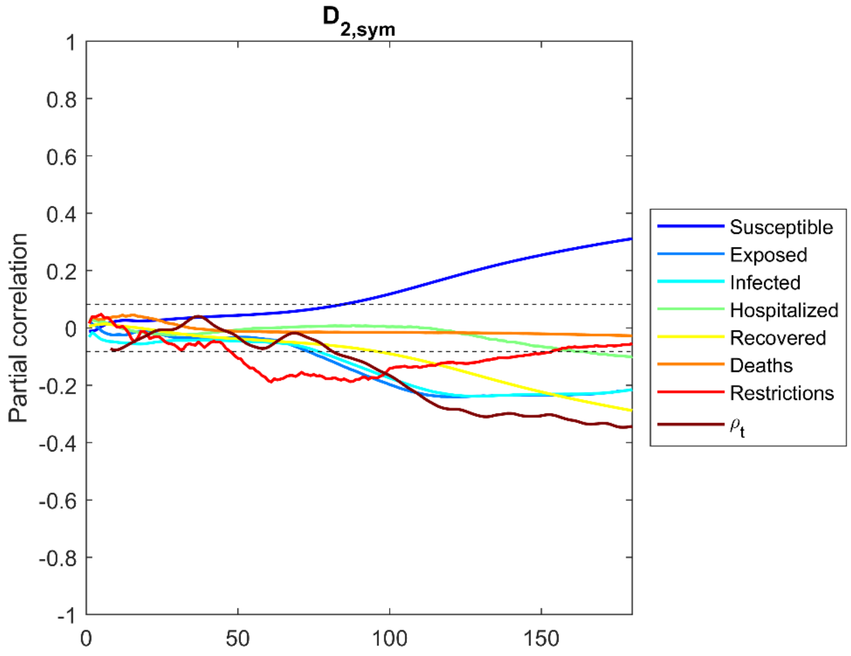

Figure 4.21. Partial correlation between input parameters efficacy of second dose on symptomatic and different compartments and restrictions.

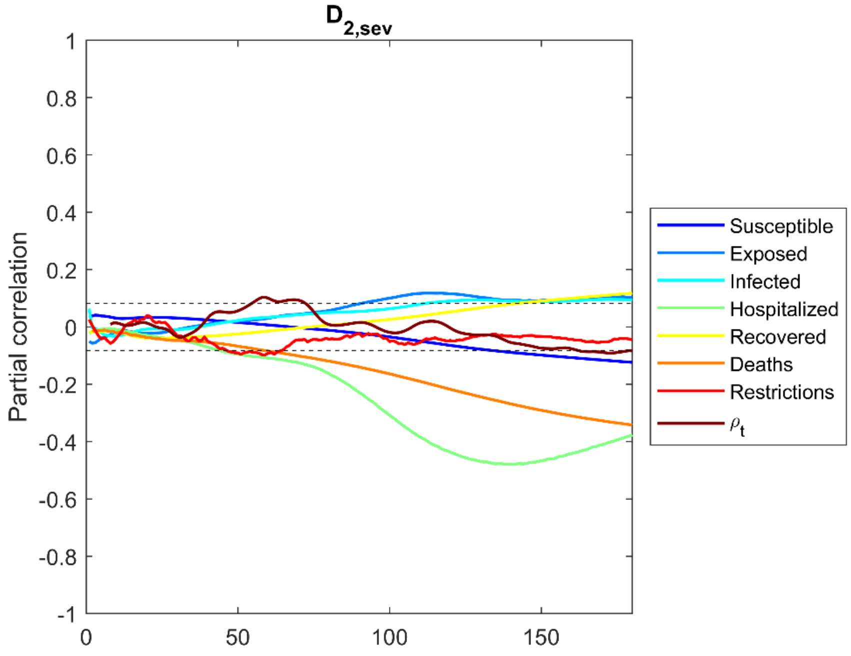

Figure 4.22. Partial correlation between input parameters efficacy of second dose on severe cases and different compartments and restrictions.

4.Partial correlation between parameters and different compartments and restrictions

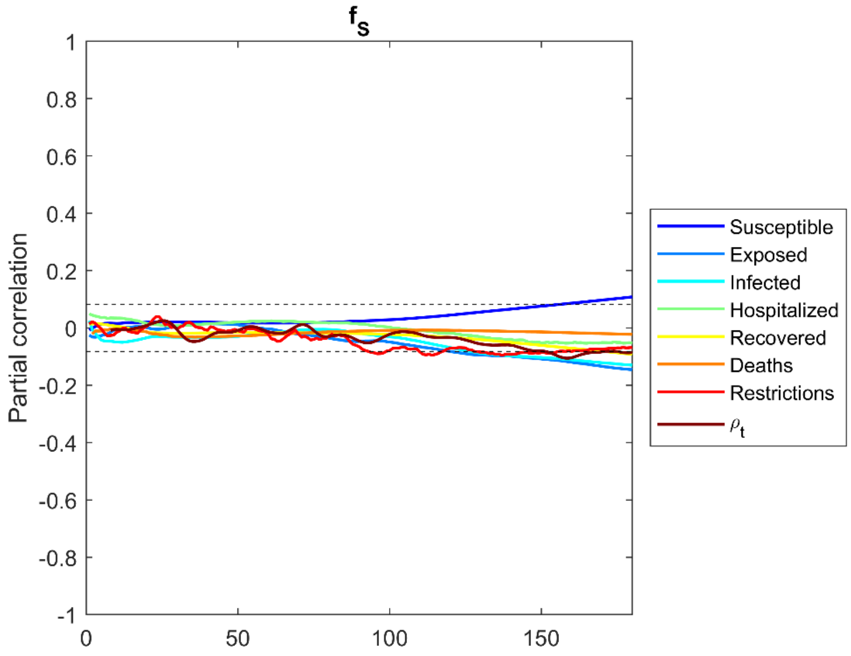

Figure 4.23. Partial correlation between input parameters increased symptomatic factor and different compartments and restrictions.
