# Supplementary material for: The impact of prioritisation and dosing intervals on the effects of COVID-19 vaccination in Europe: an agent-based cohort model
Source: Sci Rep. 2021 Sep 22;11:18812. doi: 10.1038/s41598-021-98216-0 (PMC8458447; doi:10.1038/s41598-021-98216-0)
Supplement: Supplementary file 4 — Supplementary Information 4. [file 41598_2021_98216_MOESM4_ESM.pdf]

The impact of prioritisation and dosing intervals on the effects of COVID-19 vaccination in Europe: an agent-based cohort model

Martí Català, Xintong Li, Clara Prats*, Daniel Prieto-Alhambra

*clara.prats@upc.edu

SUPPLEMENTARY MATERIAL: Different configurations figures

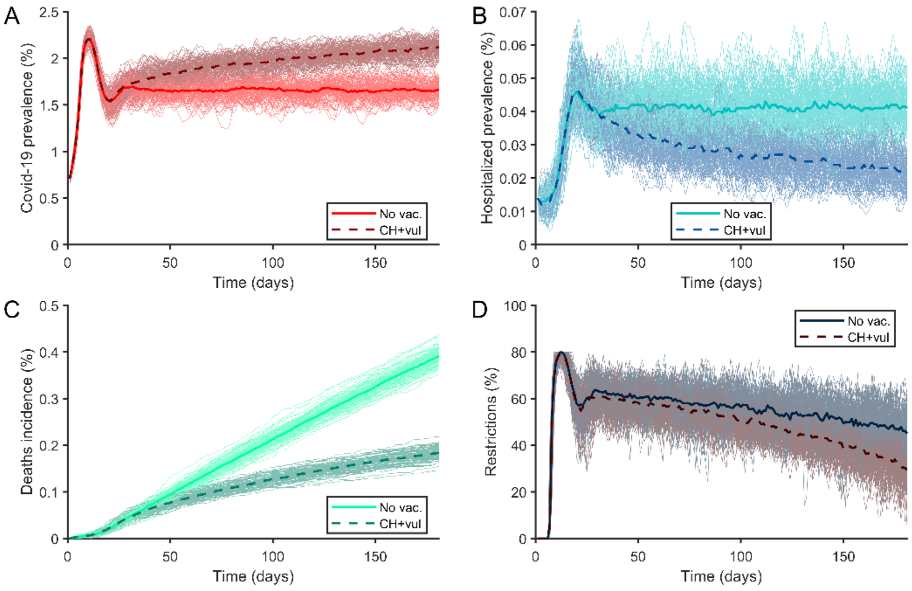

Figure 1. Output from the agent based model for the impact of COVID-19 in Europe without vaccination (No vac.) vs vaccinating with AZ vaccine with a 4 weeks separation between doses. Vaccination strategy: prioritizing CH residents and vulnerable (CH+vul).Using default values for all parameters.

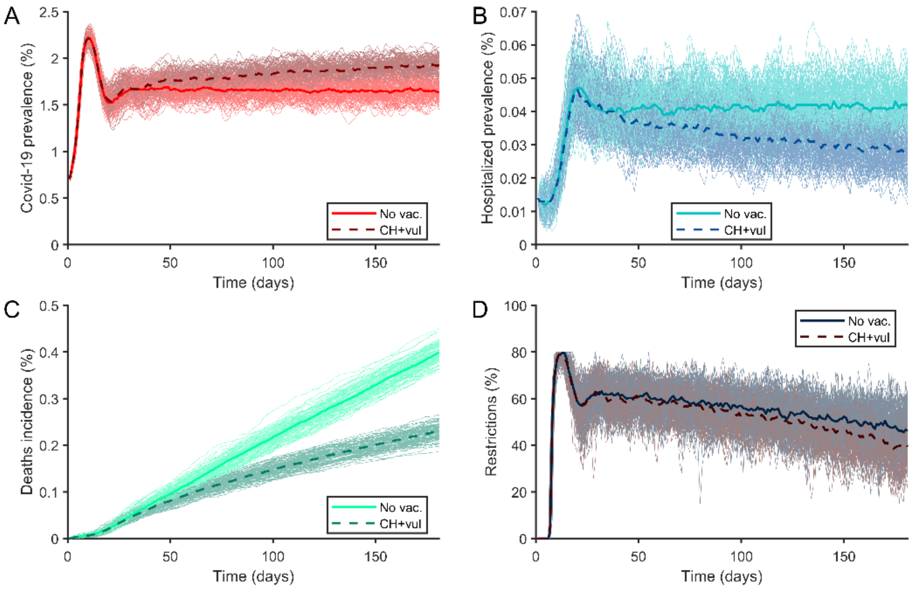

Figure 2. Output from the agent based model for the impact of COVID-19 in Europe without vaccination (No vac.) vs vaccinating with AZ vaccine with a 4 weeks separation between doses. Vaccination strategy: prioritizing CH residents and vulnerable (CH+vul).Vaccination speed: 0.1%, other parameters with their default value.

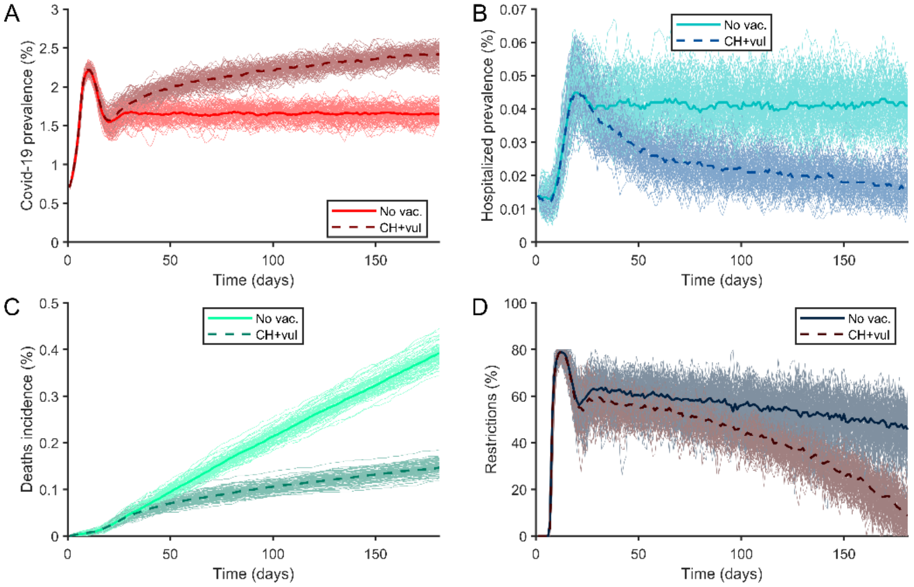

Figure 3. Output from the agent based model for the impact of COVID-19 in Europe without vaccination (No vac.) vs vaccinating with AZ vaccine with a 4 weeks separation between doses. Vaccination strategy: prioritizing CH residents and vulnerable (CH+vul).Vaccination speed: 0.4%, other parameters with their default value.

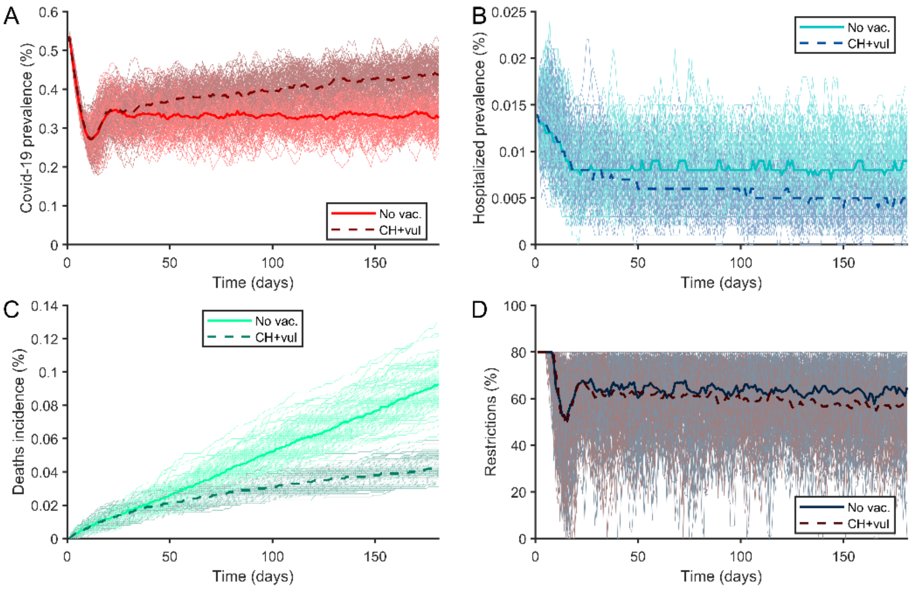

Figure 4. Output from the agent based model for the impact of COVID-19 in Europe without vaccination (No vac.) vs vaccinating with AZ vaccine with a 4 weeks separation between doses. Vaccination strategy: prioritizing CH residents and vulnerable (CH+vul).Hospital limit 100 individuals per million, other parameters with their default value.

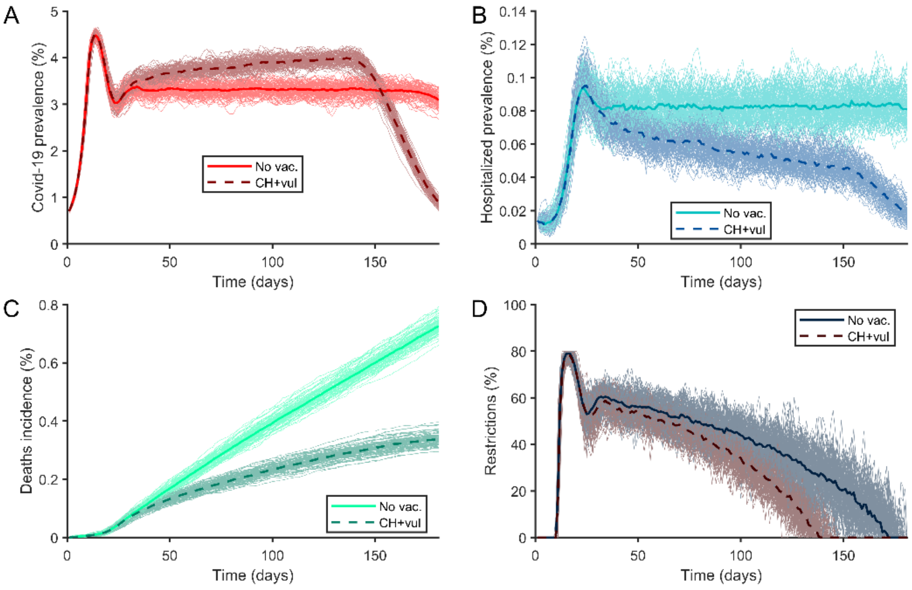

Figure 5. Output from the agent based model for the impact of COVID-19 in Europe without vaccination (No vac.) vs vaccinating with AZ vaccine with a 4 weeks separation between doses. Vaccination strategy: prioritizing CH residents and vulnerable (CH+vul).Hospital limit 1000 individuals per million, other parameters with their default value.

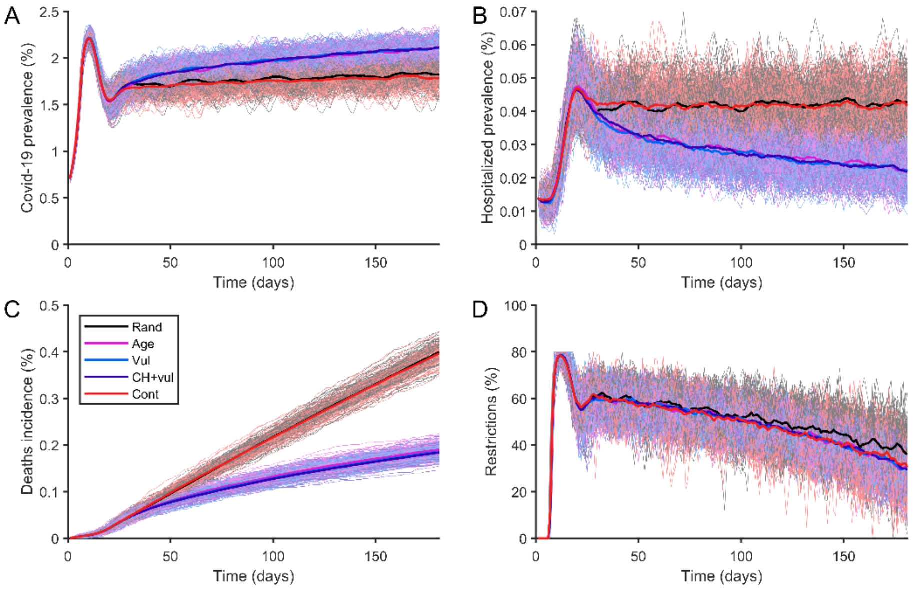

Figure 6. Proportion infected (A), hospitalized (B), and dead (C), and impact on NPI restrictions (D) according to prioritization strategy: random (black), contagiousness (red), age-based (pink), vulnerable (blue), and vulnerable+CH residents (dark-blue).Using default values for all parameters.

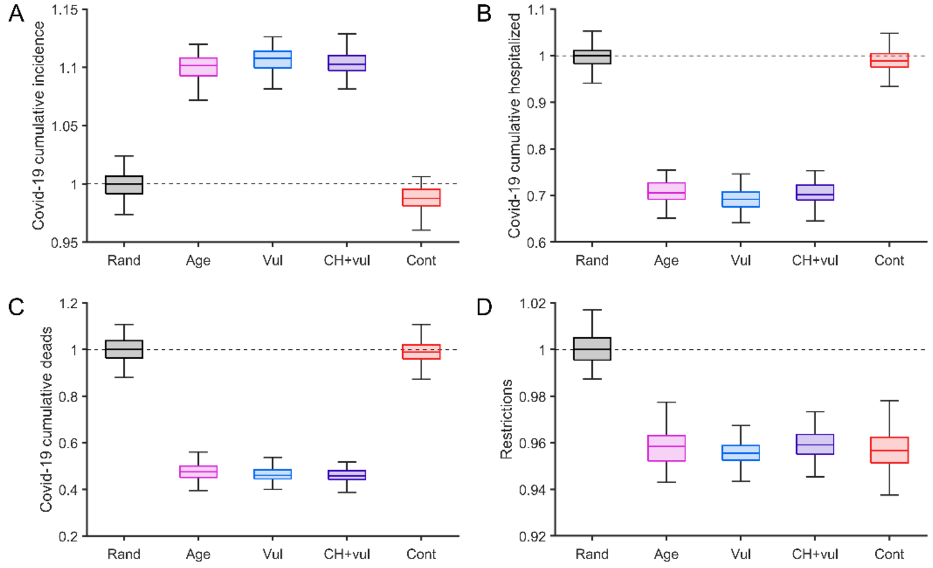

Figure 7. Boxplots to compare proportion infected (A), hospitalized (B), and dead (C), and impact on NPI restrictions (D) according to prioritization strategy: random (black), contagiousness (red), age-based (pink), vulnerable (blue), and vulnerable+CH residents (dark-blue). Values are referred to random strategy. Using default values for all parameters.

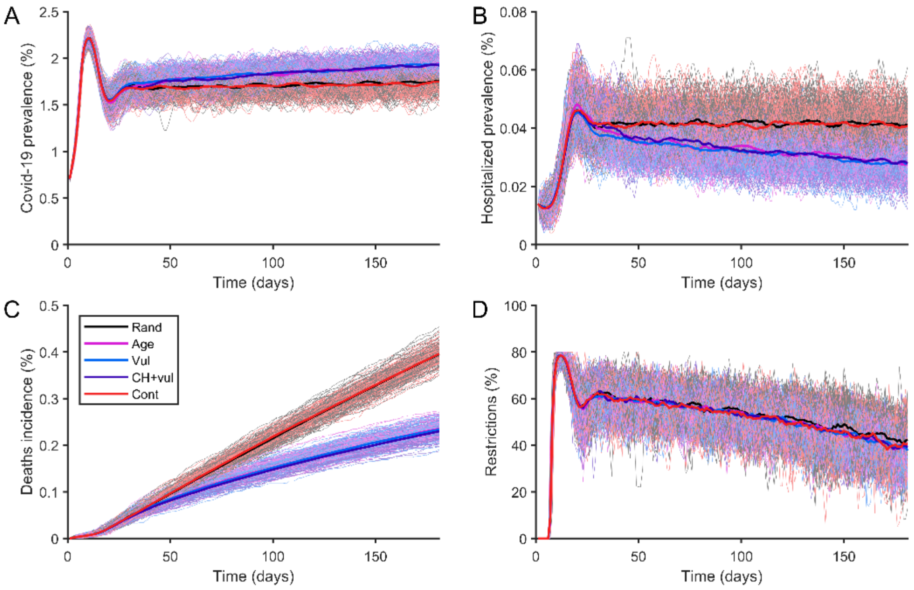

Figure 8. Proportion infected (A), hospitalized (B), and dead (C), and impact on NPI restrictions (D) according to prioritization strategy: random (black), contagiousness (red), age-based (pink), vulnerable (blue), and vulnerable+CH residents (dark-blue).Vaccination speed: 0.1%, other parameters with their default value.

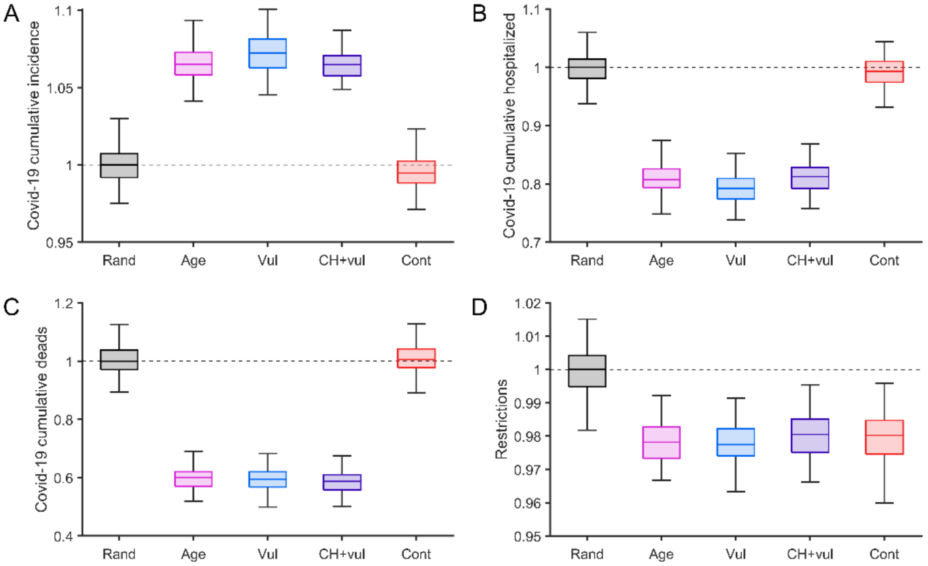

Figure 9. Boxplots to compare proportion infected (A), hospitalized (B), and dead (C), and impact on NPI restrictions (D) according to prioritization strategy: random (black), contagiousness (red), age-based (pink), vulnerable (blue), and vulnerable+CH residents (dark-blue). Values are referred to random strategy. Vaccination speed: 0.1%, other parameters with their default value.

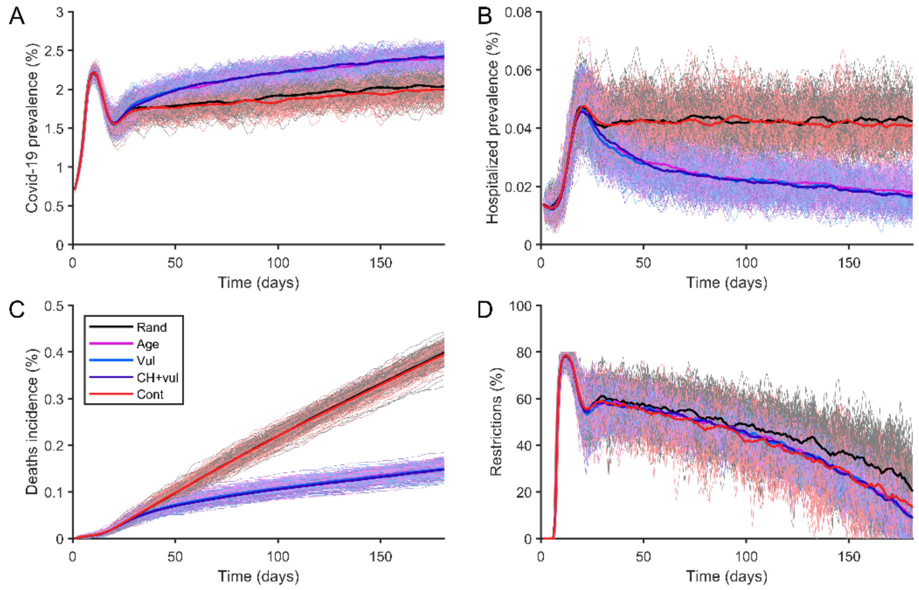

Figure 10. Proportion infected (A), hospitalized (B), and dead (C), and impact on NPI restrictions (D) according to prioritization strategy: random (black), contagiousness (red), age-based (pink), vulnerable (blue), and vulnerable+CH residents (dark-blue).Vaccination speed: 0.4%, other parameters with their default value.

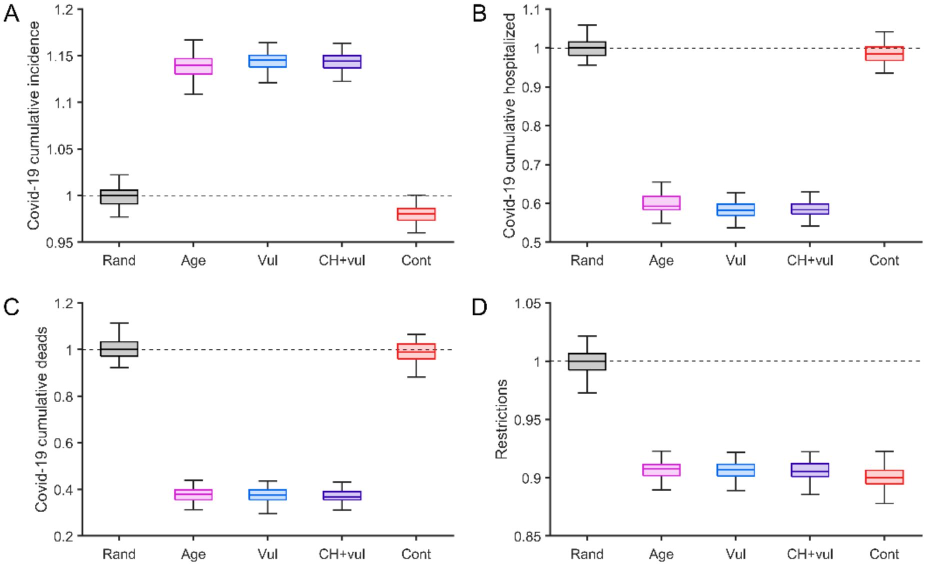

Figure 11. Boxplots to compare proportion infected (A), hospitalized (B), and dead (C), and impact on NPI restrictions (D) according to prioritization strategy: random (black), contagiousness (red), age-based (pink), vulnerable (blue), and vulnerable+CH residents (dark-blue). Values are referred to random strategy. Vaccination speed: 0.4%, other parameters with their default value.

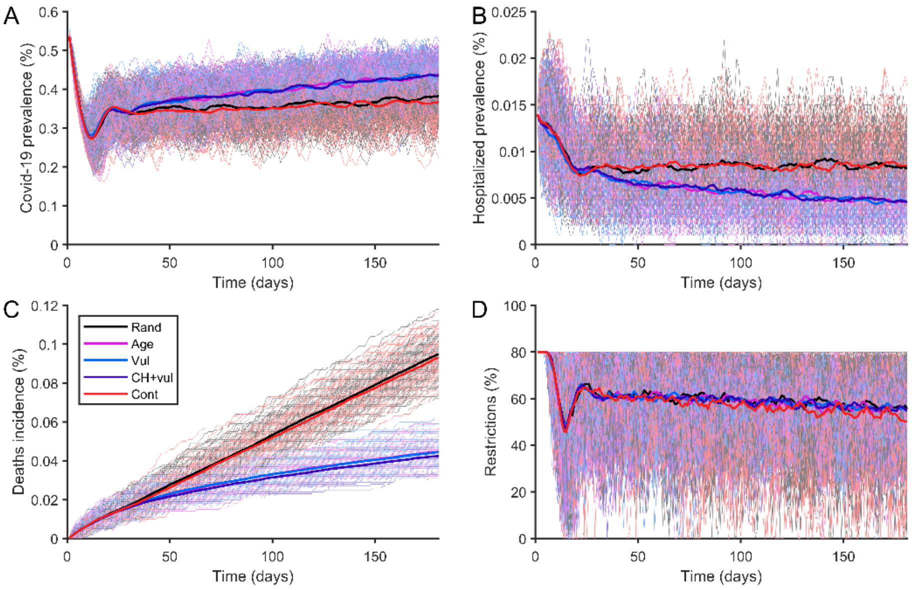

Figure 12. Proportion infected (A), hospitalized (B), and dead (C), and impact on NPI restrictions (D) according to prioritization strategy: random (black), contagiousness (red), age-based (pink), vulnerable (blue), and vulnerable+CH residents (dark-blue).Hospital limit 100 individuals per million, other parameters with their default value.

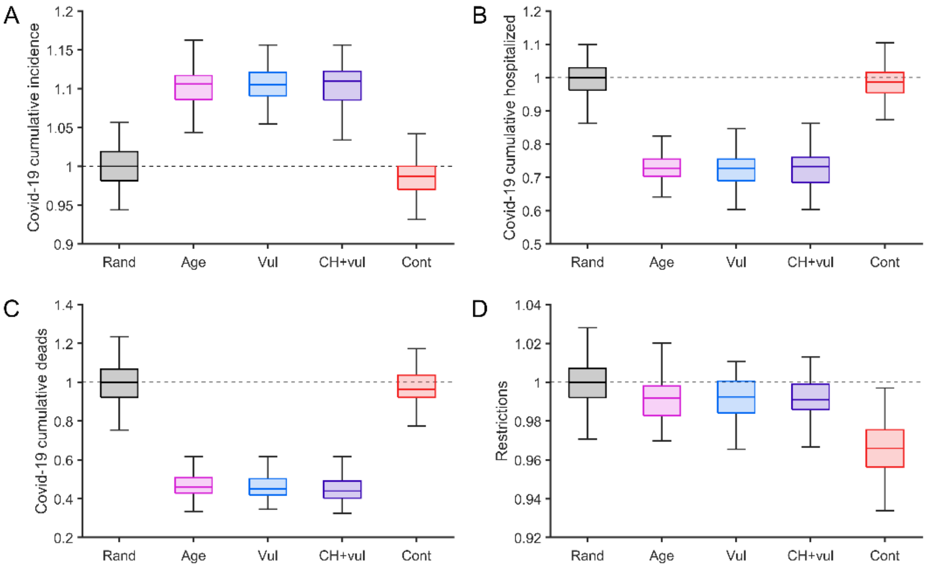

Figure 13. Boxplots to compare proportion infected (A), hospitalized (B), and dead (C), and impact on NPI restrictions (D) according to prioritization strategy: random (black), contagiousness (red), age-based (pink), vulnerable (blue), and vulnerable+CH residents (dark-blue). Values are referred to random strategy. Hospital limit 100 individuals per million, other parameters with their default value.

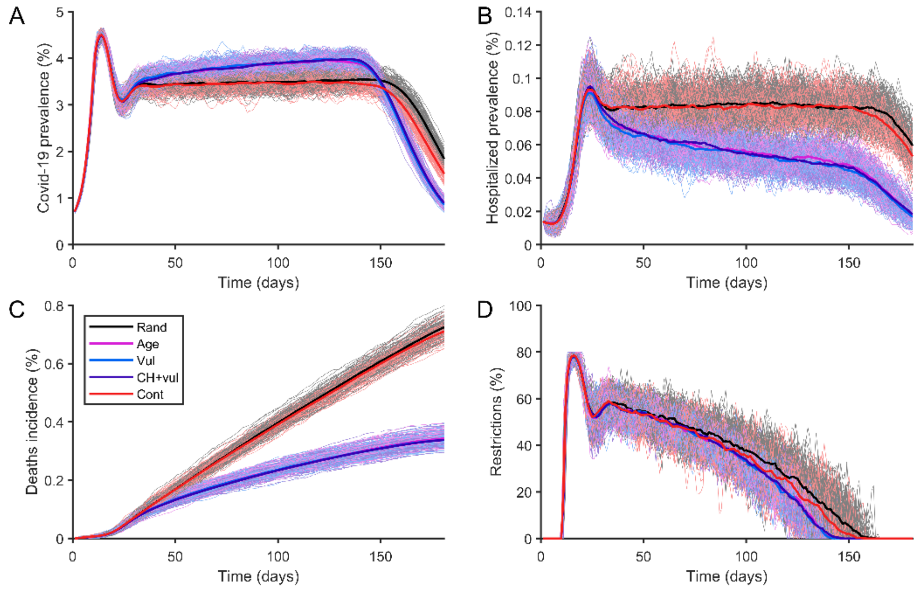

Figure 14. Proportion infected (A), hospitalized (B), and dead (C), and impact on NPI restrictions (D) according to prioritization strategy: random (black), contagiousness (red), age-based (pink), vulnerable (blue), and vulnerable+CH residents (dark-blue).Hospital limit 1000 individuals per million, other parameters with their default value.

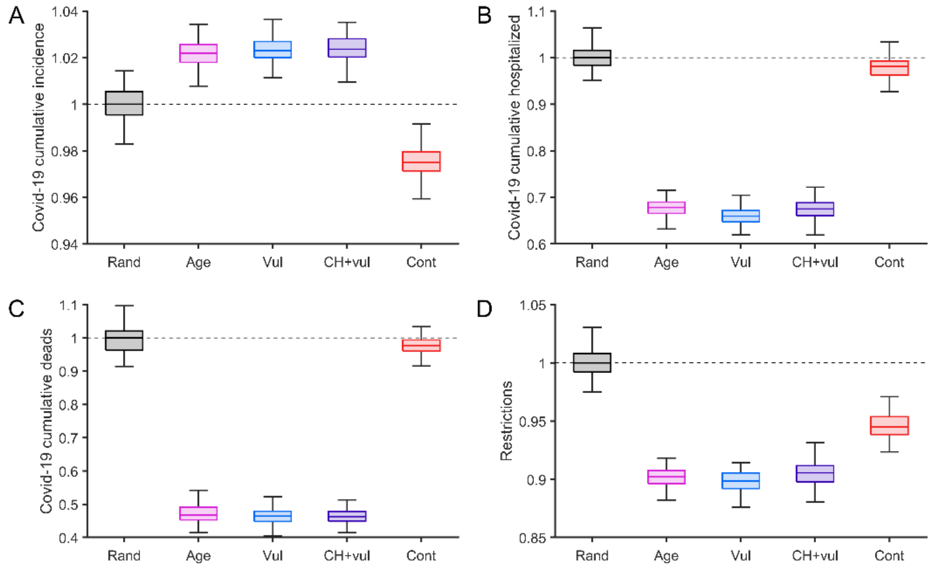

Figure 15. Boxplots to compare proportion infected (A), hospitalized (B), and dead (C), and impact on NPI restrictions (D) according to prioritization strategy: random (black), contagiousness (red), age-based (pink), vulnerable (blue), and vulnerable+CH residents (dark-blue). Values are referred to random strategy. Hospital limit 1000 individuals per million, other parameters with their default value.

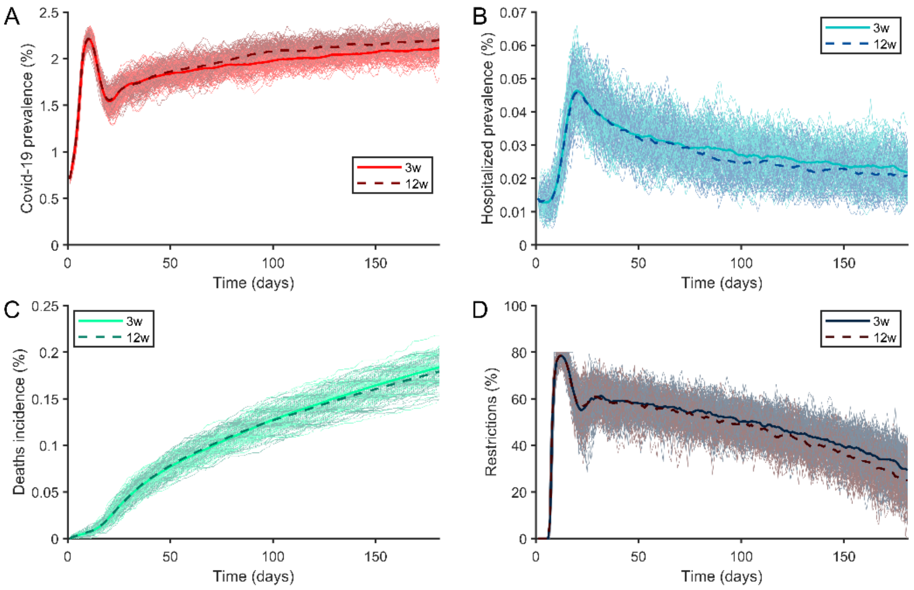

Figure 16. Output from the agent based model for the impact of COVID-19 in Europe using AztraZeneca vaccines with between-dose intervals of 3 vs 12 weeks. Using default values for all parameters.

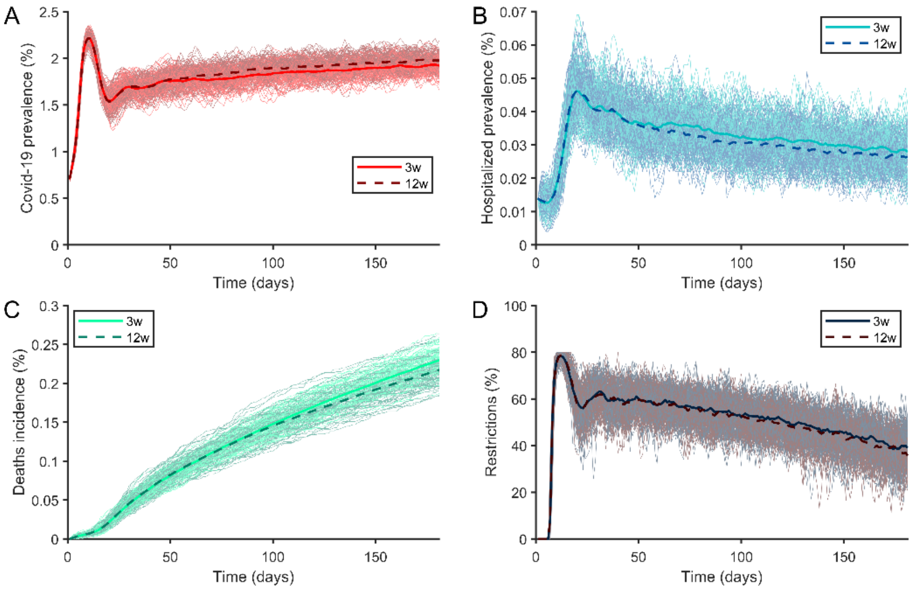

Figure 17. Output from the agent based model for the impact of COVID-19 in Europe using AztraZeneca vaccines with between-dose intervals of 3 vs 12 weeks. Vaccination speed: 0.1%, other parameters with their default value.

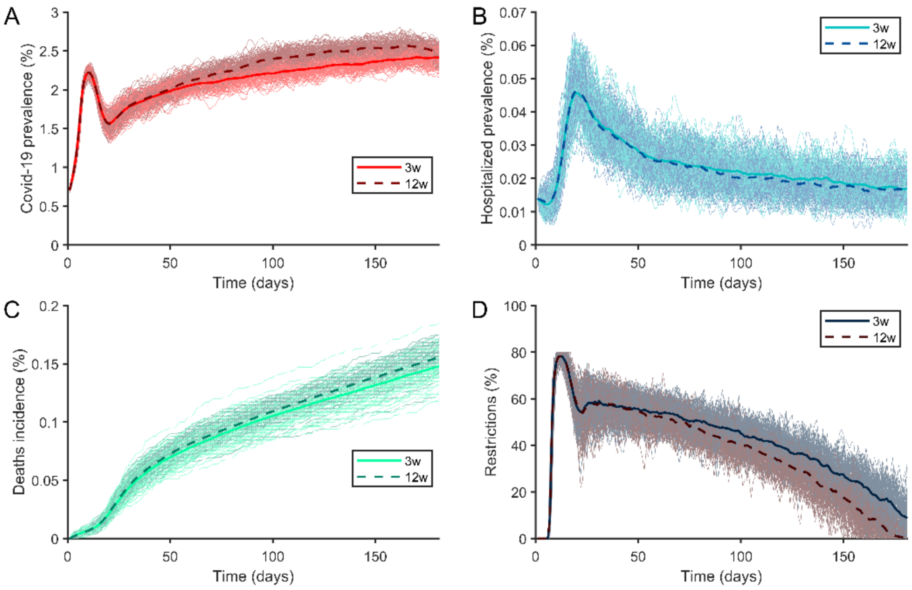

Figure 18. Output from the agent based model for the impact of COVID-19 in Europe using AztraZeneca vaccines with between-dose intervals of 3 vs 12 weeks. Vaccination speed: 0.4%, other parameters with their default value.

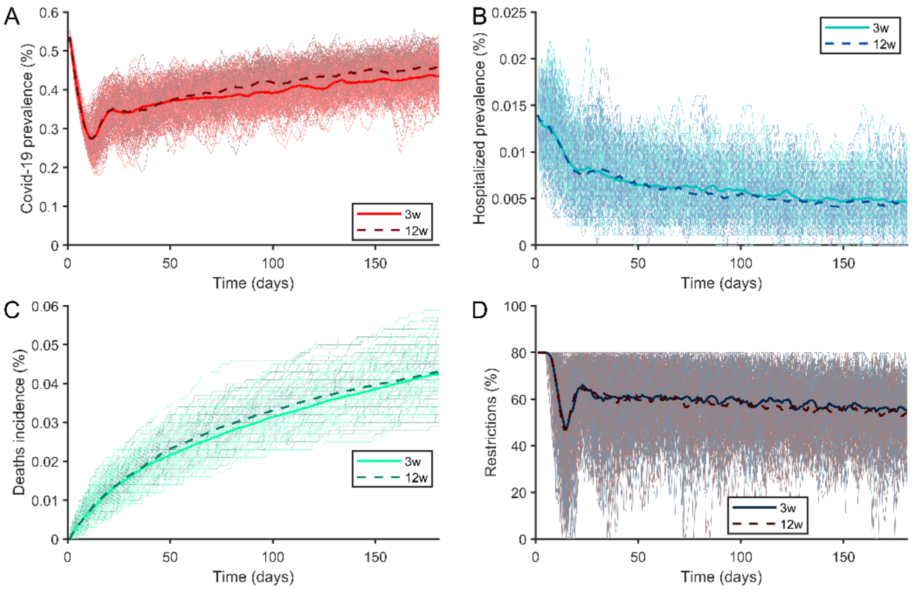

Figure 19. Output from the agent based model for the impact of COVID-19 in Europe using AztraZeneca vaccines with between-dose intervals of 3 vs 12 weeks. Hospital capacity: 100 individual per million, other parameters with their default value.

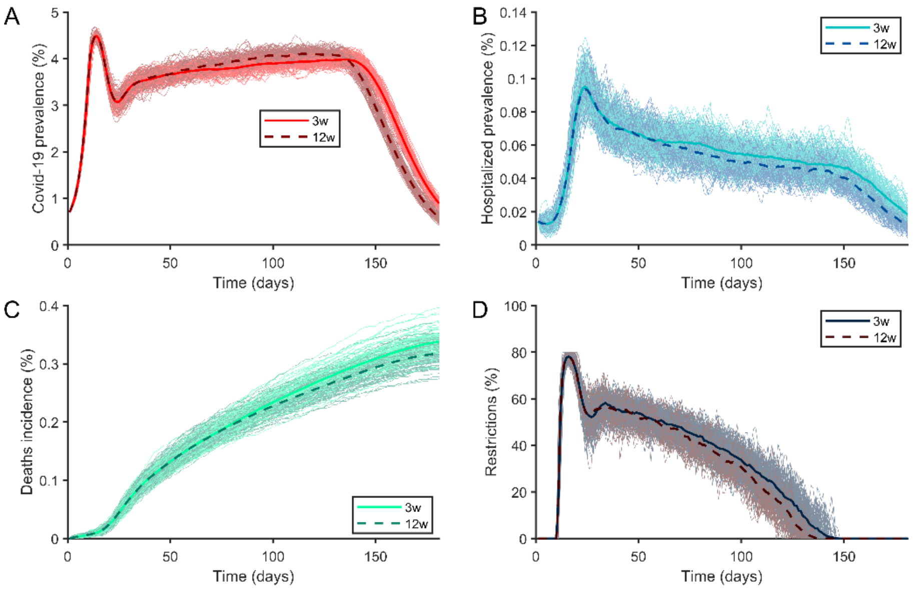

Figure 20. Output from the agent based model for the impact of COVID-19 in Europe using AztraZeneca vaccines with between-dose intervals of 3 vs 12 weeks. Hospital capacity: 1000 individual per million, other parameters with their default value.

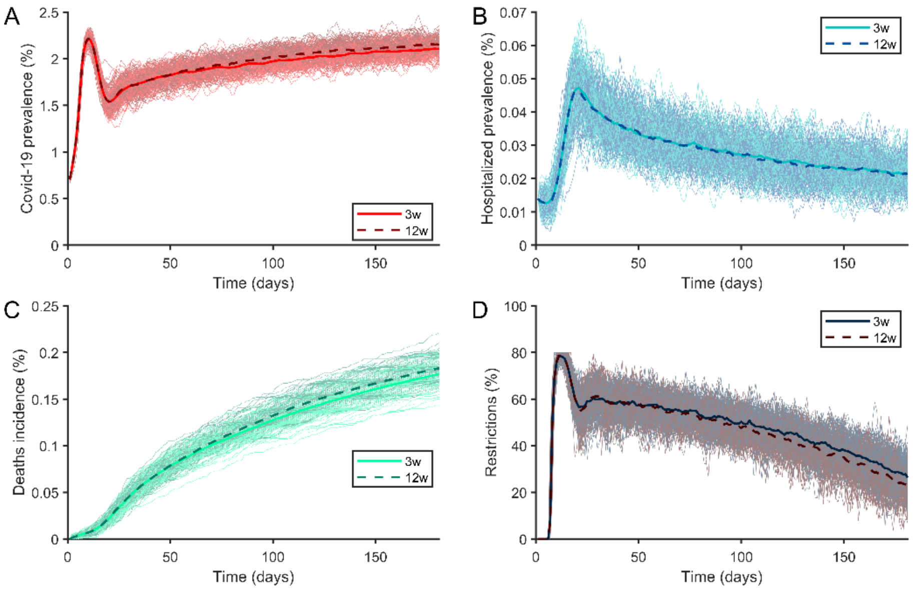

Figure 21. Output from the agent based model for the impact of COVID-19 in Europe using Moderna vaccines with between-dose intervals of 3 vs 12 weeks. Using default values for all parameters.

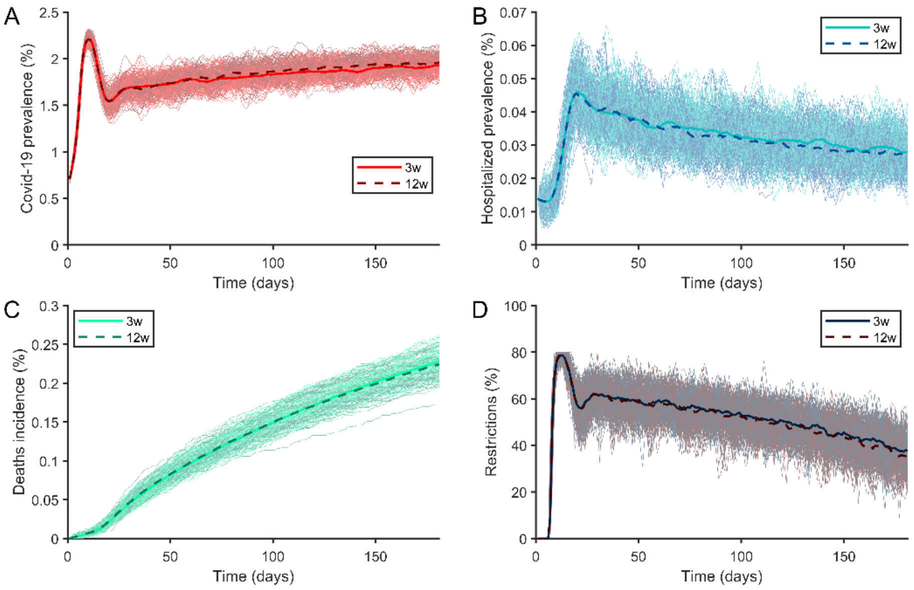

Figure 22. Output from the agent based model for the impact of COVID-19 in Europe using Moderna vaccines with between-dose intervals of 3 vs 12 weeks. Vaccination speed: 0.1%, other parameters with their default value.

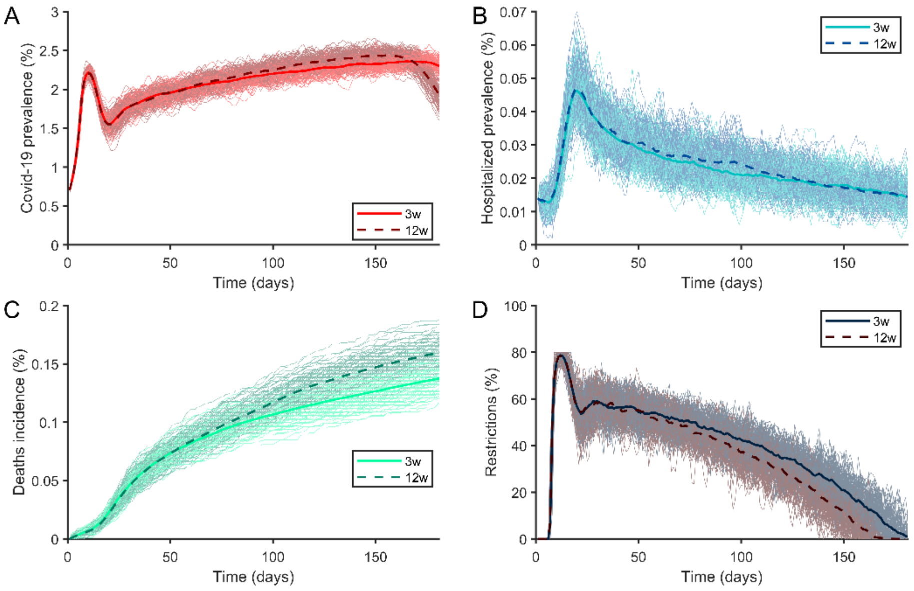

Figure 23. Output from the agent based model for the impact of COVID-19 in Europe using Moderna vaccines with between-dose intervals of 3 vs 12 weeks. Vaccination speed: 0.4%, other parameters with their default value.

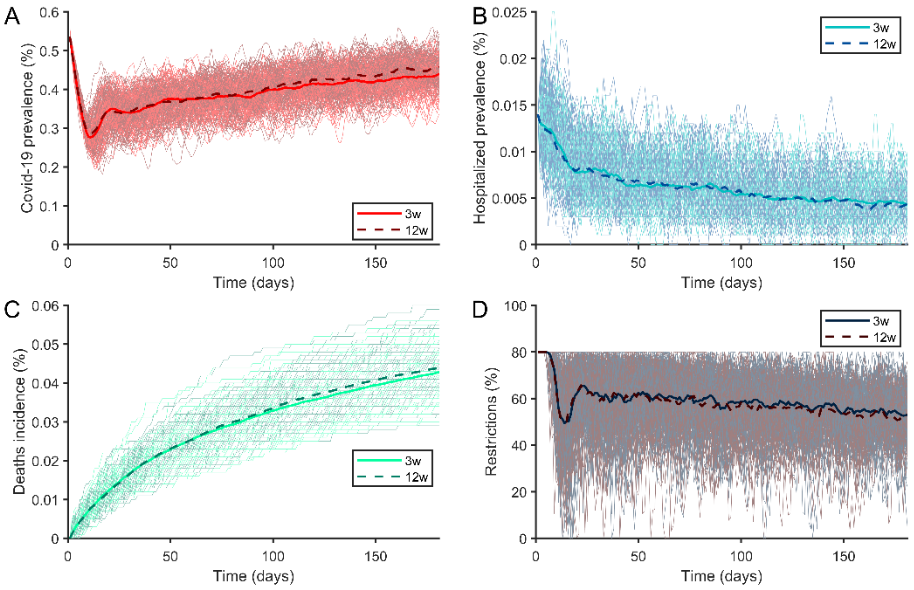

Figure 24. Output from the agent based model for the impact of COVID-19 in Europe using Moderna vaccines with between-dose intervals of 3 vs 12 weeks. Hospital capacity: 100 individual per million, other parameters with their default value.

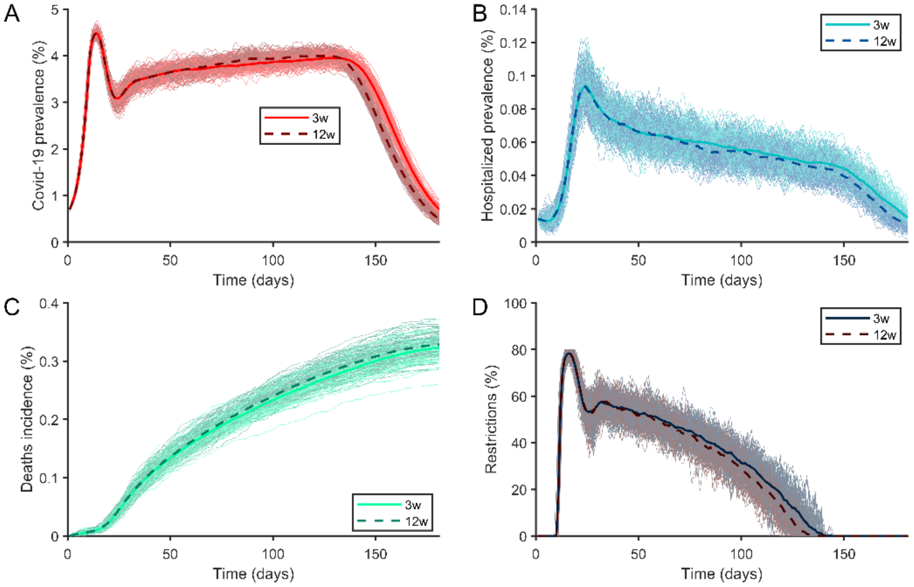

Figure 25. Output from the agent based model for the impact of COVID-19 in Europe using Moderna vaccines with between-dose intervals of 3 vs 12 weeks. Hospital capacity: 1000 individual per million, other parameters with their default value.

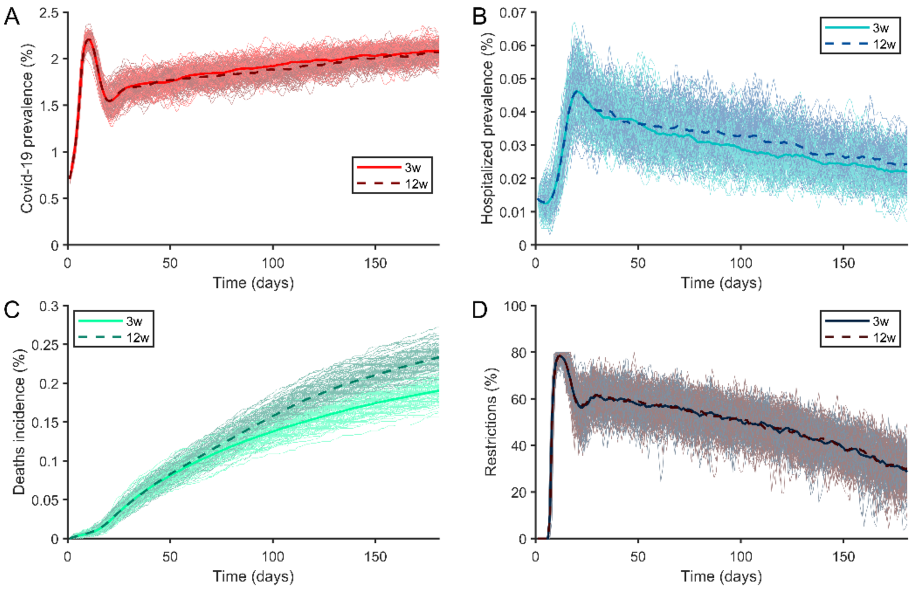

Figure 26. Output from the agent based model for the impact of COVID-19 in Europe using Pfizer vaccines with between-dose intervals of 3 vs 12 weeks. Using default values for all parameters.

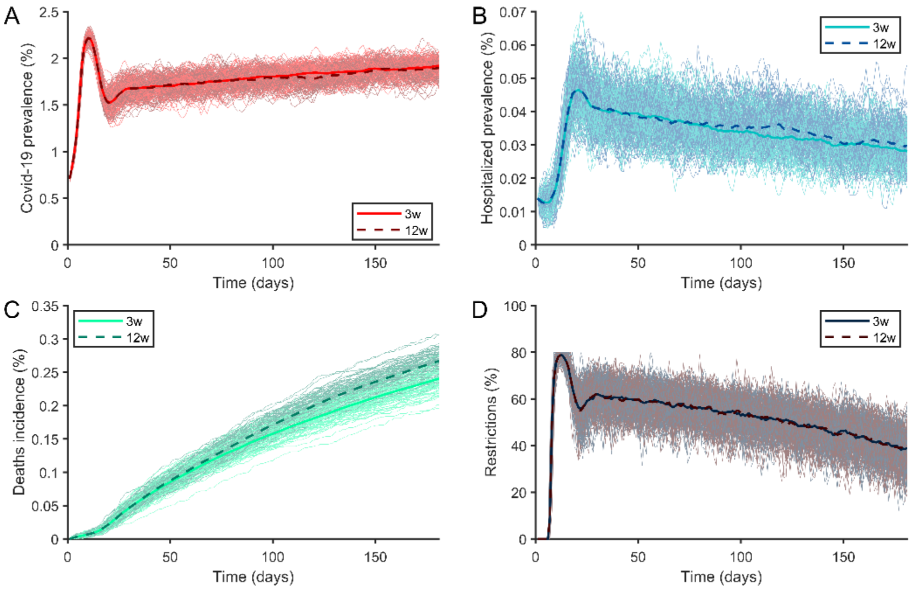

Figure 27. Output from the agent based model for the impact of COVID-19 in Europe using Pfizer vaccines with between-dose intervals of 3 vs 12 weeks. Vaccination speed: 0.1%, other parameters with their default value.

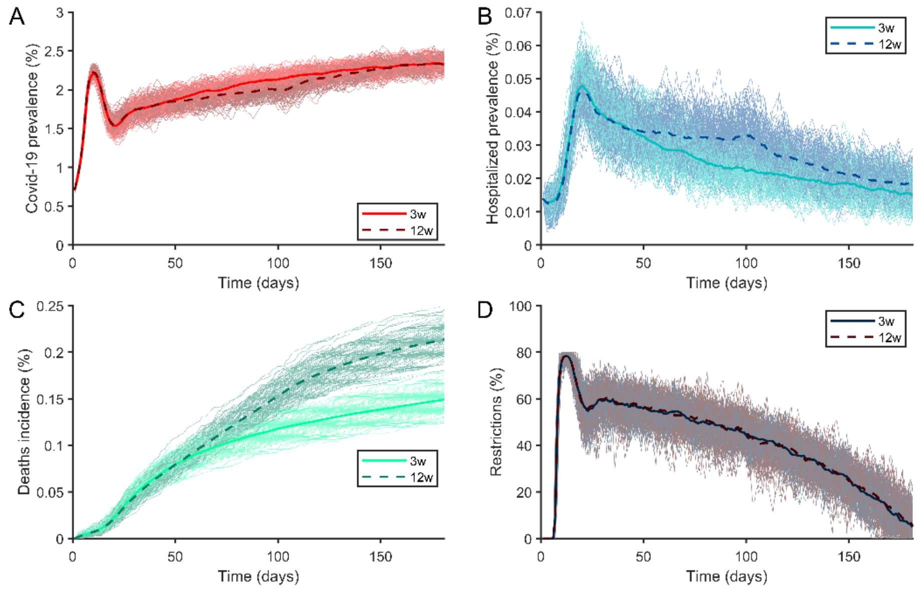

Figure 28. Output from the agent based model for the impact of COVID-19 in Europe using Pfizer vaccines with between-dose intervals of 3 vs 12 weeks. Vaccination speed: 0.4%, other parameters with their default value.

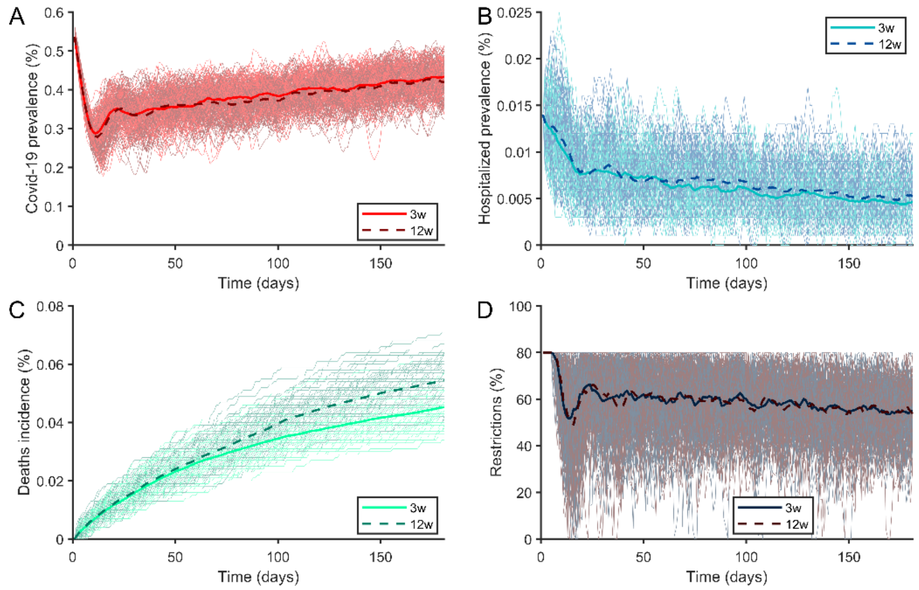

Figure 29. Output from the agent based model for the impact of COVID-19 in Europe using Pfizer vaccines with between-dose intervals of 3 vs 12 weeks. Hospital capacity: 100 individual per million, other parameters with their default value.

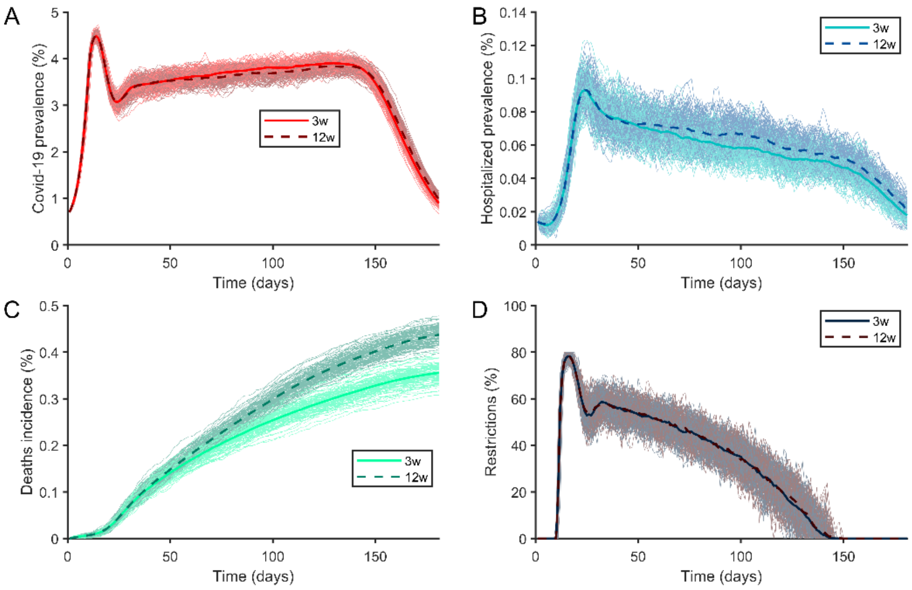

Figure 30. Output from the agent based model for the impact of COVID-19 in Europe using Pfizer vaccines with between-dose intervals of 3 vs 12 weeks. Hospital capacity: 1000 individual per million, other parameters with their default value.

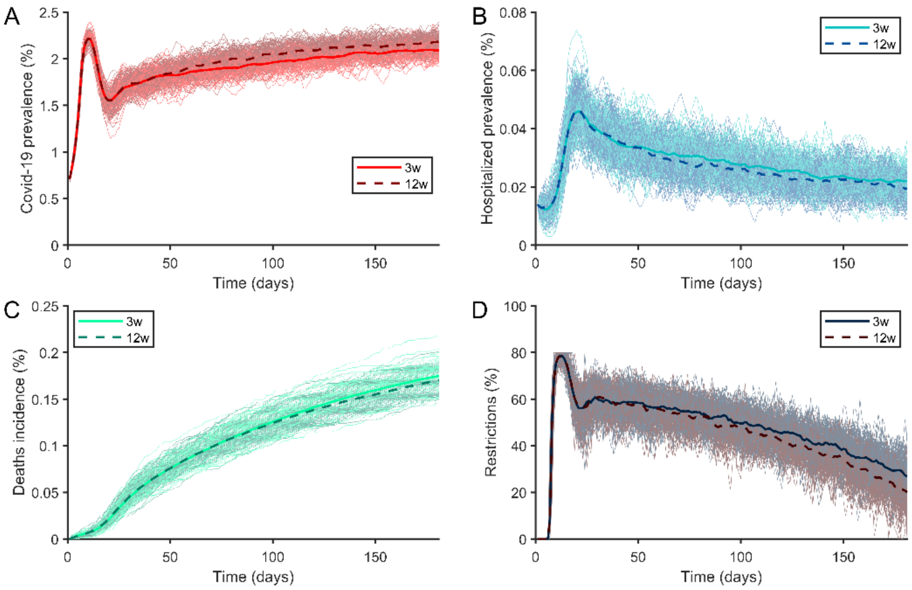

Figure 31. Output from the agent based model for the impact of COVID-19 in Europe using Pfizer vaccines with between-dose intervals of 3 vs 12 weeks. Efficacy of first dose was considered of 90%.

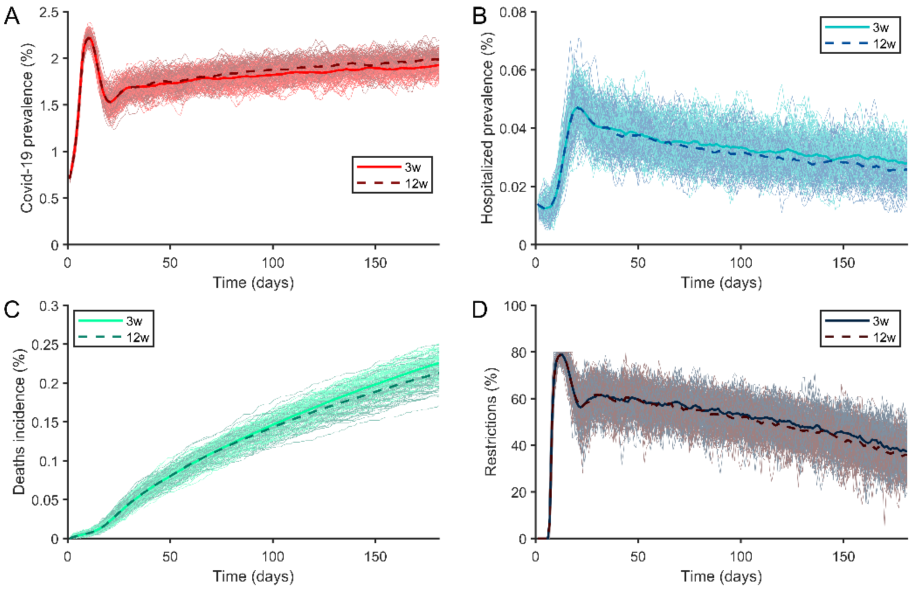

Figure 32. Output from the agent based model for the impact of COVID-19 in Europe using Pfizer vaccines with between-dose intervals of 3 vs 12 weeks. Efficacy of first dose was considered of 90%. Vaccination speed: 0.1%.

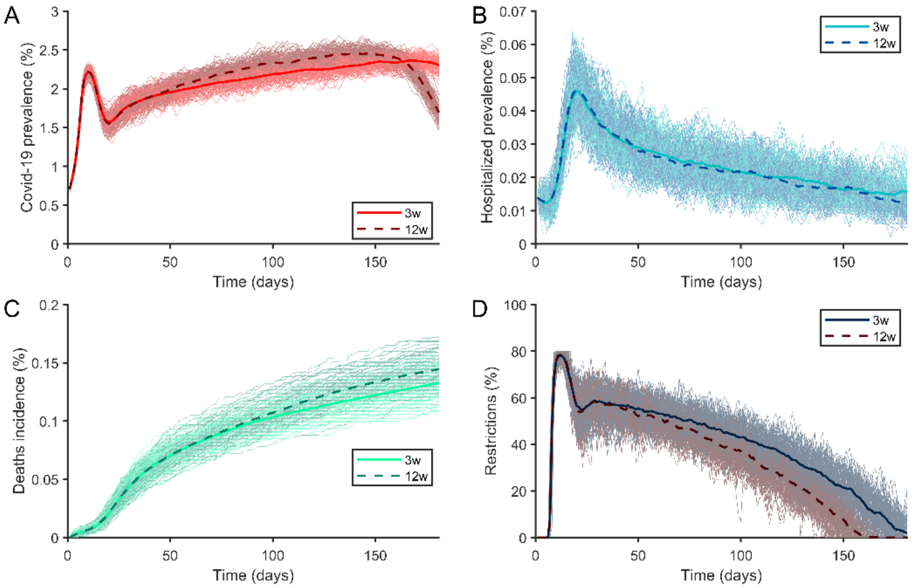

Figure 33. Output from the agent based model for the impact of COVID-19 in Europe using Pfizer vaccines with between-dose intervals of 3 vs 12 weeks. Efficacy of first dose was considered of 90%. Vaccination speed: 0.4%.

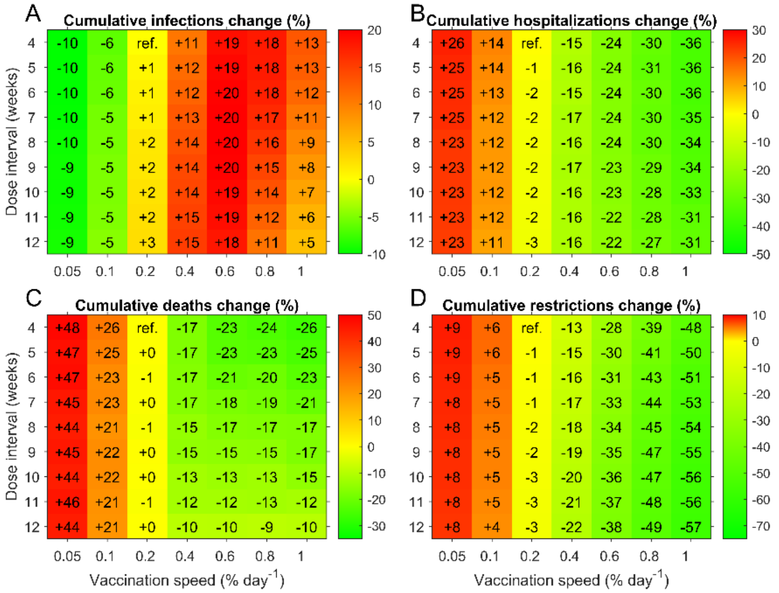

Figure 34. Heatmaps showing the impact of dose intervals and vaccination speed on infections (A), hospital admissions (B), mortality (C), and AUC of NPI (D). AztraZeneca vaccine efficacy data and 4-week dose interval anc vaccination speed of 0.2% are used as a reference (ref). The increase or decrease is given in percentage with respect to the reference scenario.

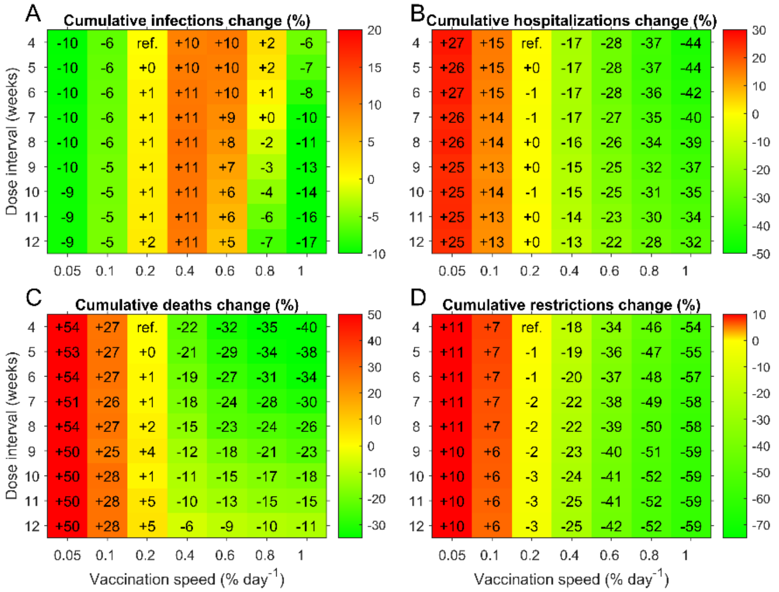

Figure 35. Heatmaps showing the impact of dose intervals and vaccination speed on infections (A), hospital admissions (B), mortality (C), and AUC of NPI (D). Moderna vaccine efficacy data and 4-week dose interval anc vaccination speed of 0.2% are used as a reference (ref). The increase or decrease is given in percentage with respect to the reference scenario.

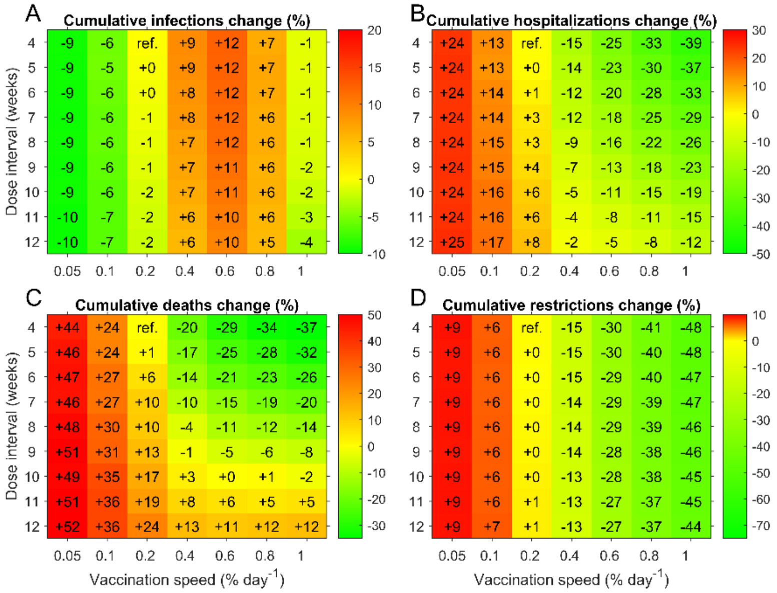

Figure 36. Heatmaps showing the impact of dose intervals and vaccination speed on infections (A), hospital admissions (B), mortality (C), and AUC of NPI (D). Pfizer vaccine efficacy data and 4-week dose interval anc vaccination speed of 0.2% are used as a reference (ref). The increase or decrease is given in percentage with respect to the reference scenario.

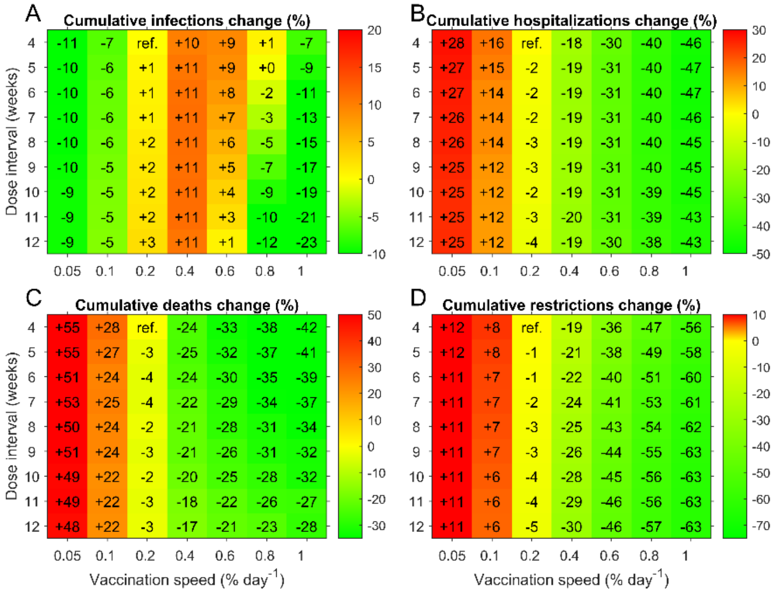

Figure 37. Heatmaps showing the impact of dose intervals and vaccination speed on infections (A), hospital admissions (B), mortality (C), and AUC of NPI (D). Pfizer vaccine efficacy data assuming a 90% first dose efficacy and 4-week dose interval anc vaccination speed of 0.2% are used as a reference (ref). The increase or decrease is given in percentage with respect to the reference scenario.

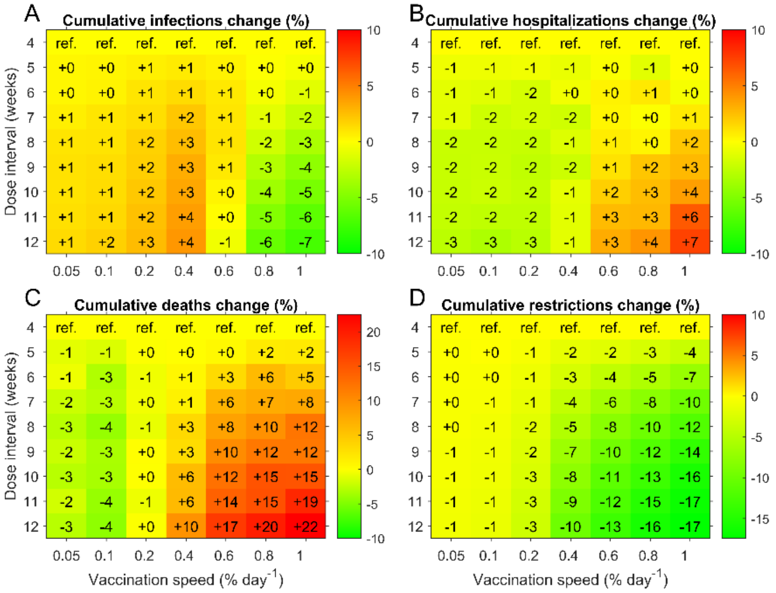

Figure 38. Heatmaps showing the impact of dose intervals and vaccination speed on infections (A), hospital admissions (B), mortality (C), and AUC of NPI (D). AztraZeneca vaccine efficacy data and 4-week dose interval are used as a reference (ref). The increase or decrease is given in percentage with respect to the reference scenario.

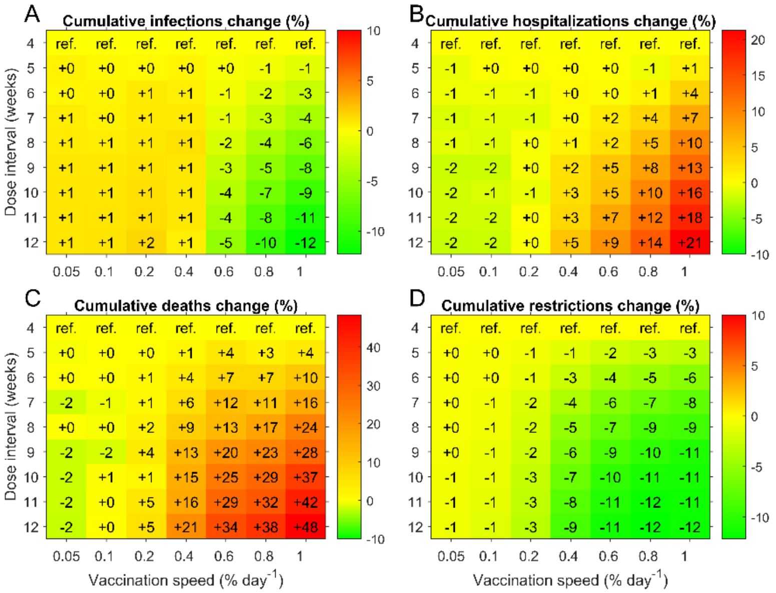

Figure 39. Heatmaps showing the impact of dose intervals and vaccination speed on infections (A), hospital admissions (B), mortality (C), and AUC of NPI (D). Moderna vaccine efficacy data and 4-week dose interval are used as a reference (ref). The increase or decrease is given in percentage with respect to the reference scenario.

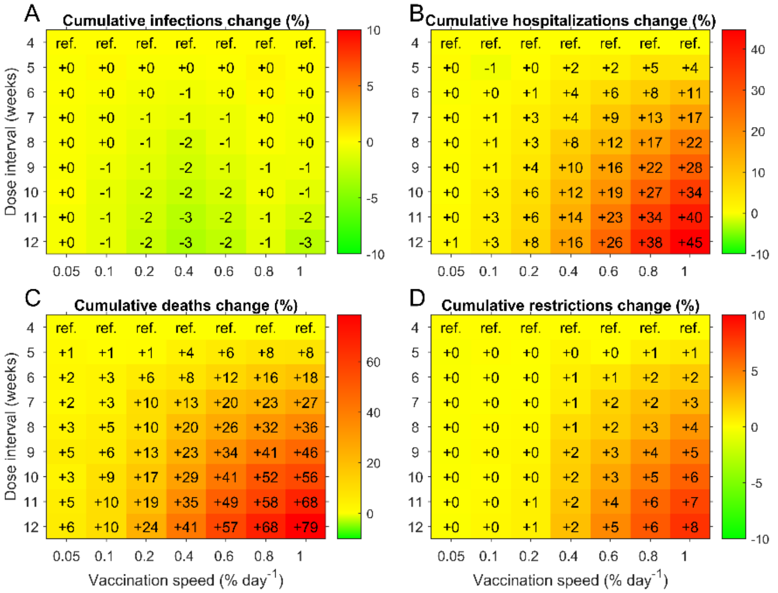

Figure 40. Heatmaps showing the impact of dose intervals and vaccination speed on infections (A), hospital admissions (B), mortality (C), and AUC of NPI (D). Pfizer vaccine efficacy data and 4-week dose interval are used as a reference (ref). The increase or decrease is given in percentage with respect to the reference scenario.

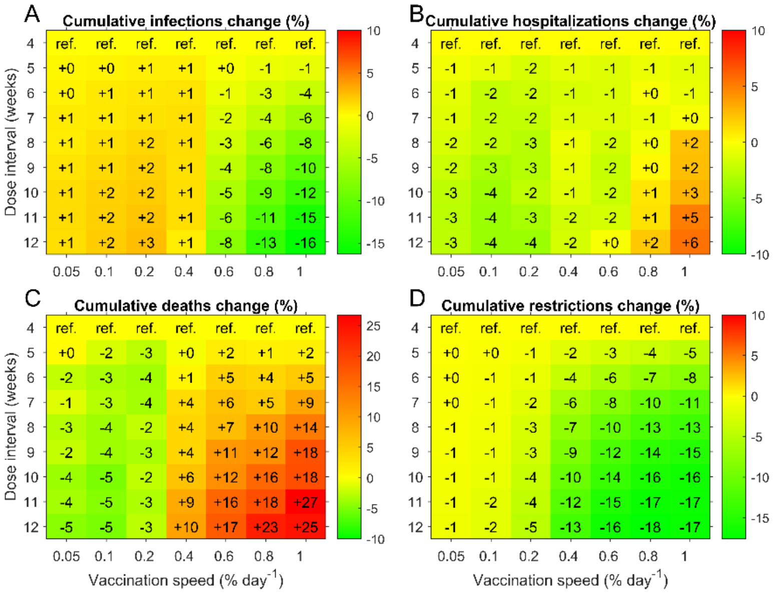

Figure 41. Heatmaps showing the impact of dose intervals and vaccination speed on infections (A), hospital admissions (B), mortality (C), and AUC of NPI (D). Pfizer vaccine efficacy data assuming a 90% first dose efficacy and 4-week dose interval are used as a reference (ref). The increase or decrease is given in percentage with respect to the reference scenario.
